# Balsalazide-Derived Heterotriaryls as Sirtuin 5 Inhibitors: A Case Study of a Reversible Covalent Inhibition Strategy

**DOI:** 10.3390/molecules30183821

**Published:** 2025-09-20

**Authors:** Ricky Wirawan, Simon A. Huber, Thomas Wein, Franz Bracher

**Affiliations:** Department of Pharmacy—Center for Drug Research, Ludwig-Maximilians University, Butenandtstr. 5-13, 81377 Munich, Germany; ricky.wirawan@cup.uni-muenchen.de (R.W.);

**Keywords:** histone deacetylases, sirtuin 5 inhibitor, Sirt5 inhibitor, balsalazide, structure–activity relationship, reversible covalent inhibitor, co-factor NAD^+^

## Abstract

Sirtuin 5 is an NAD^+^-dependent lysine deacylase that is involved in various biological processes and has emerged as a promising target for pharmaceutical therapies. The development of highly potent and subtype-selective sirtuin 5 inhibitors for their application as chemical tools and drug candidates still poses a significant challenge. Based on our own optimized balsalazide-derived sirtuin 5 inhibitors, this work presents a systematic investigation of the inhibitory effects of derivatives with moieties that were guided by docking experiments to target the nicotinamide ribose vicinal hydroxy groups of the essential co-factor NAD^+^ via reversible covalent binding to potentially enhance their potency. Our results show that functionalizations with these moieties were tolerated to some extent and possessed a distinct stereo-selective preference. The (*S*)-configured cyanomethyl derivative **50** with an IC_50_ of 27 µM emerged from our synthesized library of compounds as the most potent functionalized inhibitor and lies in a similar potency range to other established sirtuin 5 inhibitors. Our findings offer a deeper insight into the structure–activity relationships of our balsalazide-derived heterotriaryl-based sirtuin 5 inhibitors and thus could provide an avenue for further optimizations in the future.

## 1. Introduction

**Figure 1:**
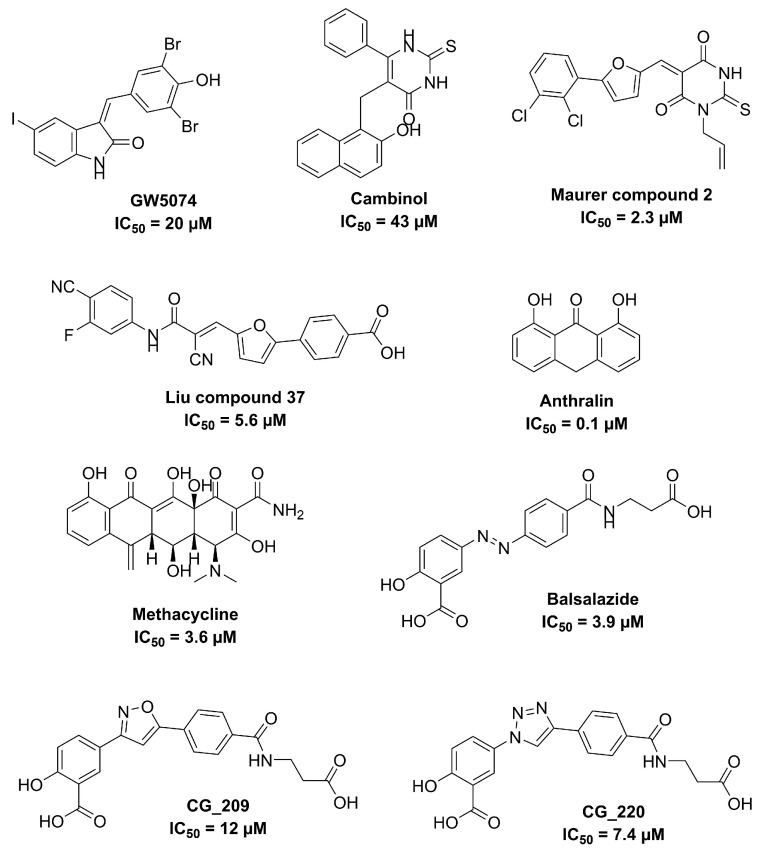
Selected examples of published sirtuin 5 inhibitors.

Sirtuins, belonging to the unique class III histone deacetylases, represent highly conserved NAD^+^-dependent enzymes [1]. In mammals, seven different sirtuin subtypes were identified, each with its own distinct function and subcellular localization [2]. As a protein that predominantly resides in the mitochondria, sirtuin 5 has gained significant interest over the years due to its governing role in various metabolic processes such as ammonia detoxification [3], ROS elimination [4], β-oxidation of fatty acids [5], glycolysis [6] and ketogenesis [7]. Dysregulation of sirtuin 5 has been implicated with the progression and exacerbation of numerous diseases, including metabolic disorders [8], neurodegeneration [9,10] and cancer [11,12,13]. Thus, sirtuin 5 presents itself as a promising biological target for pharmaceutical interventions. Extensive efforts were undertaken over the years to develop novel and effective Sirt5 inhibitors. However, most of them still suffer from sub-par potency, lack of subtype selectivity and poor pharmacokinetic properties, signifying the challenges and obstacles associated with the development of drug-like sirtuin 5 inhibitors. Representative potent sirtuin 5 inhibitors include the indolinone GW5074 [14,15], the β-naphthol-based cambinol [16] and Maurer et al. compound **2** [16] (Figure 1). However, these compounds also exhibit comparable inhibitory potency against other sirtuin subtypes such as sirtuin 2. Additionally, these inhibitors, as well as Liu et al. compound **37** [17], may exhibit promiscuity to other biological targets due to highly reactive motifs such as Michael acceptors and alkylidene thiobarbiturates that are typical in false-positive pan-assay interference compounds (PAINS) [18]. Although approved drugs such as anthralin, methacycline and balsalazide emerged as sirtuin 5 inhibitors in a high-throughput screening [19], their drug repurposing potential is limited due to significant liabilities associated with toxicity (anthralin), instability (anthralin), unwanted antimicrobial effects (methacycline) and suboptimal pharmacokinetic properties (balsalazide) [20,21]. For the latter, however, optimization efforts that involve the substitution of the gut-bacteria labile azo bond of balsalazide with metabolically more stable heteroaromatic rings such as isoxazole (**CG_209**) and triazole (**CG_220**) have been successfully conducted in our previous work [21,22].

**Figure 2:**
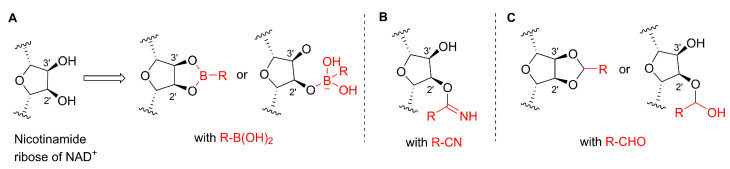
Proposed binding mode of the selected reversible covalent warheads with the nicotinamide ribose of the co-factor NAD^+^. Boronic acids (**A**) and aldehydes (**C**) can react with one or both hydroxy groups to form (cyclic) boronates, and hemiacetals or cyclic acetals. Nitriles (**B**) can react with the more reactive 2′-OH to form iminoethers. Reversible covalent inhibition strategy blends the benefits of both irreversible covalent inhibition and non-covalent inhibition by prolonging inhibition and thus potency, while maintaining reversibility that reduces off-target toxicity.

In continuation of our work on the optimization of balsalazide as a drug-like sirtuin 5 inhibitor through comprehensive SAR studies [21,22] and our work on developing reversible covalent sirtuin inhibitors [23], we sought to explore the potential of reversible covalent inhibition as a tool to further improve the potency of our subtype selective sirtuin 5 inhibitors **CG_209** and **CG_220**. Reversible covalent inhibition with suitable functional groups has seen notable success in drug development as exemplified by the boronic acid-containing bortezomib in the treatment of multiple myeloma [24,25], the nitrile-based saxagliptin for the treatment of diabetes mellitus type II [26], and the aldehyde-containing sickle cell anemia drug voxelotor [27]. Nitriles can undergo reversible covalent bonding with the more reactive 2′-OH of the nicotinamide ribose based on the mechanistic insight of sirtuin catalysis [1], while boronic acids and aldehydes can additionally react with the 3′-OH nicotinamide ribose to form cyclic boronates and acetals (Figure 2). Reversible covalent inhibitors have the advantage of prolonged inhibition and thus potency compared to non-covalent inhibitors, yet at the same time reduced off-target toxicity and immunogenicity compared to irreversible covalent inhibitors, making them promising candidates for drug development [28]. Based on our previous docking experiments of balsalazide in sirtuin 5 [21], the position of balsalazide was shown to be in spatial proximity to the nicotinamide ribose ring of the co-factor NAD^+^. These findings suggested the potential of the yet-unexploited co-factor NAD^+^ as a site for additional binding interactions. A similar strategy has been executed to great success in the other sirtuin subtypes such as the development of mechanism-based sirtuin 2 inhibitors that yielded nanomolar potency [29]. However, to the best of our knowledge, this approach has never been investigated before in the field of sirtuin 5. Furthermore, the application of reversible covalent inhibition in the development of sirtuin 5 inhibitors remains unexplored. Thus, this work focuses on the systematic investigation of rational modifications of **CG_209** and **CG_220** that employ various functional groups such as boronic acids, nitriles and aldehydes at appropriate positions on the inhibitor scaffolds to not only give further insight into their structure–activity relationships, but to perhaps also potentially improve their potency through their engagement in reversible covalent bonding with the nicotinamide ribose hydroxy groups of NAD^+^.

## 2. Results & Discussion

### 2.1. Design Rationale of Inhibitors via Docking Experiments

**Figure 3:**
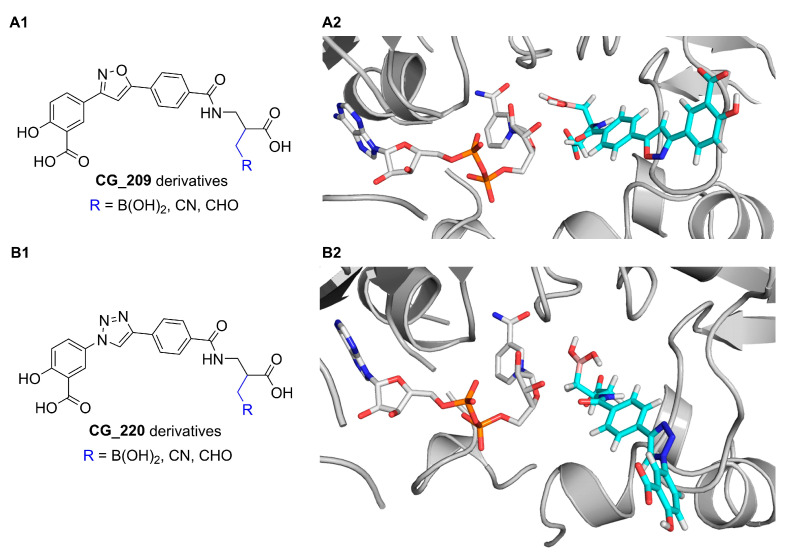
Target structures derived from **CG_209** (**A1**) and **CG_220** (**B1**). Docking experiments of the envisaged boronic acid derivatives of **CG_209** (cyan) (**A2**) and **CG_220** (cyan) (**B2**) in the presence of the co-factor NAD^+^ (grey) (based on PDB ID: 3RIY [30]). Modifications with further functional groups such as nitriles and aldehydes were additionally chosen as suitable moieties for reversible covalent binding. Colors: oxygen (red), nitrogen (blue), phosphorus (orange).

Initial docking experiments with the envisaged boronic acid derivatives in sirtuin 5 were performed to give an insight into their potential binding poses (Figure 3). The results suggested the α-carbon atom to the essential terminal carboxylic acid as the appropriate position for functional group modifications to target the nicotinamide ribose hydroxy groups of the co-factor NAD^+^. Notably, the docking calculations also predicted the requirement of an additional methylene group for optimal spacing of the modifications to allow reversible covalent bonding with the nicotinamide ribose hydroxy groups. Acknowledging the presence of a newly introduced chiral center involved in the functional group modifications, syntheses and subsequent biological evaluation of enantiomerically pure derivatives will additionally provide insight, if any, into the stereospecific preference of these moieties in the active site.

### 2.2. Chemistry

**Scheme 1:**
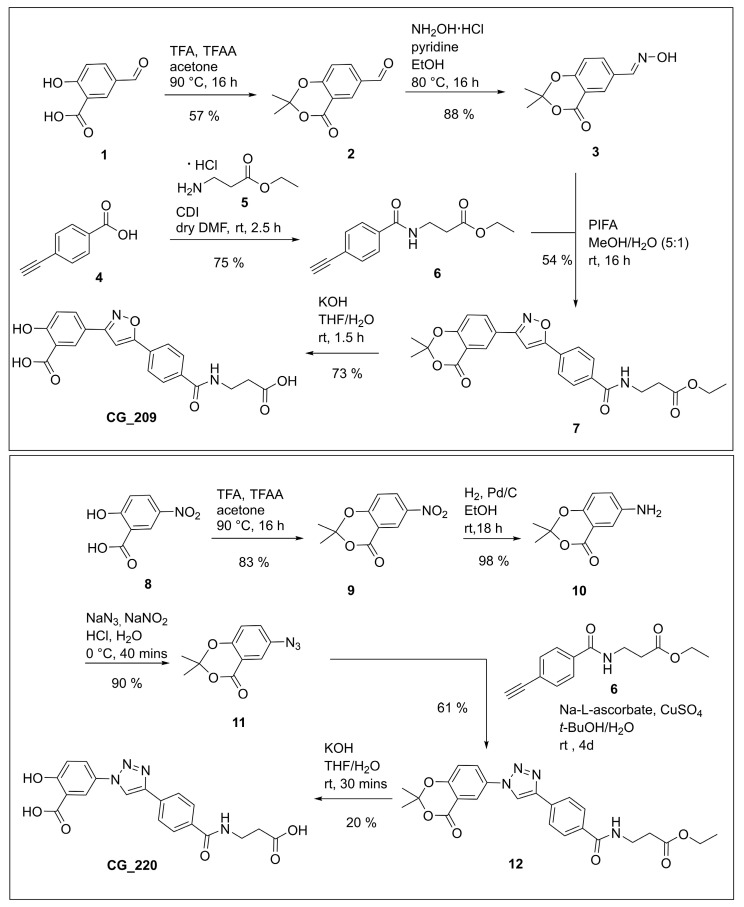
Synthesis of lead structures **CG_209** and **CG_220** according to published literature with slight modifications, particularly in the preparation of the amide **6**.

The syntheses of lead structures **CG_209** and **CG_220** were generally performed following literature procedures [22] with slight modifications, particularly in the preparation of the benzamide **6** (Scheme 1). Instead of the reported three-step procedure [22], the amide **6** was synthesized directly from 4-ethynylbenzoic acid (**4**) and β-alanine ethyl ester hydrochloride (**5**) with CDI as an amide coupling reagent. For the preparation of the isoxazole **CG_209**, the salicylic acid moiety of 5-formylsalicylic acid (**1**) was first protected with acetone to give the acetonide **2**. Aldoxime **3** was then synthesized from acetonide **2** with hydroxylamine hydrochloride. PIFA-mediated oxidation of the aldoxime **3** to the nitrile oxide intermediate allowed the 1,3-dipolar cycloaddition with alkyne **6** to yield isoxazole **7**. Subsequent saponification of the acetonide and the ethyl ester gave lead structure **CG_209** in 73% yield. Similarly, the preparation of triazole **CG_220** involved the initial protection of 5-nitrosalicylic acid (**8**) to give acetonide **9**. The nitroarene **9** was then reduced to the aniline **10** and subsequently, after diazotation, converted to azide **11** with sodium azide. The copper catalyzed azide-alkyne cycloaddition (CuAAC) between azide **11** and alkyne **6** then afforded triazole **12**. Finally, dual protective group saponification led to the formation of lead structure **CG_220**.

**Scheme 2:**
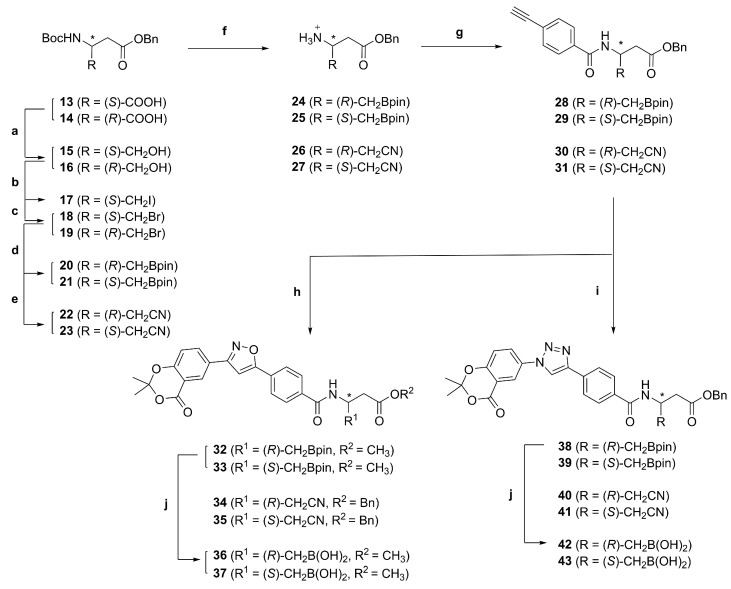
Reagents, conditions and yields: (a) ethyl chloroformate, Et_3_N, THF, 0 °C, 15 min, then NaBH_4_, H_2_O, rt, 1 h, 70% for **15** and 68% for **16**; (b) PPh_3_, I_2_, imidazole, DCM, rt, 2.5 h, 54%; (c) PPh_3_, CBr_4_, dry Et_2_O, rt, 15 min, 60% for **18** and 58% for **19**; (d) B_2_pin_2_, Pd_2_(dba)_3_, *t*-Bu_2_MeP·HBF_4_, K_2_CO_3_, *t*-BuOH/H_2_O (12:1 *v*/*v*), 60 °C, 6 h, 60% for **20** and 51% for **21**; (e) Bu_4_NCN, DCM, 55 °C, 17 h, 52% for **22** and 63% for **23**; (f) 4M HCl in dioxane, rt, 1 h, quant. for **24**, **25**, **26** and **27**; (g) 4-ethynylbenzoic acid (**4**), CDI, dry DMF, rt, 3 d, 80% for **28**, 64% for **29**, 58% for **30** and 71% for **31**; (h) aldoxime **3**, PIFA, MeOH/H_2_O (5:1 *v*/*v*), rt, 16 h, 31% for **32**, 22% for **33**, 14% for **34** and 24% for **35**; (i) azide **11**, Na-L-ascorbate, CuSO_4_, *t*-BuOH/H_2_O (21:1 *v*/*v*), rt, 18 h, 17% for **38**, 8% for **39**, 41% for **40**, 53% for **41**, 24% for **42** and 33% for **43**; (j) NaIO_4_, HCl, THF/H_2_O (4:1 *v*/*v*), rt, 16 h, 73% for **36**, 64% for **37** and 68% for **42**. * = chiral center.

The syntheses of the enantiomerically pure boronic acid and cyanomethyl derivatives were initiated by the reduction of 4-benzyl *N*-Boc-(*S*)- (**13**) and (*R*)- (**14**) aspartate to their corresponding primary alcohols **15** and **16** with ethyl chloroformate to initially generate the mixed anhydride intermediates and then sodium borohydride following procedures from Isernia et al. [31] (Scheme 2). Conversion of the primary alcohol **15** to the alkyl iodide **17** via an Appel reaction was performed with triphenylphosphine and iodine following a published protocol [31]. Similarly, bromination of alcohols **15** and **16** via an Appel reaction afforded the alkyl bromides **18** and **19** according to published work by Röhrich et al. [32]. The borylation of the alkyl bromides **18** and **19** was then successfully performed with B_2_pin_2_, Pd_2_(dba)_3_ catalyst and *t*-Bu_2_MeP·HBF_4_ ligand to give the boronic acid pinacol esters **20** and **21** in 60% and 51% yield, respectively.

Kolbe nitrile synthesis of the cyanomethyl compounds **22** and **23** from the alkyl bromides **18** and **19** was performed using Bu_4_NCN, inspired by a method from Isernia et al. [31].

**Scheme 3:**
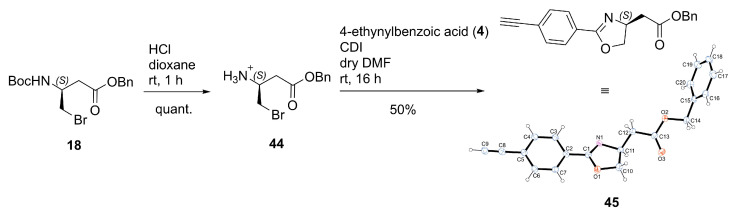
Formation of the 2-oxazoline side product **45**, most likely via the imidazole-catalyzed deprotonation of the amide hydrogen atom that leads to nucleophilic attack of the amide at the reactive alkyl bromide.

The *N*-Boc-protected derivatives **20**–**23** were then treated with HCl in dioxane following a procedure from Klein et al. [33] to give the primary amine hydrochlorides **24**–**27**, which were subsequently coupled with 4-ethynylbenzoic acid (**4**) utilizing CDI as an amide coupling reagent to give amides **28**–**31** in good yields. Of particular note was the formation of 2-oxazoline **45** as the main product when the bromo derivative **44** was subjected to amide coupling with 4-ethynylbenzoic acid (Scheme 3), suggesting that efficient introduction of the boronic acid pinacol ester and the nitrile moieties must be performed prior to the amide coupling as late-stage functionalization to the boronic acid pinacol esters or nitriles would no longer be possible with oxazoline **45**. Since the identity of the product was initially ambiguous, the X-ray crystal structure of compound **45** was solved to confirm its molecular structure (see Supplementary Materials). The duality of CDI as both an amide coupling reagent and subsequently a source of base in the form of imidazole allows the facile base-mediated formation of 2-oxazoline in a single step.

1,3-Dipolar cycloaddition of the alkynes **28**–**31** and the previously synthesized aldoxime **3** in a methanol–water mixture then afforded the isoxazoles **32**–**35**. Notably, transesterification of the benzyl ester with the solvent methanol also occurred in this reaction but was only limited to the boronic acid pinacol esters derivatives **32** and **33**, presumably due to the strong Lewis acidity of the boronic acid pinacol ester in the neighboring position to the benzyl ester that promotes this transesterification process. Nonetheless, the next synthetic steps were carried on since the benzyl ester (or the methyl ester) only served as a protecting group for the terminal carboxylic acid and will have to be hydrolyzed in the following steps. The syntheses of triazoles **38**–**41** were performed via CuAAC of alkynes **28**–**31** and the previously prepared azide **11**. Remarkably, mixtures of both the boronic acid pinacol esters and the free boronic acids were generated. For example, CuAAC of alkyne **28** and azide **11** yielded 17% of the boronic acid pinacol ester **38** and 24% of the boronic acid **42**, while CuAAC of alkyne **29** and azide **11** yielded 8% of the boronic acid pinacol ester **39** and 33% of the boronic acid **43.** Subsequent oxidative cleavage of the boronic acid pinacol esters **32**, **33** and **38** with sodium periodate gave boronic acids **36**, **37** and **42** in satisfactory yields.

The borylation step involving the conversion of alkyl bromides **18** and **19** to the boronic acid pinacol esters **20** and **21** represents a pivotal segment in the syntheses of the envisaged boronic acid derivatives and required several rounds of optimizations in the reagents, reaction conditions and purification methods used (Table 1), all of which are listed here as entries in Table 1 and were inspired from published methods that were specifically developed for the preparation of alkyl boronic acids and esters thereof. Alkyl iodide **17** was initially subjected to borylation utilizing CuI as catalyst, triphenylphosphine as ligand and lithium methanolate as a base (entry 1 [34]), but this approach was unsuccessful. Replacement of the triphenylphosphine ligand with another phosphine-based ligand Xantphos and the base with potassium *tert*-butoxide with reactions performed both at room temperature (entry 2 [35]) and at elevated temperature (entry 3) likewise showed no success.

Substitution of the transition metal catalyst to Ni(II) in combination with a chiral pyridine bisoxazoline ligand led instead to the undesired dehalogenation of alkyl iodide **17** in 33% yield (entry 4). Such undesired dehalogenation was notably not observed in the original study where this method was developed [36]. Alternatively, utilization of Pd_2_(dba)_3_ catalyst and *t*-Bu_2_MeP·HBF_4_ ligand with potassium carbonate as a base was performed in a solvent mixture of *tert*-butanol and water and gave trace amounts of the product (entry 5 [37]). Similarly, traces of the product were obtained when tetrabutylammonium iodide was additionally employed with the reagents listed in entry 1 (entry 6 [38]). However, applying the exact same reagents and conditions from entry 6 with alkyl bromide **18** as the starting material, a significant increase in the yield was obtained, and the desired pinacol ester product **20** could be isolated in 10% yield (entry 7 [38]). When the reagents and the reaction conditions from entry 1 were repeated with alkyl bromide **18**, still no desired product **20** was formed (entry 8 [34]), highlighting the importance of tetrabutylammonium iodide in entry 7. Entry 5, which initially afforded only traces amount of the desired product **20**, was repeated using alkyl bromide **18**, yielding 16% of the desired boronic acid pinacol ester **20** (entry 9 [37]). This suggests that alkyl bromide **18** is better suited than iodide **17** in the borylation, likely due to the latter’s lower stability in the given reaction conditions. The reaction time in entry 9 was then systematically varied to optimize the yield of product **20**, with 6 h identified as optimal despite incomplete consumption of starting material **18** (entry 10a). Finally, purification of the boronic acid pinacol ester **20** using boric acid-impregnated silica gel was performed, which was prepared as described by Hitosugi et al. [39]. This further improved the yield to 60% (entry 10b) due to the reduction of the Lewis basicity of the silica gel and thus the over-adsorption of the boronic acid pinacol ester **20** to the silica gel, a common problem in the purification of boronic acids and esters [39].

**Scheme 4:**
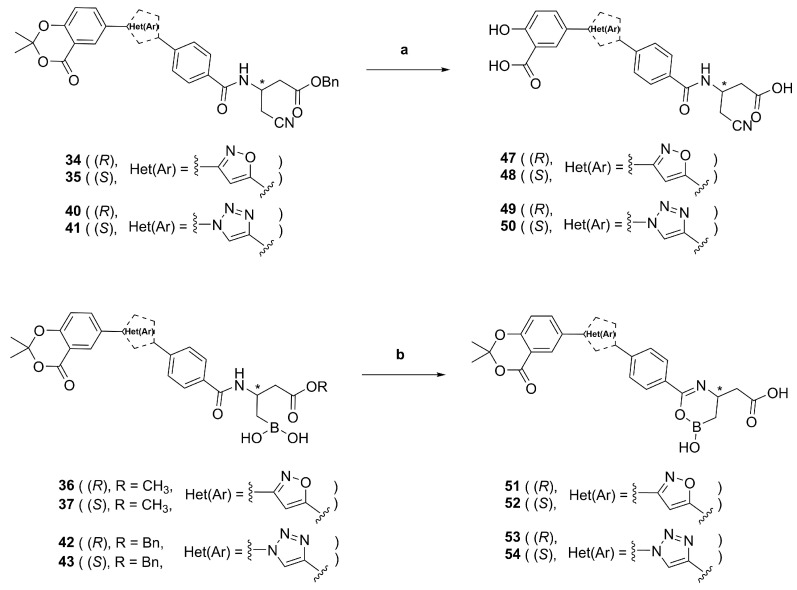
Reagents, reactions conditions and yields: (a) KOH, THF, rt, 0.5–2.5 h, 50% for **47**, 55% for **48**, 39% for **49** and 58% for **50**; (b) KOH, THF, 0 °C, 0.5–1 h, 54% for **51**, 56% for **52**, 39% for **53** and 32% for **54**. * = chiral center.

In the final step, the isoxazole cyanomethyl compounds **34** and **35** and the triazole cyanomethyl compounds **40** and **41** were subjected to saponification to afford the desired enantiomerically pure cyanomethylated isoxazoles **47** and **48** and triazoles **49** and **50** (Scheme 4). Similarly, the saponification of the boronic acids **36**, **37**, **42** and **43** was performed, albeit selective to the terminal methyl or benzyl ester to prevent the degradation of the boronic acids that stems from the inter- or intramolecular salicylic acid-boronic acid reactions. Selective saponification of the terminal methyl or benzyl ester was achieved by performing the reaction at 0 °C. Notably, in the presence of a base the boronic acids react intramolecularly with the neighboring amide moiety to form the cyclic boronates **51**–**54**. All attempts at deprotection of the acetonides to free salicylates led to very unstable products, and not even traces of the target products could be isolated.

**Scheme 5:**
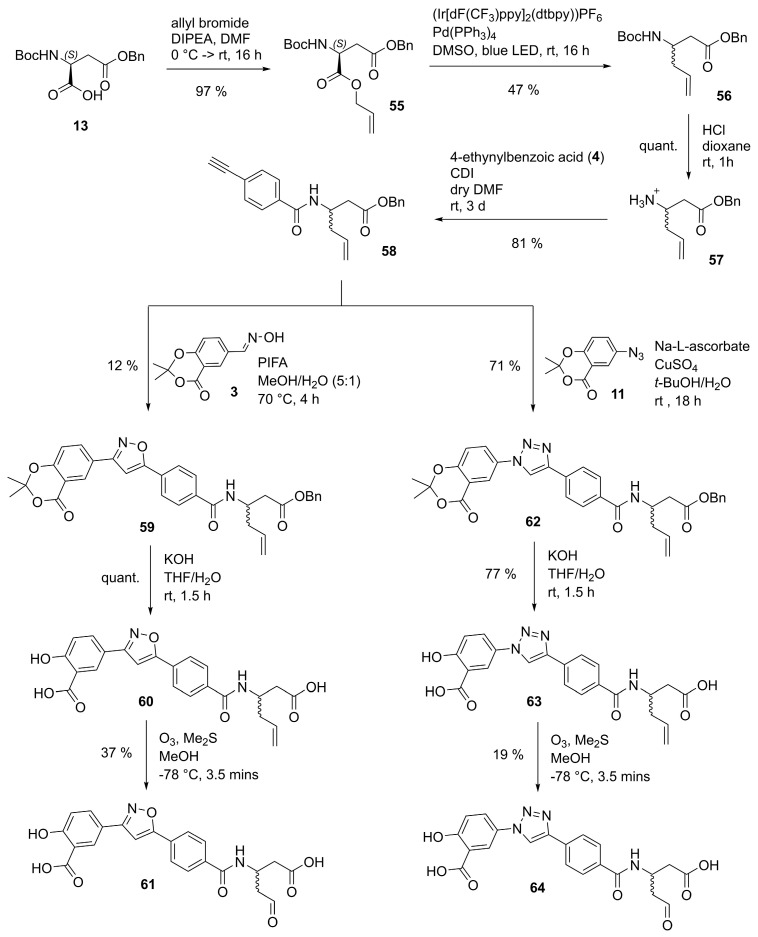
Syntheses of aldehydes **61** and **64**.

The preparation of the corresponding aldehyde derivatives was initially attempted by chemoselective reduction of the nitrile intermediates (**34**, **35**, **40**, **41**), but disappointing results were obtained. So we moved to an alternative approach, which was initiated with the formation of the allyl ester **55** from carboxylic acid **13** with allyl bromide and diisopropylethylamine following published protocols [40]. Subsequent blue LED-mediated radical decarboxylation of allyl ester **55** to form a racemic mixture of the *N*-Boc-protected homoallylic amine **56** was accomplished with a combination of palladium and iridium-photoredox catalyst following a protocol published by Lang et al. [40]. *N*-Boc deprotection with HCl in dioxane gave primary amine **57**, and subsequent amide coupling with 4-ethynylbenzoic acid (**4**) then afforded amide **58** in 81% yield. 1,3-Dipolar cycloaddition of alkyne **58** with aldoxime **3** and CuAAC of alkyne **58** with azide **11** gave isoxazole **59** and triazole **62**, respectively. The saponification of the acetonide and benzyl ester moieties of isoxazole **59** and triazole **62** then yielded isoxazole **60** and triazole **63**. In the final step, ozonolysis was performed to oxidize the terminal vinyl groups of isoxazole **60** and triazole **63** to their corresponding racemic aldehydes **61** and **64** (Scheme 5).

### 2.3. Biological Evaluation

The synthesized cyclic boronate, cyanomethyl and formylmethyl derivatives of **CG_209** and **CG_220** were tested for their inhibitory effects against sirtuin 5 using a fluorescence-based assay utilizing a 7-amino-4-methyl coumarin-based succinylated lysine as its substrate. The IC_50_ values of these compounds were calculated as a mean of three measurements (Table 2). The determined IC_50_ values of the literature-known lead structures **CG_209** and **CG_220** in this assay were in accordance with their published values of 12 µM and 7 µM, respectively [21,22]. In general, all the functionalized inhibitors showed micromolar inhibitory effects against sirtuin 5 but showed a loss in the potency compared to their lead structures. Among the three functional group modifications that were employed, cyanomethyl analogues showed the highest potency, followed by formyl and then boronate derivatives. Significant loss in the potency of the boronates **51**–**54** could be observed compared to their lead structures **CG_209** and **CG_220**.

We previously investigated the tolerance of several modifications in the salicylic acid moiety and found that a free salicylate is beneficial, but not mandatory, for affinity for Sirt5 [22]. So, although the acetonide protecting group may contribute to the loss in potency observed for **51**–**54** here, we believe that this observation is likely due to the introduction of the cyclic boronate moieties. The additional rigidization associated with the cyclic boronate may restrict the flexibility of the inhibitor that is required for optimal fitting in the active site of sirtuin 5. In contrast, functionalization with cyanomethyl groups was better tolerated with cyanomethyl derivative **50** representing the most potent functionalized inhibitor in this library screening. With an IC_50_ of 27 µM, the potency of compound **50** thus lies in a similar range of other established sirtuin 5 inhibitors published in the literature. Additionally, a general trend of higher potency for the triazole derivatives (**49**, **50**, **53**, **54** and **64**) could be interpreted when they were compared with the isoxazole derivatives (**47**, **48**, **51**, **52** and **61**) of the corresponding functionalization. For example, the *S*-enantiomer of triazole boronate **54** displayed an IC_50_ of 61 µM, a doubling in the potency compared to its corresponding isoxazole derivative **52**. Furthermore, a stereo-selective preference of these functional group modifications could be observed. In all cases, the *S*-enantiomers showed higher potency than the corresponding *R*-enantiomers. The *S*-enantiomer of the cyanomethyl isoxazole **48**, for instance, displayed an IC_50_ of 65 µM, whereas the corresponding *R*-enantiomer **47** had an IC_50_ of 97 µM. Based on these results, we concluded that modifications incorporating moieties such as boronic acids, cyanomethyl and formylmethyl groups were tolerated to a certain extent and retained micromolar inhibitory in vitro activities against sirtuin 5. Although these functionalized derivatives did not yield the anticipated enhancement in potency and thus reversible covalent inhibition of the functionalized inhibitors is not evident as their inhibitory activities would otherwise be significantly improved via reversible covalent binding with the co-factor NAD^+^, our findings offer valuable insights into the SAR of balsalazide analogues as sirtuin 5 inhibitors. Importantly, these results highlight the triazole-based scaffold with potentially further modifications in the *S*-configuration as a promising platform for further structural elaborations. Future optimization efforts focusing on diverse functional group modifications that are not limited to reversible covalent inhibition may lead to the development of more potent sirtuin 5 inhibitors. Although the exact binding mechanism of these functionalized sirtuin 5 inhibitors could not yet be shown in this study due to significant challenges in obtaining stable co-crystals with these inhibitors, ongoing efforts are actively being made to elucidate its structural biology. Notably, the majority of the obtained sirtuin 5 co-crystals are in complex with macromolecules or peptide-based larger molecules [41,42], highlighting the challenges in obtaining sirtuin 5 co-crystals with low-molecular inhibitors.

## 3. Materials and Methods

### 3.1. Chemical Synthesis

All solvents and reagents were purchased from commercial sources and used without further purification. Standard vacuum line techniques were applied. Reactions were monitored via thin-layer silica gel chromatography (TLC) using polyester sheets POLYGRAM SIL G/UV254 coated with 0.2 mm silica gel (Macherey-Nagel, Düren, Germany). Plates were visualized using UV light (254 nm or 365 nm) or staining with KMnO_4_, curcumin, CAM (ceric ammonium molybdate) or DNPH (dinitrophenylhydrazine). Products were purified by flash column chromatography (normal-phase silica gel chromatography or boric acid-impregnated normal-phase silica gel chromatography) using SiO_2_ 60 (0.040–0.063 mm, 230–400 mesh ASTM) from Merck (Darmstadt, Germany). NMR spectra were recorded with Avance III HD 400 MHz Bruker BioSpin and Avance III HD 500 MHz Bruker BioSpin (^1^H-NMR: 400 MHz and 500 MHz, ^13^C-NMR: 101 MHz and 126 MHz) (Bruker, Billerica, MA, USA) using the deuterated solvent stated. Chemical shifts (*δ*) are quoted in parts per million (ppm) and referenced to the residual solvent peak. Multiplicities are denoted as follows: s—singlet, d—doublet, t—triplet, q—quartet and quin—quintet. Coupling constants *J* are given in Hz and rounded to the nearest 0.1 Hz. Infrared spectra were recorded from 4000 to 650 cm^−1^ on a Perkin Elmer Spectrum BX-59343 FT-IR instrument (Perkin Elmer, Shelton, CT, USA). A Smiths Detection DuraSamp IR II Diamond ATR sensor (Smiths Detection, Danbury, CT, USA) was used for detection. The absorption bands are reported in wavenumbers [cm^−1^]. High-resolution mass spectra (HR-MS) were recorded using a Jeol Mstation 700 or JMS GCmate II Jeol instrument (Jeol, Tokyo, Japan) for electron impact ionization (EI). Thermo Finnigan LTQ (Thermo Finnigan, Somerset, NJ, USA) was used for electrospray ionization (ESI). Melting points were measured with a Büchi Schmelzpunktapparatur B-540 (Büchi, Flawil, Switzerland). HPLC analytical measurements at 210 nm and 254 nm for purities determination was performed with a Zorbax Eclipse Plus C18 column (Waters, Milford, MA, USA) with a diameter of 4.6 mm and a length of 150 mm and a particle size of 3.5 µm.

**General Procedure A—*N*-Boc deprotection with HCl** [33]**.** The appropriate *N*-Boc-protected amine (1.0 equivalent) was dissolved in 4.0 M HCl dioxane solution (3.2 equivalents) under N_2_ atmosphere and stirred at room temperature for 1 h. The solvent was removed in vacuo and then co-evaporated with diethyl ether (4×). The obtained amine hydrochlorides could be used for the subsequent steps without further purification.

**General Procedure B—Amide coupling with CDI.** To a stirred solution of the appropriate carboxylic acid (1.0 equivalent) in DMF with a concentration of 0.20 M, CDI (1.1 equivalents) was added. The reaction mixture was stirred at room temperature for 15 min. Afterwards, the appropriate amine (1.0 equivalent) was added, and unless stated otherwise, the solution was stirred at room temperature for 3 d. The solution was diluted with EtOAc, and the organic phase was washed with brine (3×). The organic phase was dried using a phase separation paper, and the solvent was removed in vacuo. If necessary, the crude product was purified by FCC using the indicated eluent.

**General Procedure C—PIFA-mediated isoxazole formation** [43]**.** To a stirred solution of the appropriate alkyne (1.0 equivalent) in MeOH/H_2_O (5:1 *v*/*v*) with a concentration of 0.10 M, the appropriate oxime (1.5 equivalents) was added. Then, 0.5 equivalents of PIFA were added every 2 h. After the addition of 1.5 equivalents PIFA, the reaction mixture was stirred at room temperature for another 16 h. The solvents were removed in vacuo, and the crude product was resuspended in EtOAc. Water was added, the resulting two phases were separated, and the aqueous phase was extracted with EtOAc (3×). The combined organic phases were dried using a phase separation paper and the solvent was removed in vacuo. The crude product was purified by FCC using the indicated eluent.

**General Procedure D—Copper-catalyzed azide-alkyne cycloaddition.** To a stirred solution of the appropriate azide (1.0 equivalent) in *t*-BuOH/DMSO (21:1 *v*/*v*) with a concentration of 0.13 M, we added a solution of the appropriate alkyne (1.2 equivalents) in *t*-BuOH with a concentration of 0.33 M. A 0.027 M aq. CuSO_4_ solution (0.2 equivalents) and a 0.13 M aq. sodium ascorbate solution (1.0 equivalent) were added, and the reaction mixture was stirred at room temperature for 18 h. The reaction mixture was diluted with EtOAc, and the organic phase was washed sequentially with 1 M HCl (1×), aq. sat. NaHCO_3_ (1×) and brine (1×). The organic phase was dried using a phase separation paper, and the solvent was removed in vacuo. The crude product was purified by the indicated method.

**General Procedure E—Alkaline deprotection of acetonides and esters.** To a stirred solution of the appropriate protected starting material (1.0 equivalent) in THF with a concentration of 0.15 M, an aq. KOH solution was added with the stated concentration and equivalents. The reaction mixture was stirred at the stated temperature for the stated time and then acidified to pH 1 with 2 M HCl. EtOAc was added to the suspension, the resulting two phases were separated, and the aqueous phase was extracted with EtOAc (3×). The combined organic phases were dried using a phase separation paper, and the solvent was removed in vacuo. The crude product was purified by the indicated method.

**Ethyl 3-(4-ethynylbenzamido)propanoate (6).** Prepared according to **General Procedure B** from ß-alanine ethyl ester hydrochloride (1.00 g, 6.51 mmol) and 4-ethynylbenzoic acid (1.00 g, 6.51 mmol). The reaction mixture was stirred at room temperature for 2.5 h. The amide **6** (1.20 g, 4.89 mmol, 75%) was obtained as a brownish-yellow solid. Analytical data are in alignment with literature [22].

**Benzyl (*R*)-3-((*tert*-butoxycarbonyl)amino)-4-(4,4,5,5-tetramethyl-1,3,2-dioxaborolan-2-yl)butanoate (20).** Pd_2_(dba)_3_ (31.9 mg, 0.0348 mmol), di-*tert*-butyl(methyl)phosphonium tetrafluoroborate (51.8 mg, 0.209 mmol), bis(pinacolato)diboron (2.12 g, 8.35 mmol), K_2_CO_3_ (1.92 g, 13.9 mmol) and alkyl bromide **18** (2.59 g, 6.96 mmol) were added to a 100 mL round-bottomed flask and evacuated and backfilled three times with N_2_. 19 mL of a degassed *t*-BuOH/H_2_O (12:1 *v*/*v*) solution were added. The reaction mixture was stirred at 65 °C for 6 h, then diluted with EtOAc (200 mL). The organic phase was washed with brine (2 × 200 mL), dried using a phase separation paper, concentrated in vacuo and purified by FCC with boric acid-impregnated silica gel (hexanes/EtOAc 89:11) to give boronic acid pinacol ester **20** (1.75 g, 4.17 mmol, 60%) as a yellow oil. ^1^H-NMR (400 MHz, (CDCl_3_): δ (ppm) = 7.37–7.29 (m, 5H, 2′-H, 3′-H, 4′-H, 5′-H and 6′-H), 5.12–5.09 (m, 2H, 1′-CH_2_), 4.24–4.16 (m, 1H, 3-H), 2.69–2.52 (m, 2H, 2-H), 1.42 (s, 9H, C(CH_3_)_3_), 1.24–1.20 (m, 12H, (C(CH_3_)_2_)_2_), 1.15 (d, *J* = 6.7 Hz, 2H, 4-H). ^13^C-NMR (101 MHz, (CDCl_3_): δ (ppm) = 171.59 (C-1), 155.13 (NHCOO), 136.05 (C-1′), 128.67–128.32 (C-2′, C-3′, C-4′, C-5′ and C-6′), 83.59 ((**C**(CH_3_)_2_)_2_), 79.22 (**C**(CH_3_)_3_), 66.41 (1′-CH_2_), 44.96 (C-3) 41.17 (C-2), 28.56 (C(**C**H_3_)_3_), 24.97–24.86 ((C(**C**H_3_)_2_)_2_), 17.78 (C-4). IR (ATR): *ṽ* (cm^−1^) = 3348, 2977, 1709, 1514, 1366, 1168, 1140, 1019, 965, 846, 697. HR-MS (ESI): *m*/*z* = [M + Na]^+^ calcd for C_22_H_34_BNO_6_Na^+^: 442.2377; found: 442.2372. Specific rotation: [α${]}_{D}^{20}$ +3 (c 0.1 in DMSO).

**Benzyl (*S*)-3-((*tert*-butoxycarbonyl)amino)-4-(4,4,5,5-tetramethyl-1,3,2-dioxaborolan-2-yl)butanoate (21).** Pd_2_(dba)_3_ (49.2 mg, 0.0537 mmol), di-*tert*-butyl(methyl)phosphonium tetrafluoroborate (80.0 mg, 0.322 mmol), bis(pinacolato)diboron (3.27 g, 12.9 mmol), K_2_CO_3_ (2.97 g, 21.5 mmol) and alkyl bromide **19** (4.00 g, 10.7 mmol) were added to a 100 mL round-bottomed flask and evacuated and backfilled three times with N_2_. 32 mL of a degassed *t*-BuOH/H_2_O (12:1 *v*/*v*) solution were added. The reaction mixture was stirred at 65 °C for 6 h, then diluted with EtOAc (500 mL). The organic phase was washed with brine (2 × 400 mL), dried using a phase separation paper, concentrated in vacuo and purified by FCC with boric acid-impregnated silica gel (hexanes/EtOAc 89:11) to give boronic acid pinacol ester **21** (2.30 g, 5.48 mmol, 51%) as a yellow oil. ^1^H-NMR (400 MHz, (CDCl_3_): δ (ppm) = 7.38–7.29 (m, 5H, 2′-H, 3′-H, 4′-H, 5′-H and 6′-H), 5.13–5.08 (m, 2H, 1′-CH_2_), 4.27–4.08 (m, 1H, 3-H), 2.75–2.52 (m, 2H, 2-H), 1.45–1.40 (m, 9H, C(CH_3_)_3_), 1.25–1.20 (m, 12H, (C(CH_3_)_2_)_2_)), 1.15 (d, *J* = 6.7 Hz, 2H, 4-H). ^13^C-NMR (101 MHz, (CDCl_3_): δ (ppm) = 171.65 (C-1), 155.13 (NHCOO), 136.04 (C-1′), 128.68–128.34 (C-2′, C-3′, C-4′, C-5′ and C-6′), 83.59 ((**C**(CH_3_)_2_)_2_), 79.16 (**C**(CH_3_)_3_), 66.42 (1′-CH_2_), 44.87 (C-3), 41.13 (C-2), 28.56 (C(**C**H_3_)_3_), 24.96–24.86 ((C(**C**H_3_)_2_)_2_)), 17.96 (C-4). IR (ATR): *ṽ* (cm^−1^) = 3371, 2977, 1711, 1498, 1366, 1164, 1140, 1019, 967, 846, 696. HR-MS (ESI): *m*/*z* = [M + Na]^+^ calcd for C_22_H_34_BNO_6_Na^+^: 442.2377; found: 442.2376. Specific rotation: [α${]}_{D}^{20}$ −5 (c 0.1 in DMSO).

**(*R*)-4-(Benzyloxy)-4-oxo-1-(4,4,5,5-tetramethyl-1,3,2-dioxaborolan-2-yl)butan-2-aminium chloride (24).** Prepared according to **General Procedure A** from *N*-Boc-protected amine **20** (3.20 g, 7.63 mmol). The amine hydrochloride **24** (2.45 g, 7.65 mmol, quant.) was obtained as a viscous yellow oil. ^1^H-NMR (500 MHz, (CD_3_)_2_SO): δ (ppm) = 8.04 (s, 3H, NH_3_^+^), 7.40–7.32 (m, 5H, 2′-H, 3′-H, 4′-H, 5′-H and 6′-H), 5.14 (d, *J* = 6.9 Hz, 2H, 1′-CH_2_), 3.63–3.53 (m, 1H, 2-H), 2.79–2.67 (m, 2H, 3-H), 1.27–1.22 (m, 1H, 1-H), 1.19 (d, *J* = 2.5 Hz, 12H, (C(CH_3_)_2_)_2_), 1.16–1.13 (m, 1H, 1-H). ^13^C-NMR (126 MHz, (CD_3_)_2_SO): δ (ppm) = 169.87 (C-4), 135.74 (C-1′), 128.45–128.05 (C-2′, C-3′, C-4′, C-5′ and C-6′), 83.51 ((**C**(CH_3_)_2_)_2_), 66.06 (1′-CH_2_), 44.96 (C-2), 38.12 (C-3), 24.46 ((C(**C**H_3_)_2_)_2_), 16.03 (C-1). IR (ATR): *ṽ* (cm^−1^) = 3263, 1978, 2883, 2828, 2109, 1727, 1382, 1322, 1204, 1163, 1140, 967, 844, 696. HR-MS (ESI): *m*/*z* = [M]^+^ calcd for C_17_H_27_BNO_4_^+^: 320.2028; found: 320.2027. Specific rotation: [α${]}_{D}^{20}$ −6 (c 0.1 in DMSO).

**(*S*)-4-(Benzyloxy)-4-oxo-1-(4,4,5,5-tetramethyl-1,3,2-dioxaborolan-2-yl)butan-2-aminium chloride (25).** Prepared according to **General Procedure A** from *N*-Boc-protected amine **21** (2.80 g, 6.68 mmol). The amine hydrochloride **25** (2.14 g, 6.68 mmol, quant.) was obtained as a viscous yellow oil. ^1^H-NMR (500 MHz, (CD_3_)_2_SO): δ (ppm) = 8.04 (s, 3H, NH_3_^+^), 7.40–7.33 (m, 5H, 2′-H, 3′-H, 4′-H, 5′-H and 6′-H), 5.14 (d, *J* = 6.9 Hz, 2H, 1′-CH_2_), 3.64–3.51 (m, 1H, 2-H), 2.78–2.67 (m, 2H, 3-H), 1.27–1.22 (m, 1H, 1-H), 1.19 (d, *J* = 2.5 Hz, 12H, C(CH_3_)_2_)_2_), 1.16–1.10 (m, 1H, 1-H). ^13^C-NMR (101 MHz, (CD_3_)_2_SO): δ (ppm) = 169.86 (C-4), 135.74 (C-1′), 128.44–128.04 (C-2′, C-3′, C-4′, C-5′ and C-6′), 83.50 ((**C**(CH_3_)_2_)_2_), 66.05 (1′-CH_2_), 44.96 (C-2), 38.12 (C-3), 24.46 ((C(**C**H_3_)_2_)_2_), 16.02 (C-1). IR (ATR): *ṽ* (cm^−1^) = 2977, 2883, 2833, 2034, 1730, 1381, 1324, 1211, 1165, 1139, 966, 845, 696. HR-MS (ESI): *m*/*z* = [M]^+^ calcd for C_17_H_27_BNO_4_^+^: 320.2028; found: 320.2038. Specific rotation: [α${]}_{D}^{20}$ +7 (c 0.1 in DMSO).

**(*R*)-4-(Benzyloxy)-1-cyano-4-oxobutan-2-aminium chloride (26).** Prepared according to **General Procedure A** from *N*-Boc-protected amine **22** (2.50 g, 7.85 mmol). The amine hydrochloride **26** (1.72 g, 7.84 mmol, quant.) was obtained as a purple-gray solid. m.p.: 107 °C. ^1^H-NMR (400 MHz, (CD_3_)_2_SO): δ (ppm) = 8.59 (s, 3H, NH_3_^+^), 7.44–7.32 (m, 5H, 2′-H, 3′-H, 4′-H, 5′-H and 6′-H), 5.15 (s, 2H, 1′-CH_2_), 3.84 (p, *J* = 6.3 Hz, 1H, 2-H), 3.06 (dd, *J* = 6.0, 1.4 Hz, 2H, 1-H), 2.96–2.81 (m, 2H, 3-H). ^13^C-NMR (126 MHz, (CD_3_)_2_SO): δ (ppm) = 168.99 (C-4), 135.58 (C-1′), 128.47–128.16 (C-2′, C-3′, C-4′, C-5′ and C-6′), 116.74 (CN), 66.36 (1′-CH_2_), 43.86 (C-2), 36.09 (C-3), 20.80 (C-1). IR (ATR): *ṽ* (cm^−1^) = 3033, 2923, 2854, 2641, 2500, 2252, 1716, 1541, 1454, 1401, 1217, 1188, 1157, 1117, 972, 754, 696. HR-MS (ESI): *m*/*z* = [M]^+^ calcd for C_12_H_15_N_2_O_2_^+^: 219.1128; found: 219.1137. Specific rotation: [α${]}_{D}^{20}$ −8 (c 0.1 in DMSO).

**(*S*)-4-(Benzyloxy)-1-cyano-4-oxobutan-2-aminium chloride (27).** Prepared according to **General Procedure A** from *N*-Boc-protected amine **23** (1.30 g, 4.08 mmol). The amine hydrochloride **27** (895 g, 4.08 mmol, quant.) was obtained as a beige solid. m.p.: 109 °C. ^1^H-NMR (400 MHz, (CD_3_)_2_SO): δ (ppm) = 8.59 (s, 3H, NH_3_^+^), 7.43–7.33 (m, 5H, 2′-H, 3′-H, 4′-H, 5′-H and 6′-H), 5.15 (s, 2H, 1′-CH_2_), 3.84 (p, *J* = 6.3 Hz, 1H, 2-H), 3.06 (dd, *J* = 6.0, 1.4 Hz, 2H, 1-H), 2.95–2.80 (m, 2H, 3-H). ^13^C-NMR (101 MHz, (CD_3_)_2_SO): δ (ppm) = 168.98 (C-4), 135.57 (C-1′), 128.46–128.15 (C-2′, C-3′, C-4′, C-5′ and C-6′), 116.74 (CN), 66.36 (1′-CH_2_), 43.86 (C-2), 36.09 (C-3), 20.80 (C-1). IR (ATR): *ṽ* (cm^−1^) = 3033, 2922, 2854, 2641, 2541, 2252, 1716, 1541, 1454, 1401, 1217, 1188, 1157, 1117, 972, 754, 696. HR-MS (ESI): *m*/*z* = [M]^+^ calcd for C_12_H_15_N_2_O_2_^+^: 219.1128; found: 219.1124. Specific rotation: [α${]}_{D}^{20}$ +8 (c 0.1 in DMSO).

**Benzyl (*R*)-3-(4-ethynylbenzamido)-4-(4,4,5,5-tetramethyl-1,3,2-dioxaborolan-2-yl)butanoate (28).** Prepared according to **General Procedure B** from amine hydrochloride **24** (2.45 g, 7.68 mmol) and 4-ethynylbenzoic acid (1.18 g, 7.68 mmol). The crude product was purified by FCC with boric acid-impregnated silica gel (hexanes/EtOAc 75:25) to give amide **28** (2.73 mg, 6.10 mmol, 80%) as a yellow oil. ^1^H-NMR (400 MHz, (CD_3_)_2_SO): δ (ppm) = 8.38 (d, *J* = 8.2 Hz, 1H, CONH), 7.79–7.75 (m, 2H, 2′-H and 6′-H), 7.57–7.53 (m, 2H, 3′-H and 5′-H), 7.34–7.25 (m, 5H, 2″-H, 3″-H, 4″-H, 5″-H and 6″-H), 5.06–5.04 (m, 2H, 1″-CH_2_), 4.50–4.43 (m, 1H, 3-H), 4.36 (s, 1H, C≡CH), 2.69–2.55 (m, 2H, 2-H), 1.14 (s, 12H, C(CH_3_)_2_)_2_), 1.10–1.05 (m, 2H, 4-H). ^13^C-NMR (101 MHz, (CD_3_)_2_SO): δ (ppm) = 170.65 (C-1), 164.56 (CONH), 136.12 (C-1″), 134.80 (C-1′), 131.49 (C-3′ and C-5′), 128.30–127.80 (C-2″, C-3″, C-4″, C-5″ and C-6″), 127.48 (C-2′ and C-6′), 124.19 (C-4′), 82.88 ((**C**(CH_3_)_2_)_2_), 82.72 (**C**≡CH and C≡**C**H), 65.46 (1″-CH_2_), 43.97 (C-3), 41.04 (C-2), 24.48 ((C(**C**H_3_)_2_)_2_), 17.81 (C-4). IR (ATR): *ṽ* (cm^−1^) = 3263, 2978, 2109, 1729, 1641, 1537, 1372, 1323, 1140, 967, 848, 696. HR-MS (ESI): *m*/*z* = [M + H]^+^ calcd for C_26_H_31_BNO_5_^+^: 448.2290; found: 448.2288. Specific rotation: [α${]}_{D}^{20}$ +6 (c 0.1 in DMSO).

**Benzyl (*S*)-3-(4-ethynylbenzamido)-4-(4,4,5,5-tetramethyl-1,3,2-dioxaborolan-2-yl)butanoate (29).** Prepared according to **General Procedure B** from amine hydrochloride **25** (2.14 g, 6.68 mmol) and 4-ethynylbenzoic acid (1.03 g, 6.68 mmol). The crude product was purified by FCC with boric acid-impregnated silica gel (hexanes/EtOAc 75:25) to give amide **29** (1.92 g, 4.29 mmol, 64%) as a yellow oil. ^1^H-NMR (500 MHz, (CD_3_)_2_SO): δ (ppm) = 8.38 (d, *J* = 8.2 Hz, 1H, CONH), 7.78–7.75 (m, 2H, 2′-H and 6′-H), 7.57–7.53 (m, 2H, 3′-H and 5′-H), 7.33–7.26 (m, 5H, 2″-H, 3″-H, 4″-H, 5″-H and 6″-H), 5.07–5.04 (m, 2H, 1″-CH_2_), 4.50–4.43 (m, 1H, 3-H), 4.36 (s, 1H, C≡CH), 2.69–2.55 (m, 2H, 2-H), 1.14 (s, 12H, C(CH_3_)_2_)_2_), 1.10–1.03 (m, 2H, 4-H). ^13^C-NMR (101 MHz, (CD_3_)_2_SO): δ (ppm) = 170.65 (C-1), 164.56 (CONH), 136.12 (C-1′), 134.80 (C-1′), 131.49 (C-3′ and C-5′), 128.30–127.80 (C-2′′, C-3″, C-4″, C-5″ and C-6″), 127.48 (C-2′ and C-6′), 124.20 (C-4′), 82.88 ((**C**(CH_3_)_2_)_2_, **C**≡CH and C≡**C**H), 65.46 (1″-CH_2_), 43.98 (C-3), 41.04 (C-2), 24.48 ((C(**C**H_3_)_2_)_2_), 17.80 (C-4). IR (ATR): *ṽ* (cm^−1^) = 3284, 2978, 2107, 1731, 1639, 1535, 1372, 1323, 1139, 966, 846, 696. HR-MS (ESI): *m*/*z* = [M + H]^+^ calcd for C_26_H_31_BNO_5_^+^: 448.2290; found: 448.2295. Specific rotation: [α${]}_{D}^{20}$ −5 (c 0.1 in DMSO).

**Benzyl (*R*)-4-cyano-3-(4-ethynylbenzamido)butanoate (30).** Prepared according to **General Procedure B** from amine hydrochloride **26** (1.72 g, 7.84 mmol) and 4-ethynylbenzoic acid (1.21 g, 7.84 mmol). The crude product was purified by FCC (hexanes/EtOAc 80:20) to give amide **30** (1.58 g, 4.56 mmol, 58%) as a white solid. ^1^H-NMR (500 MHz, CDCl_3_): δ (ppm) = 7.66 (m, 2H, 2′-H and 6′-H), 7.53 (m, 2H, 3′-H and 5′-H), 7.36 (m, 5H, 2″-H, 3″-H, 4″-H, 5″-H and 6″-H), 7.00 (CONH), 5.18 (s, 2H, 1″-CH_2_), 4.71 (m, 1H, 3-H), 3.22 (s, 1H, C≡CH), 2.90 (m, 4H, 2-H and 4-H). ^13^C-NMR (126 MHz, CDCl_3_): δ (ppm) = 170.69 (C-1), 166.31 (CONH), 135.06 (C-1″), 133.32 (C-1′), 132.54 (C-3′ and C-5′), 128.91–128.65 (C-2″, C-3″, C-4″, C-5″ and C-6″), 127.14 (C-2′ and C-6′), 126.16 (C-4′), 116.88 (CN), 82.73 (**C**≡CH), 80.05 (C≡**C**H), 67.51 (1″-CH_2_), 43.67 (C-3), 36.92 (C-2), 22.68 (C-4). IR (ATR): *ṽ* (cm^−1^) = 3366, 3337, 3295, 3263, 2938, 2248, 1731, 1717, 1650, 1607, 1585, 1525, 1426, 1385, 1290, 1272, 1165, 1084, 1001, 967, 900, 853, 766, 751, 699. HR-MS (ESI): *m*/*z* = [M + Na]^+^ calcd for C_21_H_18_O_3_N_2_Na^+^: 369.1215; found: 369.1208. Specific rotation: [α${]}_{D}^{20}$ −9 (c 0.1 in CHCl_3_).

**Benzyl (*S*)-4-cyano-3-(4-ethynylbenzamido)butanoate (31).** Prepared according to **General Procedure B** from amine hydrochloride **27** (895 mg, 4.08 mmol) and 4-ethynylbenzoic acid (628 mg, 4.08 mmol). The crude product was purified by FCC (hexanes/EtOAc 70:30) to give amide **31** (998 mg, 2.88 mmol, 71%) as a beige solid. m.p.: 103 °C. ^1^H-NMR (500 MHz, CDCl_3_): δ (ppm) = 7.66 (m, 2H, 2′-H and 6′-H), 7.53 (m, 2H, 3′-H and 5′-H), 7.36 (m, 5H, 2′′-H, 3″-H, 4″-H, 5″-H and 6″-H), 7.00 (CONH), 5.18 (s, 2H, 1″-CH_2_), 4.71 (m, 1H, 3-H), 3.22 (s, 1H, C≡CH), 2.90 (m, 4H, 2-H and 4-H). ^13^C-NMR (126 MHz, CDCl_3_): δ (ppm) = 170.69 (C-1), 166.33 (CONH), 135.07 (C-1″), 133.32 (C-1′), 132.54 (C-3′ and C-5′), 128.91–128.64 (C-2″, C-3″, C-4″, C-5″ and C-6″), 127.15 (C-2′ and C-6′), 126.16 (C-4′), 116.89 (CN), 82.73 (**C**≡CH), 80.05 (C≡**C**H), 67.51 (1″-CH_2_), 43.68 (C-3), 36.93 (C-2), 22.68 (C-4). IR (ATR): *ṽ* (cm^−1^) = 3366, 3294, 3263, 2962, 2938, 2248, 1729, 1717, 1649, 1607, 1525, 1495, 1454, 1384, 1322, 1273, 1166, 1082, 1012, 967, 900, 853, 766, 750, 730, 699. HR-MS (ESI): *m*/*z* = [M + Na]^+^ calcd for C_21_H_18_O_3_N_2_Na^+^: 369.1215; found: 369.1210. Specific rotation: [α${]}_{D}^{20}$ +10 (c 0.1 in CHCl_3_).

**Methyl (*R*)-3-(4-(3-(2,2-dimethyl-4-oxo-4*H*-benzo[*d*][1,3]dioxin-6-yl)isoxazol-5-yl)benzamido)-4-(4,4,5,5-tetramethyl-1,3,2-dioxaborolan-2-yl)butanoate (32).** Prepared according to **General Procedure C** from alkyne **28** (350 mg, 0.782 mmol) and aldoxime **3** (260 mg, 1.17 mmol). The crude product was purified by FCC with boric acid-impregnated silica gel (hexanes/EtOAc 60:40) to give isoxazole **32** (141 mg, 0.239 mmol, 31%) as a pale yellow oil. ^1^H-NMR (500 MHz, (CD_3_)_2_SO): δ (ppm) = 8.44 (d, *J* = 8.2 Hz, 1H, CONH), 8.39 (d, *J* = 2.2 Hz, 1H, 5′′′-H), 8.24 (dd, *J* = 8.6, 2.2 Hz, 1H, 7′′′-H), 8.04–8.00 (m, 2H, 3′-H and 5′-H), 7.99–7.95 (m, 2H, 2′-H and 6′-H), 7.87 (s, 1H, 4″-H), 7.34 (d, *J* = 8.6 Hz, 1H, 8′′′-H), 4.50–4.41 (m, 1H, 3-H), 3.57 (s, 3H, OCH_3_), 2.66–2.54 (m, 2H, 2-H), 1.75 (s, 6H, 2′′′-(CH_3_)_2_), 1.16–1.15 (m, 12H, (C(CH_3_)_2_)_2_), 1.13–1.08 (m, 2H, 4-H). ^13^C-NMR (126 MHz, (CD_3_)_2_SO): δ (ppm) = 171.22 (C-1), 169.26 (C-5″), 164.50 (CONH), 161.38 (C-3″), 159.75 (C-4′′′), 156.78 (C-8a′′′), 136.25 (C-1′), 134.79 (C-7′′′), 128.74 (C-4′), 128.18 (C-2′ and C-6′), 127.33 (C-5′′′), 125.41 (C-3′ and C-5′), 123.34 (C-6′′′), 118.54 (C-8′′′), 113.56 (C-4a′′′), 107.01 (C-2′′′), 99.67 (C-4″), 82.90 (**C**(CH_3_)_2_)_2_), 51.32 (OCH_3_), 43.96 (C-3), 40.95 (C-2), 25.32 (2′′′-(CH_3_)_2_), 24.95–24.48 (C(**C**H_3_)_2_)_2_), 17.76 (C-4). IR (ATR): *ṽ* (cm^−1^) = 3324, 2979, 1732, 1626, 1498, 1378, 1286, 1200, 1141, 1046, 846, 826, 673. HR-MS (ESI): *m*/*z* = [M + H]^+^ calcd for C_31_H_36_BN_2_O_9_^+^: 591.2514; found: 591.2500. Specific rotation: [α${]}_{D}^{20}$ +3 (c 0.1 in DMSO).

**Methyl (*S*)-3-(4-(3-(2,2-dimethyl-4-oxo-4*H*-benzo[*d*][1,3]dioxin-6-yl)isoxazol-5-yl)benzamido)-4-(4,4,5,5-tetramethyl-1,3,2-dioxaborolan-2-yl)butanoate (33).** Prepared according to **General Procedure C** from alkyne **29** (650 mg, 1.45 mmol) and aldoxime **3** (482 mg, 2.18 mmol). The crude product was purified by FCC with boric acid-impregnated silica gel (hexanes/EtOAc 60:40) to give isoxazole **33** (187 mg, 0.316 mmol, 22%) as a pale yellow oil. ^1^H-NMR (400 MHz, (CD_3_)_2_SO): δ (ppm) = 8.44 (d, *J* = 8.2 Hz, 1H, CONH), 8.39 (d, *J* = 2.2 Hz, 1H, 5′′′-H), 8.24 (dd, *J* = 8.6, 2.2 Hz, 1H, 7′′′-H), 8.04–8.00 (m, 2H, 3′-H and 5′-H), 7.98–7.95 (m, 2H, 2′-H and 6′-H), 7.87 (s, 1H, 4″-H), 7.34 (d, *J* = 8.6 Hz, 1H, 8′′′-H), 4.50–4.41 (m, 1H, 3-H), 3.57 (s, 3H, OCH_3_), 2.65–2.54 (m, 2H, 2-H), 1.75 (s, 6H, 2′′′-(CH_3_)_2_), 1.16–1.15 (m, 12H, (C(CH_3_)_2_)_2_), 1.13–1.09 (m, 2H, 4-H). ^13^C-NMR (126 MHz, (CD_3_)_2_SO): δ (ppm) = 171.22 (C-1), 169.26 (C-5″), 164.51 (CONH), 161.38 (C-3″), 159.75 (C-4′′′), 156.78 (C-8a′′′), 136.26 (C-1′), 134.79 (C-7′′′), 128.74 (C-4′), 128.18 (C-2′ and C-6′), 127.34 (C-5′′′), 125.41 (C-3′ and C-5′), 123.33 (C-6′′′), 118.54 (C-8′′′), 113.56 (C-4a′′′), 107.01 (C-2′′′), 99.68 (C-4″), 82.90 (**C**(CH_3_)_2_)_2_), 51.33 (OCH_3_), 43.96 (C-3), 40.95 (C-2), 25.32 (2′′′-(CH_3_)_2_), 24.95–24.48 (C(**C**H_3_)_2_)_2_), 17.73 (C-4). IR (ATR): *ṽ* (cm^−1^) = 3388, 2980, 1735, 1624, 1498, 1379, 1288, 1200, 1139, 1052, 847, 828, 673. HR-MS (ESI): *m*/*z* = [M + H]^+^ calcd for C_31_H_36_BN_2_O_9_^+^: 591.2514; found: 591.2510. Specific rotation: [α${]}_{D}^{20}$ −3 (c 0.1 in DMSO).

**Benzyl (*R*)-4-cyano-3-(4-(3-(2,2-dimethyl-4-oxo-4*H*-benzo[*d*][1,3]dioxin-6-yl)isoxazol-5-yl)benzamido)butanoate (34).** Prepared according to **General Procedure C** from alkyne **30** (50.0 mg, 0.144 mmol) and aldoxime **3** (47.9 mg, 0.217 mmol). The crude product was purified by FCC (hexanes/EtOAc 60:40) to give isoxazole **34** (11.5 mg, 0.0203 mmol, 14%) as a white solid. m.p.: 161 °C. ^1^H-NMR (400 MHz, (CD_3_)_2_SO): δ (ppm) = 8.88 (d, *J* = 7.9 Hz, 1H, CONH), 8.40 (d, *J* = 2.3 Hz, 1H, 5″-H), 8.25 (dd, *J* = 8.6, 2.3 Hz, 1H, 7′′′-H), 8.02 (m, 4H, 2′-H, 3′-H, 5′-H and 6′-H), 7.90 (s, 1H, 4″-H), 7.35 (d, *J* = 3.7 Hz, 1H, 8′′′-H), 7.32 (m, 5H, 2′′′′-H, 3′′′′-H, 4′′′′-H, 5′′′′-H and 6′′′′-H), 5.11 (s, 2H,1′′′′-CH_2_), 4.63 (d, *J* = 6.9 Hz, 1H, 3-H), 2.93 (d, *J* = 11.9 Hz, 2H, 4-H), 2.83 (d, *J* = 9.5 Hz, 2H, 2-H), 1.75 (s, 6H, C(CH_3_)_2_). ^13^C-NMR (126 MHz, (CD_3_)_2_SO): δ (ppm) = 169.84 (C-1), 169.15 (C-5″), 165.29 (CONH), 161.43 (C-3″), 159.76 (C-4′′′), 156.79 (C-8a′′′), 135.92 (C-1′′′′), 135.30 (C-1′), 134.81 (C-7′′′), 129.17 (C-4′), 128.35 (C-2′ and C-6′), 128.00 -127.88, (C-2′′′′, C-3′′′′, C-4′′′′, C-5′′′′ and C-6′′′′), 127.36 (C-5′′′), 125.54 (C-3′ and C-5′), 123.31 (C-4a′′′), 118.55 (C-8′′′), 118.28 (CN), 107.02 (C-2′′′), 99.89 (C-4″), 65.82 (1′′′′-CH_2_), 43.67 (C-3), 37.71 (C-2), 25.32 (C(**C**H_3_)_2_), 22.43 (C-4). IR (ATR): *ṽ* (cm^−1^) = 3354, 1735, 1642, 1620, 1598, 1534, 1500, 1417, 1389, 1312, 1291, 1194, 1144, 1059, 981, 925, 855, 816, 774, 745, 697, 672. HR-MS (ESI): *m*/*z* = [M − H]^−^ calcd for C_32_H_26_O_7_N_3_^−^: 564.1771; found: 564.1770. Specific rotation: [α${]}_{D}^{20}$ −25 (c 0.1 in CHCl_3_).

**Benzyl (*S*)-4-cyano-3-(4-(3-(2,2-dimethyl-4-oxo-4*H*-benzo[*d*][1,3]dioxin-6-yl)isoxazol-5-yl)benzamido)butanoate (35).** Prepared according to **General Procedure C** from alkyne **31** (620 mg, 1.79 mmol) and aldoxime **3** (594 mg, 2.68 mmol). The crude product was purified by FCC (hexanes/EtOAc 60:40) to give isoxazole **35** (239 mg, 0.423 mmol, 24%) as a white solid. m.p.: 162 °C. ^1^H-NMR (400 MHz, (CD_3_)_2_SO): δ (ppm) = 8.88 (d, *J* = 7.9 Hz, 1H, CONH), 8.40 (d, *J* = 2.3 Hz, 1H, 5″-H), 8.25 (dd, *J* = 8.6, 2.3 Hz, 1H, 7′′′-H), 8.02 (m, 4H, 2′-H, 3′-H, 5′-H and 6′-H), 7.90 (s, 1H, 4″-H), 7.35 (d, *J* = 3.7 Hz, 1H, 8′′′-H), 7.32 (m, 5H, 2′′′′-H, 3′′′′-H, 4′′′′-H, 5′′′′-H and 6′′′′-H), 5.11 (s, 2H, 1′′′′-CH_2_), 4.63 (d, *J* = 6.9 Hz, 1H, 3-H), 2.93 (d, *J* = 11.9 Hz, 2H, 4-H), 2.83 (d, *J* = 9.5 Hz, 2H, 2-H), 1.75 (s, 6H, C(CH_3_)_2_). ^13^C-NMR (126 MHz, (CD_3_)_2_SO): δ (ppm) = 169.84 (C-1), 169.15 (C-5″), 165.29 (CONH), 161.43 (C-3″), 159.75 (C-4′′′), 156.79 (C-8a′′′), 135.92 (C-1′′′′), 135.30 (C-1′), 134.81 (C-7′′′), 129.17 (C-4′), 128.35 (C-2′ and C-6′), 128.00 -127.88 (C-2′′′′, C-3′′′′, C-4′′′′, C-5′′′′ and C-6′′′′), 127.36 (C-5′′′), 125.54 (C-3′ and C-5′), 123.31 (C-4a′′′), 118.54 (C-8′′′), 118.28 (CN), 107.01 (C-2′′′), 99.88 (C-4″), 65.82 (C-7′′′′), 43.67 (C-3), 37.71 (C-2), 25.32 (C(**C**H_3_)_2_), 22.43 (C-4). IR (ATR): *ṽ* (cm^−1^) = 3354, 1732, 1642, 1620, 1598, 1533, 1499, 1441, 1389, 1312, 1290, 1193, 1144, 1059, 962, 924, 855, 816, 774, 745, 697, 672. HR-MS (ESI): *m*/*z* = [M + Na]^+^ calcd for C_32_H_27_O_7_N_3_Na^+^: 588.1747; found: 588.1737. Specific rotation: [α${]}_{D}^{20}$ +13 (c 0.1 in CHCl_3_).

**(*R*)-(2-(4-(3-(2,2-Dimethyl-4-oxo-4*H*-benzo[*d*][1,3]dioxin-6-yl)isoxazol-5-yl)benzamido)-4-methoxy-4-oxobutyl)boronic acid (36).** To a stirred solution of boronic acid pinacol ester **32** (50 mg, 0.0847 mmol) in 3.5 mL THF/water (4:1 *v*/*v*), sodium periodate (90.6 mg, 0.423 mmol) was added, and the reaction mixture was stirred vigorously for 45 min at room temperature. 1M HCl (0.102 mmol, 0.102 mL) was then added to the resulting suspension, and the mixture stirred for another 16 h. The suspension was diluted with EtOAc (50 mL) and water (50 mL), the phases were separated, and the aqueous phase was extracted with EtOAc (2 × 50 mL). The combined organic phases were dried with Na_2_SO_4,_ concentrated in vacuo and purified by FCC with boric acid-impregnated silica gel (DCM/MeOH 97:3 -> 95:5) to give boronic acid **36** (31.2 mg, 0.0614 mmol, 73%) as a white solid. m.p.: 152 °C. ^1^H-NMR (500 MHz, (CD_3_)_2_SO): δ (ppm) = 8.39 (d, *J* = 2.2 Hz, 1H, 5′′′-H), 8.37 (d, *J* = 8.1 Hz, 1H, CONH), 8.25 (dd, *J* = 8.6, 2.2 Hz, 1H, 7′′′-H), 8.03–8.00 (m, 2H, 3′-H and 5′-H), 7.98–7.96 (m, 2H, 2′-H and 6′-H), 7.87 (s, 1H, 4″-H), 7.66 (s, 2H, B(OH)_2_), 7.34 (d, *J* = 8.6 Hz, 1H, 8′′′-H), 4.50–4.43 (m, 1H, 2-H), 3.56 (s, 3H, OCH_3_), 2.62–2.55 (m, 2H, 3-H), 1.75 (s, 6H, 2′′′-(CH_3_)_2_), 1.07–1.01 (m, 2H, 1-H). ^13^C-NMR (126 MHz, (CD_3_)_2_SO): 171.47 (C-4), 169.28 (C-5″), 164.65 (CONH), 161.40 (C-3″), 159.76 (C-4′′′), 156.78 (C-8a′′′), 136.32 (C-1′), 134.81 (C-7′′′), 128.73 (C-4′), 128.20 (C-2′ and C-6′), 127.34 (C-5′′′), 125.43 (C-3′ and C-5′), 123.34 (C-6′′′), 118.54 (C-8′′′), 113.56 (C-4a′′′), 107.01 (C-2′′′), 99.68 (C-4″), 51.21 (OCH_3_), 44.57 (C-2), 40.81 (C-3), 25.32 (2′′′-(CH_3_)_2_), 22.16 (C-1). IR (ATR): *ṽ* (cm^−1^) = 3323, 2952, 1731, 1623, 1498, 1286, 1200, 927, 764. HR-MS (ESI): *m*/*z* = [M − H]^−^ calcd for C_25_H_24_BN_2_O_9_^−^: 507.1575; found: 507.1579. Specific rotation: [α${]}_{D}^{20}$ −11 (c 0.1 in DMSO).

**(*S*)-(2-(4-(3-(2,2-Dimethyl-4-oxo-4*H*-benzo[*d*][1,3]dioxin-6-yl)isoxazol-5-yl)benzamido)-4-methoxy-4-oxobutyl)boronic acid (37).** To a stirred solution of boronic acid pinacol ester **33** (170 mg, 0.288 mmol) in 12 mL THF/water (4:1 *v*/*v*), sodium periodate (308 mg, 1.44 mmol) was added, and the reaction mixture was stirred vigorously for 45 min at room temperature. 1M HCl (0.346 mmol, 0.346 mL) was then added to the resulting suspension, and the mixture stirred for another 16 h. The suspension was diluted with EtOAc (100 mL) and water (100 mL), the phases were separated, and the aqueous phase was extracted with EtOAc (2 × 100 mL). The combined organic phases were dried with Na_2_SO_4_, concentrated in vacuo and purified by FCC with boric acid-impregnated silica gel (DCM/MeOH 97:3 -> 95:5) to give boronic acid **37** (93.1 mg, 0.183 mmol, 64%) as a white solid. m.p.: 157 °C. ^1^H-NMR (500 MHz, (CD_3_)_2_SO): δ (ppm) = 8.39 (d, *J* = 2.2 Hz, 1H, 5′′′-H), 8.37 (d, *J* = 8.2 Hz, 1H), 8.25 (dd, *J* = 8.6, 2.2 Hz, 1H, 7′′′-H), 8.03–8.00 (m, 2H, 3′-H and 5′-H), 7.99–7.96 (m, 2H, 2′-H and 6′-H), 7.87 (s, 1H, 4″-H), 7.66 (s, 2H, B(OH)_2_), 7.34 (d, *J* = 8.6 Hz, 1H, 8′′′-H), 4.51–4.41 (m, 1H, 2-H), 3.56 (s, 3H, OCH_3_), 2.64–2.55 (m, 2H, 3-H), 1.75 (s, 6H, 2′′′-(CH_3_)_2_), 1.08–0.99 (m, 2H, 1-H). ^13^C-NMR (126 MHz, (CD_3_)_2_SO): δ (ppm) = 171.47 (C-4), 169.28 (C-5″), 164.65 (CONH), 161.40 (C-3″), 159.76 (C-4′′′), 156.78 (C-8a′′′), 136.32 (C-1′), 134.81 (C-7′′′), 128.73 (C-4′), 128.20 (C-2′ and C-6′), 127.34 (C-5′′′), 125.43 (C-3′ and C-5′), 123.34 (C-6′′′), 118.54 (C-8′′′), 113.56 (C-4a′′′), 107.01 (C-2′′′), 99.68 (C-4″), 51.22 (OCH_3_), 44.56 (C-2), 40.82 (C-3), 25.32 (2′′′-(CH_3_)_2_), 22.09 (C-1). IR (ATR): *ṽ* (cm^−1^) = 3364, 2997, 1730, 1622, 1498, 1287, 1200, 927, 764. HR-MS (ESI): *m*/*z* = [M − H]^−^ calcd for C_25_H_24_BN_2_O_9_^−^: 507.1575; found: 507.1568. Specific rotation: [α${]}_{D}^{20}$ +10 (c 0.1 in DMSO).

**Benzyl (*R*)-3-(4-(1-(2,2-dimethyl-4-oxo-4*H*-benzo[*d*][1,3]dioxin-6-yl)-1*H*-1,2,3-triazol-4-yl)benzamido)-4-(4,4,5,5-tetramethyl-1,3,2-dioxaborolan-2-yl)butanoate (38).** Prepared according to **General Procedure D** from azide **11** (300 mg, 1.37 mmol) and alkyne **28** (735 mg, 1.64 mmol). The crude product was purified by FCC with boric acid-impregnated silica gel (hexanes/EtOAc 50:50) to give triazole **38** (153 mg, 0.230 mmol, 17%) as a pale yellow solid. m.p.: 140 °C. ^1^H-NMR (500 MHz, (CD_3_)_2_SO): δ (ppm) = 9.54 (s, 1H, 5″-H), 8.42 (d, *J* = 2.7 Hz, 1H, 5′′′-H), 8.37 (d, *J* = 8.2 Hz, 1H, CONH), 8.32 (dd, *J* = 8.9, 2.7 Hz, 1H, 7′′′-H), 8.05–8.00 (m, 2H, 3′-H and 5′-H), 7.95–7.89 (m, 2H, 2′-H and 6′-H), 7.44 (d, *J* = 8.9 Hz, 1H, 8′′′-H), 7.37–7.27 (m, 5H, 2′′′′-H, 3′′′′-H, 4′′′′-H, 5′′′′-H and 6′′′′-H), 5.09–5.07 (m, 2H, 1′′′′-CH_2_), 4.55–4.48 (m, 1H, 3-H), 2.73–2.67 (m, 1H, 2-H), 2.65–2.58 (m, 1H, 2-H), 1.78 (s, 6H, 2′′′-(CH_3_)_2_), 1.17 (s, 12H, (C(CH_3_)_2_)_2_), 1.16–1.06 (m, 2H, 4-H). ^13^C-NMR (126 MHz, (CD_3_)_2_SO): δ (ppm) = 170.71 (C-1), 164.88 (CONH), 159.48 (C-4′′′), 155.21 (C-8a′′′), 146.69 (C-4″), 136.16 (C-1′′′′), 134.27 (C-1′), 132.54 (C-4′), 131.70 (C-6′′′), 128.66 (C-7′′′), 128.31 (C-2′′′′, C-3′′′′, C-4′′′′, C-5′′′′ and C-6′′′′), 128.03 (C-2′ and C-6′), 127.90–127.81 (C-2′′′′, C-3′′′′, C-4′′′′, C-5′′′′ and C-6′′′′), 124.90 (C-3′ and C-5′), 120.66 (C-5″), 120.18 (C-5′′′), 119.26 (C-8′′′), 113.77 (C-4a′′′), 107.23 (C-2′′′), 82.90 ((**C**(CH_3_)_2_)_2_), 65.47 (1′′′′-CH_2_), 43.97 (C-3), 41.10 (C-2), 25.27 (2′′′-(CH_3_)_2_), 24.66–24.51 ((C(**C**H_3_)_2_)_2_), 17.85 (C-4). IR (ATR): *ṽ* (cm^−1^) = 3316, 2977, 1736, 1628, 1509, 1378, 1295, 1139, 1047, 853, 697. HR-MS (ESI): *m*/*z* = [M + H]^+^ calcd for C_36_H_40_BN_4_O_8_^+^: 667.2939; found: 667.2961. Specific rotation: [α${]}_{D}^{20}$ +4 (c 0.1 in DMSO).

**Benzyl (*S*)-3-(4-(1-(2,2-dimethyl-4-oxo-4*H*-benzo[*d*][1,3]dioxin-6-yl)-1*H*-1,2,3-triazol-4-yl)benzamido)-4-(4,4,5,5-tetramethyl-1,3,2-dioxaborolan-2-yl)butanoate (39).** Prepared according to **General Procedure D** from azide **11** (300 mg, 1.37 mmol) and alkyne **29** (735 mg, 1.64 mmol). The crude product was purified by FCC with boric acid-impregnated silica gel (hexanes/EtOAc 50:50) to give triazole **39** (76.8 mg, 0.115 mmol, 8%) as a pale yellow solid. m.p.: 129 °C. ^1^H-NMR (400 MHz, (CD_3_)_2_SO): δ (ppm) = 9.53 (s, 1H, 5′′-H), 8.41 (d, *J* = 2.7 Hz, 1H, 5′′′-H), 8.36 (d, *J* = 8.2 Hz, 1H, CONH), 8.31 (dd, *J* = 8.9, 2.7 Hz, 1H, 7′′′-H), 8.05–7.98 (m, 2H, 3′-H and 5′-H), 7.95–7.87 (m, 2H, 2′-H and 6′-H), 7.43 (d, *J* = 8.9 Hz, 1H, 8′′′-H), 7.35–7.26 (m, 5H, 2′′′′-H, 3′′′′-H, 4′′′′-H, 5′′′′-H and 6′′′′-H), 5.08–5.06 (m, 2H, 1′′′′-CH_2_), 4.55–4.44 (m, 1H, 3-H), 2.72–2.65 (m, 1H, 2-H), 2.64–2.57 (m, 1H, 2-H), 1.77 (s, 6H, 2′′′-(CH_3_)_2_), 1.16 (s, 12H, (C(CH_3_)_2_)_2_), 1.16–1.04 (m, 1H, 4-H). ^13^C-NMR (101 MHz, (CD_3_)_2_SO): δ (ppm) = 170.71 (C-1), 164.87 (CONH), 159.48 (C-4′′′), 155.21 (C-8a′′′), 146.69 (C-4″), 136.15 (C-1′′′′), 134.27 (C-1′), 132.53 (C-4′), 131.70 (C-6′′′), 128.65 (C-7′′′), 128.31 (C-2′′′′, C-3′′′′, C-4′′′′, C-5′′′′ and C-6′′′′), 128.02 (C-2′ and C-6′), 127.89–127.80 (C-2′′′′, C-3′′′′, C-4′′′′, C-5′′′′ and C-6′′′′), 124.90 (C-3′ and C-5′), 120.66 (C-5″), 120.18 (C-5′′′), 119.25 (C-8′′′), 113.77 (C-4a′′′), 107.23 (C-2′′′), 82.89 ((**C**(CH_3_)_2_)_2_), 65.47 (1′′′′-CH_2_), 43.97 (C-3), 41.09 (C-2), 25.27 (2′′′-(CH_3_)_2_), 24.66–24.50 ((C(**C**H_3_)_2_)_2_), 17.87 (C-4). IR (ATR): *ṽ* (cm^−1^) = 3316, 2977, 1734, 1627, 1509, 1377, 1295, 1139, 1046, 847, 697. HR-MS (ESI): *m*/*z* = [M + H]^+^ calcd for C_36_H_40_BN_4_O_8_^+^: 667.2939; found: 667.2949. Specific rotation: [α${]}_{D}^{20}$ −3 (c 0.1 in DMSO).

**Benzyl (*R*)-4-cyano-3-(4-(1-(2,2-dimethyl-4-oxo-4*H*-benzo[*d*][1,3]dioxin-6-yl)-1*H*-1,2,3-triazol-4-yl)benzamido)butanoate (40).** Prepared according to **General Procedure D** from azide **11** (150 mg, 0.684 mmol) and alkyne **30** (284 mg, 0.821 mmol). The crude product was resuspended in EtOAc (20 mL), filtered and the solid residue collected to give triazole **40** (157 mg, 0.278 mmol, 41%) as a pale yellow solid. m.p.: 196 °C. ^1^H-NMR (400 MHz, (CD_3_)_2_SO): δ (ppm) = 9.55 (s, 1H, 5′′-H), 8.79 (d, *J* = 7.9 Hz, 1H, CONH), 8.41 (d, *J* = 2.7 Hz, 1H, 5′′′-H), 8.31 (dd, *J* = 8.9, 2.8 Hz, 1H, 7′′′-H), 8.08–8.04 (m, 2H, 3′-H and 5′-H), 7.98–7.94 (m, 2H, 2′-H and 6′-H), 7.44 (d, *J* = 8.9 Hz, 1H, 8′′′-H), 7.36–7.28 (m, 5H, 2′′′′-H, 3′′′′-H, 4′′′′-H, 5′′′′-H and 6′′′′-H), 5.11 (s, 2H, 1′′′′-CH_2_), 4.68–4.57 (m, 1H, 3-H), 2.99–2.75 (m, 4H, 2-H and 4-H), 1.77 (s, 6H, (CH_3_)_2_). ^13^C-NMR (101 MHz, (CD_3_)_2_SO): δ (ppm) = 169.88 (C-1), 165.63 (CONH), 159.48 (C-4′′′), 155.23 (C-8a′′′), 146.59 (C-4″), 135.94 (C-1′′′′), 133.35 (C-1′), 133.04 (C-4′), 131.69 (C-6′′′), 128.69 (C-7′′′), 128.35 (C-2′′′′, C-3′′′′, C-4′′′′, C-5′′′′ and C-6′′′′), 128.17 (C-2′ and C-6′), 127.99–127.86 (C-2′′′′, C-3′′′′, C-4′′′′, C-5′′′′ and C-6′′′′), 125.04 (C-3′ and C-5′), 120.81 (C-5″), 120.22 (C-5′′′), 119.26 (C-8′′′), 118.32 (CN), 113.77 (C-4a′′′), 107.23 (C-2′′′), 65.81 (1′′′′-CH_2_), 43.61 (C-3), 37.77 (C-2), 25.27 ((CH_3_)_2_), 22.45 (C-4). IR (ATR): *ṽ* (cm^−1^) = 3330, 1735, 1721, 1645, 1524, 1506, 1296, 1040. HR-MS (ESI): *m*/*z* = [M + H]^+^ calcd for C_31_H_28_N_5_O_6_^+^: 566.2040; found: 566.2034. Specific rotation: [α${]}_{D}^{20}$ −9 (c 0.1 in DMSO).

**Benzyl (*S*)-4-cyano-3-(4-(1-(2,2-dimethyl-4-oxo-4*H*-benzo[*d*][1,3]dioxin-6-yl)-1*H*-1,2,3-triazol-4-yl)benzamido)butanoate (41).** Prepared according to **General Procedure D** from azide **11** (200 mg, 0.912 mmol) and alkyne **31** (379 mg, 1.09 mmol). The crude product was resuspended in EtOAc (20 mL), filtered and the solid residue collected to give triazole **41** (275 mg, 0.487 mmol, 53%) as a yellow solid. m.p.: 198 °C. ^1^H-NMR (400 MHz, (CD_3_)_2_SO): δ (ppm) = 9.55 (s, 1H, 5′′-H), 8.80 (d, *J* = 7.9 Hz, 1H, CONH), 8.41 (d, *J* = 2.7 Hz, 1H, 5′′′-H), 8.31 (dd, *J* = 8.9, 2.7 Hz, 1H, 7′′′-H), 8.10–8.03 (m, 2H, 3′-H and 5′-H), 8.00–7.93 (m, 2H, 2′-H and 6′-H), 7.44 (d, *J* = 9.0 Hz, 1H, 8′′′-H), 7.37–7.28 (m, 5H, 2′′′′-H, 3′′′′-H, 4′′′′-H, 5′′′′-H and 6′′′′-H), 5.11 (s, 2H, 1′′′′-CH_2_), 4.67–4.57 (m, 1H, 3-H), 2.99–2.76 (m, 4H, 2-H and 4-H), 1.77 (s, 6H, (CH_3_)_2_). ^13^C-NMR (126 MHz, (CD_3_)_2_SO): δ (ppm) = 169.89 (C-1), 165.62 (CONH), 159.48 (C-4′′′), 155.23 (C-8a′′′), 146.59 (C-4″), 135.94 (C-1′′′′), 133.35 (C-1′), 133.04 (C-4′), 131.69 (C-6′′′), 128.69 (C-7′′′), 128.35 (C-2′′′′, C-3′′′′, C-4′′′′, C-5′′′′ and C-6′′′′), 128.17 (C-2′ and C-6′), 127.99–127.87 (C-2′′′′, C-3′′′′, C-4′′′′, C-5′′′′ and C-6′′′′), 125.05 (C-3′ and C-5′), 120.82 (C-5″), 120.22 (C-5′′′), 119.26 (C-8′′′), 118.33 (CN), 113.77 (C-4a′′′), 107.24 (C-2′′′), 65.81 (1′′′′-CH_2_), 43.61 (C-3), 37.77 (C-2), 25.27 ((CH_3_)_2_), 22.45 (C-4). IR (ATR): *ṽ* (cm^−1^) = 3325, 1734, 1720, 1636, 1523, 1506, 1295, 1039. HR-MS (ESI): *m*/*z* = [M + Na]^+^ calcd for C_31_H_27_N_5_O_6_Na^+^: 588.1859; found: 588.1864. Specific rotation: [α${]}_{D}^{20}$ +7 (c 0.1 in DMSO).

**(*R*)-(4-(Benzyloxy)-2-(4-(1-(2,2-dimethyl-4-oxo-4*H*-benzo[*d*][1,3]dioxin-6-yl)-1*H*-1,2,3-triazol-4-yl)benzamido)-4-oxobutyl)boronic acid (42). Method A**: Prepared according to **General Procedure D** from azide **11** (300 mg, 1.37 mmol) and alkyne **28** (735 mg, 1.64 mmol). The crude product was purified by FCC with boric acid-impregnated silica gel (DCM/MeOH 95:5) to give triazole **42** (193 mg, 0.331 mmol, 24%) as a yellow solid. **Method B**: To a stirred solution of boronic acid pinacol ester 38 (70 mg, 0.105 mmol) in 5 mL THF/water (4:1 *v*/*v*), sodium periodate (112 mg, 0.525 mmol) was added, and the reaction mixture was stirred vigorously for 45 min at room temperature. 1 M HCl (0.126 mmol, 0.126 mL) was added to the resulting suspension, and the mixture stirred for another 16 h. The suspension was then filtered and the filtrate concentrated in vacuo. The crude product was purified by FCC with boric acid-impregnated silica gel (DCM/MeOH 95:5) to give triazole **42** (41.9 mg, 0.0717 mmol, 68%) as a yellow solid. m.p.: 138 °C. ^1^H-NMR (400 MHz, (CD_3_)_2_SO): δ (ppm) = 9.53 (s, 1H, 5″-H), 8.41 (d, *J* = 2.7 Hz, 1H, 5′′′-H), 8.31 (dd, *J* = 8.8, 2.5 Hz, 2H, CONH and 7′′′-H), 8.05–8.00 (m, 2H, 3′-H and 5′-H), 7.94–7.88 (m, 2H, 2′-H and 6′-H), 7.67 (s, 2H, B(OH)_2_), 7.44 (d, *J* = 8.9 Hz, 1H, 8′′′-H), 7.35–7.26 (m, 5H, 2′′′′-H, 3′′′′-H, 4′′′′-H, 5′′′′-H and 6′′′′-H), 5.05 (s, 2H, 1′′′′-CH_2_), 4.47–4.55 (m, 1H, 2-H), 2.69–2.62 (m, 2H, 3-H), 1.77 (s, 6H, (CH_3_)_2_), 1.08–1.01 (m, 2H, 1-H). ^13^C-NMR (126 MHz, (CD_3_)_2_SO): δ (ppm) = 170.97 (C-4), 165.07 (CONH), 159.49 (C-4′′′), 155.22 (C-8a′′′), 146.69 (C-4″), 136.21 (C-1′′′′), 134.28 (C-1′), 132.55 (C-4′), 131.71 (C-6′′′), 128.69 (C-7′′′), 128.30–128.04 (C-2′′′′, C-3′′′′, C-4′′′′, C-5′′′′ and C-6′′′′), 127.85 (C-2′ and C-6′), 127.81 (C-2′′′′, C-3′′′′, C-4′′′′, C-5′′′′ and C-6′′′′), 124.92 (C-3′ and C-5′), 120.68 (C-5″), 120.20 (C-5′′′), 119.26 (C-8′′′), 113.77 (C-4a′′′), 107.24 (C-2′′′), 65.40 (1′′′′-CH_2_), 44.53 (C-2), 41.01 (C-3), 25.27 ((CH_3_)_2_), 22.49 (C-1). IR (ATR): *ṽ* (cm^−1^) = 3307, 1735, 1624, 1509, 1295, 1202, 1047, 932, 697. HR-MS (ESI): *m*/*z* = [M + Na]^+^ calcd for C_30_H_29_BN_4_O_8_Na^+^: 607.1976; found: 607.1970. Specific rotation: [α${]}_{D}^{20}$ −15 (c 0.1 in DMSO).

**(*S*)-(4-(Benzyloxy)-2-(4-(1-(2,2-dimethyl-4-oxo-4*H*-benzo[*d*][1,3]dioxin-6-yl)-1*H*-1,2,3-triazol-4-yl)benzamido)-4-oxobutyl)boronic acid (43).** Prepared according to **General Procedure D** from azide **11** (300 mg, 1.37 mmol) and alkyne **29** (735 mg, 1.64 mmol). The crude product was purified by FCC with boric acid-impregnated silica gel (DCM/MeOH 95:5) to give triazole **43** (263 mg, 0.450 mmol, 33%) as a yellow solid. m.p.: 141 °C. ^1^H-NMR (500 MHz, (CD_3_)_2_SO): δ (ppm) = 9.53 (s, 1H, 5″-H), 8.41 (d, *J* = 2.7 Hz, 1H, 5″′-H), 8.31 (dd, *J* = 9.0, 2.5 Hz, 2H, CONH and 7′′′-H), 8.05–7.99 (m, 2H, 3′-H and 5′-H), 7.93–7.90 (m, 2H, 2′-H and 6′-H), 7.67 (s, 2H, B(OH)_2_), 7.44 (d, *J* = 8.9 Hz, 1H, 8′′′-H), 7.35–7.26 (m, 5H, 2′′′′-H, 3′′′′-H, 4′′′′-H, 5′′′′-H and 6′′′′-H), 5.05 (s, 2H, 1′′′′-CH_2_), 4.47–4.55 (m, 1H, 2-H), 2.67–2.63 (m, 2H, 3-H), 1.77 (s, 6H, (CH_3_)_2_), 1.10–1.00 (m, 2H, 1-H). ^13^C-NMR (126 MHz, (CD_3_)_2_SO): δ (ppm) = 170.98 (C-4), 165.09 (CONH), 159.50 (C-4′′′), 155.23 (C-8a′′′), 146.70 (C-4″), 136.21 (C-1′′′′), 134.28 (C-1′), 132.57 (C-4′), 131.72 (C-6′′′), 128.70 (C-7′′′), 128.31–128.05 (C-2′′′′, C-3′′′′, C-4′′′′, C-5′′′′ and C-6′′′′), 127.87 (C-2′ and C-6′), 127.82 (C-2′′′′, C-3′′′′, C-4′′′′, C-5′′′′ and C-6′′′′), 124.93 (C-3′ and C-5′), 120.69 (C-5″), 120.22 (C-5′′′), 119.27 (C-8′′′), 113.78 (C-4a′′′), 107.25 (C-2′′′), 65.41 (1′′′′-CH_2_), 44.54 (C-2), 41.02 (C-3), 25.28 ((CH_3_)_2_), 22.63 (C-1). IR (ATR): *ṽ* (cm^−1^) = 3307, 1733, 1617, 1506, 1296, 1199, 1036, 929, 697. HR-MS (ESI): *m*/*z* = [M + Na]^+^ calcd for C_30_H_29_BN_4_O_8_Na^+^: 607.1976; found: 607.1970. Specific rotation: [[α${]}_{D}^{20}$ +14 (c 0.1 in DMSO).

**(*S*)-4-(Benzyloxy)-1-bromo-4-oxobutan-2-aminium (44).** Prepared according to **General Procedure A** from *N*-Boc-protected amine **18** (520 mg, 1.40 mmol). The aminium **44** (380 mg, 1.39 mmol, quant.) was obtained as a white solid. m.p.: 109 °C. ^1^H-NMR (400 MHz, (CD_3_)_2_SO): δ (ppm) = 8.49 (s, 3H, NH_3_^+^), 7.45–7.29 (m, 5H, 2′-H, 3′-H, 4′-H, 5′-H and 6′-H), 5.15 (s, 2H, 1′-CH_2_), 3.88–3.72 (m, 3H, 1-H and 2-H), 2.87 (dd, *J* = 6.4, 1.4 Hz, 2H, 3-H). ^13^C-NMR (101 MHz, (CD_3_)_2_SO): δ (ppm) = 169.19 (C-4), 135.62 (C-1′), 128.47–128.13 (C-2′, C-3′, C-4′, C-5′ and C-6′), 66.30 (1′-CH_2_), 47.67 (C-2), 35.66 (C-3), 33.90 (C-1). IR (ATR): *ṽ* (cm^−1^) = 3203, 2866, 2812, 2590, 1720, 1708, 1501, 1395, 1348, 1227, 1154, 1138, 1082, 944, 739, 697. HR-MS (ESI): *m*/*z* = [M]^+^ calcd for C_11_H_15_BrNO_2_^+^: 272.0281; found: 272.0279. Specific rotation: [α${]}_{D}^{20}$ −4 (c 0.1 in DMSO).

**Benzyl (*S*)-2-(2-(4-ethynylphenyl)-4,5-dihydrooxazol-4-yl)acetate (45).** Prepared according to **General Procedure B** from aminium **44** (459 mg, 1.68 mmol) and 4-ethynylbenzoic acid (284 mg, 1.85 mmol). The reaction mixture was stirred at room temperature for 16 h. The crude product was purified by FCC (hexanes/EtOAc 75:25) to give oxazoline **45** (269 mg, 0.842 mmol, 50%) as a yellow solid. m.p.: 82 °C. ^1^H-NMR (400 MHz, (CD_3_)_2_SO): δ (ppm) = 7.86–7.82 (m, 2H, 2″-H and 6″-H), 7.60–7.55 (m, 2H, 3″-H and 5″-H), 7.39–7.28 (m, 5H, 2′′′-H, 3′′′-H, 4′′′-H, 5′′′-H and 6′′′-H), 5.13 (d, *J* = 2.0 Hz, 2H, 1′′′-CH_2_), 4.63–4.55 (m, 2H, 4′-H and 5′-H), 4.41 (s, 1H, C≡CH), 4.20–4.11 (m, 1H, 5′-H), 2.78–2.73 (m, 2H, 2-H). ^13^C-NMR (101 MHz, (CD_3_)_2_SO): δ (ppm) = 170.65 (C-1), 162.29 (C-2′), 136.08 (C-1′′′), 131.94 (C-3″ and C-5″), 128.40–127.97 (C-2′′′, C-3′′′, C-4′′′, C-5′′′ and C-6′′′), 127.84 (C-2″ and C-6″), 127.41 (C-1″), 124.73 (C-4″), 83.24 (**C**≡CH or C≡**C**H), 82.80 (**C**≡CH or C≡**C**H), 72.12 (C-5′), 65.53 (1′′′-CH_2_), 62.91 (C-4′), 39.50 (C-2). IR (ATR): *ṽ* (cm^−1^) = 3212, 2812, 2590, 1719, 1710, 1645, 1500, 1348, 1227, 1167, 1082, 954, 739, 697, 679. HR-MS (ESI): *m*/*z* = [M + H]^+^ calcd for C_20_H_18_NO_3_^+^: 320.1287; found: 320.1280. Specific rotation: [α${]}_{D}^{20}$ −42 (c 0.1 in DMSO).

**(*R*)-5-(5-(4-((1-Carboxy-3-cyanopropan-2-yl)carbamoyl)phenyl)isoxazol-3-yl)-2-hydroxybenzoic acid (47).** Prepared according to **General Procedure E** from isoxazole **34** (12.0 mg, 0.0212 mmol) and 0.70 M aq. KOH solution (5.95 mg, 0.106 mmol). The reaction mixture was stirred at room temperature for 2.5 h. The crude product was purified by FCC (hexanes/EtOAc 30:70 + 1% AcOH) to give isoxazole **47** (4.60 mg, 0.0106 mmol, 50%) as a white solid. m.p.: 228 °C. ^1^H-NMR (400 MHz, (CD_3_)_2_SO): δ (ppm) = 12.41 (s, 1H, 1-COOH or 1′′′-COOH), 8.85 (d, *J* = 7.8 Hz, 1H, CONH), 8.33 (d, *J* = 2.3 Hz, 1H, 6-H), 8.07 (s, 1H, 4-H), 8.03 (m, 4H, 2″-H, 3″-H, 5″-H and 6″-H), 7.77 (s, 1H, 4′-H), 7.11 (d, *J* = 8.6 Hz, 1H, 3-H), 4.54 (d, *J* = 7.2 Hz, 1H, 2′′′-H), 2.89 (d, *J* = 5.0 Hz, 2H, 3′′′-H), 2.67 (d, *J* = 7.9 Hz, 2H, 1′′′-H). ^13^C-NMR (126 MHz, (CD_3_)_2_SO): δ (ppm) = 171.53 (1′′′-COOH), 171.21 (1-COOH), 168.73 (C-5′), 165.27 (CONH), 163.49 (C-2), 161.95 (C-3′), 135.23 (C-4″), 132.99 (C-4), 129.32 (C-1″), 128.65 (C-6), 128.28 (C-3″ and C-5″), 125.51 (C-2″ and C-6″), 119.09 (C-5), 118.38 (CN), 118.09 (C-3), 114.62 (C-1), 99.62 (C-4′), 43.61 (C-2′′′), 37.66 (C-1″), 22.34 (C-3″). IR (ATR): *ṽ* (cm^−1^) = 2922, 1653, 1598, 1540, 1497, 1423, 1291, 1207, 1074, 948, 798, 768, 686. HR-MS (ESI): *m*/*z* = [M − H]^−^ calcd for C_22_H_16_O_7_N_3_^−^: 434.0988; found: 434.0986. Specific rotation: [α${]}_{D}^{20}$ −28 (c 0.1 in DMSO). Purity (HPLC): 210 nm: >95%; 254 nm: >95%.

**(*S*)-5-(5-(4-((1-Carboxy-3-cyanopropan-2-yl)carbamoyl)phenyl)isoxazol-3-yl)-2-hydroxybenzoic acid (48).** Prepared according to **General Procedure E** from isoxazole **35** (228 mg, 0.403 mmol) and 0.70 M aq. KOH solution (113 mg, 2.02 mmol). The reaction mixture was stirred at room temperature for 2.5 h. The crude product was purified by FCC (hexanes/EtOAc 30:70 + 1% AcOH) to give isoxazole **48** (95.6 mg, 0.220 mmol, 55%) as a white solid. m.p.: 231 °C. ^1^H-NMR (400 MHz, (CD_3_)_2_SO): δ (ppm) = 12.41 (s, 1H, 1-COOH or 1′′′-COOH), 8.85 (d, *J* = 7.8 Hz, 1H, CONH), 8.33 (d, *J* = 2.3 Hz, 1H, 6-H), 8.07 (s, 1H, 4-H), 8.03 (m, 4H, 2″-H, 3″-H, 5″-H and 6″-H), 7.77 (s, 1H, 4′-H), 7.11 (d, *J* = 8.6 Hz, 1H, 3-H), 4.54 (d, *J* = 7.2 Hz, 1H, 2′′′-H), 2.89 (d, *J* = 5.0 Hz, 2H, 3′′′-H), 2.67 (d, *J* = 7.9 Hz, 2H, 1′′′-H). ^13^C-NMR (126 MHz, (CD_3_)_2_SO): δ (ppm) = 171.53 (1′′′-COOH), 171.31 (1-COOH), 168.81 (C-5′), 165.26 (CONH), 162.53 (C-2), 161.82 (C-3′), 135.26 (C-4″), 133.35 (C-4), 129.28 (C-1″), 128.65 (C-6), 128.29 (C-3″ and C-5″), 125.52 (C-2″ and C-6″), 119.47 (C-5), 118.38 (CN), 118.22 (C-3), 113.88 (C-1), 99.64 (C-4′), 43.61 (C-2′′′), 37.66 (C-1″), 22.34 (C-3″). IR (ATR): *ṽ* (cm^−1^) = 2922, 1664, 1597, 1533, 1496, 1421, 1287, 1197, 797, 768, 686**.** HR-MS (ESI): *m*/*z* = [M − H]^−^ calcd for C_22_H_16_O_7_N_3_^−^: 434.0988; found: 434.0986. Specific rotation: [α${]}_{D}^{20}$ +28 (c 0.1 in DMSO). Purity (HPLC): 210 nm: >95%; 254 nm: >95%.

**(*R*)-5-(4-(4-((1-Carboxy-3-cyanopropan-2-yl)carbamoyl)phenyl)-1*H*-1,2,3-triazol-1-yl)-2-hydroxybenzoic acid (49).** Prepared according to **General Procedure E** from triazole **40** (60.0 mg, 0.106 mmol) and 0.70 M aq. KOH solution (29.8 mg, 0.530 mmol). The reaction mixture was stirred at room temperature for 30 min. The crude product was resuspended in EtOAc (10 mL), filtered and the solid residue collected to give cyanomethyl **49** (18.0 mg, 0.0413 mmol, 39%) as a white solid. m.p.: 290 °C. ^1^H-NMR (400 MHz, (CD_3_)_2_SO): δ (ppm) = 12.43 (s, 1H, 1-COOH or 1′′′-COOH), 9.42 (s, 1H, 5′-H), 8.74 (d, *J* = 7.7 Hz, 1H, CONH), 8.30 (d, *J* = 2.8 Hz, 1H, 6-H), 8.12–8.06 (m, 1H, 4-H), 8.08–8.04 (m, 2H, 2″-H and 6″-H), 8.01–7.94 (m, 2H, 3″-H and 5″-H), 7.22 (d, *J* = 9.0 Hz, 1H, 3-H), 4.58–4.48 (m, 1H, 2′′′-H), 2.98–2.80 (m, 2H, 3′′′-H), 2.77–2.60 (m, 2H, 1′′′-H). ^13^C-NMR (101 MHz, (CD_3_)_2_SO): δ (ppm) = 171.58 (1′′′-COOH), 170.84 (1-COOH), 165.60 (CONH), 160.93 (C-2), 146.40 (C-4′), 133.30 (C-4″), 133.19 (C-1″), 128.48 (C-5), 128.13 (C-3″ and C-5″), 127.37 (C-4), 125.01 (C-2″ and C-6″), 121.67 (C-6), 120.62 (C-5′), 118.68 (C-3), 118.42 (CN), 114.03 (C-1), 43.55 (C-2′′′), 37.71 (C-3′′′), 22.36 (C-1′′′). IR (ATR): *ṽ* (cm^−1^) = 3364, 3116, 1728, 1673, 1521, 1291, 1184, 1042, 829, 772, 691. HR-MS (ESI): *m*/*z* = [M + H]^+^ calcd for C_21_H_18_N_5_O_6_^+^: 436.1257; found: 436.1250. Specific rotation: [α${]}_{D}^{20}$ −18 (c 0.1 in DMSO). Purity (HPLC): 210 nm: >95%; 254 nm: >95%.

**(*S*)-5-(4-(4-((1-Carboxy-3-cyanopropan-2-yl)carbamoyl)phenyl)-1*H*-1,2,3-triazol-1-yl)-2-hydroxybenzoic acid (50).** Prepared according to **General Procedure E** from triazole **41** (120 mg, 0.212 mmol) and 0.70 M aq. KOH solution (59.5 mg, 1.06 mmol). The reaction mixture was stirred at room temperature for 30 min. The crude product was resuspended in EtOAc (10 mL), filtered and the solid residue collected to give cyanomethyl **50** (53.7 mg, 0.123 mmol, 58%) as a white solid. m.p.: 292 °C. ^1^H-NMR (400 MHz, (CD_3_)_2_SO): δ (ppm) = 12.44 (s, 1H, 1-COOH or 1′-COOH), 9.42 (s, 1H, 5′-H), 8.74 (d, *J* = 7.7 Hz, 1H, CONH), 8.30 (d, *J* = 2.8 Hz, 1H, 6-H), 8.11–8.08 (m, 1H, 4-H), 8.10–8.03 (m, 2H, 2″-H and 6″-H), 8.01–7.94 (m, 2H, 3″-H and 5″-H), 7.22 (d, *J* = 8.9 Hz, 1H, 3-H), 4.58–4.48 (m, 1H, 2′′′′-H), 2.97–2.81 (m, 2H, 3′′′-H), 2.77–2.61 (m, 2H, 1′′′-H). ^13^C-NMR (101 MHz, (CD_3_)_2_SO): δ (ppm) = 171.58 (1′′′-COOH), 170.85 (1-COOH), 165.60 (CONH), 160.91 (C-2), 146.40 (C-4′), 133.31 (C-4″), 133.19 (C-1″), 128.50 (C-5), 128.13 (C-3″ and C-5″), 127.40 (C-4), 125.01 (C-2″ and C-6″), 121.67 (C-6), 120.62 (C-5′), 118.69 (C-3), 118.42 (CN), 113.99 (C-1), 43.55 (C-2′′′), 37.71 (C-3′′′), 22.36 (C-1′′′). IR (ATR): *ṽ* (cm^−1^) = 3364, 3116, 1716, 1675, 1546, 1292, 1194, 1045, 828, 768, 691. HR-MS (ESI): *m*/*z* = [M − H]^−^ calcd for C_21_H_16_N_5_O_6_^−^: 434.1101; found: 434.1102. Specific rotation: [α${]}_{D}^{20}$ +12 (c 0.1 in DMSO). Purity (HPLC): 210 nm: >95%; 254 nm: >95%.

**(*R*)-2-(6-(4-(3-(2,2-Dimethyl-4-oxo-4*H*-benzo[*d*][1,3]dioxin-6-yl)isoxazol-5-yl)phenyl)-2-hydroxy-3,4-dihydro-2*H*-1,5,2-oxazaborinin-4-yl)acetic acid (51).** Prepared according to G**eneral Procedure E** from isoxazole **36** (30.0 mg, 0.0590 mmol) and 0.40 M aq. KOH solution (4.97 mg, 0.0885 mmol). The reaction mixture was stirred at 0 °C for 30 min. The crude product was resuspended in DCM (10 mL), filtered and the solid residue collected to give cyclic boronic acid **51** (15.1 mg, 0.0317 mmol, 54%) as an off-white solid. m.p.: 233 °C. ^1^H-NMR at 90 °C (400 MHz, (CD_3_)_2_SO): δ (ppm) = 8.41–8.37 (m, 1H, 5′′′′-H), 8.25–8.19 (m, 1H, 7′′′′-H), 8.09–7.97 (m, 4H, 2″-H, 3″-H, 5″-H and 6″-H), 7.77–7.68 (m, 1H, 4′′′-H), 7.29 (d, *J* = 8.6 Hz, 1H, 8′′′′-H), 4.27–4.10 (m, 1H, 4′-H), 3.78–3.53 (m, 2H, 2-H or 3′-H), 2.69–2.54 (m, 2H, 2-H or 3′-H), 1.76 (s, 6H, (CH_3_)_2_). ^13^C-NMR (126 MHz, (CD_3_)_2_SO): δ (ppm) = 172.68 (C-1), 169.16 (C-5′′′), 166.79 (C-6′), 161.45 (C-3′′′), 159.75 (C-4′′′′), 156.81 (C-8a′′′′), 134.79 (C-1″ and C-7′′′′), 128.75 (C-4″), 128.37 (C-2″ and C-6″ or C-3″ and C-5″), 127.36 (C-5′′′′), 125.87–125.51 (C-2″ and C-6″ or C-3″ and C-5″), 123.23 (C-6′′′′), 118.56 (C-8′′′′), 113.56 (C-4a′′′′), 107.02 (C-2′′′′), 99.66 (C-4′′′), 64.03 (C-2 or C-3′), 48.18 (C-4′), 25.33 ((CH_3_)_2_). IR (ATR): *ṽ* (cm^−1^) = 3122, 2995, 1736, 1619, 1287, 1199, 1016, 927, 763, 674. HR-MS (ESI): *m*/*z* = [M + H]^+^ calcd for C_24_H_22_BN_2_O_8_^+^: 477.1469; found: 477.1427. Specific rotation: [α${]}_{D}^{20}$ −6 (c 0.1 in DMSO). Purity (HPLC): 210 nm: 94%; 254 nm: >95%.

**(*S*)-2-(6-(4-(3-(2,2-Dimethyl-4-oxo-4*H*-benzo[*d*][1,3]dioxin-6-yl)isoxazol-5-yl)phenyl)-2-hydroxy-3,4-dihydro-2*H*-1,5,2-oxazaborinin-4-yl)acetic acid (52).** Prepared according to **General Procedure E** from isoxazole **37** (20.0 mg, 0.0393 mmol) and 0.40 M aq. KOH solution (3.31 mg, 0.0590 mmol). The reaction mixture was stirred at 0 °C for 30 min. The crude product was resuspended in DCM (10 mL), filtered and the solid residue collected to give cyclic boronic acid **52** (10.4 mg, 0.0218 mmol, 56%) as an off-white solid. m.p.: 188 °C. ^1^H-NMR at 90 °C (400 MHz, (CD_3_)_2_SO): δ (ppm) = 8.41–8.37 (m, 1H, 5′′′′-H), 8.22 (dd, *J* = 8.6, 2.2 Hz, 1H, 7′′′′-H), 8.08–7.95 (m, 4H, 2″-H, 3″-H, 5″-H and 6″-H), 7.75–7.69 (m, 1H, 4′′′-H), 7.29 (d, *J* = 8.7 Hz, 1H, 8′′′′-H), 4.41–4.00 (m, 1H, 4′-H), 3.70–3.41 (m, 2H, 2-H or 3′-H), 2.63–2.56 (m, 1H, 2-H or 3′-H), 2.41–2.31 (m, 1H, 2-H or 3′-H), 1.76 (s, 6H, (CH_3_)_2_). ^13^C-NMR (101 MHz, (CD_3_)_2_SO): δ (ppm) = 161.07 (C-3″), 159.11 (C-4′′′′), 156.42 (C-8a′′′′), 134.33 (C-7′′′′ and C-1″), 127.92 (C-2″ and C-6″ or C-3″ and C-5″), 126.91 (C-5′′′′), 125.28 (C-2″ and C-6″ or C-3″ and C-5″), 123.05 (C-6′′′′), 117.93 (C-8′′′′), 113.27 (C-4a′′′′), 106.50 (C-2′′′′), 25.01 ((CH_3_)_2_). IR (ATR): *ṽ* (cm^−1^) = 3118, 2918, 1738, 1619, 1287, 1199, 1018, 926, 762, 674. HR-MS (ESI): *m*/*z* = [M − H]^−^ calcd for C_24_H_20_BN_2_O_8_^−^: 475.1313; found: 475.1294. Specific rotation: [α${]}_{D}^{20}$ +4 (c 0.1 in DMSO). Purity (HPLC): 210 nm: >95%; 254 nm: >95%.

**(*R*)-2-(6-(4-(1-(2,2-Dimethyl-4-oxo-4*H*-benzo[*d*][1,3]dioxin-6-yl)-1*H*-1,2,3-triazol-4-yl)phenyl)-2-hydroxy-3,4-dihydro-2*H*-1,5,2-oxazaborinin-4-yl)acetic acid (53).** Prepared according to **General Procedure E** from triazole **42** (180 mg, 0.308 mmol) and 0.40 M aq. KOH solution (25.9 mg, 0.462 mmol). The reaction mixture was stirred at 0 °C for 1 h. The crude product was purified by FCC with boric acid-impregnated silica gel (DCM/MeOH 90:10 + 1% AcOH) to give cyclic boronic acid **53** (56.5 mg, 0.119 mmol, 39%) as a white solid. m.p.: 292 °C (decomposition). ^1^H-NMR (400 MHz, CD_3_OD): δ (ppm) = 9.08 (s, 1H, 5″-H), 8.42 (d, *J* = 2.7 Hz, 1H, 5′′′′-H), 8.22 (dd, *J* = 8.9, 2.7 Hz, 1H, 7′′′′-H), 8.07–8.02 (m, 2H, 3″-H and 5″-H), 7.97–7.92 (m, 2H, 2″-H and 6″-H), 7.31 (d, *J* = 8.9 Hz, 1H, 8′′′′-H), 4.38–4.29 (m, 1H, 4′-H), 2.74–2.66 (m, 1H, 2-H), 2.56–2.47 (m, 1H, 2-H), 1.80 (s, 6H, (CH_3_)_2_), 0.92–0.83 (m, 1H, 3′-H), 0.77–0.68 (m, 1H, 3′-H). ^13^C-NMR (101 MHz, CD_3_OD): δ (ppm) = 174.68 (C-1), 168.94 (C-6′), 161.47 (C-4′′′′), 157.46 (C-8a′′′′), 148.62 (C-4′′′), 136.12 (C-1′ and C-4″), 133.45 (C-6′′′′), 129.95 (C-7′′′′), 129.18 (C-2″ and C-6″), 126.84 (C-3″ and C-5″), 121.99 (C-5′′′′), 121.34 (C-5′′′), 120.35 (C-8′′′′), 115.55 (C-4a′′′′), 108.66 (C-2′′′′), 47.39 (C-4′), 40.50 (C-2), 25.86 ((CH_3_)_2_). IR (ATR): *ṽ* (cm^−1^) = 3343, 1733, 1618, 1509, 1379, 1299, 1044, 828, 767. HR-MS (ESI): *m*/*z* = [M + H]^+^ calcd for C_23_H_22_BN_4_O_7_^+^: 477.1582; found: 477.1564. Specific rotation: [α${]}_{D}^{20}$ −12 (c 0.1 in DMSO). Purity (HPLC): 210 nm: >95%; 254 nm: >95%.

**(*S*)-2-(6-(4-(1-(2,2-Dimethyl-4-oxo-4*H*-benzo[*d*][1,3]dioxin-6-yl)-1*H*-1,2,3-triazol-4-yl)phenyl)-2-hydroxy-3,4-dihydro-2*H*-1,5,2-oxazaborinin-4-yl)acetic acid (54).** Prepared according to **General Procedure E** from triazole **43** (205 mg, 0.351 mmol) and 0.40 M aq. KOH solution (29.5 mg, 0.526 mmol). The reaction mixture was stirred at 0 °C for 1 h. The crude product was purified by FCC with boric acid-impregnated silica gel (DCM/MeOH 90:10 + 1% AcOH) to give cyclic boronic acid **54** (53.0 mg, 0.111 mmol, 32%) as a pale yellow solid. m.p.: 304 °C (decomposition). ^1^H-NMR (500 MHz, CD_3_OD): δ (ppm) = 9.10 (s, 1H, 5′′′-H), 8.42 (d, *J* = 2.7 Hz, 1H, 5′′′′-H), 8.23 (dd, *J* = 8.9, 2.7 Hz, 1H, 7′′′′-H), 8.10–8.05 (m, 2H, 3″-H and 5″-H), 8.00–7.95 (m, 2H, 2″-H and 6″-H), 7.31 (d, *J* = 9.0 Hz, 1H, 8′′′′-H), 4.36–4.26 (m, 1H, 4′-H), 2.76–2.67 (m, 1H, 2-H), 2.64–2.50 (m, 1H, 2-H), 1.80 (s, 6H, (CH_3_)_2_), 0.92–0.86 (m, 1H, 3′-H), 0.82–0.73 (m, 1H, 3′-H). ^13^C-NMR (126 MHz, CD_3_OD): δ (ppm) = 175.06 (C-1), 169.18 (C-6′), 161.46 (C-4′′′′), 157.48 (C-8a′′′′), 148.51 (C-4′′′), 135.11 (C-1″ and C-4″), 133.43 (C-6′′′′), 129.95 (C-7′′′′), 129.33 (C-2″ and C-6″), 126.91 (C-3″ and C-5″), 121.99 (C-5′′′′), 121.46 (C-5′′′), 120.36 (C-8′′′′), 115.56 (C-4a′′′′), 108.66 (C-2′′′′), 45.77 (C-4′), 40.53 (C-2), 25.86 ((CH_3_)_2_). IR (ATR): *ṽ* (cm^−1^) = 3343, 1733, 1616, 1508, 1378, 1298, 1041, 826, 766. HR-MS (ESI): *m*/*z* = [M + H]^+^ calcd for C_23_H_22_BN_4_O_7_^+^: 477.1582; found: 477.1583. Specific rotation: [α${]}_{D}^{20}$ +8 (c 0.1 in DMSO). Purity (HPLC): 210 nm: >95%; 254 nm: >95%.

**1-(Benzyloxy)-1-oxohex-5-en-3-aminium (57).** Prepared according to **General Procedure A** from *N*-Boc-protected homoallyl amine **56** (1.37 g, 4.29 mmol). The aminium **57** (945 mg, 4.31 mmol, quant.) was obtained as a viscous yellow oil. ^1^H-NMR (400 MHz, (CD_3_)_2_SO): δ (ppm) = 8.22 (s, 3H, NH_3_^+^), 7.40–7.32 (m, 5H, 2′-H, 3′-H, 4′-H, 5′-H and 6′-H), 5.83–5.71 (m, 1H, 5-H), 5.17–5.10 (m, 4H, 6-H and 1′-CH_2_), 3.58–3.48 (m, 1H, 3-H), 2.81–2.64 (m, 2H, 2-H), 2.47–2.30 (m, 2H, 4-H). ^13^C-NMR (101 MHz, (CD_3_)_2_SO): δ (ppm) = 169.74 (C-1), 135.71 (C-1′), 132.41 (C-5), 128.45 -128.18 (2′-H, 3′-H, 4′-H, 5′-H and 6′-H), 119.51 (C-6), 66.10 (1′-CH_2_), 46.86 (C-3), 36.42 (C-4), 36.09 (C-2). IR (ATR): *ṽ* (cm^−1^) = 2977, 2880, 2834, 1998, 1728, 1601, 1497, 1395, 1216, 1190, 1128, 923, 738, 696. HR-MS (ESI): *m*/*z* = [M]^+^ calcd for C_13_H_18_NO_2_^+^: 220.1332; found: 220.1353.

**Benzyl 3-(4-ethynylbenzamido)hex-5-enoate (58).** Prepared according to **General Procedure B** from aminium **57** (490 mg, 2.23 mmol) and 4-ethynylbenzoic acid (344 mg, 2.23 mmol). The crude product was purified by FCC (hexanes/EtOAc 80:20) to give amide **58** (629 mg, 1.81 mmol, 81%) as a pale yellow solid. m.p.: 104 °C. ^1^H-NMR (400 MHz, (CD_3_)_2_SO): δ (ppm) = 8.42 (d, *J* = 8.4 Hz, 1H, CONH), 7.81–7.77 (m, 2H, 2′-H and 6′-H), 7.57–7.54 (m, 2H, 3′-H and 5′-H), 7.33–7.27 (m, 5H, 2″-H, 3″-H, 4″-H, 5″-H and 6″-H), 5.77 (ddt, *J* = 17.2, 10.2, 7.0 Hz, 1H, 5-H), 5.10–4.99 (m, 4H, 6-H and 1″-CH_2_), 4.45–4.38 (m, 1H, 3-H), 4.37 (s, 1H, C≡CH), 2.66–2.61 (m, 2H, 2-H), 2.33–2.28 (m, 2H, 4-H). ^13^C-NMR (101 MHz, (CD_3_)_2_SO): δ (ppm) = 170.67 (C-1), 164.98 (CONH), 136.08 (C-1″), 134.92 (C-5), 134.62 (C-1′), 131.55 (C-3′ and C-5′), 128.31–127.86 (C-2″, C-3″, C-4″, C-5″ and C-6″), 127.50 (C-2′ and C-6′), 124.32 (C-4′), 117.42 (C-6), 82.90 (**C**≡CH and C≡**C**H), 65.54 (1″-CH_2_), 46.34 (C-3), 38.63 (C-2), 38.46 (C-4). IR (ATR): *ṽ* (cm^−1^) = 3317, 3271, 1737, 1716, 1638, 1543, 1536, 1495, 1301, 1258, 1164, 1116, 854, 757, 698, 675. HR-MS (ESI): *m*/*z* = [M + H]^+^ calcd for C_22_H_22_NO_3_^+^: 348.1600; found: 348.1607.

**Benzyl 3-(4-(3-(2,2-dimethyl-4-oxo-4*H*-benzo[*d*][1,3]dioxin-6-yl)isoxazol-5-yl)benzamido)hex-5-enoate (59).** To a stirred solution of alkyne **58** (844 mg, 2.43 mmol) in 24 mL MeOH/H_2_O (5:1 *v*/*v*), aldoxime **3** (806 mg, 3.64 mmol) and PIFA (403 mg, 1.82 mmol) were added. The reaction mixture was stirred at 70 °C for 2 h. Another equivalent of PIFA (403 mg, 1.82 mmol) was added, and the reaction mixture was stirred at 70 °C for another 2 h. The solvents were removed in vacuo, and the crude product was redissolved in EtOAc (150 mL). Water (150 mL) was added, the resulting two phases were separated, and the aqueous phase was extracted with EtOAc (3 × 150 mL). The combined organic phases were dried using a phase separation paper, and the solvent was removed in vacuo. The crude product was purified by FCC (hexanes/EtOAc 70:30) to give isoxazole **59** (163.2 mg, 0.290 mmol, 12%) as a white solid. m.p.: 173 °C. ^1^H-NMR (500 MHz, CDCl_3_): δ (ppm) = 8.37 (d, *J* = 2.2 Hz, 1H, 5′′′-H), 8.19 (dd, *J* = 8.6, 2.2 Hz, 1H, 7′′′-H), 7.89–7.86 (m, 2H, 3′-H and 5′-H), 7.84–7.81 (m, 2H, 2′-H and 6′-H), 7.39–7.31 (m, 5H, 2′′′′-H, 3′′′′-H, 4′′′′-H, 5′′′′-H and 6′′′′-H), 7.11 (d, *J* = 8.6 Hz, 1H, 8′′′-H), 6.97 (d, *J* = 8.7 Hz, 1H, CONH), 6.94 (s, 1H, 4″-H), 5.81 (ddt, *J* = 19.5, 9.6, 7.1 Hz, 1H, 5-H), 5.20–5.09 (m, 4H, 6-H and 1′′′′-CH_2_), 4.60–4.52 (m, 1H, 3-H), 2.74 (d, *J* = 5.0 Hz, 2H, 2-H), 2.54–2.38 (m, 2H, 4-H), 1.79 (s, 6H, (CH_3_)_2_). ^13^C-NMR (126 MHz, CDCl_3_): δ (ppm) = 171.98 (C-1), 169.88 (C-5″), 165.75 (CONH), 161.72 (C-3″), 160.74 (C-4′′′), 157.46 (C-8a′′′), 136.06 (C-1′), 135.57 (C-1′′′′), 134.69 (C-7′′′), 133.95 (C-5), 129.88 (C-4′), 128.85–128.57 (C-2′′′′, C-3′′′′, C-4′′′′, C-5′′′′ and C-6′′′′), 128.35 (C-5′′′), 127.89 (C-2′ and C-6′), 126.12 (C-3′ and C-5′), 123.97 (C-6′′′), 118.77 (C-6), 118.39 (C-8′′′), 113.90 (C-4a′′′), 107.10 (C-2′′′), 98.46 (C-4″), 66.91 (1′′′′-CH_2_), 46.21 (C-3), 38.59 (C-4), 37.59 (C-2), 26.04 ((CH_3_)_2_). IR (ATR): *ṽ* (cm^−1^) = 3300, 2922, 2851, 1748, 1720, 1634, 1627, 1534, 1429, 1284, 1200, 1050, 922, 914, 822, 763, 688. HR-MS (ESI): *m*/*z* = [M + H]^+^ calcd for C_33_H_31_N_2_O_7_^+^: 567.2131; found: 567.2117.

**5-(5-(4-((1-Carboxypent-4-en-2-yl)carbamoyl)phenyl)isoxazol-3-yl)-2-hydroxybenzoic acid (60).** Prepared according to **General Procedure E** from isoxazole **59** (165 mg, 0.291 mmol) and 0.70 M aq. KOH solution (81.4 mg, 1.45 mmol). The reaction mixture was stirred at room temperature for 1.5 h. The crude product was resuspended in EtOAc (20 mL), filtered and the solid residue collected to give vinyl **60** (127 mg, 0.291 mmol, quant.) as a white solid. m.p.: 225 °C. ^1^H-NMR (500 MHz, (CD_3_)_2_SO): δ (ppm) = 12.19 (s, 1H, 1-COOH or 1′′′-COOH), 8.45 (d, *J* = 8.4 Hz, 1H, CONH), 8.34 (d, *J* = 2.3 Hz, 1H, 6-H), 8.06 (dd, *J* = 8.7, 2.3 Hz, 1H, 4-H), 8.05–7.99 (m, 2H, 2′′-H and 6′′-H), 8.01–7.95 (m, 2H, 3′′-H and 5′′-H), 7.76 (s, 1H, 4′-H), 7.14 (d, *J* = 8.6 Hz, 1H, 3-H), 5.81 (ddt, *J* = 17.1, 10.2, 7.0 Hz, 1H, 4′′′-H), 5.11–5.01 (m, 2H, 5′′′-H), 4.43–4.34 (m, 1H, 2′′′-H), 2.57–2.51 (m, 2H, 1′′′-H), 2.37–2.30 (m, 2H, 3′′′-H). ^13^C-NMR (126 MHz, (CD_3_)_2_SO): δ (ppm) = 172.42 (1′′′-COOH), 171.32 (1-COOH), 168.91 (C-5′), 164.84 (CONH), 162.53 (C-2), 161.80 (C-3′), 136.02 (C-4″), 135.10 (C-4′′′), 133.35 (C-4), 128.94 (C-1″), 128.65 (C-6), 128.16 (C-3″ and C-5″), 125.42 (C-2″ and C-6″), 119.50 (C-5), 118.22 (C-3), 117.31 (C-5′′′), 113.88 (C-1), 99.48 (C-4′), 46.26 (C-2′′′), 38.71 (C-1′′′), 38.45 (C-3′′′). IR (ATR): *ṽ* (cm^−1^) = 3315, 2916, 1729, 1688, 1632, 1589, 1531, 1499, 1421, 1290, 1212, 926, 796, 769, 695. HR-MS (ESI): *m*/*z* = [M − H]^−^ calcd for C_23_H_19_N_2_O_7_^−^: 435.1192; found: 435.1192.

**5-(5-(4-((1-Carboxy-4-oxobutan-2-yl)carbamoyl)phenyl)isoxazol-3-yl)-2-hydroxybenzoic acid (61).** A solution of vinyl **60** (85 mg, 0.195 mmol) in 7 mL MeOH was flushed with N_2_ and cooled to -78 °C. O_3_ (flowrate: 25–30, power: 35%) was then bubbled into the solution for 3.5 min. Me_2_S (21.7 µL, 0.292 mmol) was added and the solution stirred at room temperature for 1 h. The solvent was removed in vacuo and the crude product purified by PTLC (DCM/MeOH 88:12 + 1% AcOH) to give formylmethyl **61** (31.4 mg, 0.0716 mmol, 37%) as a beige solid. m.p.: 275 °C. ^1^H-NMR (500 MHz, (CD_3_)_2_SO): δ (ppm) = 9.73 (s, 1H, CONH), 9.68 (s, 1H, 4′′′-H), 8.23 (d, *J* = 2.4 Hz, 1H, 6-H), 8.04–8.00 (m, 2H, 2′′-H and 6″-H), 7.94–7.90 (m, 2H, 3″-H and 5″-H), 7.71 (dd, *J* = 8.5, 2.4 Hz, 1H, 4-H), 7.61 (s, 1H, 4′-H), 6.74 (d, *J* = 8.4 Hz, 1H, 3-H), 4.63–4.53 (m, 1H, 2′′′-H), 2.76–2.55 (m, 2H, 3′′′-H), 2.32–2.22 (m, 2H, 1′′′-H). ^13^C-NMR (126 MHz, (CD_3_)_2_SO): δ (ppm) = 202.04 (C-4′′′), 174.07 (1′′′-COOH), 170.65 (1-COOH), 168.14 (C-5′), 166.02 (C-2), 164.19 (CONH), 162.94 (C-3′), 135.74 (C-4″), 129.76 (C-4), 129.28 (C-1″), 128.61 (C-6), 127.76 (C-3″ and C-5″), 125.50 (C-2″ and C-6″), 120.44 (C-5), 116.97 (C-3), 115.53 (C-1), 99.33 (C-4′), 48.90 (C-3′′′), 43.15 (C-2′′′), 41.28 (C-1′′′). IR (ATR): *ṽ* (cm^−1^) = 3300, 2925, 1715, 1635, 1560, 1497, 1392, 1257, 1077, 948, 834, 768, 700. HR-MS (ESI): *m*/*z* = [M − H]^−^ calcd for C_22_H_17_N_2_O_8_^−^: 437.0985; found: 437.0988. Purity (HPLC): 210 nm: 90%; 254 nm: 91%.

**Benzyl 3-(4-(1-(2,2-dimethyl-4-oxo-4*H*-benzo[*d*][1,3]dioxin-6-yl)-1*H*-1,2,3-triazol-4-yl)benzamido)hex-5-enoate (62).** Prepared according to **General Procedure D** from azide **11** (300 mg, 1.37 mmol) and alkyne **58** (571 mg, 1.64 mmol). The crude product was resuspended in EtOAc (20 mL), filtered and the solid residue collected to give triazole **62** (550 mg, 0.971 mmol, 71%) as a white solid. m.p.: 173 °C. ^1^H-NMR (400 MHz, (CD_3_)_2_SO): δ (ppm) = 9.53 (s, 1H, 5′′-H), 8.42–8.38 (m, 2H, CONH and 5′′′-H), 8.31 (dd, *J* = 8.9, 2.7 Hz, 1H, 7′′′-H), 8.06–8.00 (m, 2H, 3′-H and 5′-H), 7.96–7.90 (m, 2H, 2′-H and 6′-H), 7.43 (d, *J* = 8.9 Hz, 1H, 8′′′-H), 7.35–7.27 (m, 5H, 2′′′′-H, 3′′′′-H, 4′′′′-H, 5′′′′-H and 6′′′′-H), 5.87–5.74 (m, 1H, 5-H), 5.13–5.01 (m, 4H, 6-H and 1′′′′-CH_2_), 4.50–4.40 (m, 1H, 3-H), 2.69–2.64 (m, 2H, 2-H), 2.34 (t, *J* = 6.9 Hz, 2H, 4-H), 1.77 (s, 6H, (CH_3_)_2_). ^13^C-NMR (101 MHz, (CD_3_)_2_SO): δ (ppm) = 170.73 (C-1), 165.27 (CONH), 159.48 (C-4′′′), 155.22 (C-8a′′′), 146.67 (C-4″), 136.11 (C-1′′′′), 134.99 (C-5), 134.08 (C-1′), 132.66 (C-4′), 131.70 (C-6′′′), 128.67 (C-7′′′), 128.33 (C-2′′′′, C-3′′′′, C-4′′′′, C-5′′′′ and C-6′′′′), 128.04 (C-2′ and C-6′), 127.92–127.87 (C-2′′′′, C-3′′′′, C-4′′′′, C-5′′′′ and C-6′′′′), 124.95 (C-3′ and C-5′), 120.69 (C-5″), 120.20 (C-5′′′), 119.25 (C-8′′′), 117.41 (C-6), 113.77 (C-4a′′′), 107.23 (C-2′′′), 65.55 (1′′′′-CH_2_), 46.33 (C-3), 38.71 (C-2 or C-4), 38.53 (C-2 or C-4), 25.27 ((CH_3_)_2_). IR (ATR): *ṽ* (cm^−1^) = 3313, 1735, 1630, 1507, 1295, 1170, 1047, 932, 851, 697. HR-MS (ESI): *m*/*z* = [M + H]^+^ calcd for C_32_H_31_N_4_O_6_^+^: 567.2244; found: 567.2259.

**5-(4-(4-((1-Carboxypent-4-en-2-yl)carbamoyl)phenyl)-1*H*-1,2,3-triazol-1-yl)-2-hydroxybenzoic acid (63).** Prepared according to **General Procedure E** from triazole **62** (422 mg, 0.745 mmol) and 0.70 M aq. KOH solution (208 mg, 3.71 mmol). The reaction mixture was stirred at room temperature for 1.5 h. The crude product was resuspended in EtOAc (50 mL), filtered and the solid residue collected to give vinyl **63** (249 mg, 0.570 mmol, 77%) as a white solid. m.p.: 223 °C. ^1^H-NMR (400 MHz, (CD_3_)_2_SO): δ (ppm) = 12.12 (s, 1H, 1-COOH or 1′′′-COOH), 9.40 (s, 1H, 5′-H), 8.34 (d, *J* = 8.4 Hz, 1H, CONH), 8.30 (d, *J* = 2.8 Hz, 1H, 6-H), 8.09 (dd, *J* = 8.9, 2.8 Hz, 1H, 4-H), 8.06–7.99 (m, 2H, 2′′-H and 6′′-H), 7.98–7.90 (m, 2H, 3′′-H and 5′′-H), 7.22 (d, *J* = 8.9 Hz, 1H, 3-H), 5.87–5.75 (m, 1H, 4′′′-H), 5.13–5.00 (m, 2H, 5′′′-H), 4.44–4.34 (m, 1H, 2′′′-H), 2.52–2.48 (m, 2-H, 1′′′-H), 2.33 (t, *J* = 6.9 Hz, 2H, 3′′′-H). ^13^C-NMR (126 MHz, (CD_3_)_2_SO): δ (ppm) = 172.47 (1′′′-COOH), 170.87 (1-COOH), 165.19 (CONH), 160.89 (C-2), 146.49 (C-4′), 135.17 (C-4′′′), 134.06 (C-4″), 132.80 (C-1″), 128.52 (C-5), 128.00 (C-3″ and C-5″), 127.40 (C-4), 124.93 (C-2″ and C-6″), 121.65 (C-6), 120.50 (C-5′), 118.70 (C-3), 117.26 (C-5′′′), 113.96 (C-1), 46.18 (C-2′′′), 38.77 (C-1′′′), 38.49 (C-3′′′). IR (ATR): *ṽ* (cm^−1^) = 3119, 1723, 1669, 1593, 1546, 1492, 1288, 1209, 1182, 1046, 828, 689. HR-MS (ESI): *m*/*z* = [M + H]^+^ calcd for C_22_H_21_N_4_O_6_^+^: 437.1461; found: 437.1471.

**5-(4-(4-((1-Carboxy-4-oxobutan-2-yl)carbamoyl)phenyl)-1*H*-1,2,3-triazol-1-yl)-2-hydroxybenzoic acid (64).** A solution of vinyl **63** (100 mg, 0.229 mmol) in 8 mL MeOH was flushed with N_2_ and cooled to −78 °C. O_3_ (flowrate: 25–30, power: 35%) was then bubbled into the solution for 3.5 min. Me_2_S (25.5 µL, 0.344 mmol) was added and the solution stirred at room temperature for 1 h. The solvent was removed in vacuo and the crude product purified by PTLC (DCM/MeOH 85:15 + 1% AcOH) to give aldehyde **64** (18.9 mg, 0.0431 mmol, 19%) as a beige solid. m.p.: 151 °C (decomposition). ^1^H-NMR (500 MHz, (CD_3_)_2_SO): δ (ppm) = 9.69 (s, 1H, 4′′′-H), 9.65 (s, 1H, CONH), 9.27 (s, 1H, 5′-H), 8.13 (d, *J* = 2.9 Hz, 1H, 6-H), 8.06–8.01 (m, 2H, 2′′-H and 6″-H), 7.89–7.85 (m, 2H, 3″-H and 5″-H), 7.69 (dd, *J* = 8.7, 2.9 Hz, 1H, 4-H), 6.82 (d, *J* = 8.7 Hz, 1H, 3-H), 4.61–4.55 (m, 1H, 2′′′-H), 2.76–2.55 (m, 2H, 3′′′-H), 2.30–2.20 (m, 2H, 1′′′-H). ^13^C-NMR (126 MHz, (CD_3_)_2_SO): δ (ppm) = 202.17 (C-4′′′), 174.34 (1′′′-COOH), 170.08 (1-COOH), 164.53 (CONH), 164.03 (C-2), 146.09 (C-4′), 133.81 (C-4″), 133.18 (C-1″), 127.57 (C-3″ and C-5″), 125.87 (C-5), 125.00 (C-2″ and C-6″), 123.76 (C-4), 121.70 (C-6), 120.71 (C-1), 120.23 (C-5′), 117.03 (C-3), 49.01 (C-3′′′), 43.08 (C-2′′′), 41.44 (C-1′′′). IR (ATR): *ṽ* (cm^−1^) = 3300, 2918, 1716, 1636, 1576, 1487, 1374, 1252, 1043, 829, 769, 706. HR-MS (ESI): *m*/*z* = [M-H]^−^ calcd for C_21_H_17_N_4_O_7_^−^: 437.1097; found: 437.1102. Purity (HPLC): 210 nm: 94%; 254 nm: 92%.

### 3.2. Sirtuin 5 Assay

The inhibitory activities of the synthesized target compounds were determined by Reaction Biology Corporation (Malvern, PA, USA) using a fluorescence-based assay. The sirtuin 5 enzyme utilized in this assay was produced in-house by Reaction Biology. The enzyme construct (accession number: NM_012241) comprised amino acids 37–310 (C-terminal region), carried an *N*-terminal His-tag and was expressed in *Escherichia coli*. The recombinant protein was purified to >95% purity, as confirmed by SDS-PAGE, and supplied in a solution containing 50 mM Tris-HCl (pH 8.0), 137 mM NaCl, 2.7 mM KCl, 1 mM MgCl_2_, 1 mg/mL BSA, and 1% DMSO with a protein concentration of 19 nM. Test compounds were prepared at a concentration of 10 mM in DMSO and incubated with 5 µL sirtuin 5 in the assay buffer per well at 30 °C for 10 min in a concentration range between 5 nM and 0.1 mM. 5 µL assay buffer without sirtuin 5 were used for control wells. The deacylation reaction was initiated by the addition of 5 µL substrate mixture comprising 50 µM fluorogenic substrate Ac-Lys-succ-AMC and the co-factor NAD^+^. After a 2 h incubation at 30 °C, the reaction was terminated by adding 10 µL protease-based developer with 4 mM nicotinamide, which cleaves 7-amino-4-methylcoumarin to yield a fluorescent signal. Fluorescence was measured after an additional 1 h incubation at 30 °C, with excitation/emission wavelengths of 360/460 nm with EnVision^®^ Plate Reader (Revvity, Waltham, MA, USA). To standardize results, a no-inhibitor control representing 100% enzyme activity was included in all assays. IC_50_ values for sirtuin 5 inhibition were determined in triplicate using a 10-dose, 3-fold serial dilution series. For each replicate, individual IC_50_ values were calculated by fitting sigmoidal dose–response curves using Prism 8.0.2 software (GraphPad Software, Boston, MA, USA). The final IC_50_ values are reported as the mean ± standard deviation of the triplicate measurements.

### 3.3. Computational Methods

Molecular docking studies were performed using the Schrödinger software suite (version 2020-3, Schrödinger Inc., New York, NY, USA) [44]. X-ray crystal structure of sirtuin 5 in complex with its reference succinyl peptide substrate (PDB ID: 3RIY [30]) was obtained from the Protein Data Bank [45] and prepared using the Protein Preparation Wizard. Ligands were prepared with the Ligand Preparation Wizard, employing Epik for protonation state and charge assignments with a pH value of 7.4 assumed [46]. The protein was prepared without the ligand in the active site. The center of mass of ligand 2 in Chain A of 3RIY was set as center of the docking grid (x = −12.8, y = 2.41 and z = −6.59). Docking was conducted using Glide in standard precision (SP) mode with default parameters. OPLS4 force field was applied for structural optimization and docking. The resulting poses were analyzed and visualized using PyMOL 2.5.8 (Schrödinger Inc., New York, NY, USA). Top-ranked docking poses (5 poses) were evaluated based on their spatial alignment and interactions relative to the co-crystallized succinyl peptide substrate.

## 4. Conclusions

A growing body of literature implicating sirtuin 5 with the development and exacerbation of various diseases has solidified this NAD^+^-dependent lysine deacylase as a promising biological target for pharmaceutical interventions. However, the accessibility to potent sirtuin 5 inhibitors with satisfactory pharmacokinetic properties remains limited. Through a comprehensive SAR analysis of balsalazide, we previously generated a series of derivatives with optimized pharmacokinetic properties and further attempted to optimize their potency through various inhibitor–enzyme interactions. Herein, we continued this endeavor by employing the principles of reversible covalent bonding, a well-established optimization method in drug development, to potentially enhance the binding affinity and potency of these sirtuin 5 inhibitors. Guided by initial docking experiments, the introduction of boronic acid, cyanomethyl and formylmethyl groups was rationally employed at the most optimal position for reversible covalent bonding with the nicotinamide ribose vicinal hydroxy groups of the essential co-factor NAD^+^. Challenges associated with the syntheses and purification of alkyl boronic acids, as well as the syntheses of enantiomerically pure derivatives, were addressed and navigated through method developments and optimizations, and chiral-pool syntheses from commercially available amino acid derivatives. Biological evaluation of the synthesized functionalized inhibitors showed that these modifications were tolerated to some extent, but did not show any significant improvement in inhibitory potency compared to their lead structures. Additionally, triazole-based inhibitors demonstrated superior potency compared to their corresponding isoxazole-based inhibitors. Furthermore, a stereo-selective preference of these functional group modifications was observed with *S*-enantiomers showing higher potency compared to their corresponding *R*-enantiomers. Among the functional group modifications, the cyanomethyl derivatives emerged as the most potent inhibitors, highlighted by the *S*-enantiomer of the triazole-based cyanomethyl derivative **50** with an IC_50_ of 27 µM, which lies in a similar potency range to current established sirtuin 5 inhibitors. The exact binding mechanisms of the functionalized sirtuin 5 inhibitors could not yet be proven in this work due to the lack of co-crystal structures, but we are still actively pursuing this area of research. Nevertheless, our findings offer valuable insight into the SAR of balsalazide analogues as sirtuin 5 inhibitors and thus a viable foundation for the design and development of more potent balsalazide-based sirtuin 5 inhibitors in the future.

## Figures and Tables

**Table 1:** Method development in the borylation of alkyl halides **17** and **18** for the preparation of boronic acid pinacol ester **20**.

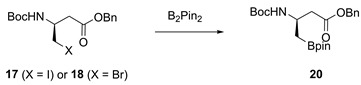							
Entry	X	Catalyst/Ligand	Base	Solvent	Temp.	Time	Yield
1	I	CuI, PPh_3_	LiOMe	DMF	rt	16 h	-
2	I	CuCl, Xantphos	KO*t*Bu	THF	rt	16 h	-
3	I	CuCl, Xantphos	KO*t*Bu	THF	50 °C	16 h	-
4	I	NiBr_2_·diglyme, *i*-Pr-PyBOX	KOEt	*i*-Pr_2_O/DMA	60 °C	16 h	-/33% (**46**) ^1^
5	I	Pd_2_(dba)_3_, *t*-Bu_2_MeP·HBF_4_	K_2_CO_3_	*t*-BuOH/H_2_O	60 °C	16 h	traces
6	I	CuI, PPh_3_, Bu_4_NI	LiO*t*Bu	DMF	60 °C	18 h	traces
7	Br	CuI, PPh_3_, Bu_4_NI	LiO*t*Bu	DMF	60 °C	18 h	10%
8	Br	CuI, PPh_3_	LiOMe	DMF	60 °C	16 h	-
9	Br	Pd_2_(dba)_3_, *t*-Bu_2_MeP·HBF_4_	K_2_CO_3_	*t*-BuOH/H_2_O	60 °C	16 h	16%
10a	Br	Pd_2_(dba)_3_, *t*-Bu_2_MeP·HBF_4_	K_2_CO_3_	*t*-BuOH/H_2_O	60 °C	6 h	42%
10b ^2^	Br	Pd_2_(dba)_3_, *t*-Bu_2_MeP·HBF_4_	K_2_CO_3_	*t*-BuOH/H_2_O	60 °C	6 h	60%

^1^ Dehalogenated side product **46**. 
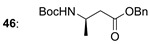
 ^2^ Entry 10b differs from entry 10a in the purification method.

**Table 2:** Inhibition of sirtuin 5 by **CG_209** and **CG_220** and the functionalized derivatives thereof. The IC_50_ values were calculated as a mean of three independent measurements with standard deviations shown. * = chiral center.

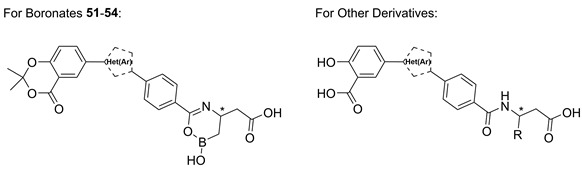				
Compound ID	Configuration	Het(Ar)	R	IC_50_ (hSirt5) [µM] ± SD
**CG_209**	-	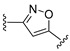	H	11 ± 1
**CG_220**	-	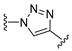	H	9 ± 2
**47**	*R*	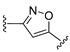	CH_2_CN	97 ± 22
**48**	*S*	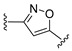	CH_2_CN	65 ± 15
**49**	*R*	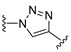	CH_2_CN	35 ± 6
**50**	*S*	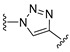	CH_2_CN	27 ± 9
**51**	*R*	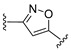	-	203 ± 50
**52**	*S*	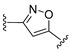	-	139 ± 18
**53**	*R*	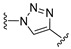	-	137 ± 63
**54**	*S*	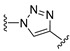	-	61 ± 9
**61**	*rac*	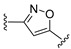	CH_2_CHO	82 ± 3
**64**	*rac*	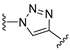	CH_2_CHO	60 ± 15

## Data Availability

The entirety of the experimental data and protocols are stored in the university’s electronic lab journal at LMU Munich.

## Supplementary Materials

The following supporting information can be downloaded at: https://www.mdpi.com/article/10.3390/molecules30183821/s1: S1. ^1^H and ^13^C NMR spectra of synthesized compounds; S2. HPLC chromatograms of tested compounds; S3. Crystal structure data of compound **45** (including relevant references no. [47,48,49,50]).

## Abbreviations

The following abbreviations are used in this manuscript:

NAD^+^	Nicotinamide adenine dinucleotide
IC_50_	Half maximal inhibitory concentration
CDI	1,1′-Carbonyldiimidazole
PIFA	(Bis(trifluoroacetoxy)iodo)benzene
CuAAC	Copper-catalyzed azide-alkyne cycoladdition
LED	Light-emitting diode
FCC	Flash column chromatography
PTLC	Preparative thin-layer chromatography
