# Supplementary material for: Balsalazide-Derived Heterotriaryls as Sirtuin 5 Inhibitors: A Case Study of a Reversible Covalent Inhibition Strategy
Source: Molecules. 2025 Sep 20;30(18):3821. doi: 10.3390/molecules30183821 (PMC12472749; doi:10.3390/molecules30183821)
Supplement: Supplementary file 1 [file molecules-30-03821-s001.zip › molecules-3861391-supplementary.pdf]

Supporting Information

Balsalazide-Derived Heterotriaryls as Sirtuin 5 Inhibitors: A Case Study of a Reversible Covalent Inhibition Strategy

Ricky Wirawan, Simon A. Huber, Thomas Wein and Franz Bracher *

Department of Pharmacy—Center for Drug Research, Ludwig-Maximilians University, Butenandtstr. 5-13, 81377 Munich, Germany; ricky.wirawan@cup.uni-muenchen.de (R.W.); simon.huber@campus.lmu.de (S.A.H.); thomas.wein@cup.uni-muenchen.de (T.W.)
* Correspondence: franz.bracher@cup.uni-muenchen.de; Tel.: +49-89-218077301

Contents:

1. ^{1}H and ^{13}C NMR spectra of synthesized compounds.. 2

2. HPLC chromatograms of tested compounds 42

3. Crystal structure data of oxazoline **45** 52

S1. ¹H and ¹³C NMR spectra of synthesized compounds

¹H and ¹³C NMR spectra of benzyl (*R*)-3-((*tert*-butoxycarbonyl)amino)-4-(4,4,5,5-tetramethyl-1,3,2-dioxaborolan-2-yl)butanoate (**20**).

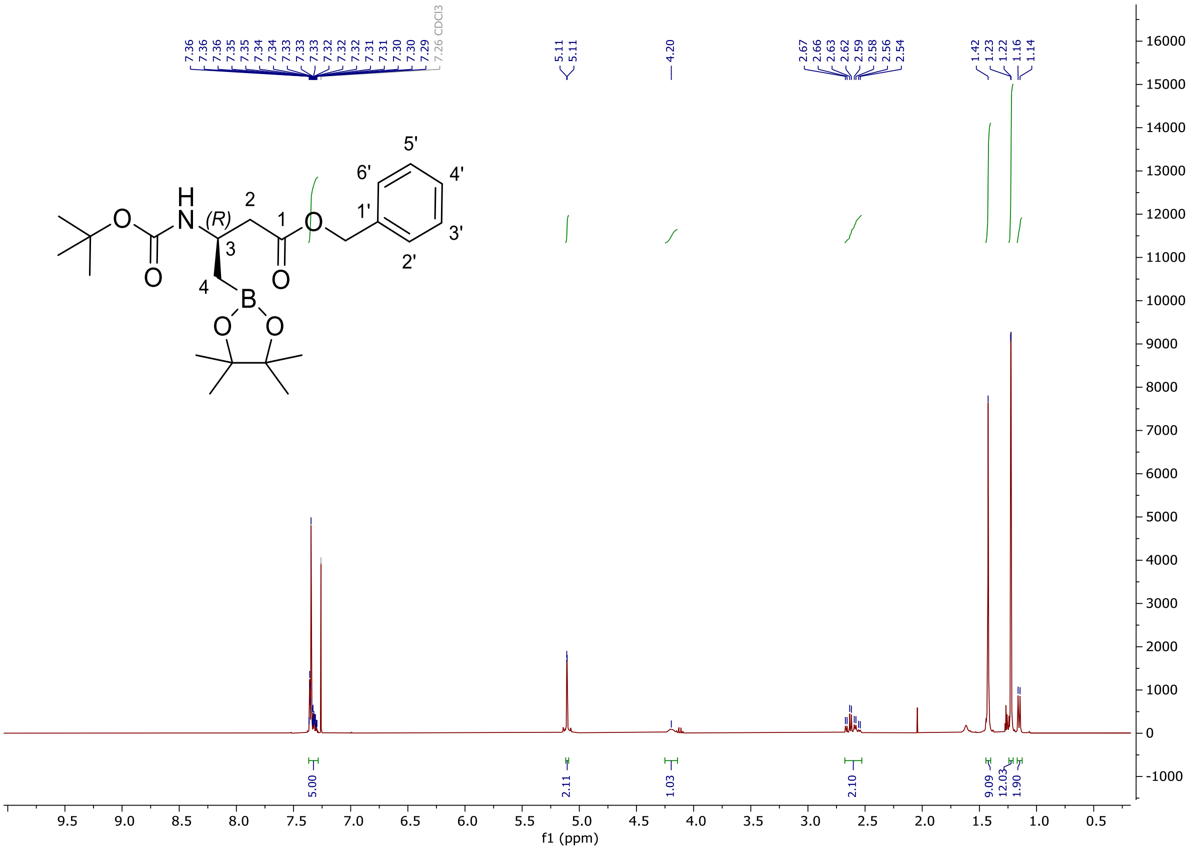

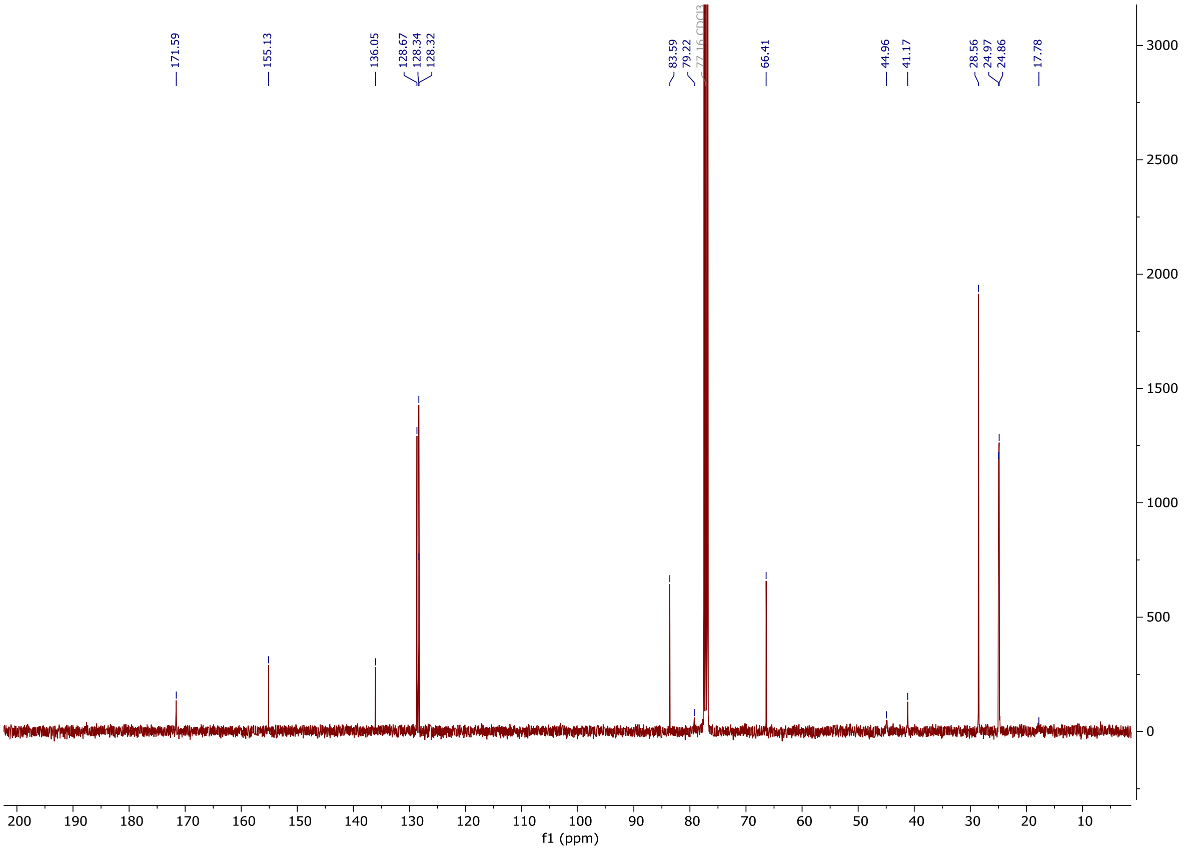

^{1}H and ^{13}C NMR spectra of benzyl (*S*)-3-((*tert*-butoxycarbonyl)amino)-4-(4,4,5,5-tetramethyl-1,3,2-dioxaborolan-2-yl)butanoate (**21**).

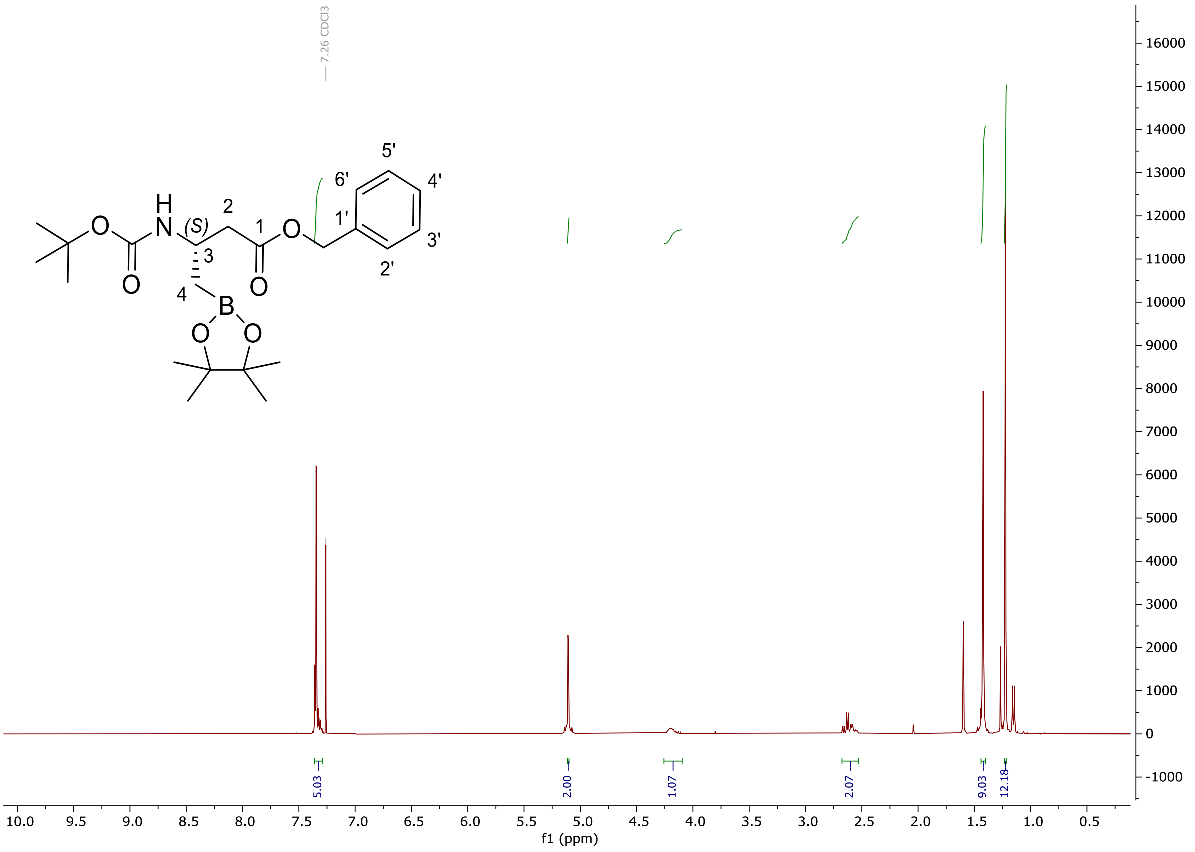

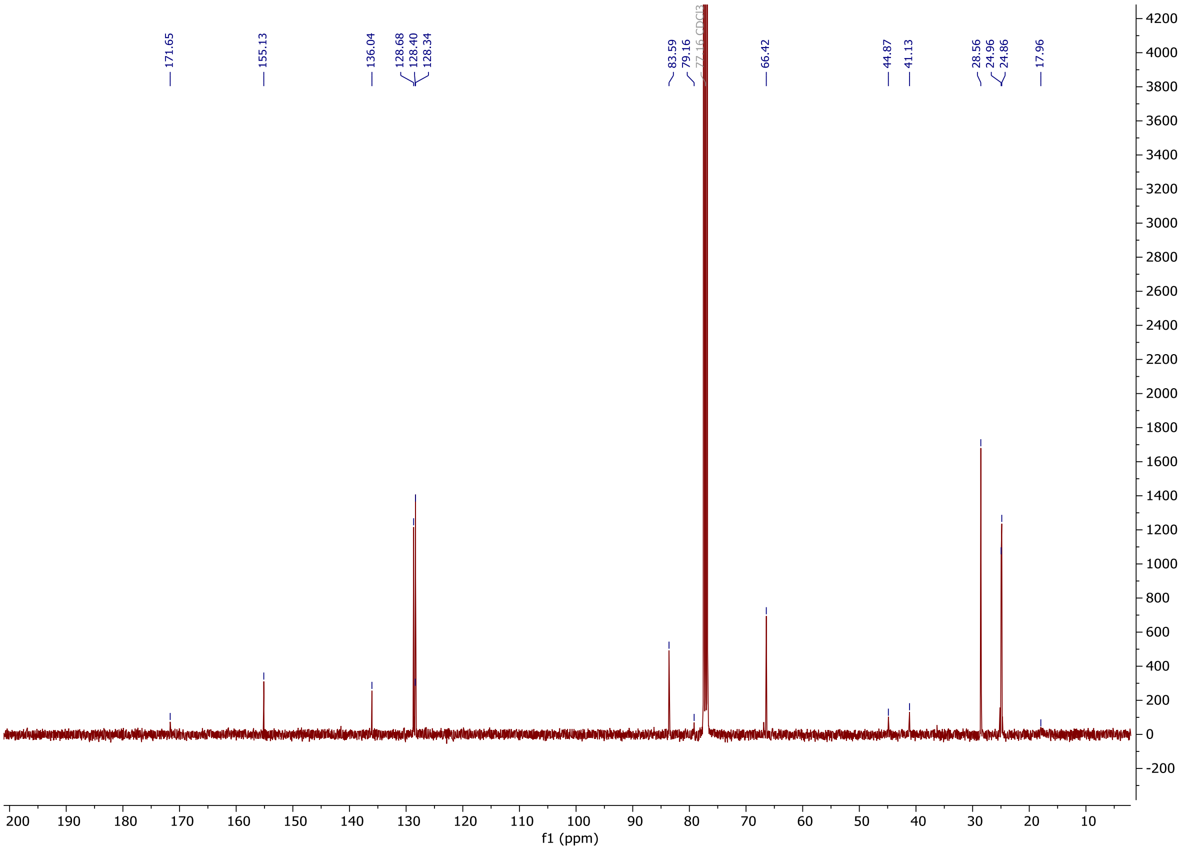

^{1}H and ^{13}C NMR spectra of (*R*)-4-(benzyloxy)-4-oxo-1-(4,4,5,5-tetramethyl-1,3,2-dioxaborolan-2-yl)butan-2-aminium (**24**).

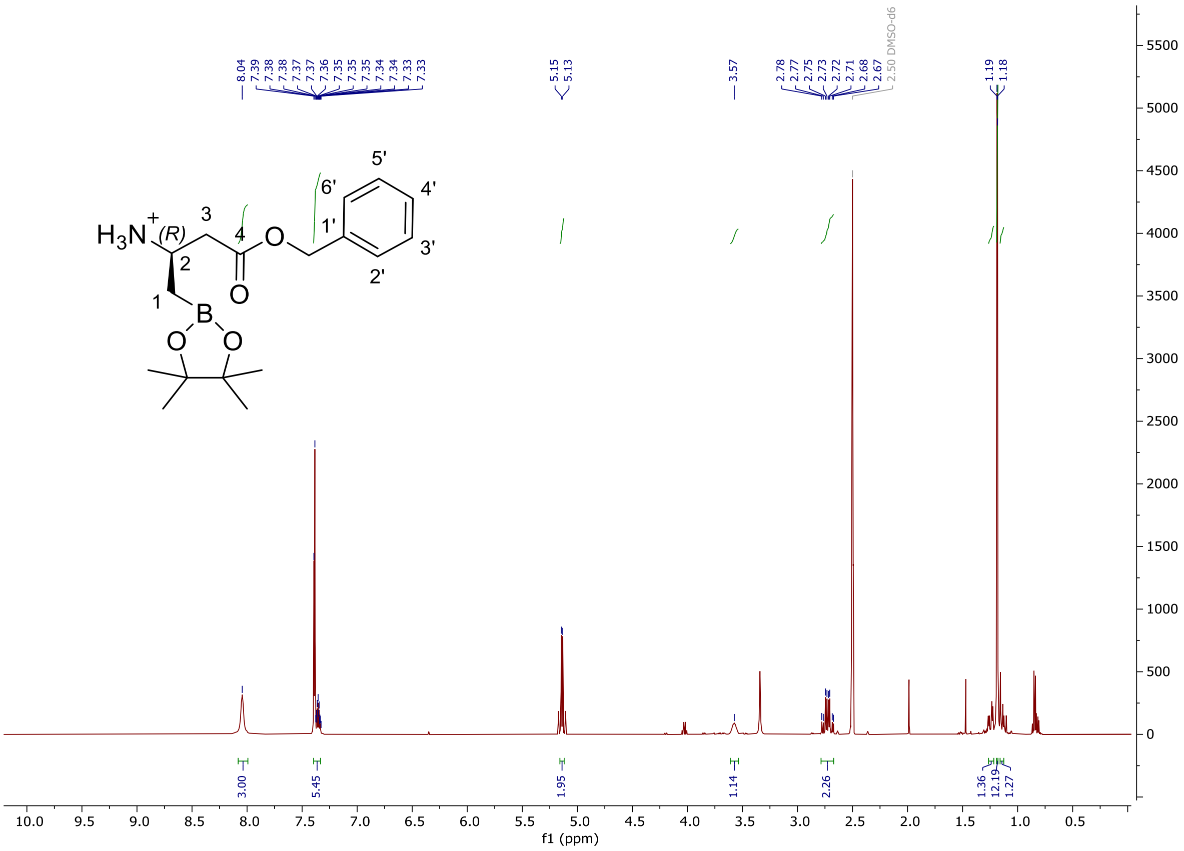

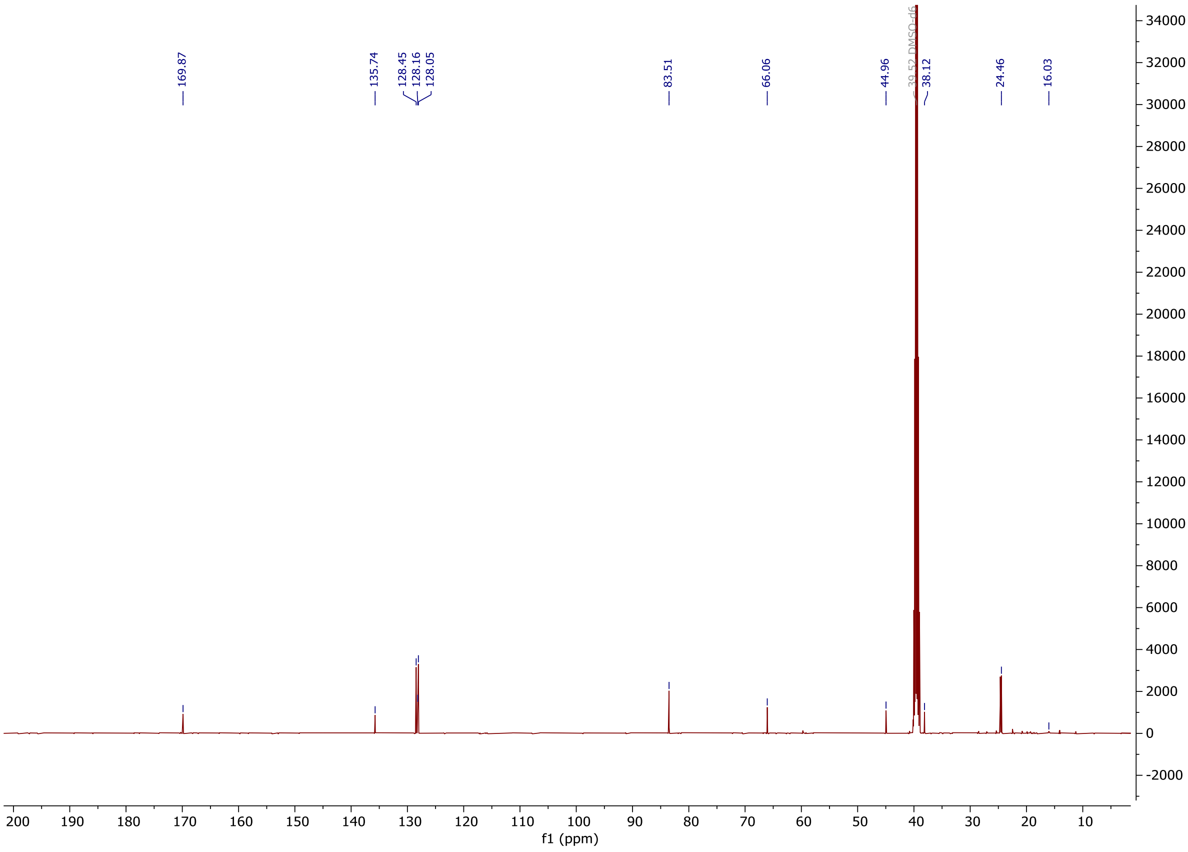

^{1}H and ^{13}C NMR spectra of (*S*)-4-(benzyloxy)-4-oxo-1-(4,4,5,5-tetramethyl-1,3,2-dioxaborolan-2-yl)butan-2-aminium (**25**).

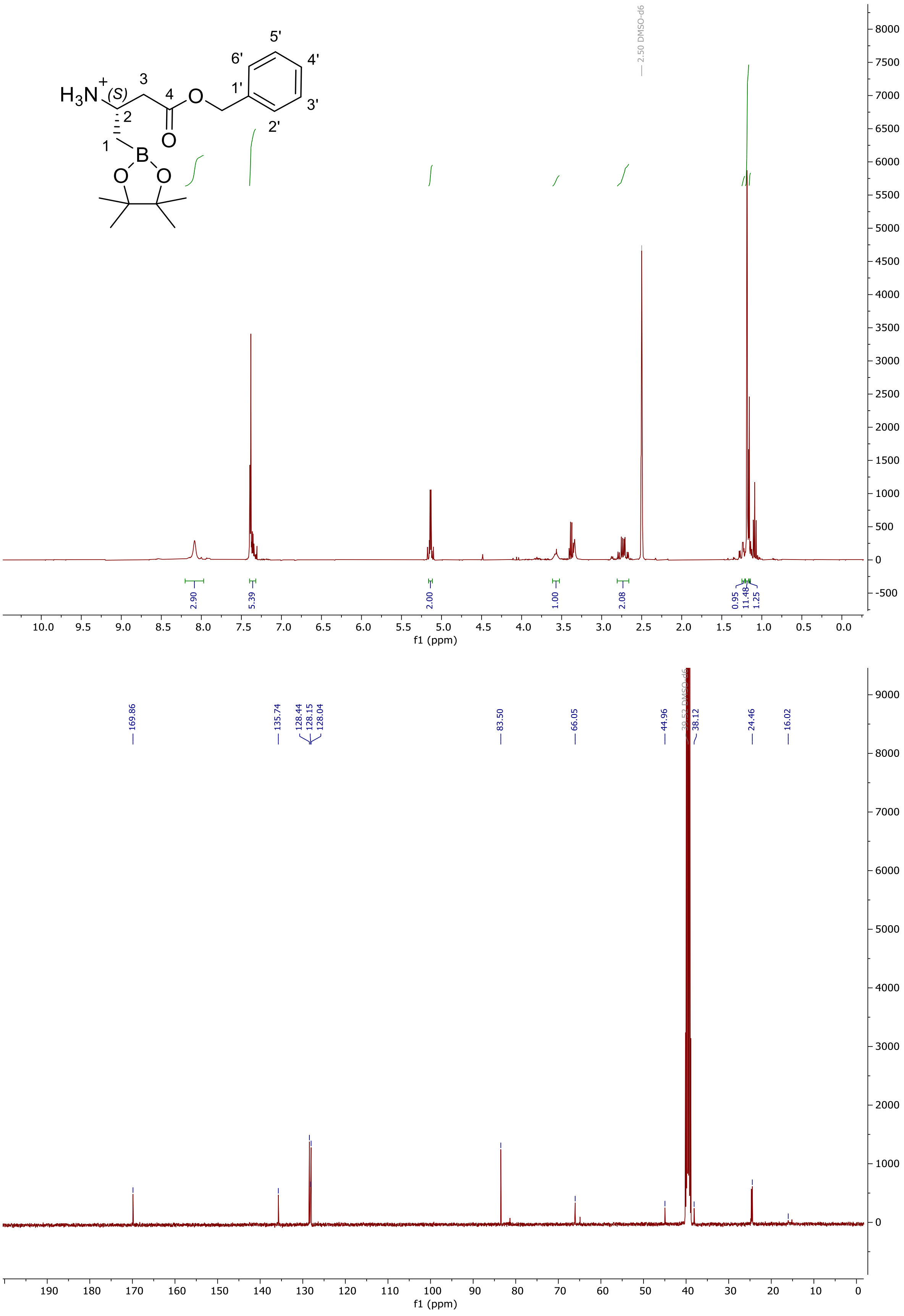

¹H and ¹³C NMR spectra of (*R*)-4-(benzyloxy)-1-cyano-4-oxobutan-2-aminium (**26**).

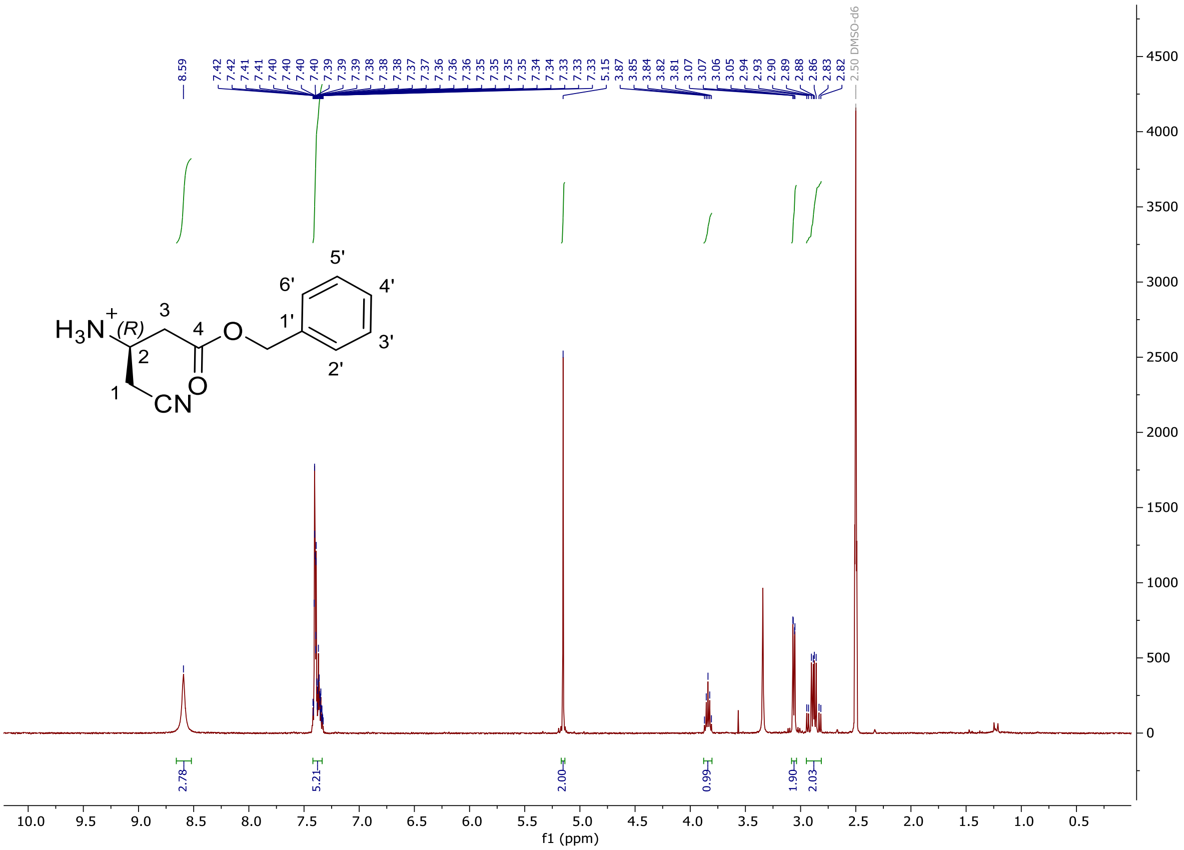

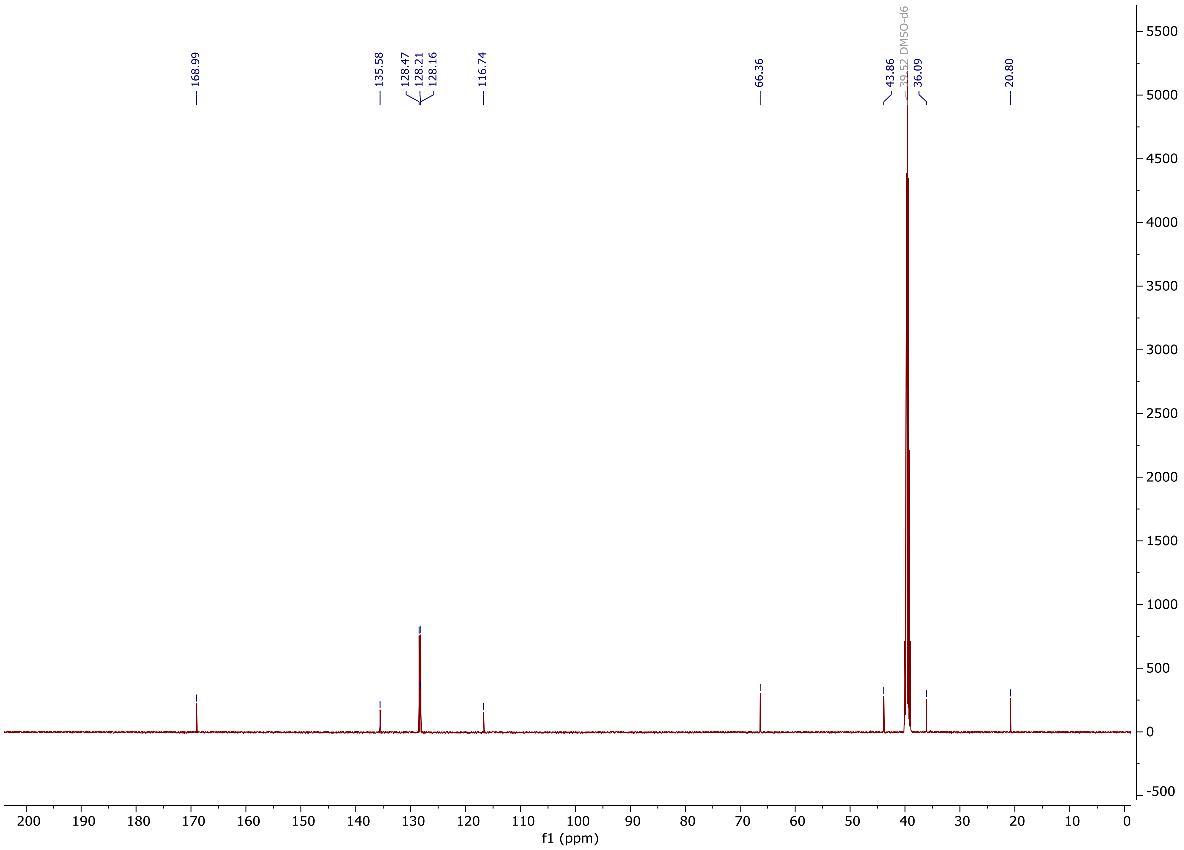

^{1}H and ^{13}C NMR spectra of (*S*)-4-(benzyloxy)-1-cyano-4-oxobutan-2-aminium (**27**).

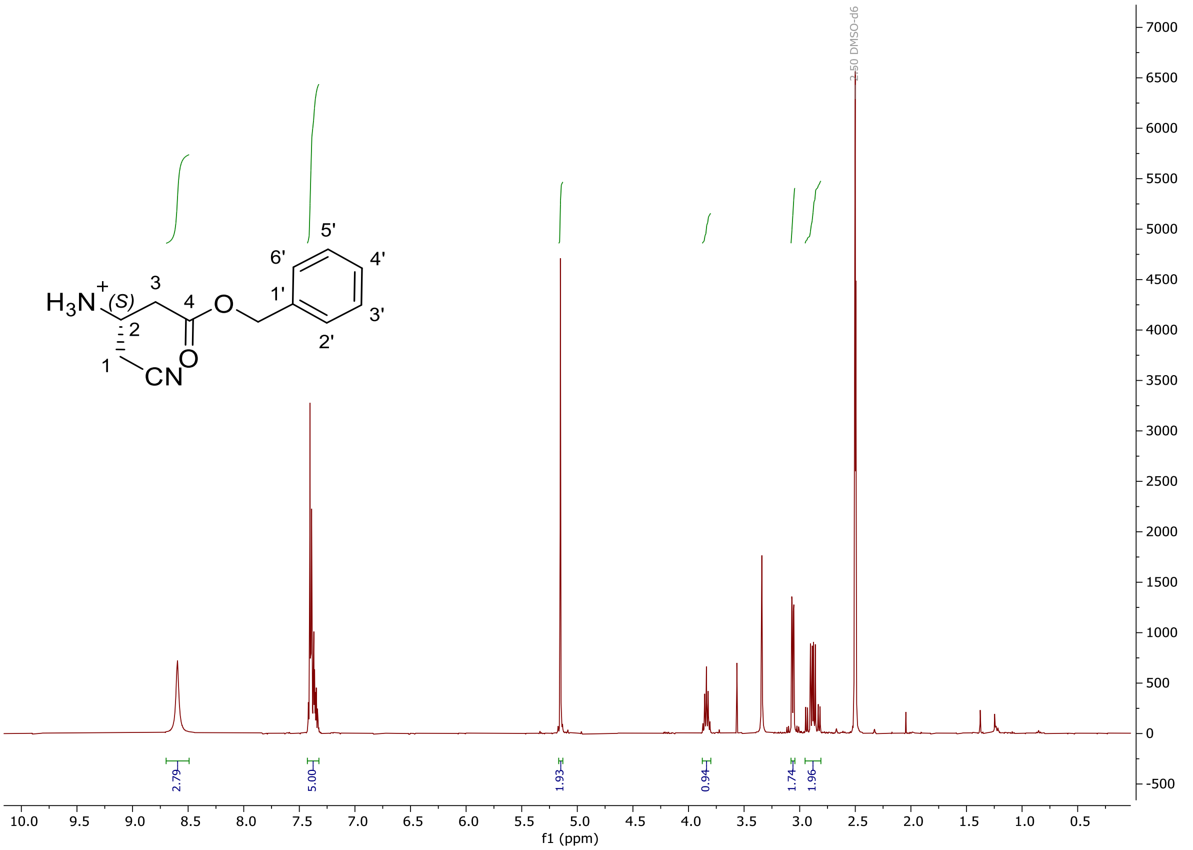

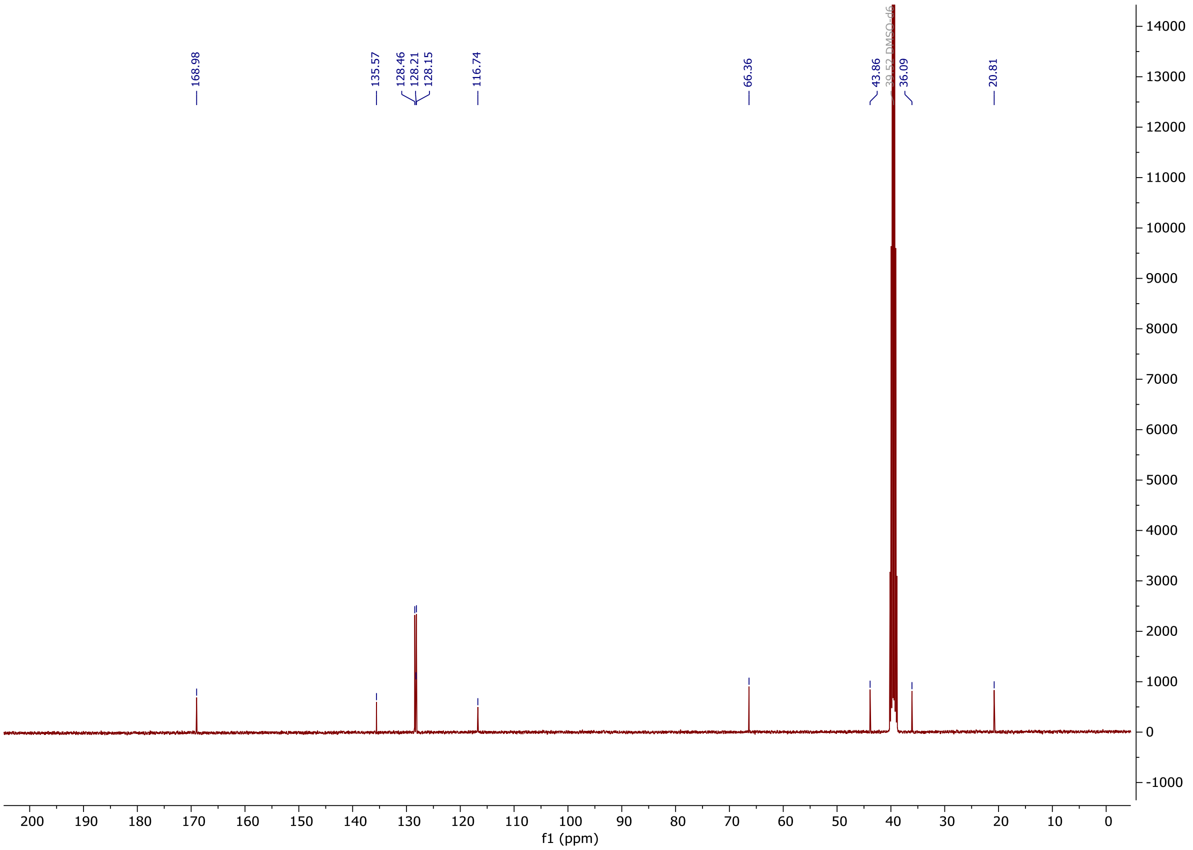

^{1}H and ^{13}C NMR spectra of benzyl (*R*)-3-(4-ethynylbenzamido)-4-(4,4,5,5-tetramethyl-1,3,2-dioxaborolan-2-yl)butanoate (**28**).

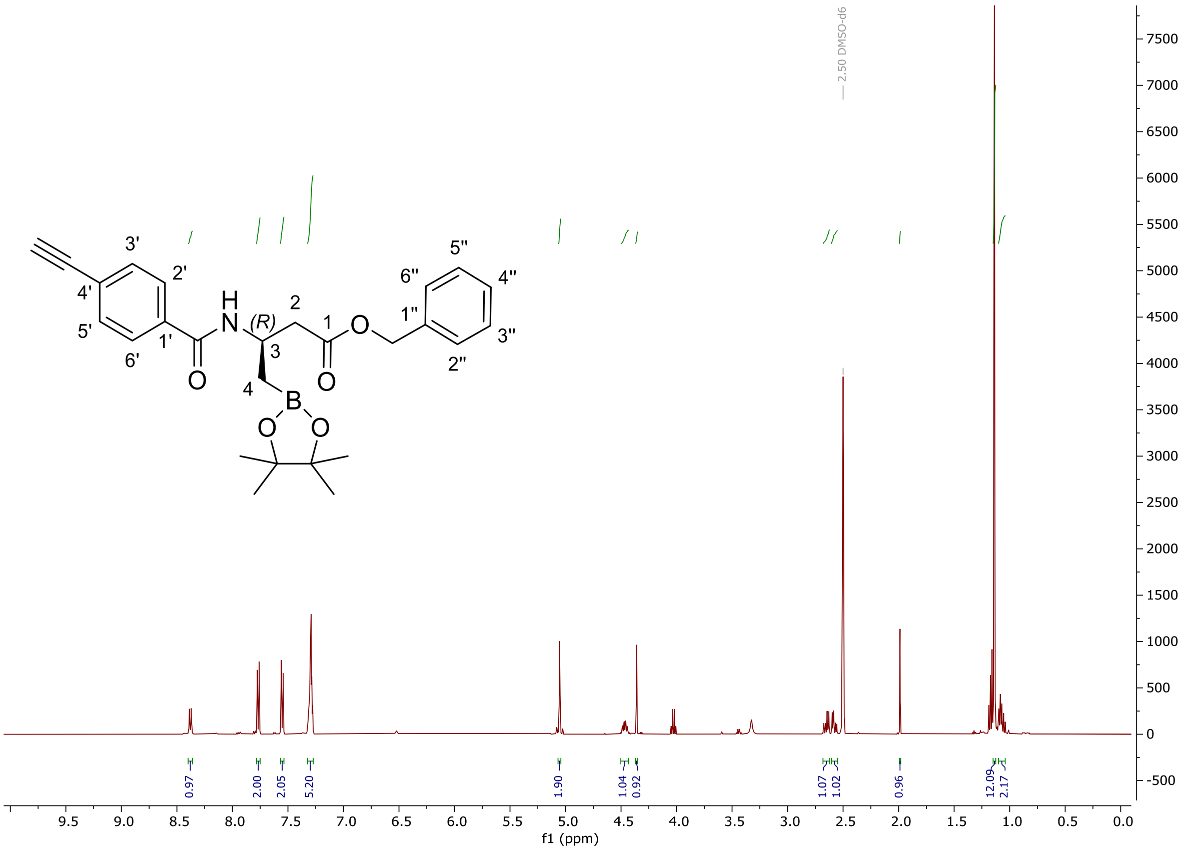

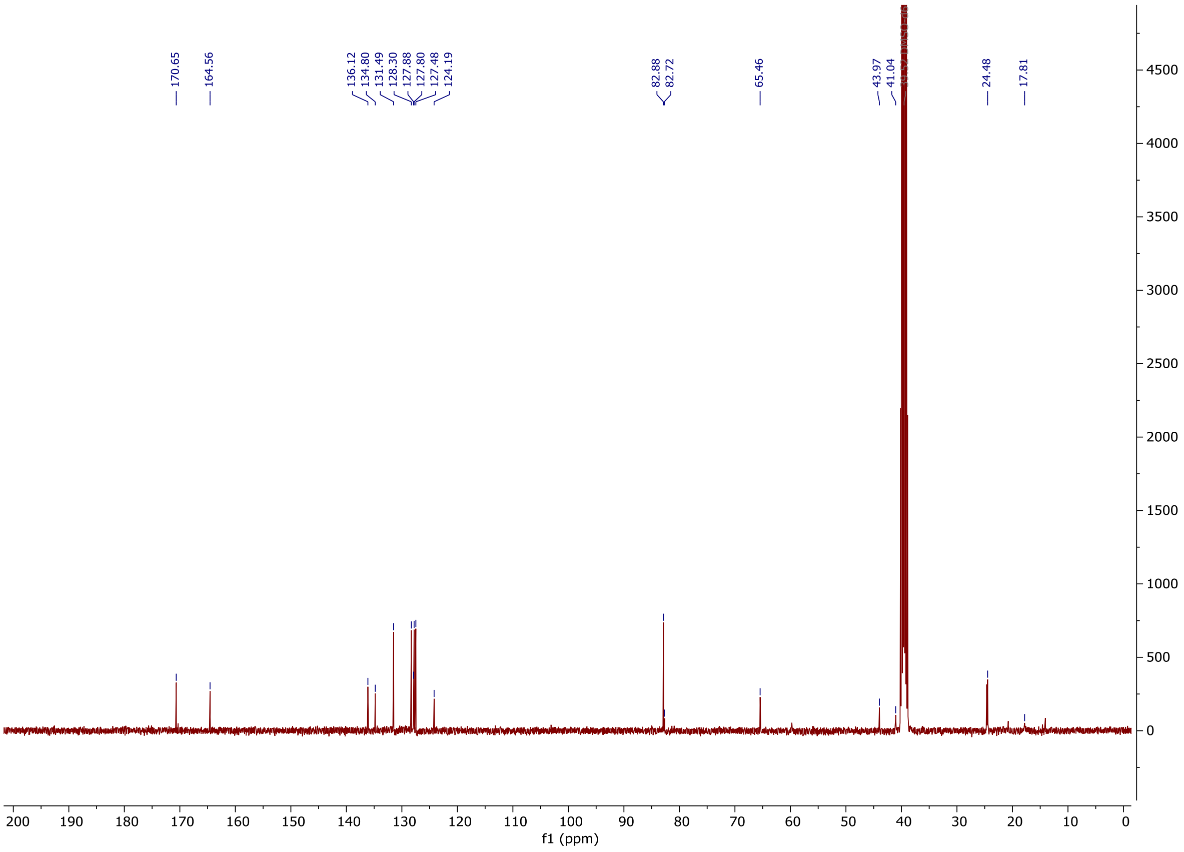

^{1}H and ^{13}C NMR spectra of benzyl (*S*)-3-(4-ethynylbenzamido)-4-(4,4,5,5-tetramethyl-1,3,2-dioxaborolan-2-yl)butanoate (**29**).

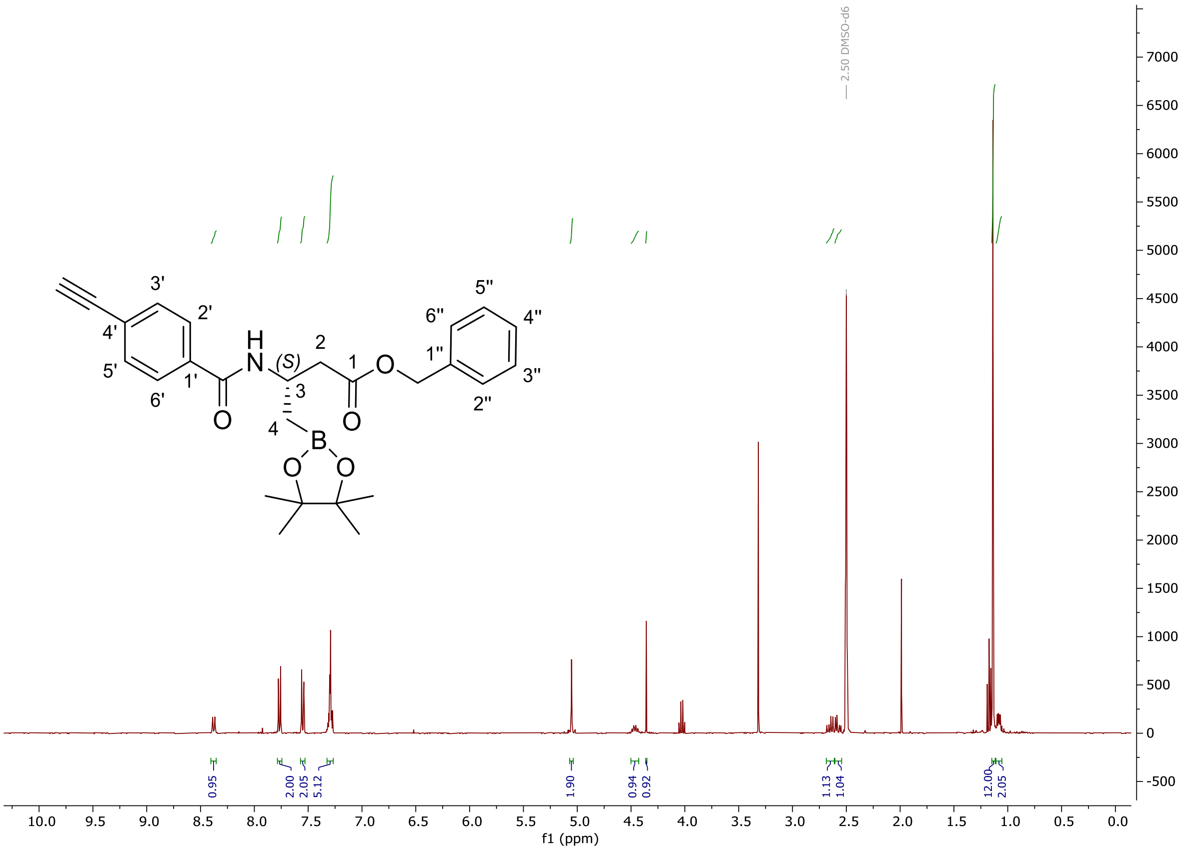

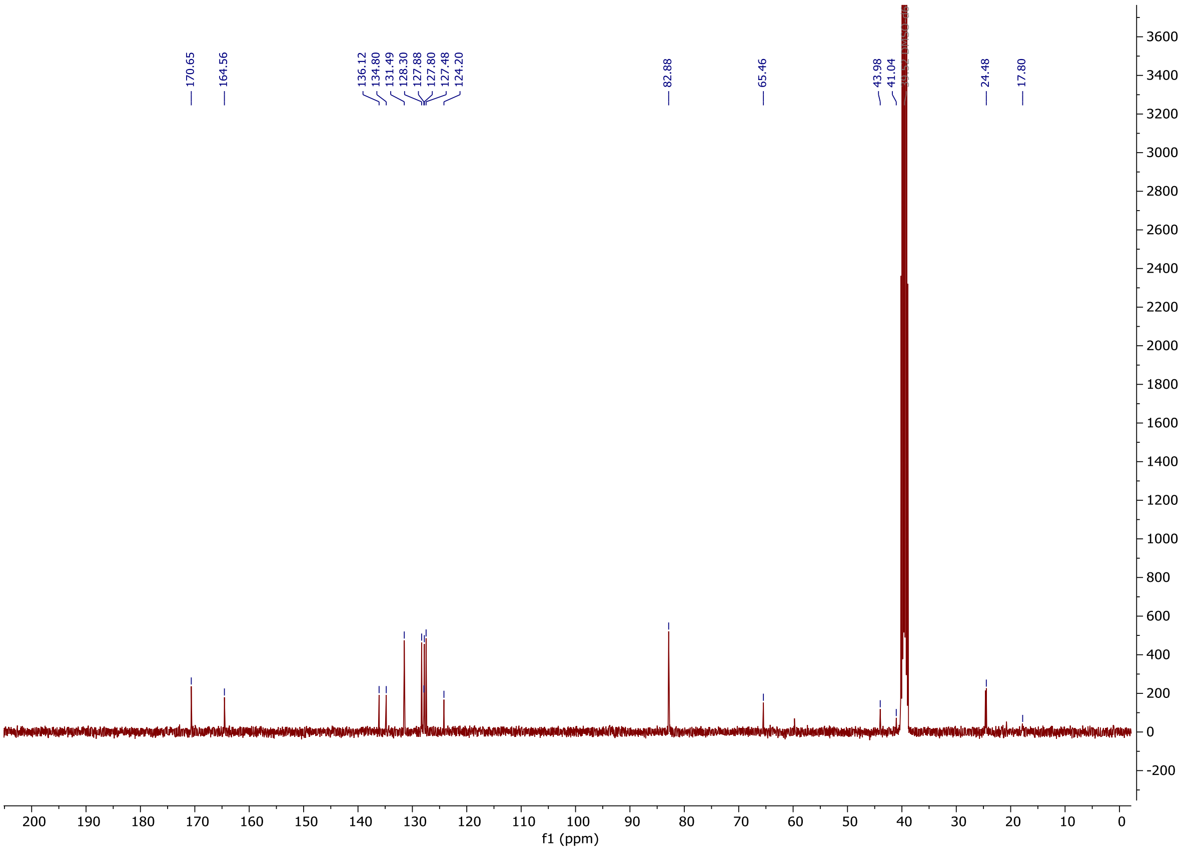

^{1}H and ^{13}C NMR spectra of benzyl (*R*)-4-cyano-3-(4-ethynylbenzamido)butanoate (**30**).

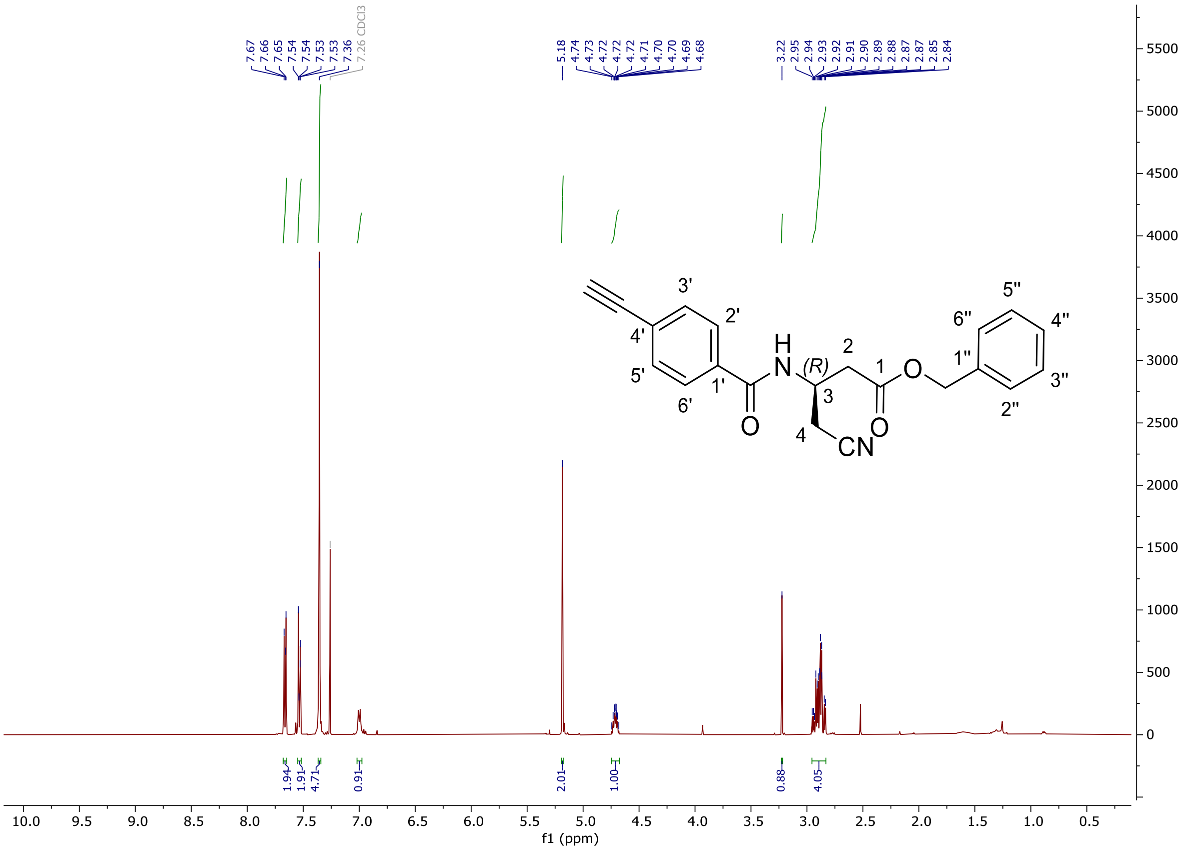

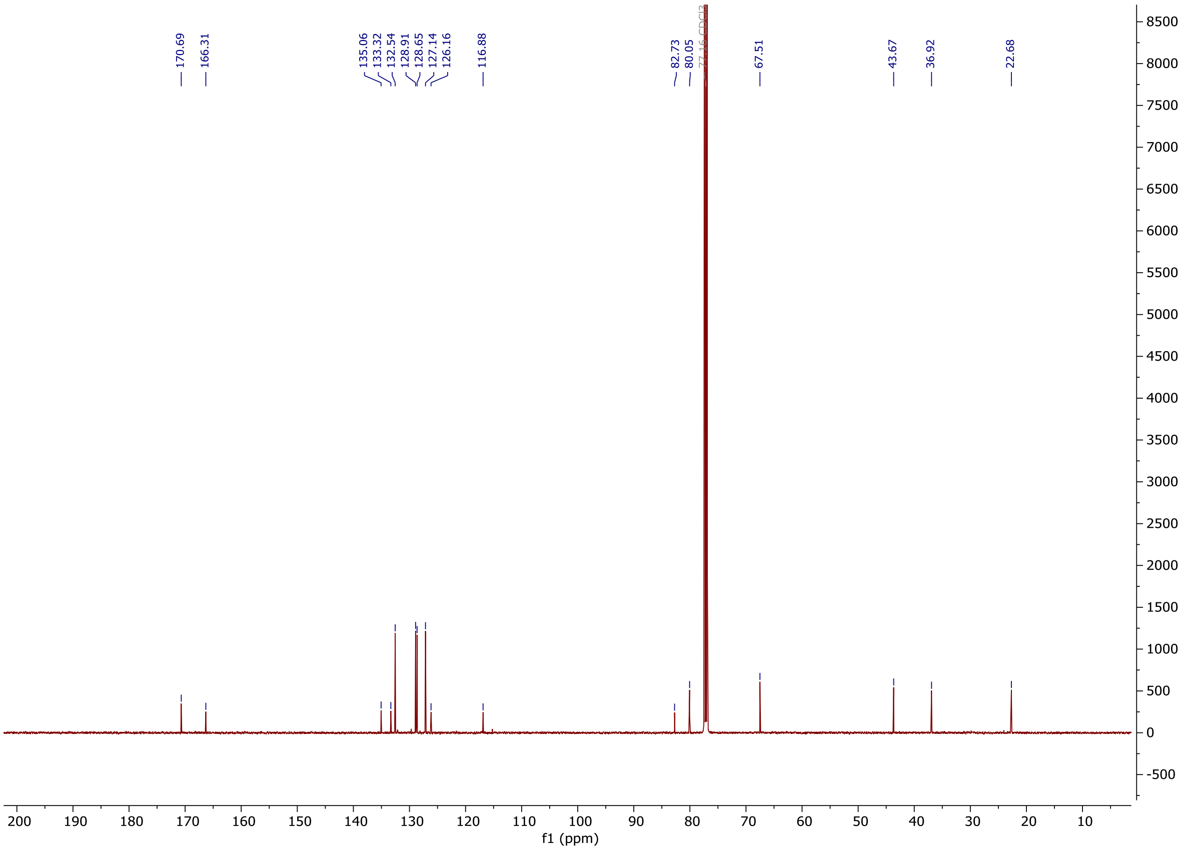

¹H and ¹³C NMR spectra of benzyl (*S*)-4-cyano-3-(4-ethynylbenzamido)butanoate (**31**).

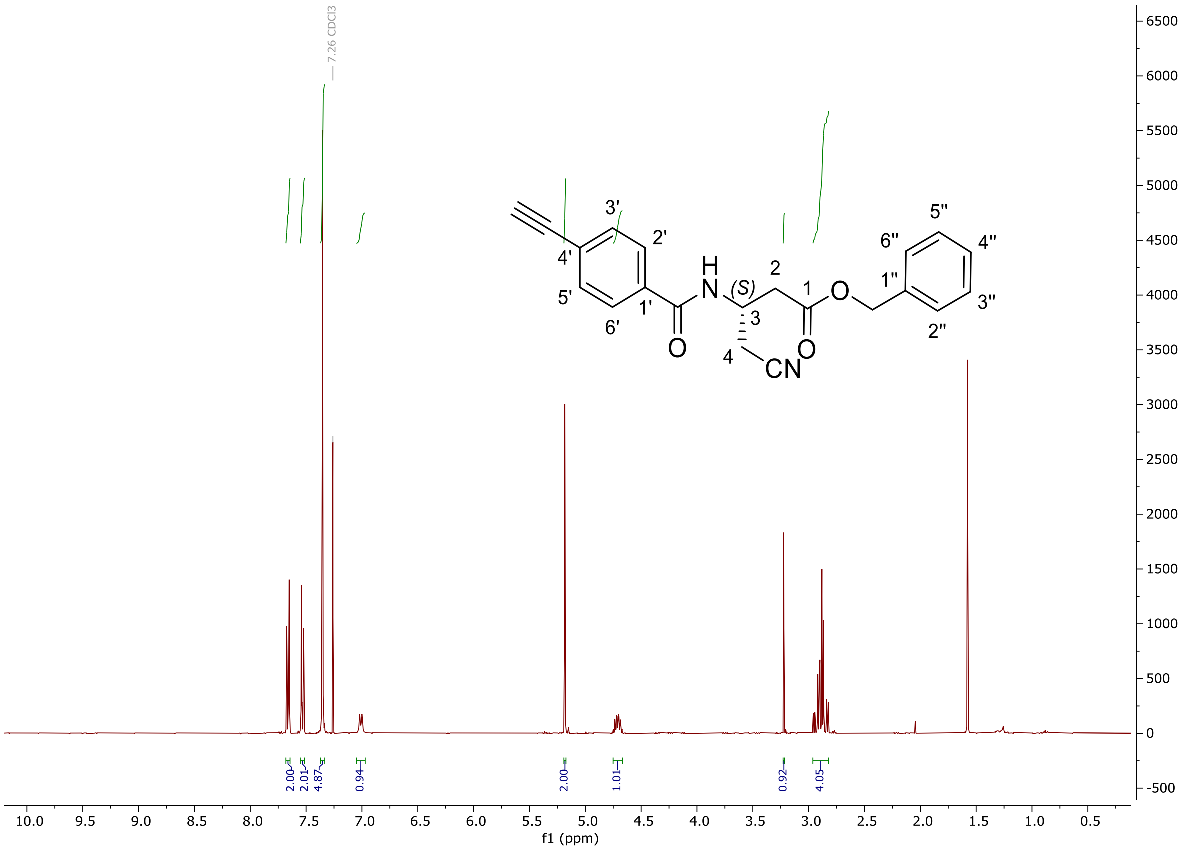

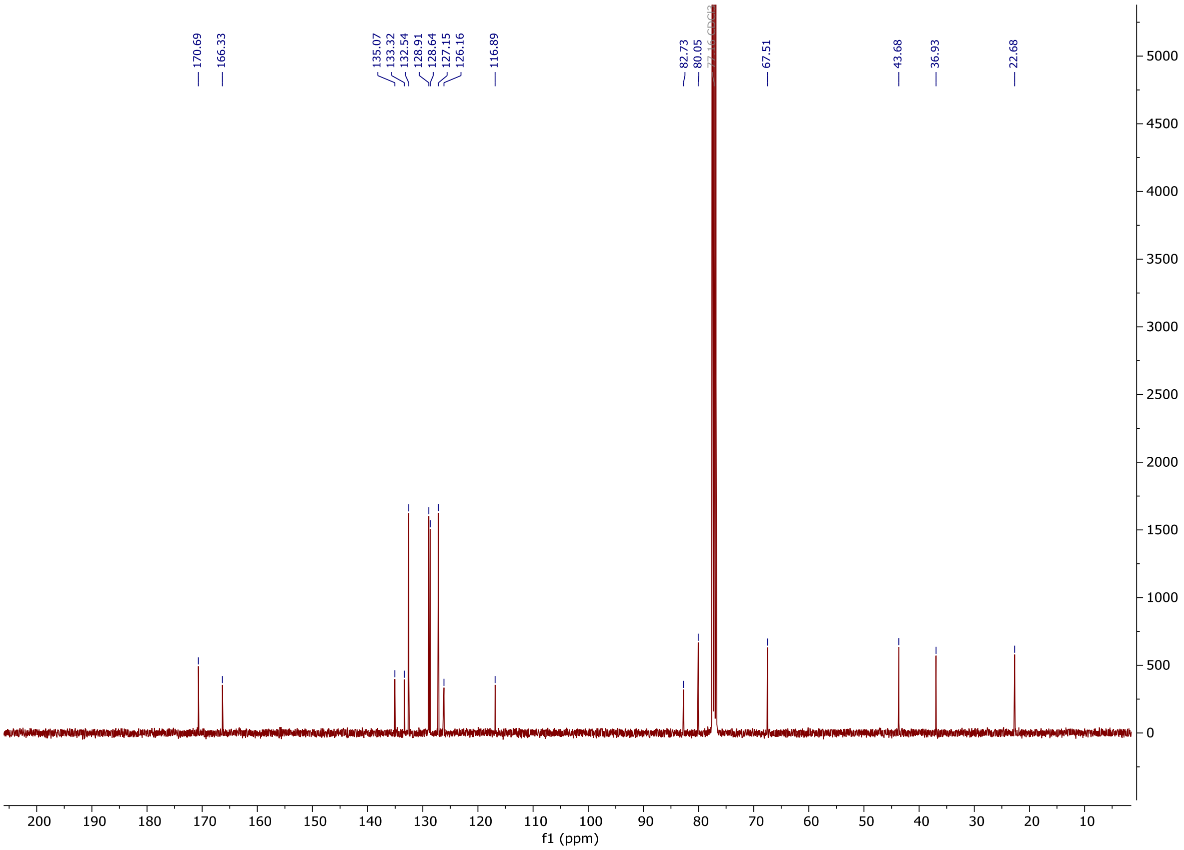

^{1}H and ^{13}C NMR spectra of methyl (*R*)-3-(4-(3-(2,2-dimethyl-4-oxo-4*H*-benzo[*d*][1,3]dioxin-6-yl)isoxazol-5-yl)benzamido)-4-(4,4,5,5-tetramethyl-1,3,2-dioxaborolan-2-yl)butanoate (**32**).

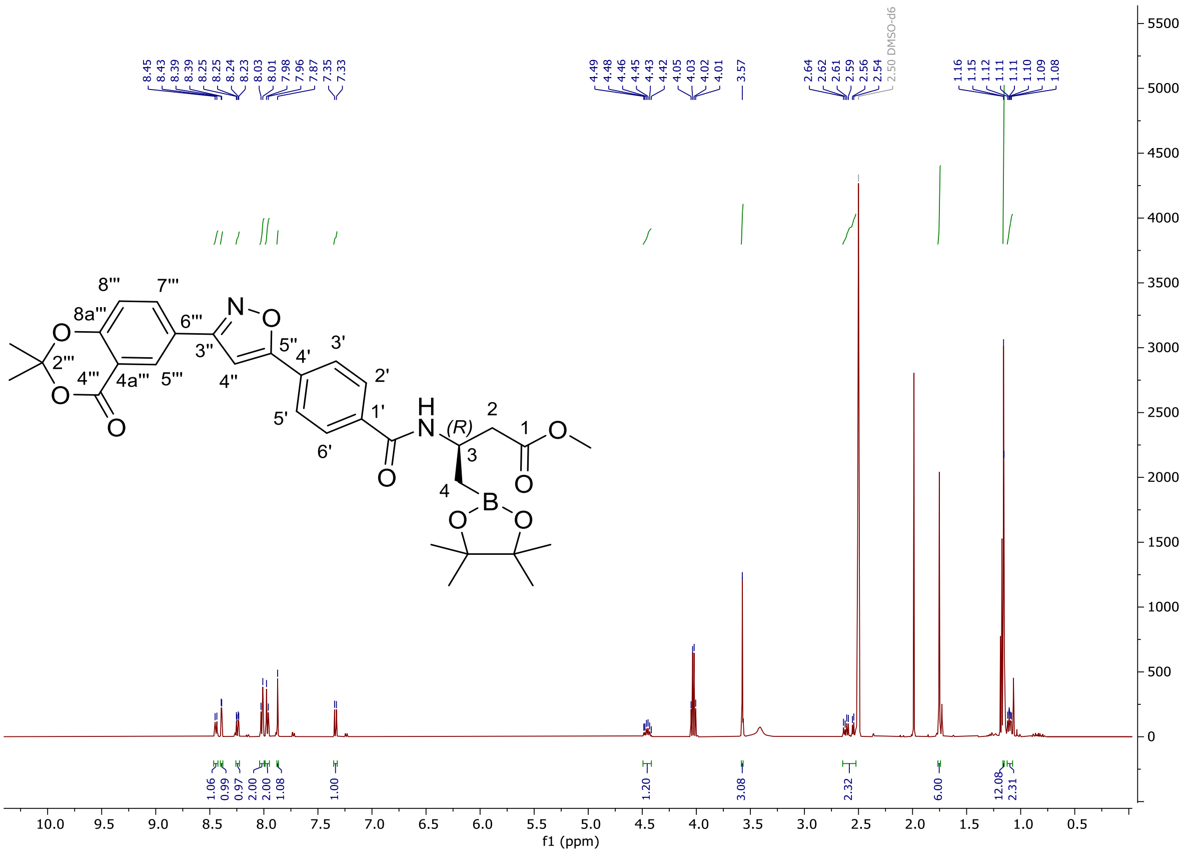

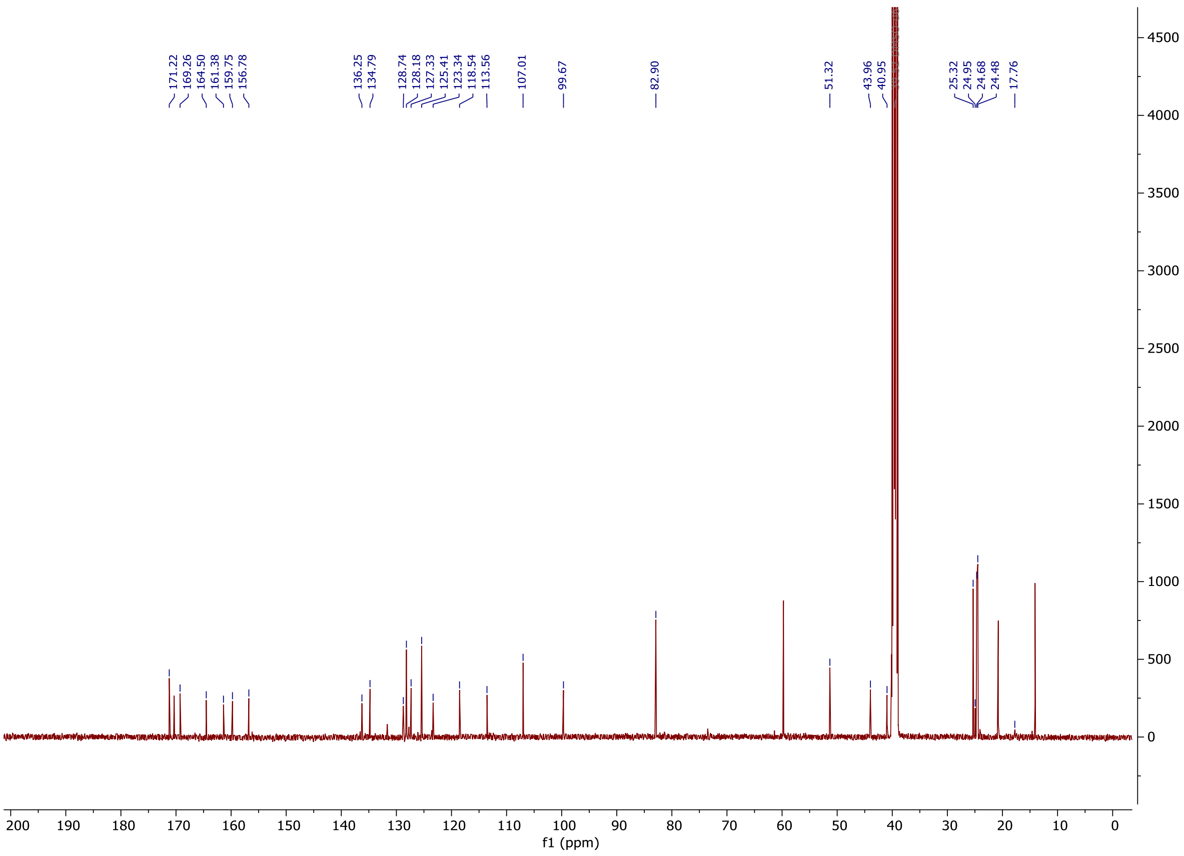

^{1}H and ^{13}C NMR spectra of methyl (*S*)-3-(4-(3-(2,2-dimethyl-4-oxo-4*H*-benzo[*d*][1,3]dioxin-6-yl)isoxazol-5-yl)benzamido)-4-(4,4,5,5-tetramethyl-1,3,2-dioxaborolan-2-yl)butanoate (**33**).

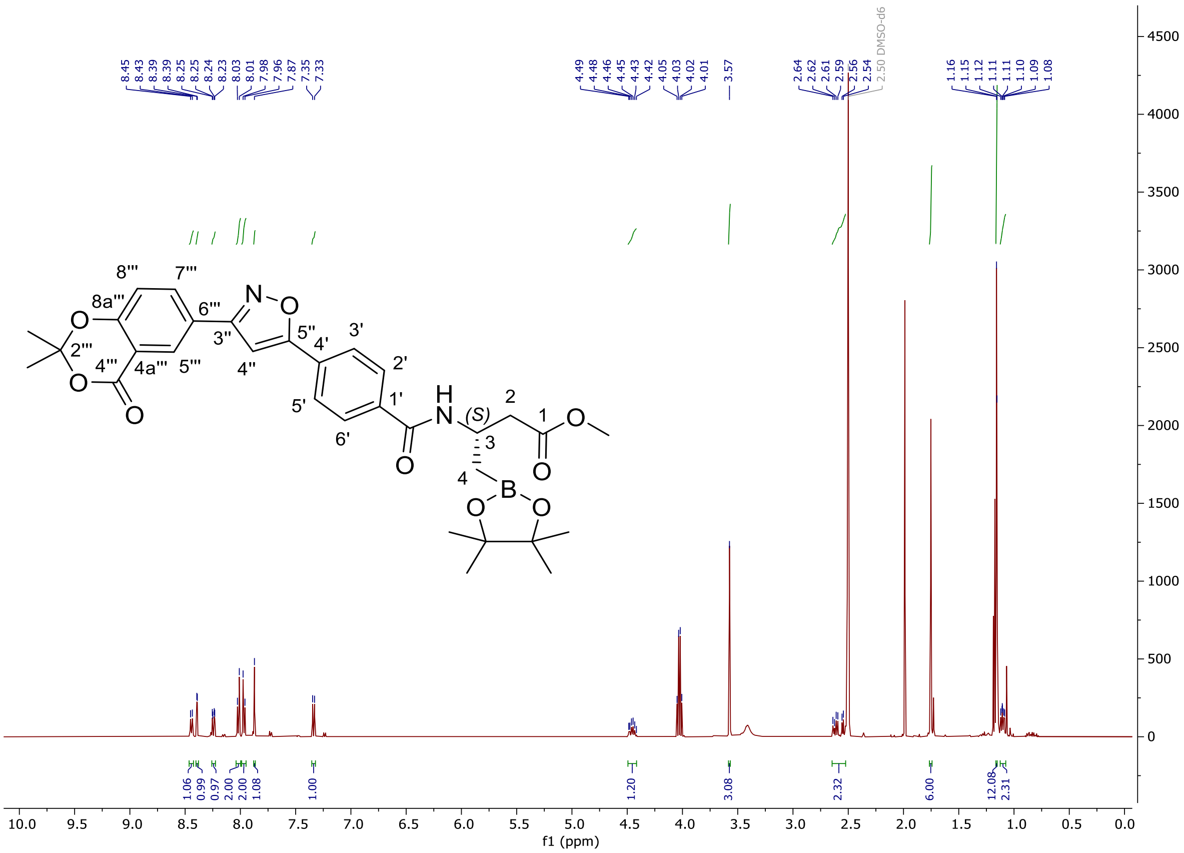

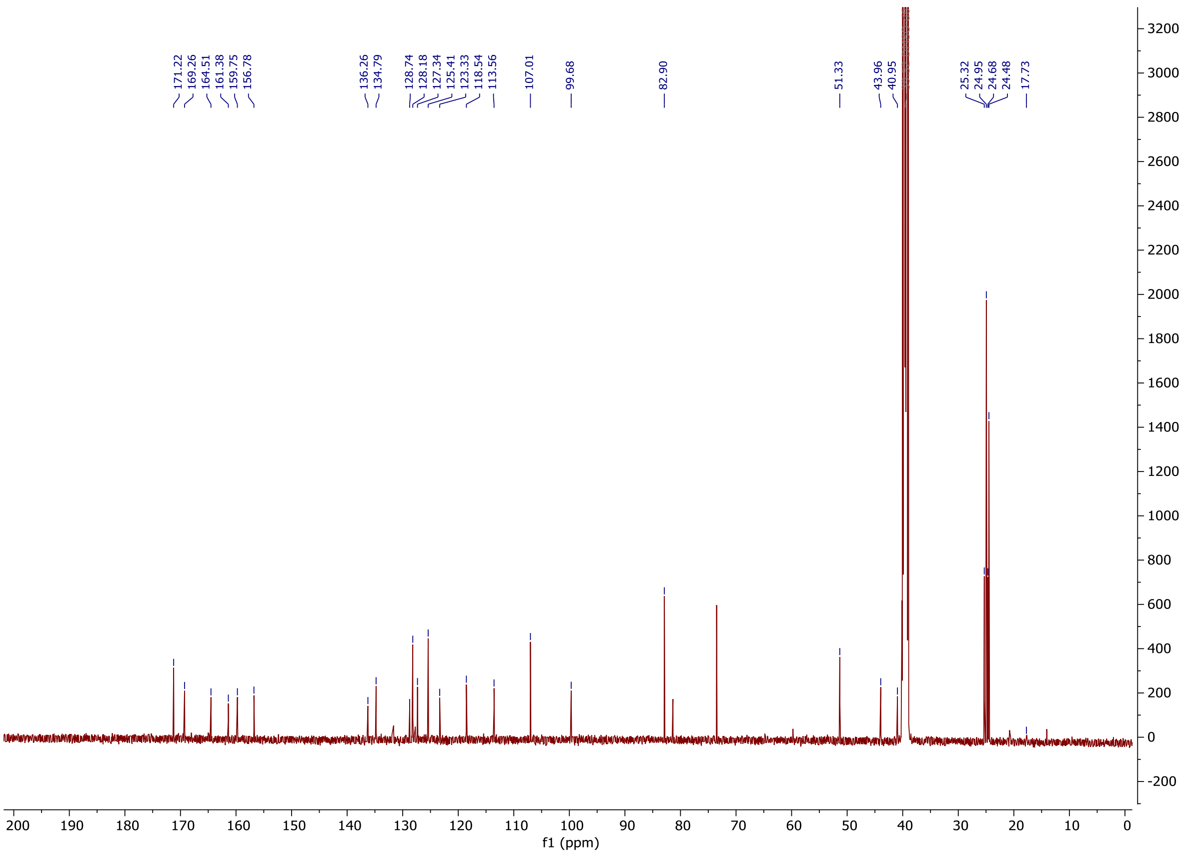

^{1}H and ^{13}C NMR spectra of benzyl (*R*)-4-cyano-3-(4-(3-(2,2-dimethyl-4-oxo-4*H*-benzo[*d*][1,3]dioxin-6-yl)isoxazol-5-yl)benzamido)butanoate (**34**).

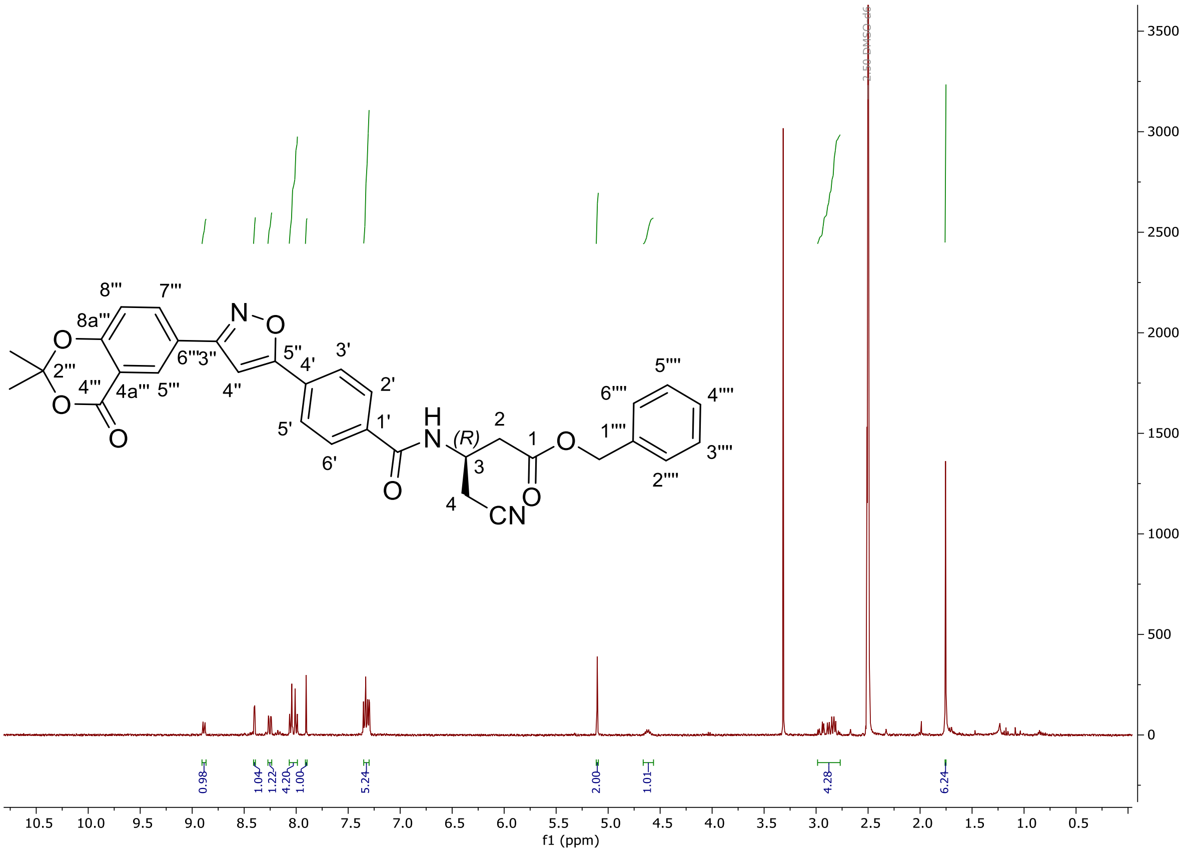

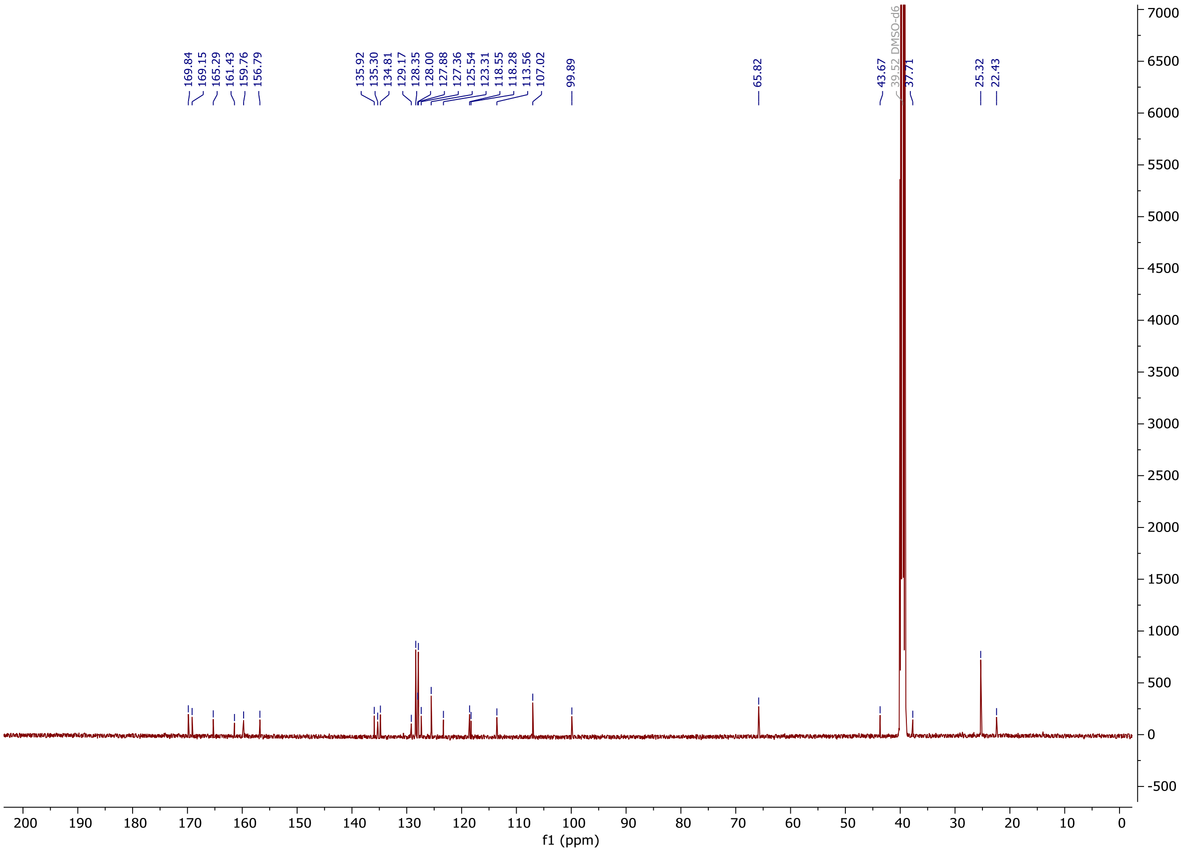

^{1}H and ^{13}C NMR spectra of benzyl (*S*)-4-cyano-3-(4-(3-(2,2-dimethyl-4-oxo-4*H*-benzo[*d*][1,3]dioxin-6-yl)isoxazol-5-yl)benzamido)butanoate (**35**).

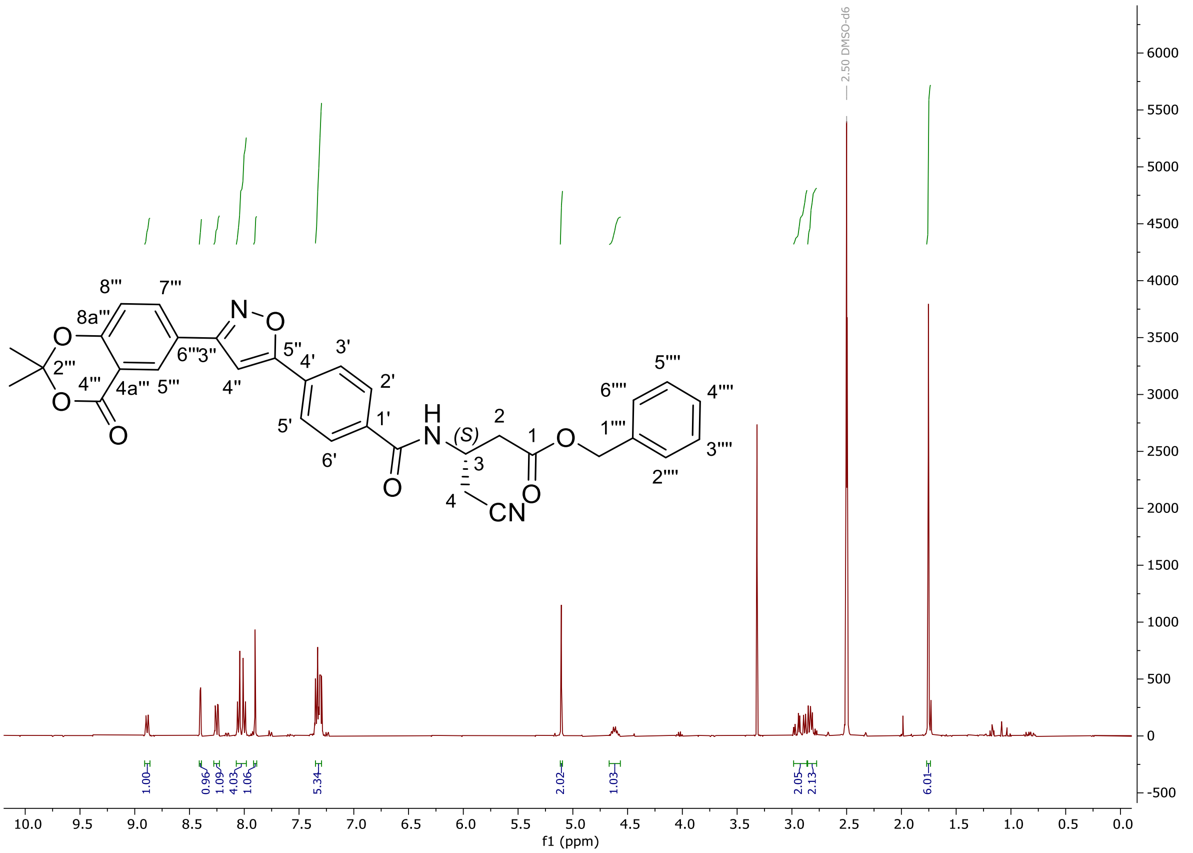

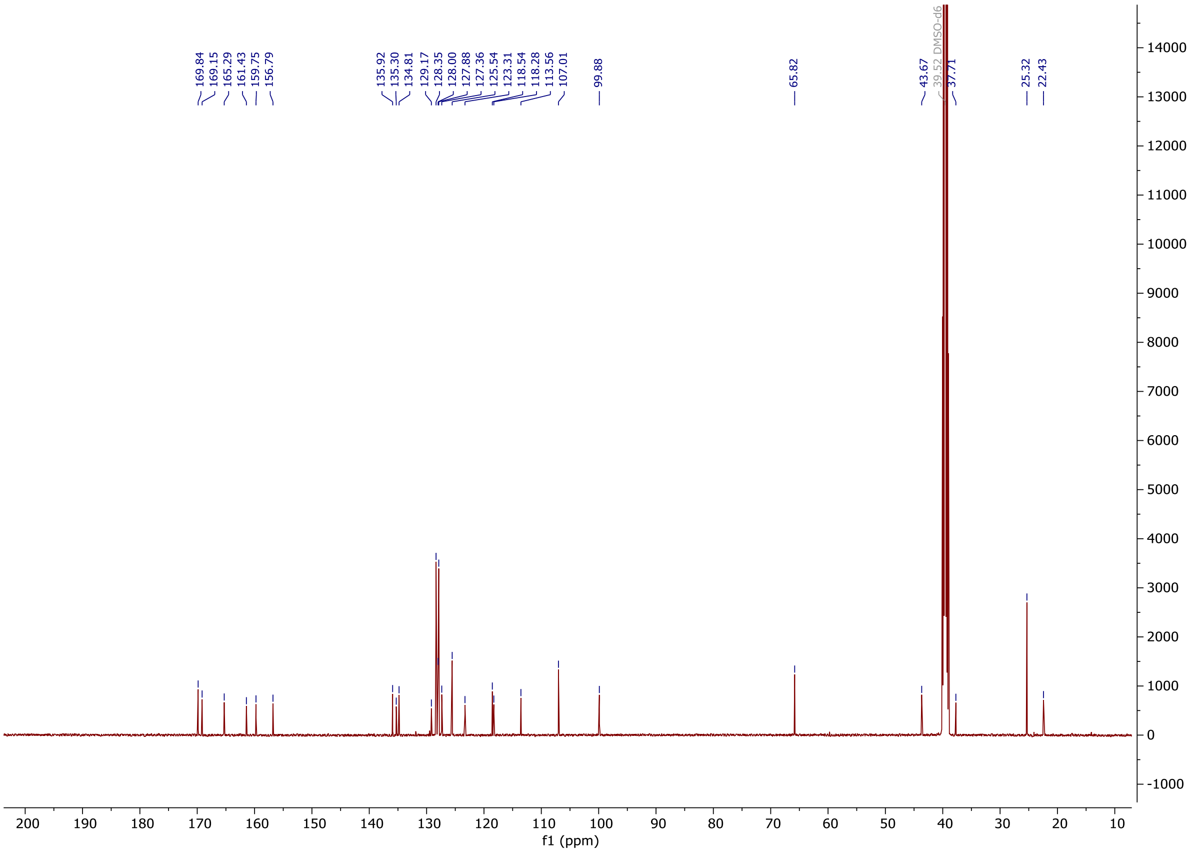

^{1}H and ^{13}C NMR spectra of (*R*)-(2-(4-(3-(2,2-dimethyl-4-oxo-4*H*-benzo[*d*][1,3]dioxin-6-yl)isoxazol-5-yl)benzamido)-4-methoxy-4-oxobutyl)boronic acid (**36**).

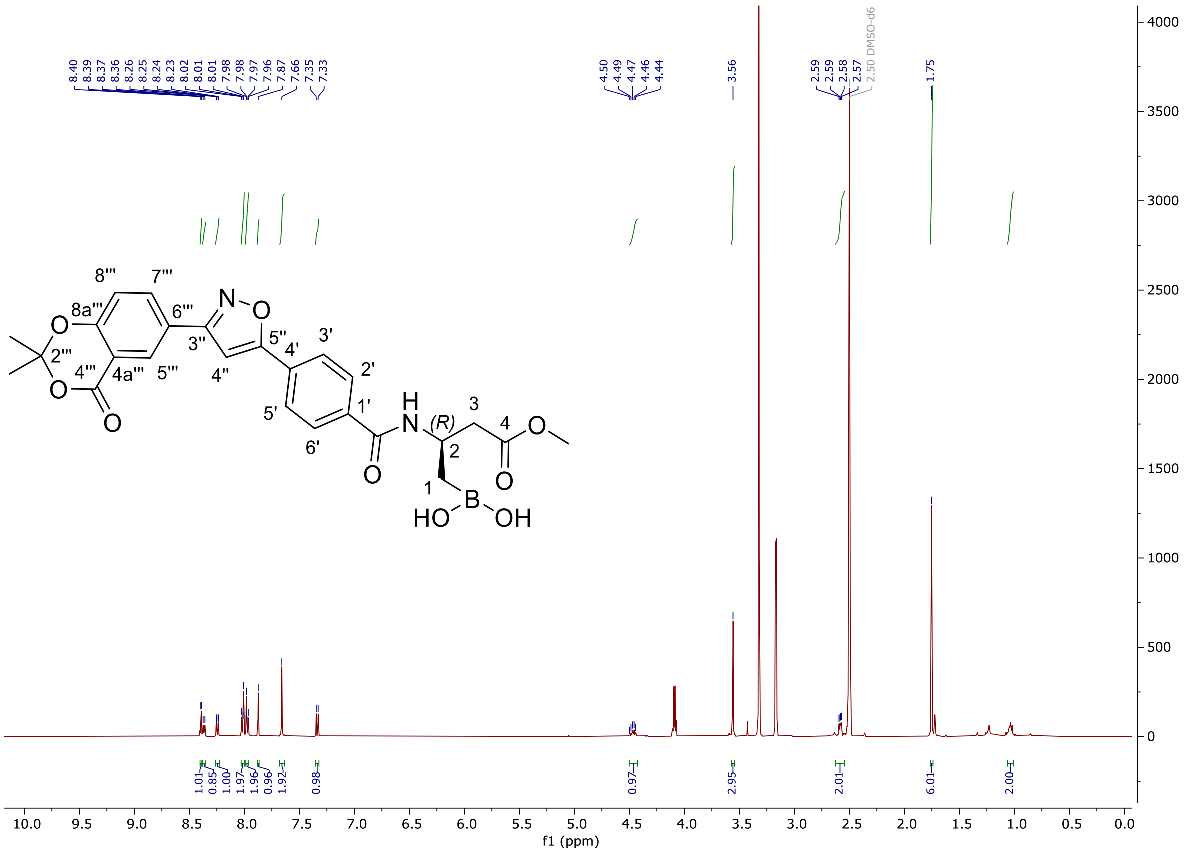

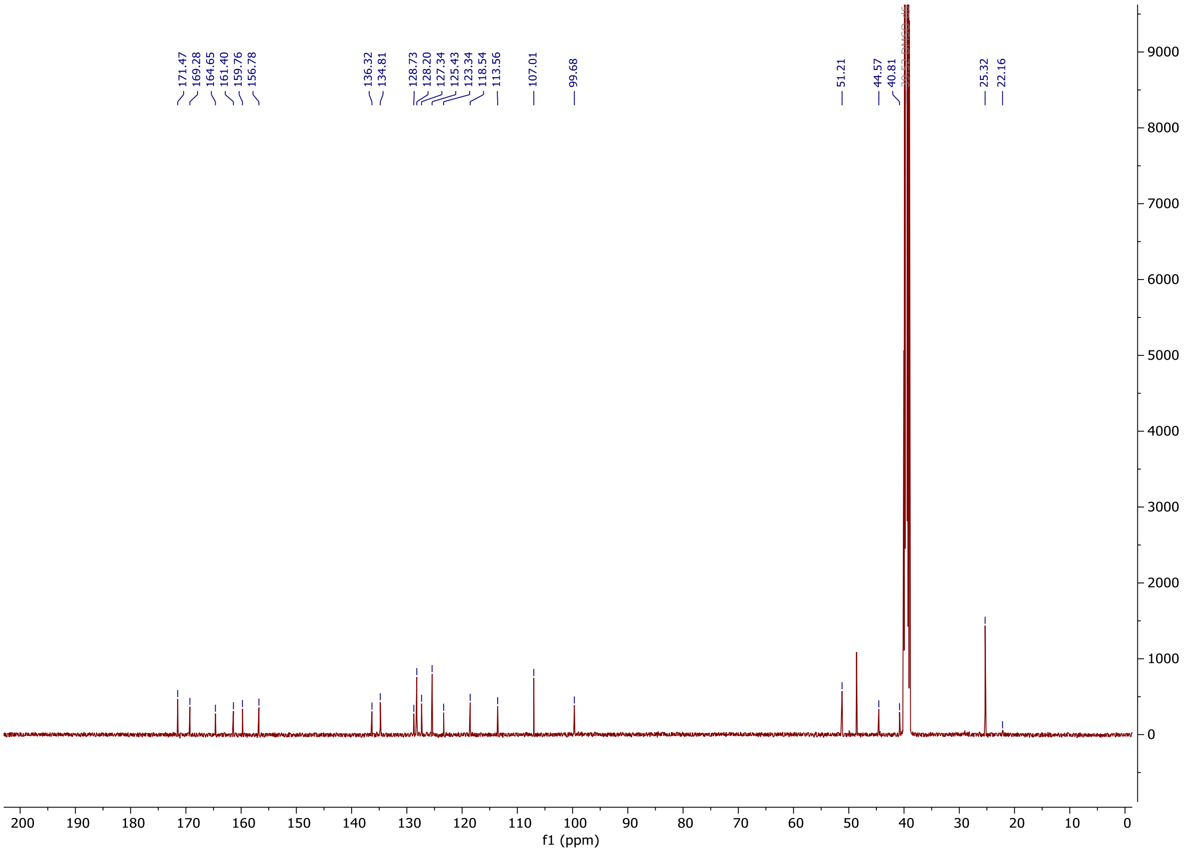

^{1}H and ^{13}C NMR spectra of (*S*)-(2-(4-(3-(2,2-Dimethyl-4-oxo-4*H*-benzo[*d*][1,3]dioxin-6-yl)isoxazol-5-yl)benzamido)-4-methoxy-4-oxobutyl)boronic acid (**37**).

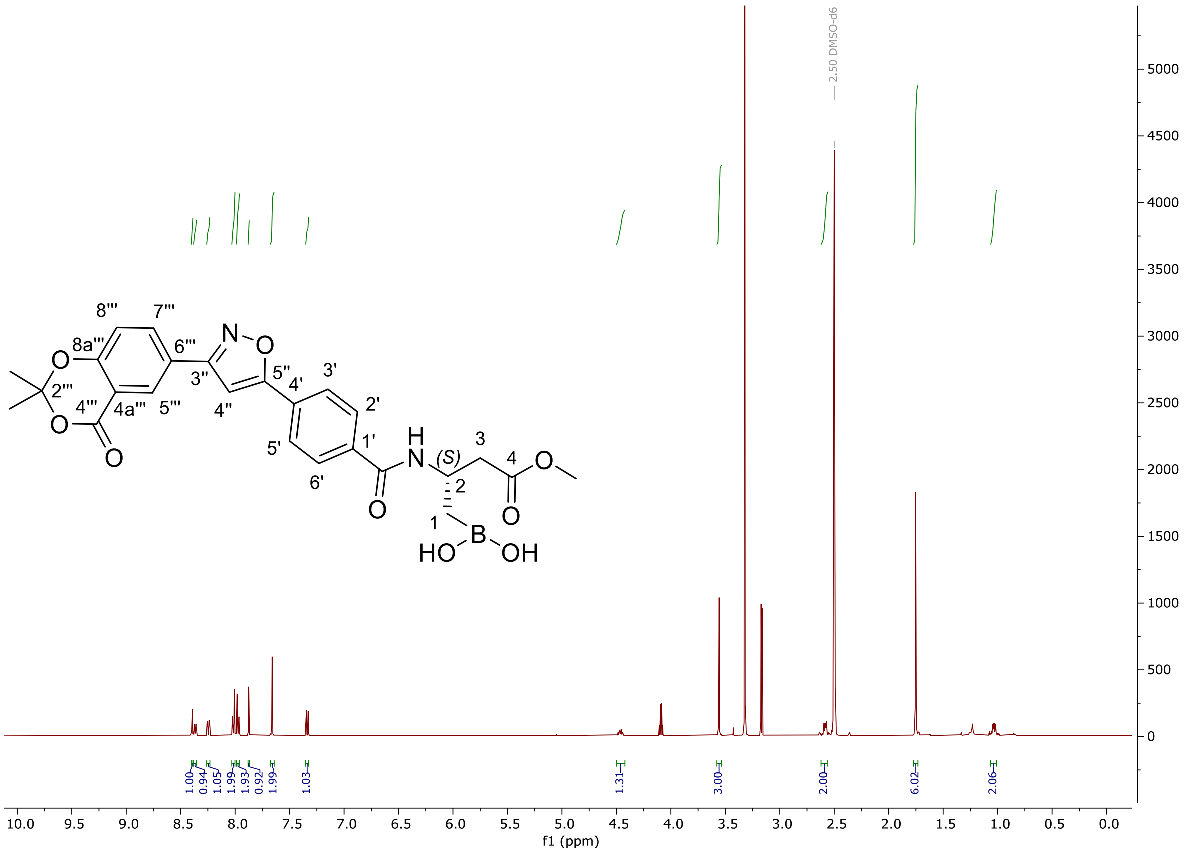

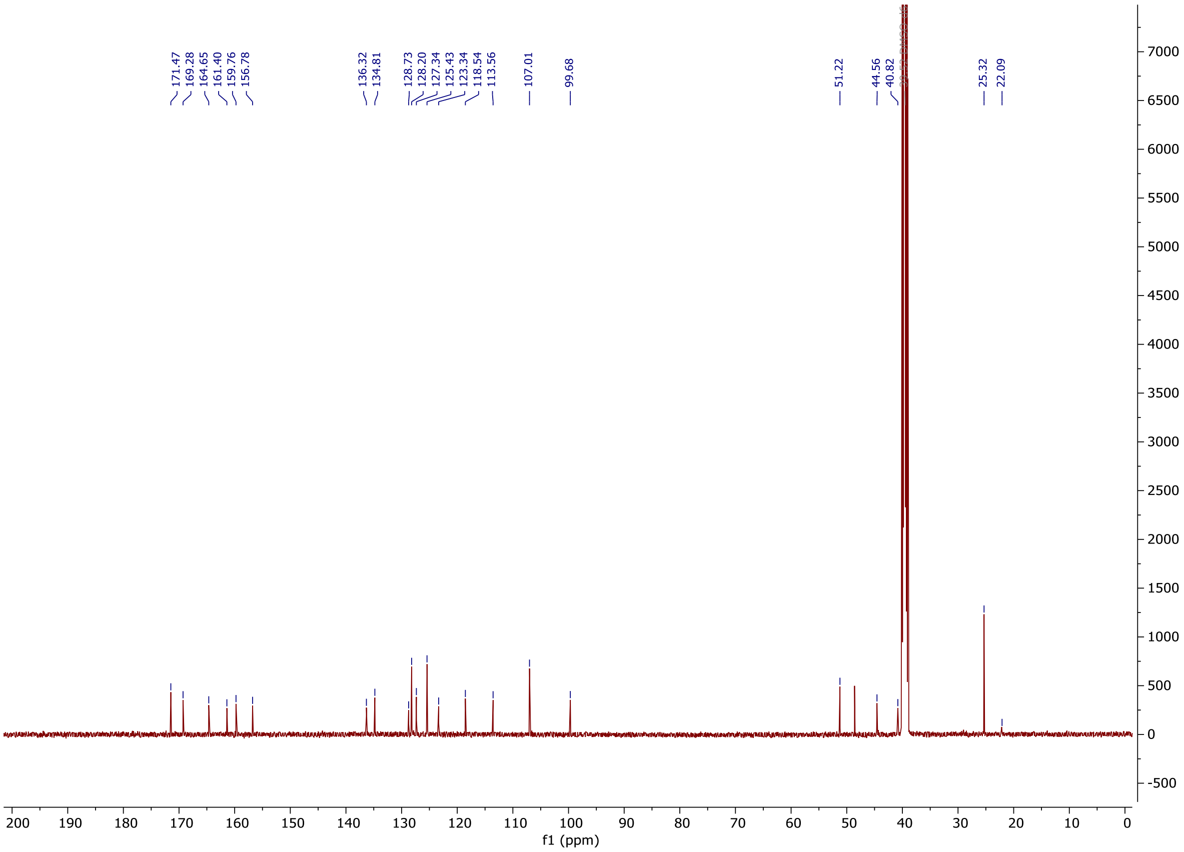

^{1}H and ^{13}C NMR spectra of benzyl (*R*)-3-(4-(1-(2,2-dimethyl-4-oxo-4*H*-benzo[*d*][1,3]dioxin-6-yl)-1*H*-1,2,3-triazol-4-yl)benzamido)-4-(4,4,5,5-tetramethyl-1,3,2-dioxaborolan-2-yl)butanoate (**38**).

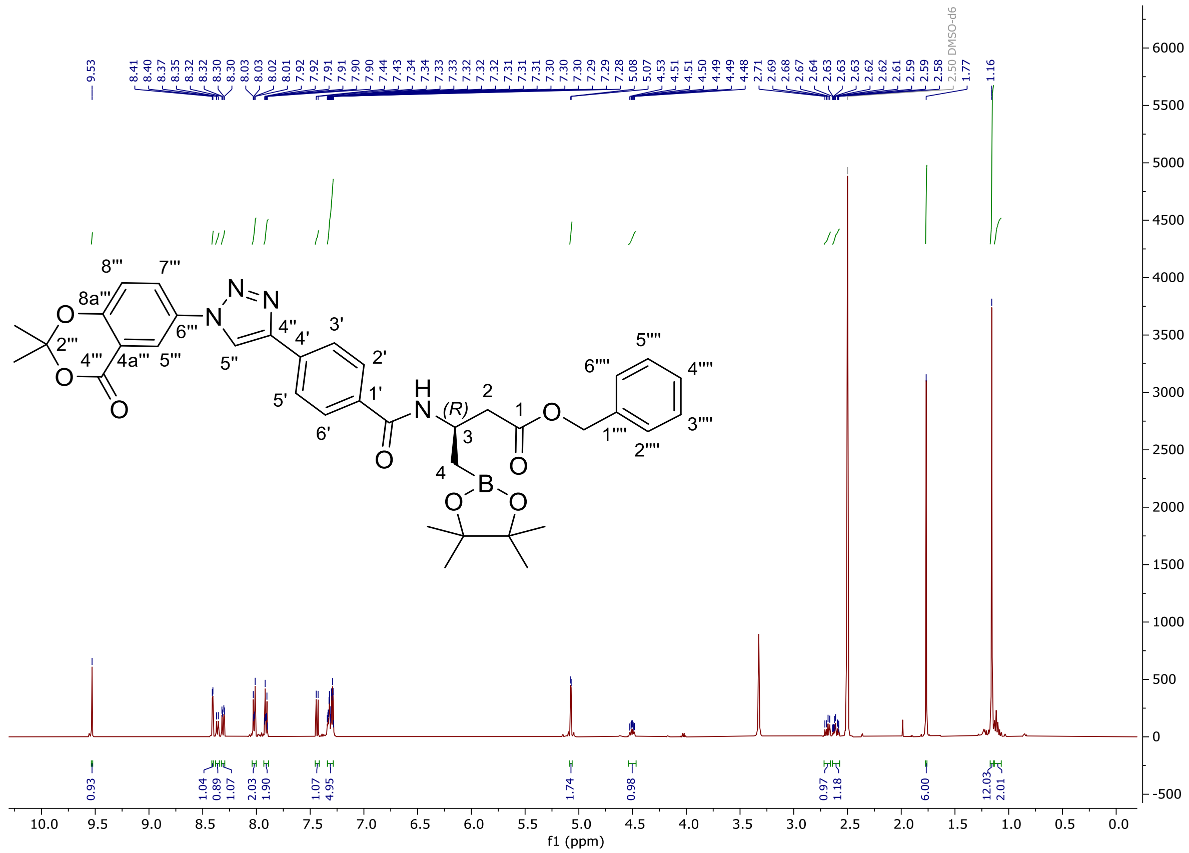

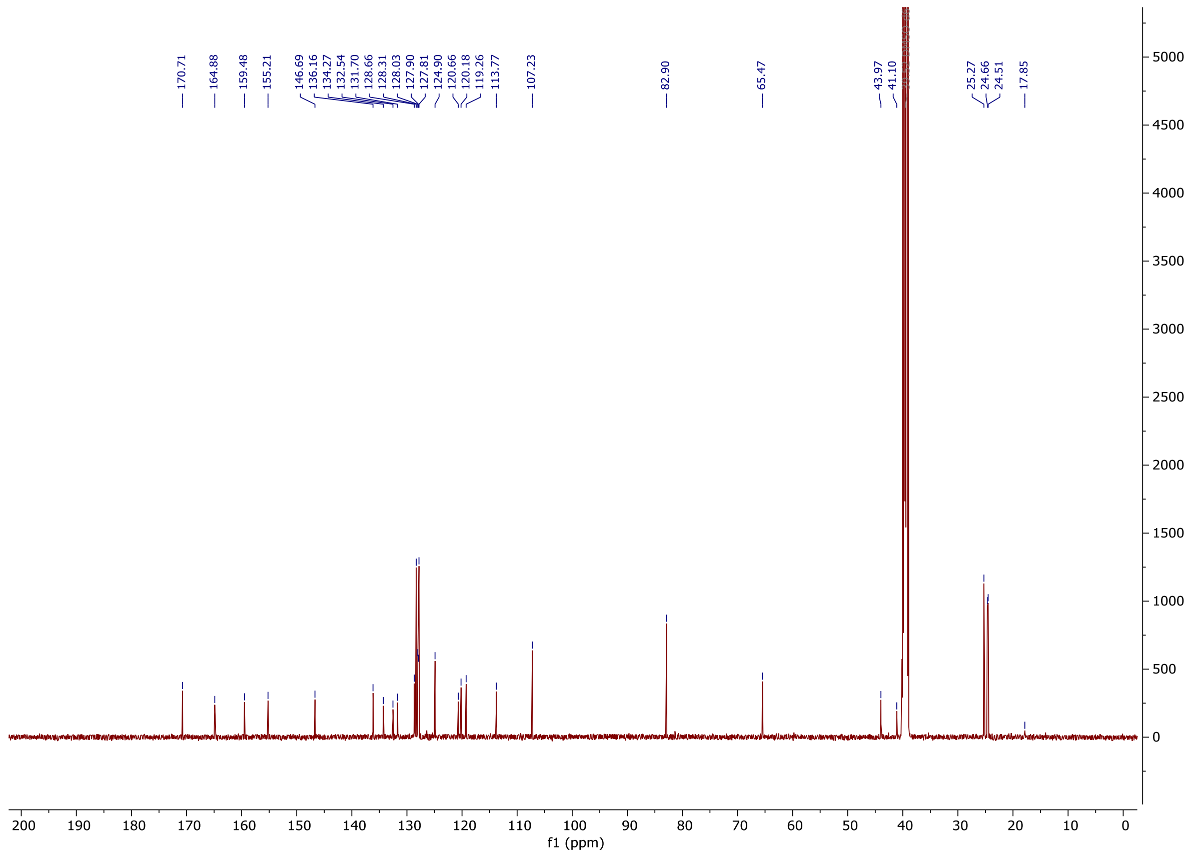

¹H and ¹³C NMR spectra of benzyl (*S*)-3-(4-(1-(2,2-dimethyl-4-oxo-4*H*-benzo[*d*][1,3]dioxin-6-yl)-1*H*-1,2,3-triazol-4-yl)benzamido)-4-(4,4,5,5-tetramethyl-1,3,2-dioxaborolan-2-yl)butanoate (**39**).

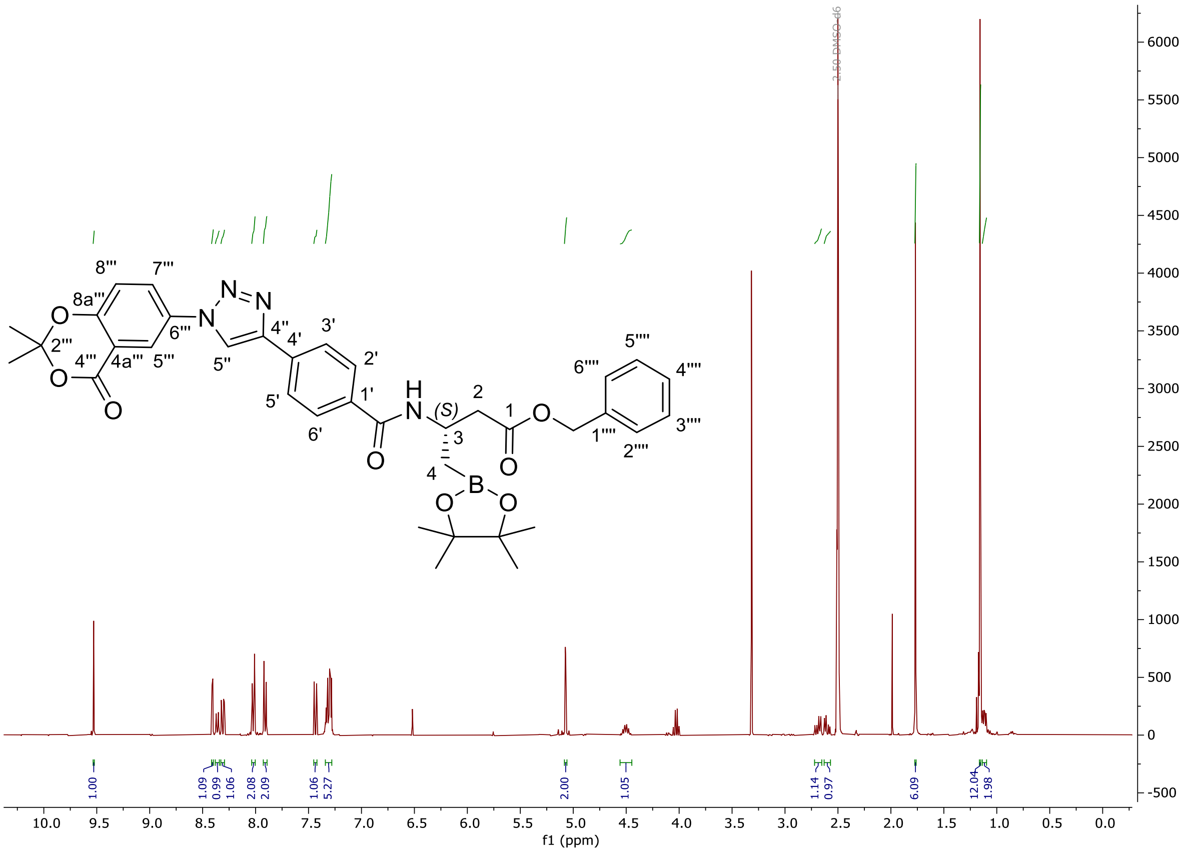

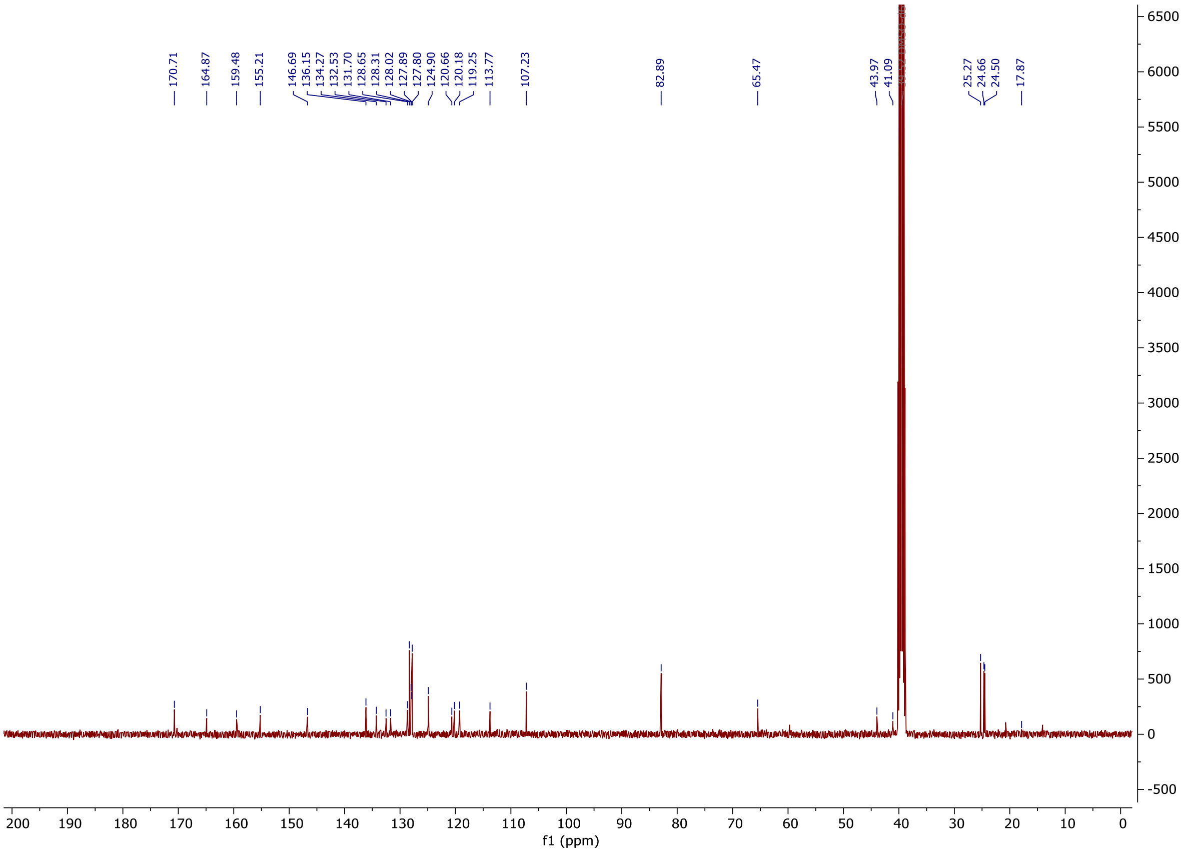

^{1}H and ^{13}C NMR spectra of benzyl (*R*)-4-cyano-3-(4-(1-(2,2-dimethyl-4-oxo-4*H*-benzo[*d*][1,3]dioxin-6-yl)-1*H*-1,2,3-triazol-4-yl)benzamido)butanoate (**40**).

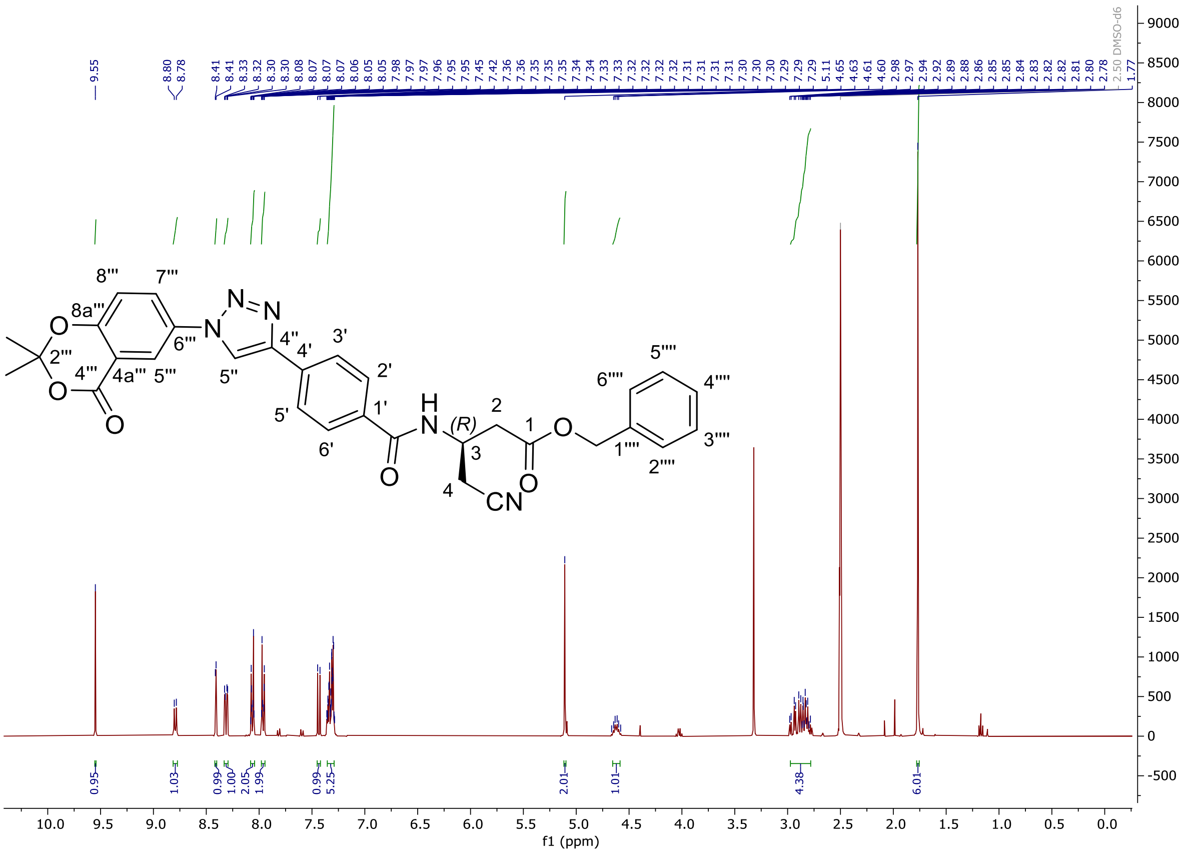

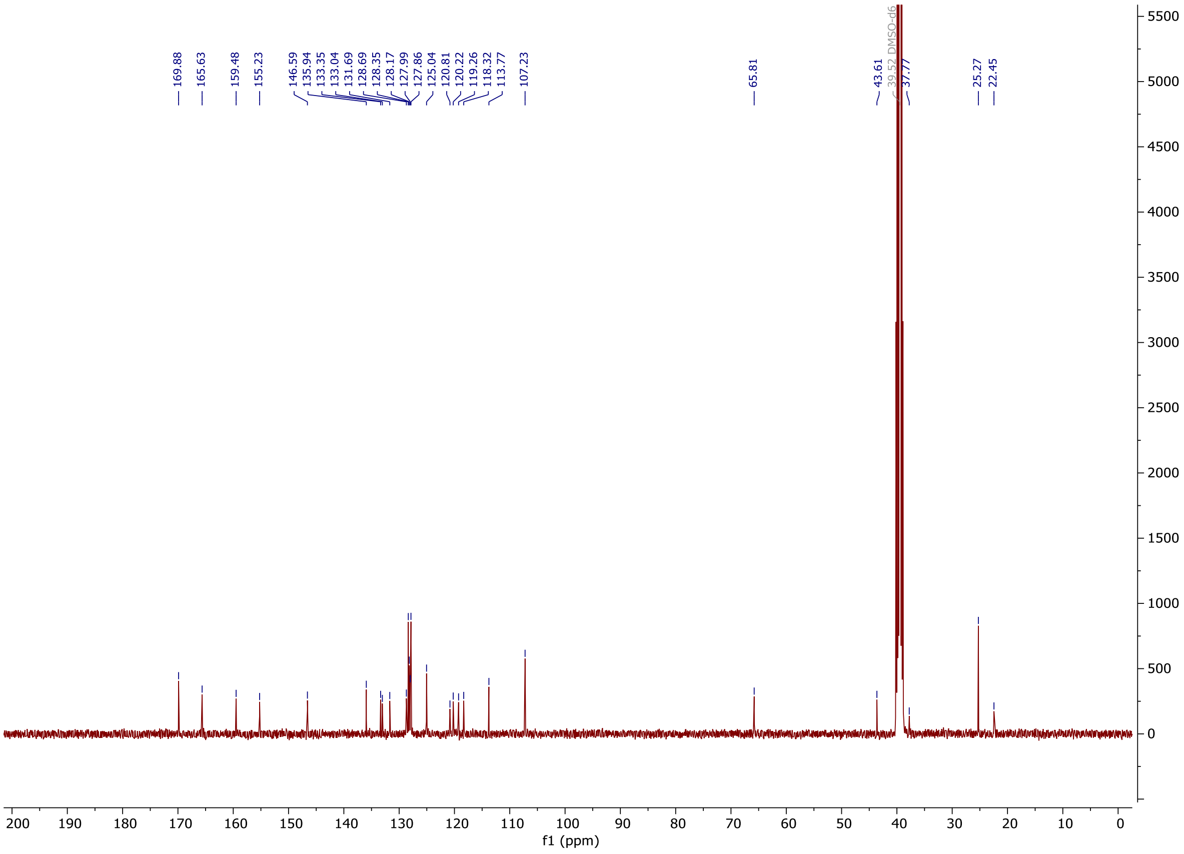

^{1}H and ^{13}C NMR spectra of benzyl (*S*)-4-cyano-3-(4-(1-(2,2-dimethyl-4-oxo-4*H*-benzo[*d*][1,3]dioxin-6-yl)-1*H*-1,2,3-triazol-4-yl)benzamido)butanoate (**41**).

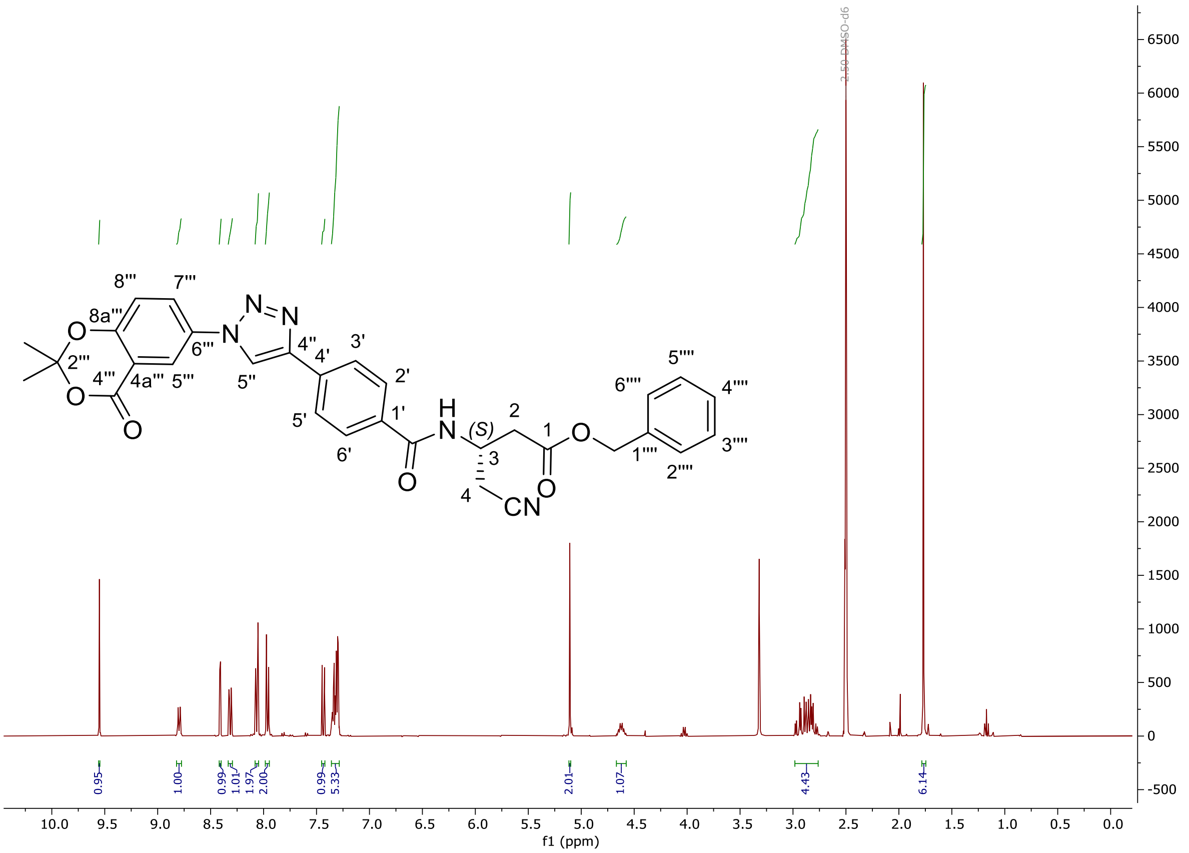

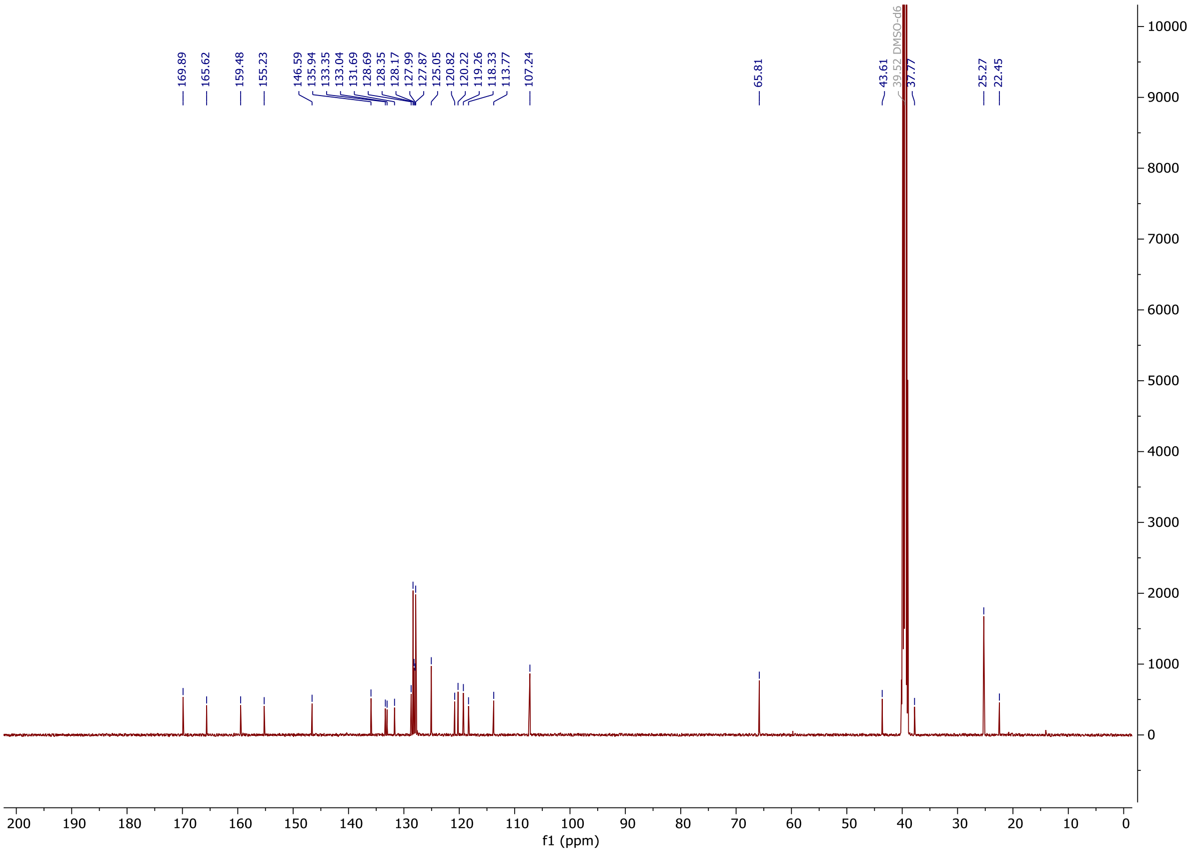

^{1}H and ^{13}C NMR spectra of (*R*)-(4-(benzyloxy)-2-(4-(1-(2,2-dimethyl-4-oxo-4*H*-benzo[*d*][1,3]dioxin-6-yl)-1*H*-1,2,3-triazol-4-yl)benzamido)-4-oxobutyl)boronic acid (**42**).

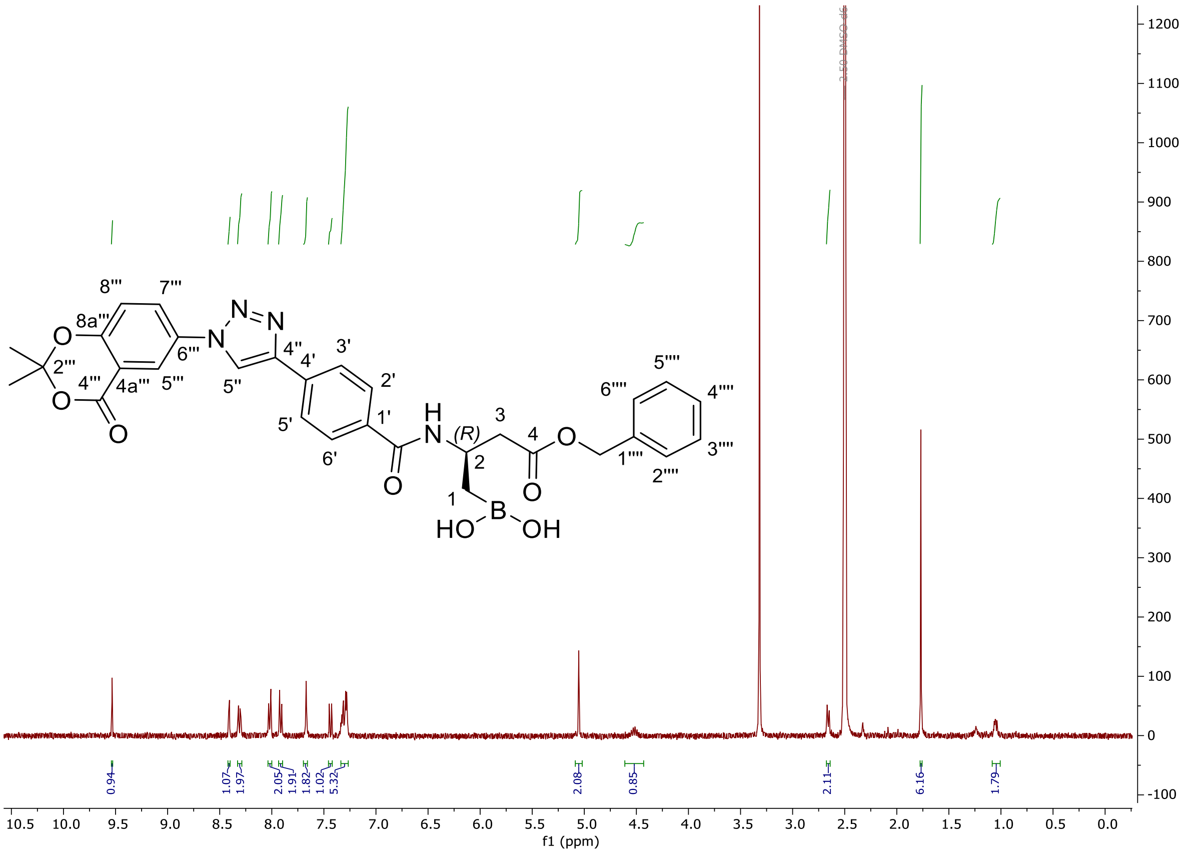

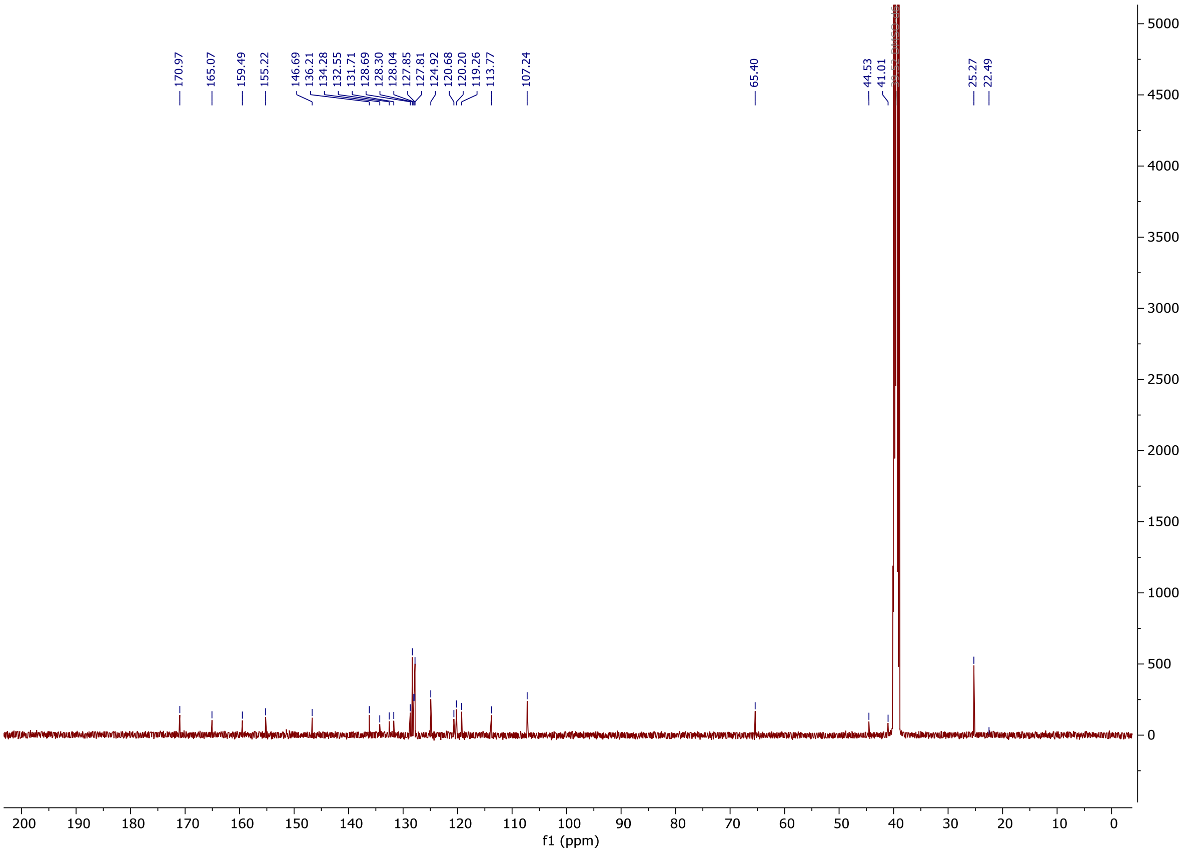

^{1}H and ^{13}C NMR spectra of (*S*)-(4-(benzyloxy)-2-(4-(1-(2,2-dimethyl-4-oxo-4*H*-benzo[*d*][1,3]dioxin-6-yl)-1*H*-1,2,3-triazol-4-yl)benzamido)-4-oxobutyl)boronic acid (**43**).

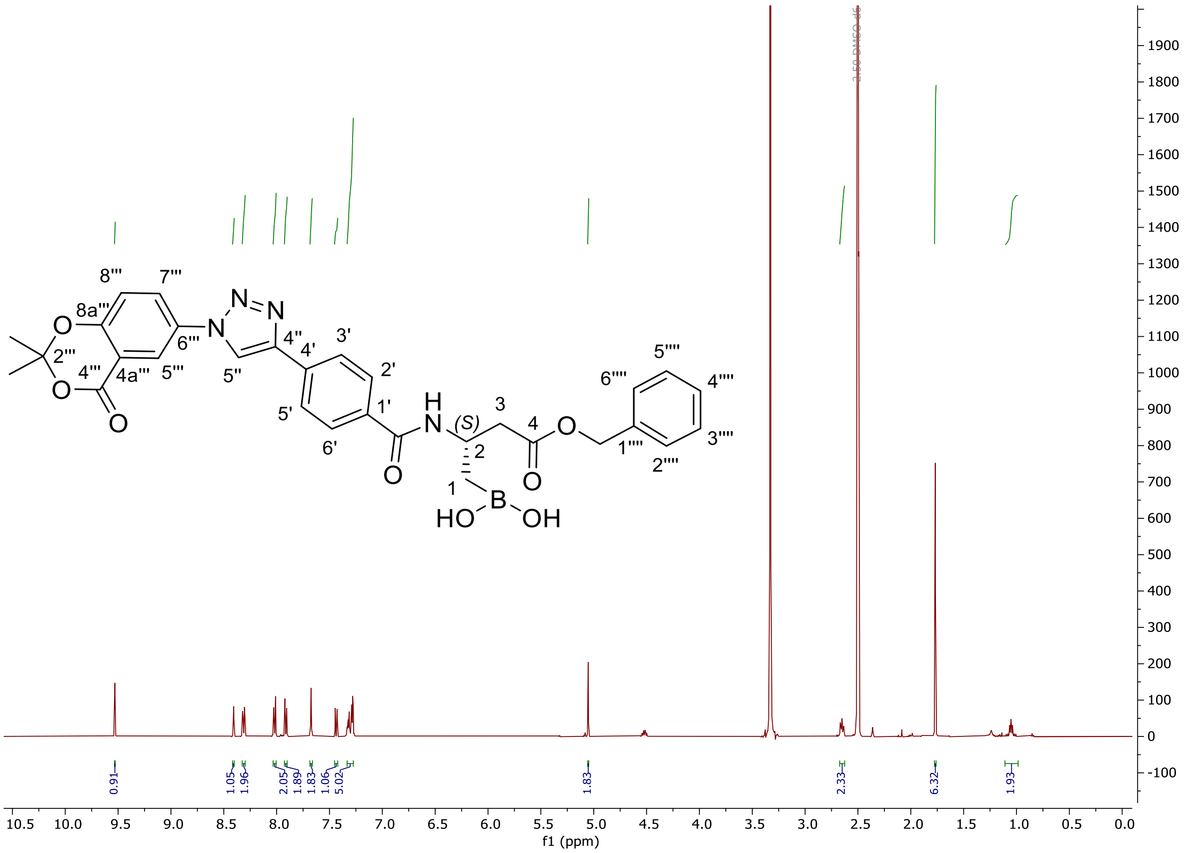

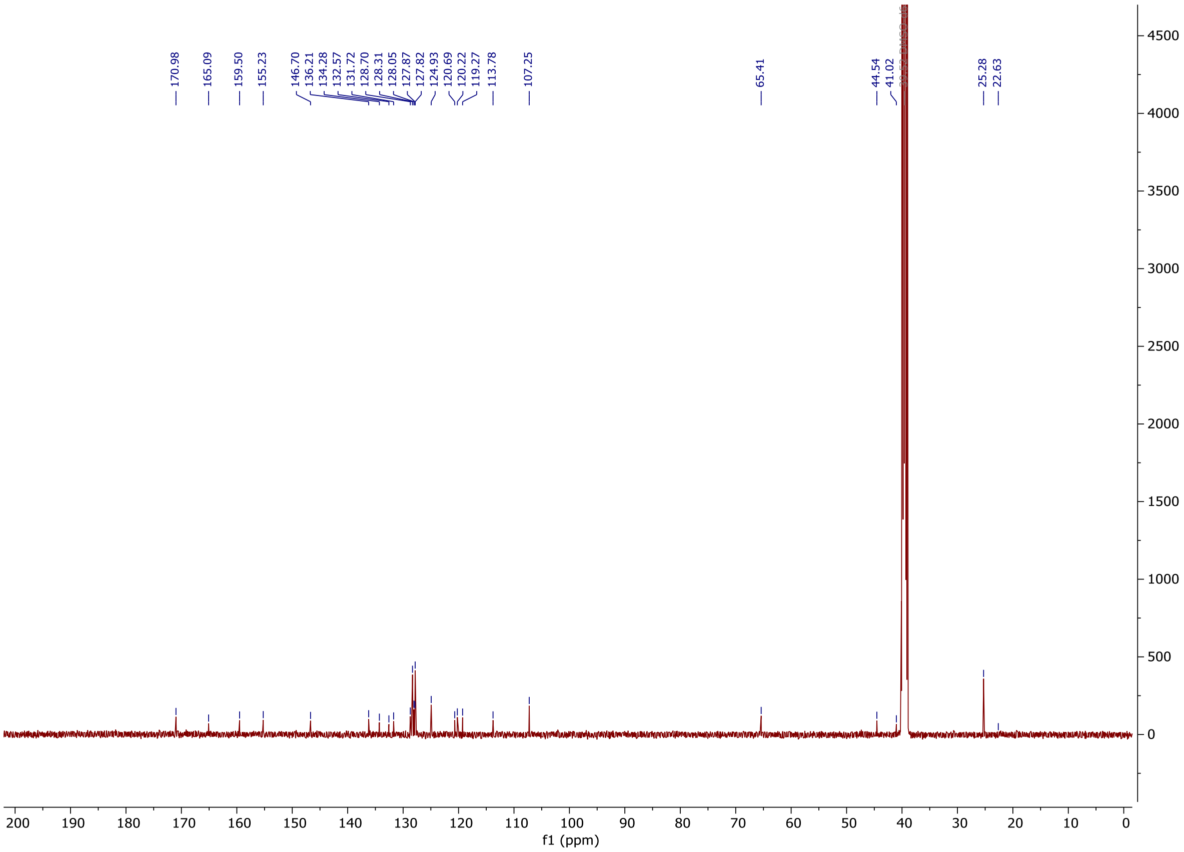

¹H and ¹³C NMR spectra of (*S*)-4-(benzyloxy)-1-bromo-4-oxobutan-2-aminium (**44**).

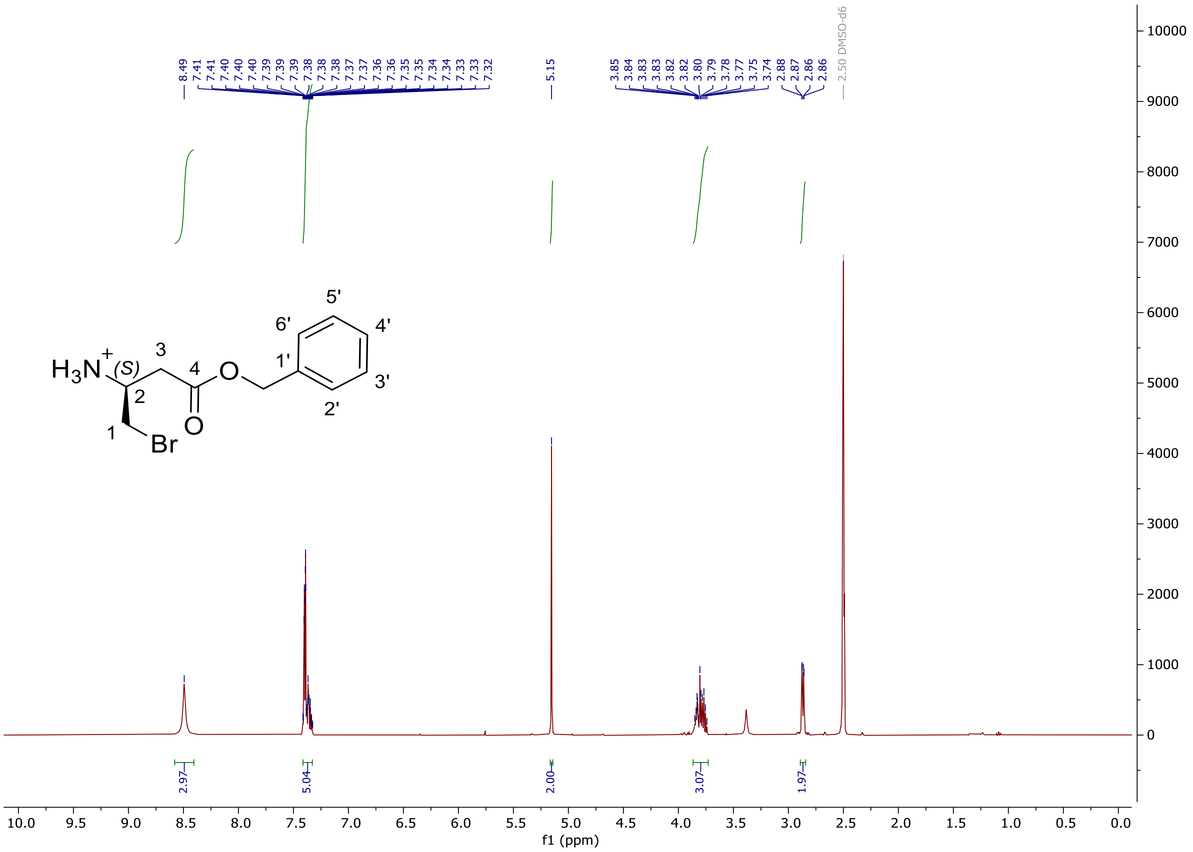

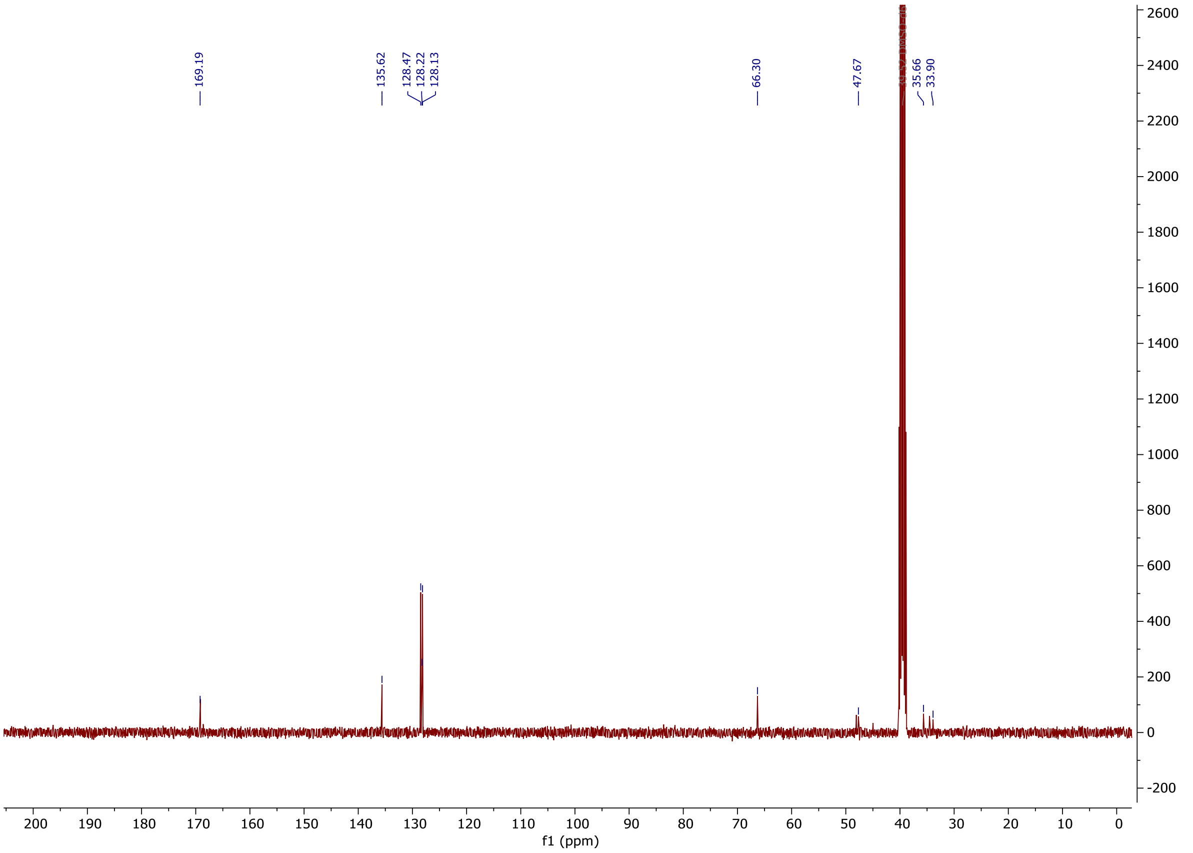

^{1}H and ^{13}C NMR spectra of benzyl (*S*)-2-(2-(4-ethynylphenyl)-4,5-dihydrooxazol-4-yl)acetate (**45**).

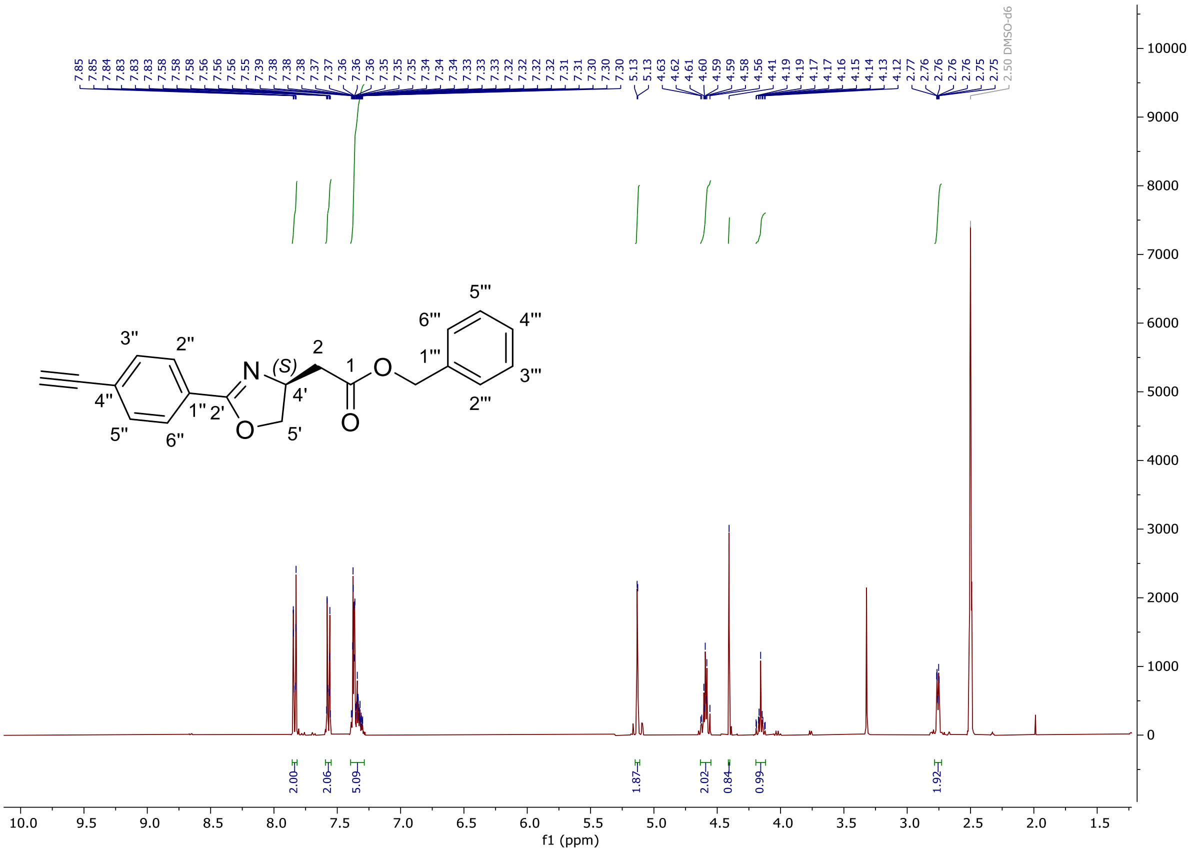

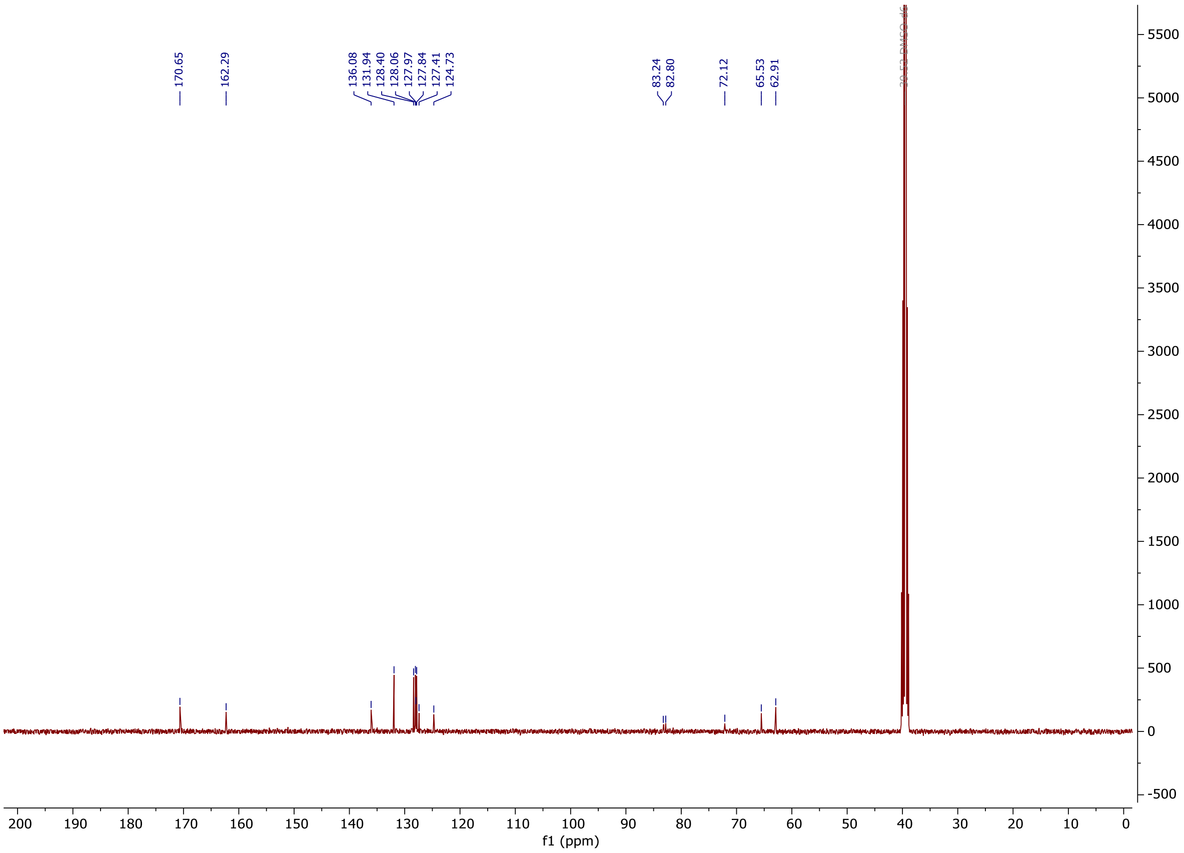

^{1}H and ^{13}C NMR spectra of (*R*)-5-(5-(4-((1-carboxy-3-cyanopropan-2-yl)carbamoyl)phenyl)isoxazol-3-yl)-2-hydroxybenzoic acid (**47**).

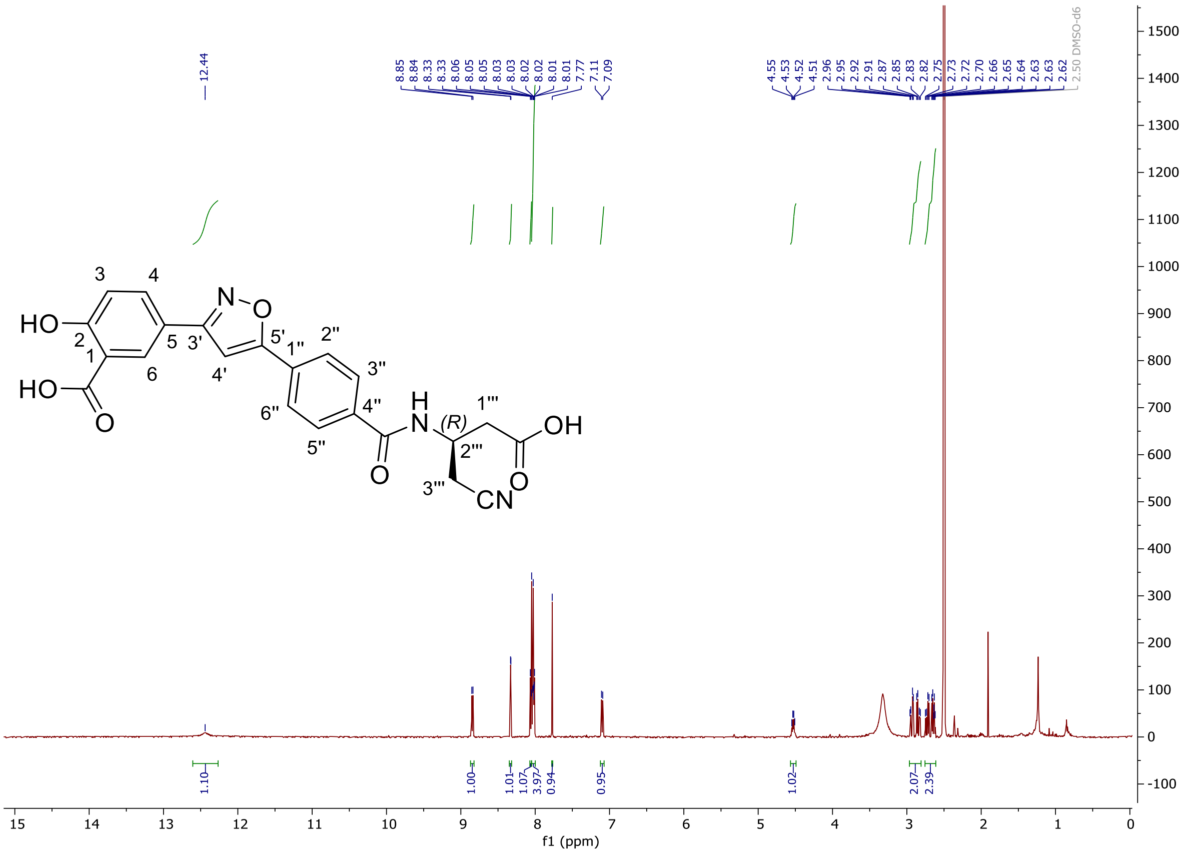

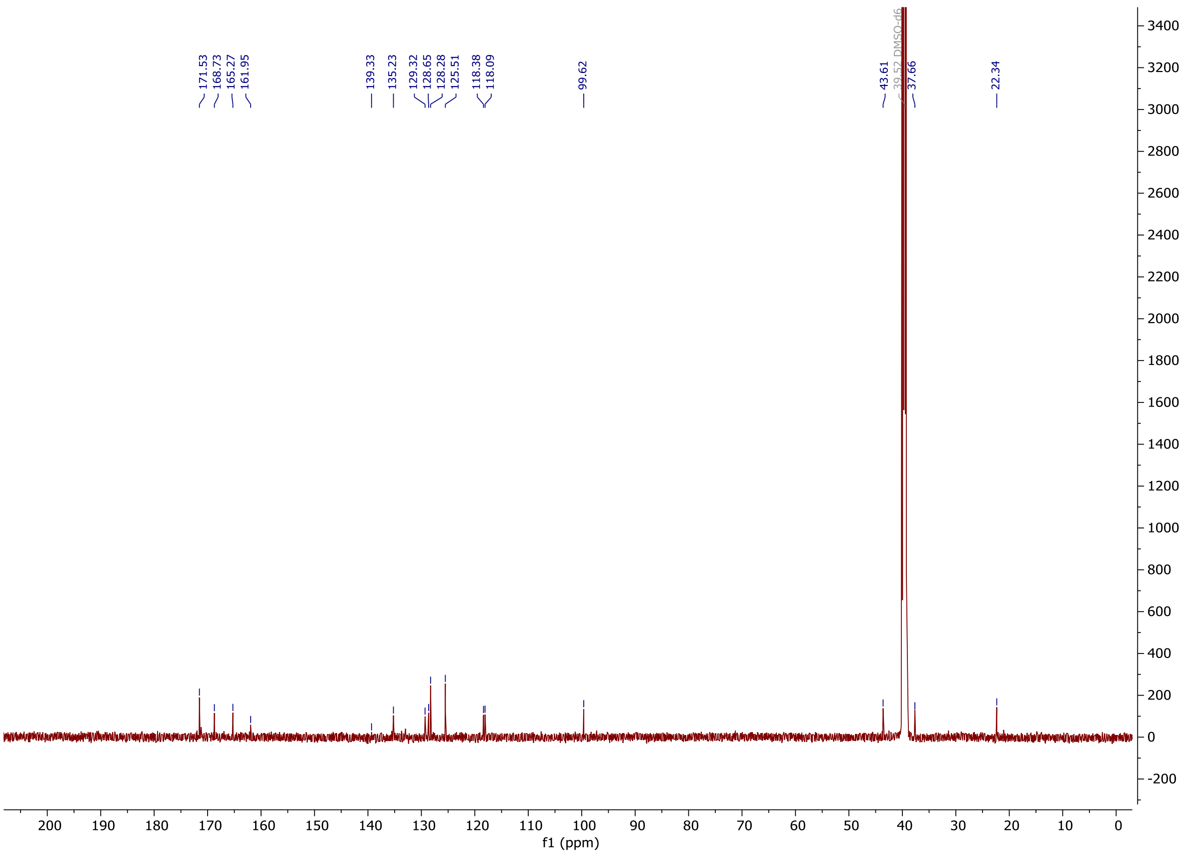

^{1}H and ^{13}C NMR spectra of (*S*)-5-(5-(4-((1-carboxy-3-cyanopropan-2-yl)carbamoyl)phenyl)isoxazol-3-yl)-2-hydroxybenzoic acid (**48**).

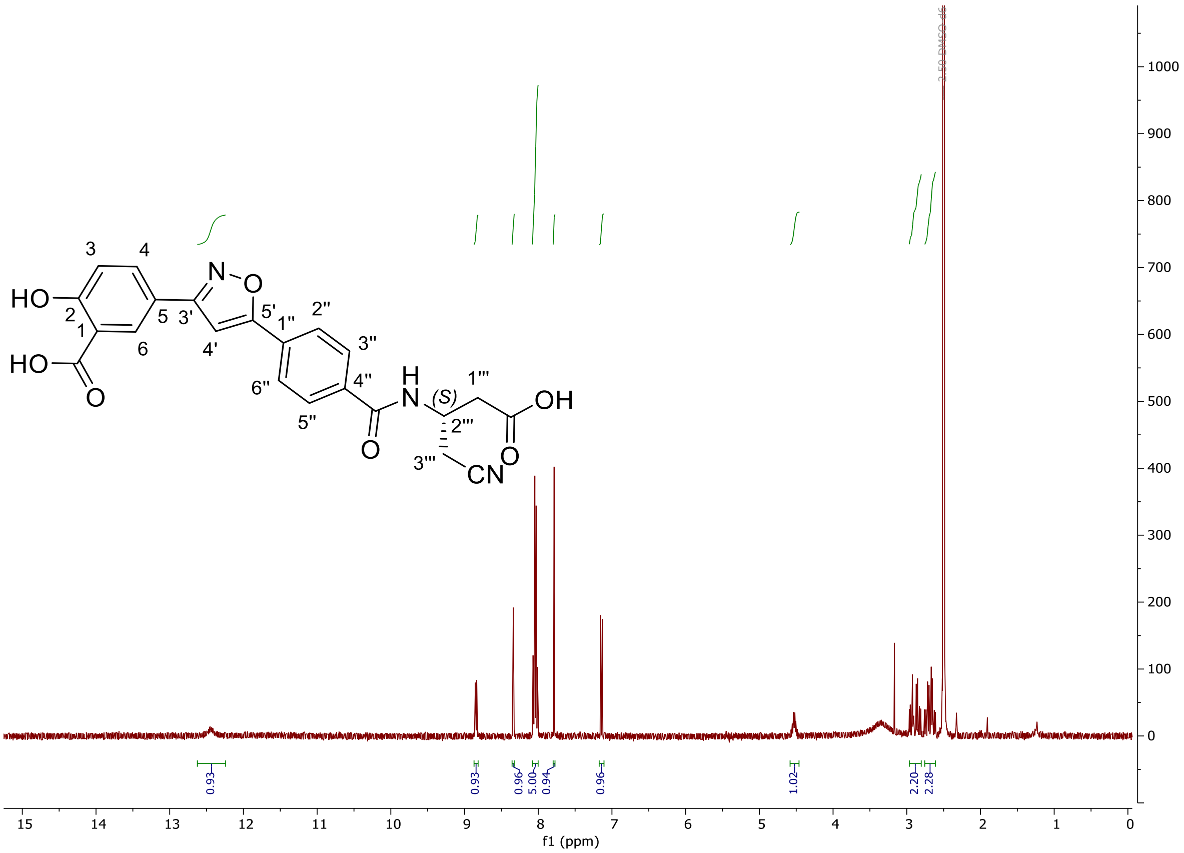

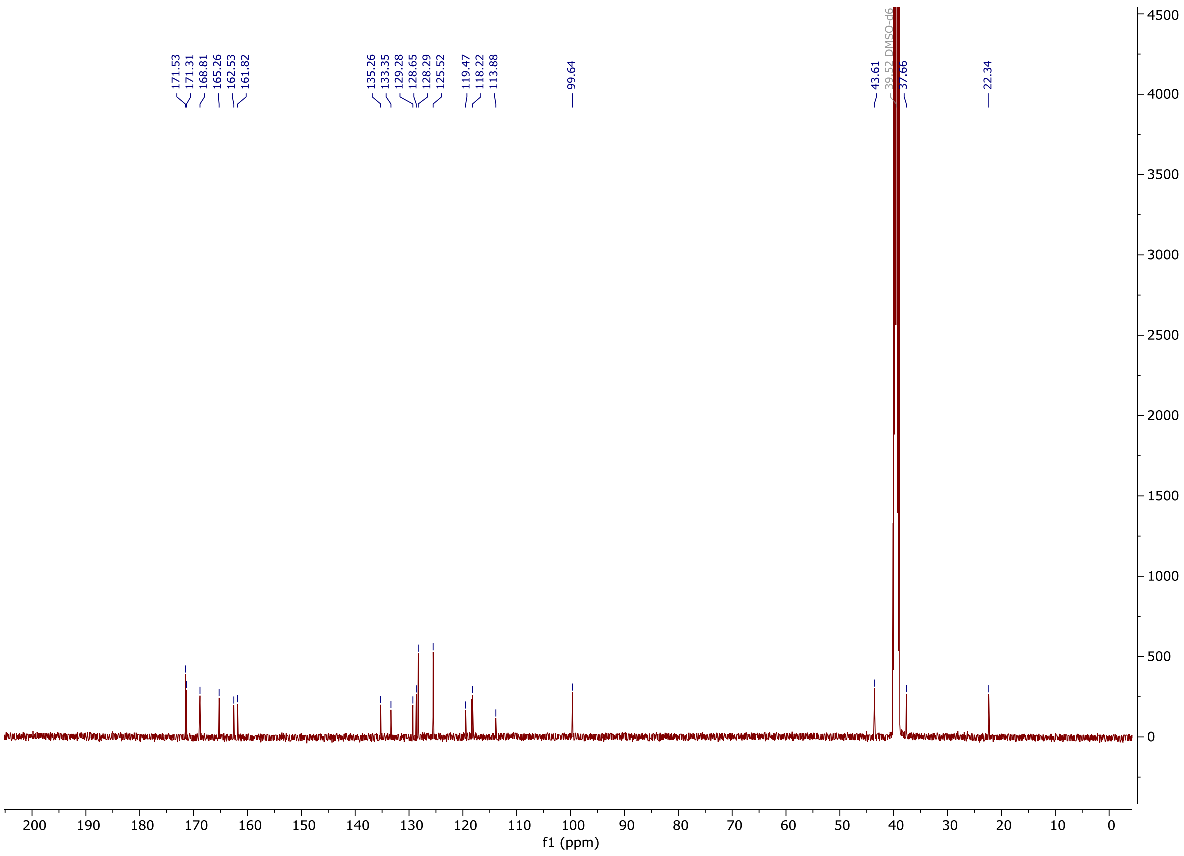

^{1}H and ^{13}C NMR spectra of (*R*)-5-(4-(4-((1-carboxy-3-cyanopropan-2-yl)carbamoyl)phenyl)-1*H*-1,2,3-triazol-1-yl)-2-hydroxybenzoic acid (**49**).

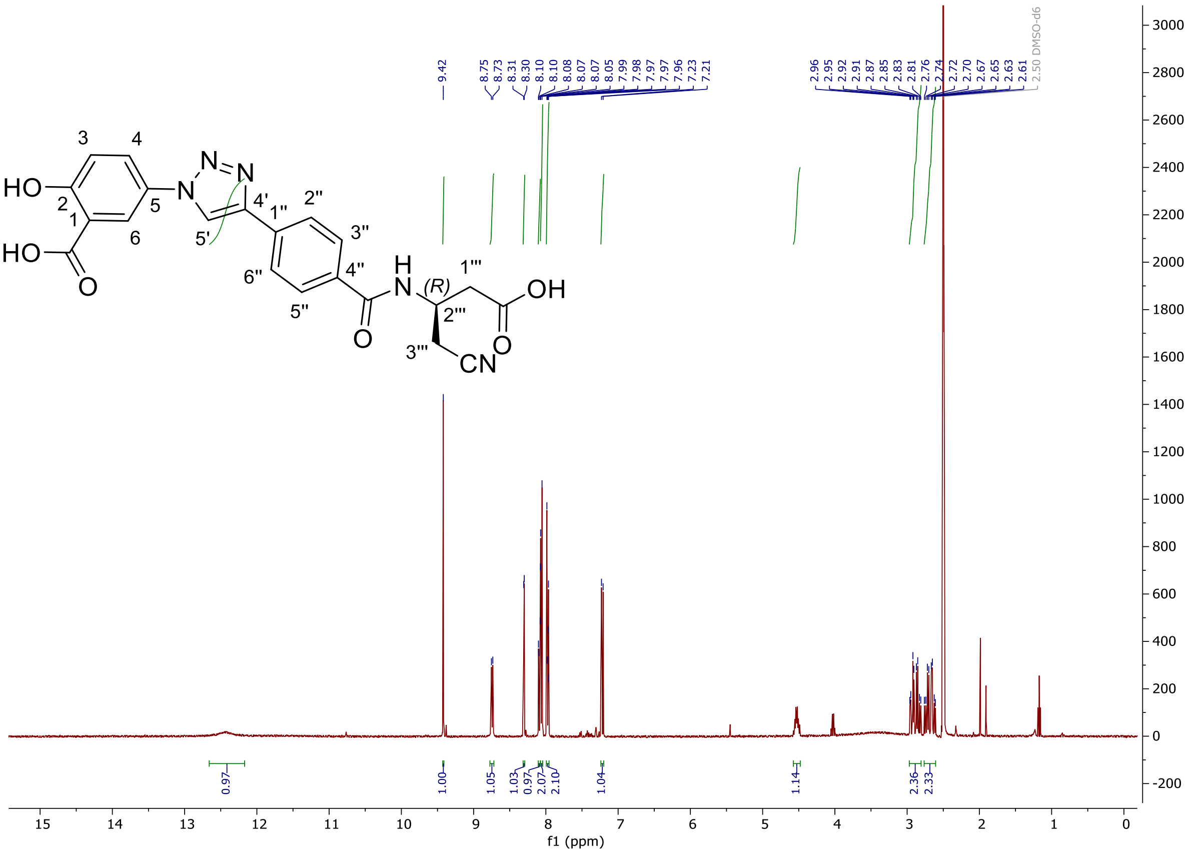

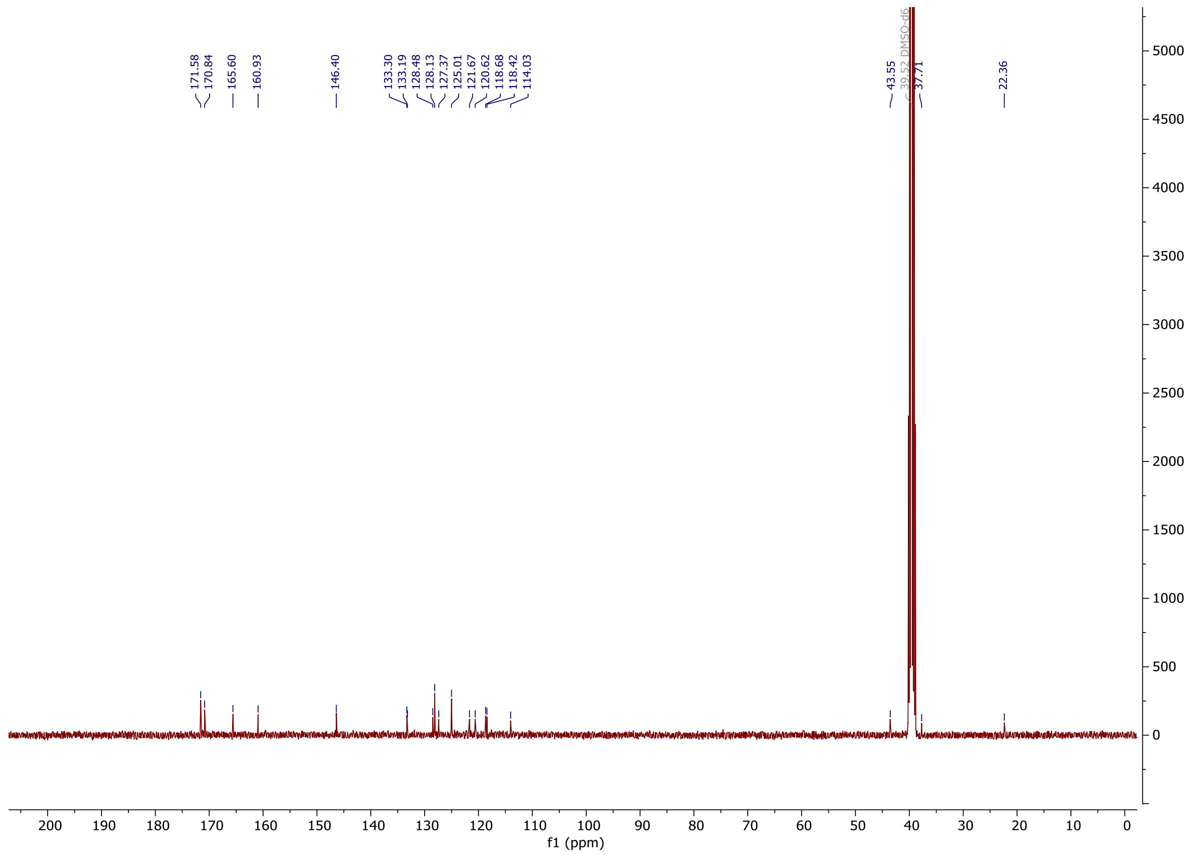

^{1}H and ^{13}C NMR spectra of (*S*)-5-(4-(4-((1-carboxy-3-cyanopropan-2-yl)carbamoyl)phenyl)-1*H*-1,2,3-triazol-1-yl)-2-hydroxybenzoic acid (**50**).

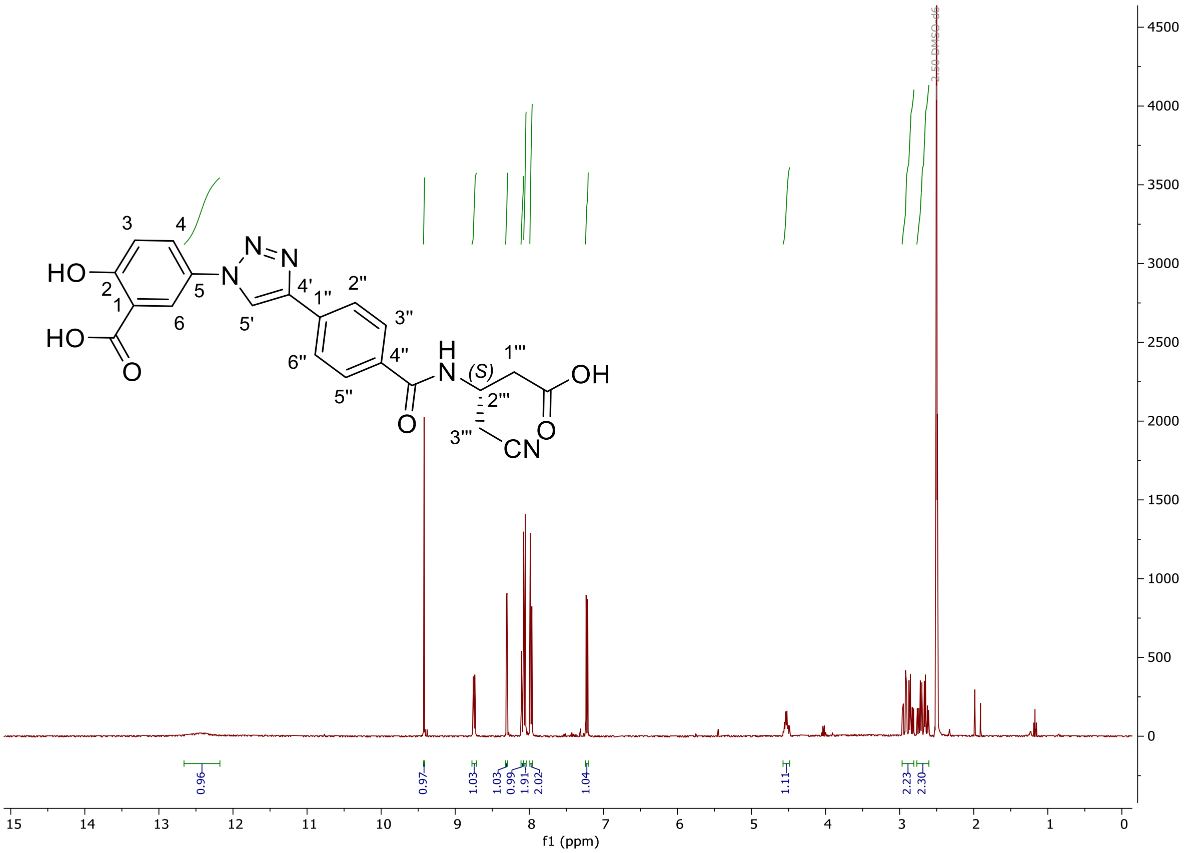

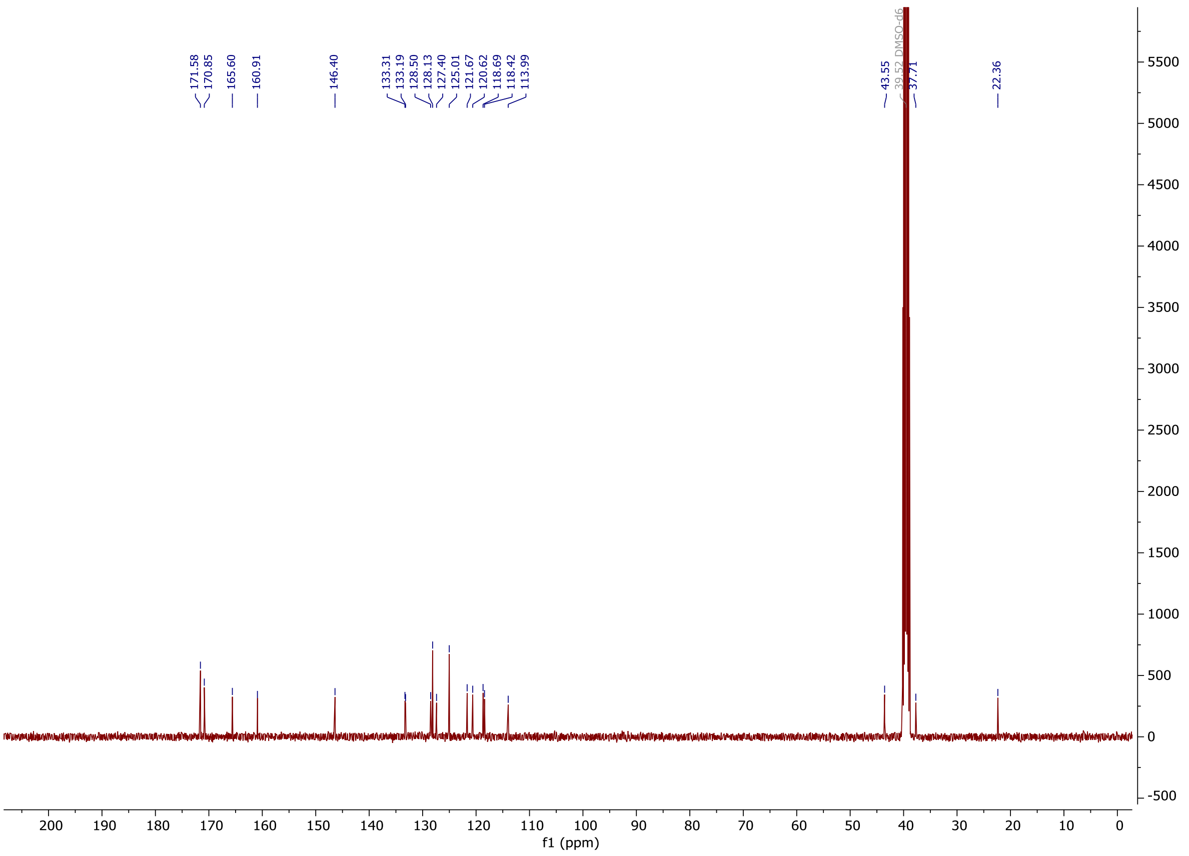

¹H and ¹³C NMR spectra of (*R*)-2-(6-(4-(3-(2,2-dimethyl-4-oxo-4*H*-benzo[*d*][1,3]dioxin-6-yl)isoxazol-5-yl)phenyl)-2-hydroxy-3,4-dihydro-2*H*-1,5,2-oxazaborinin-4-yl)acetic acid (**51**).

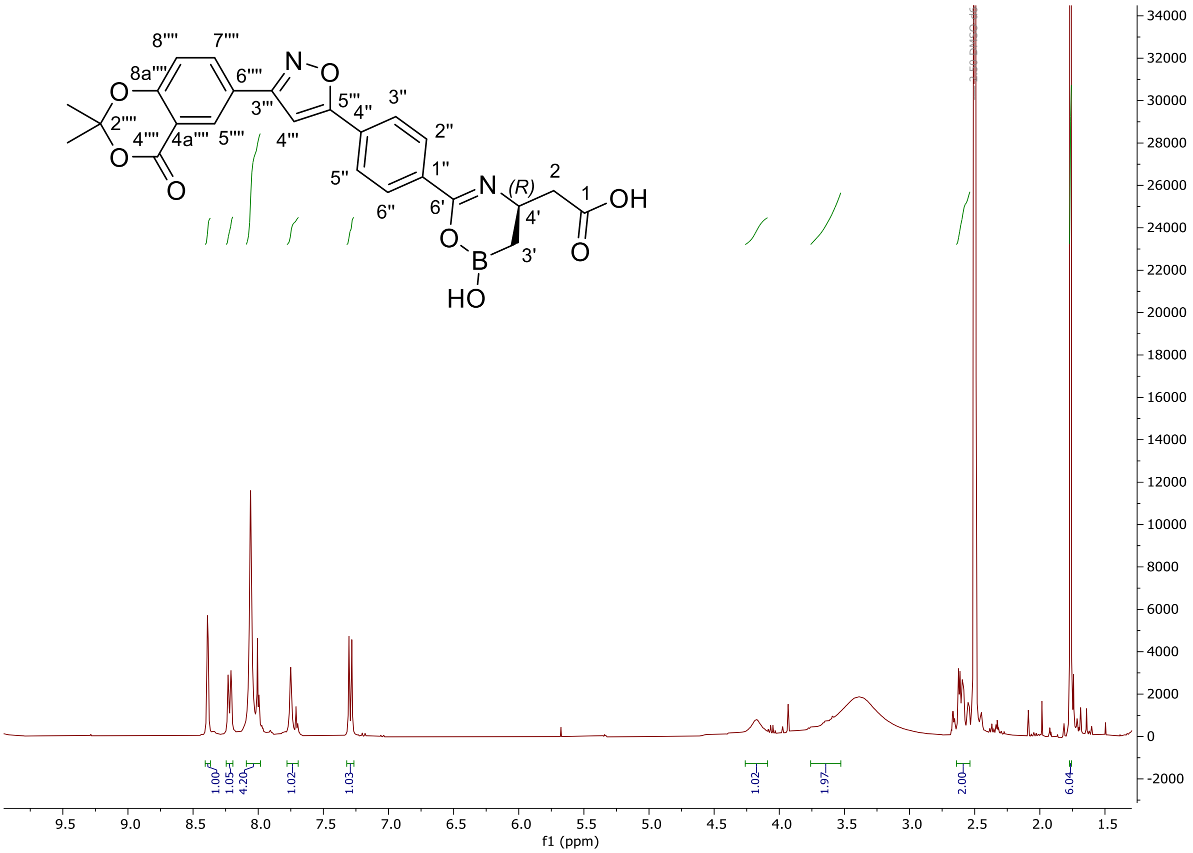

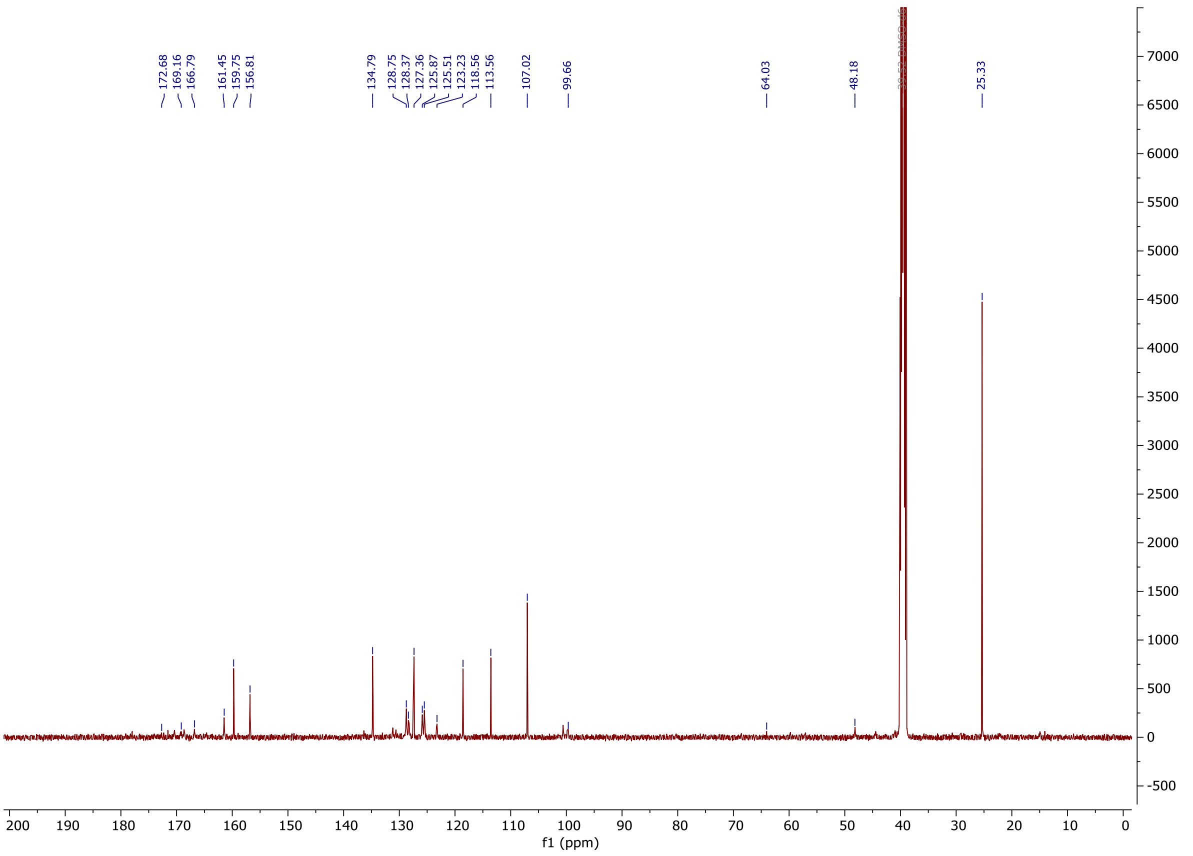

^{1}H and ^{13}C NMR spectra of (*S*)-2-(6-(4-(3-(2,2-dimethyl-4-oxo-4*H*-benzo[*d*][1,3]dioxin-6-yl)isoxazol-5-yl)phenyl)-2-hydroxy-3,4-dihydro-2*H*-1,5,2-oxazaborinin-4-yl)acetic acid (**52**).

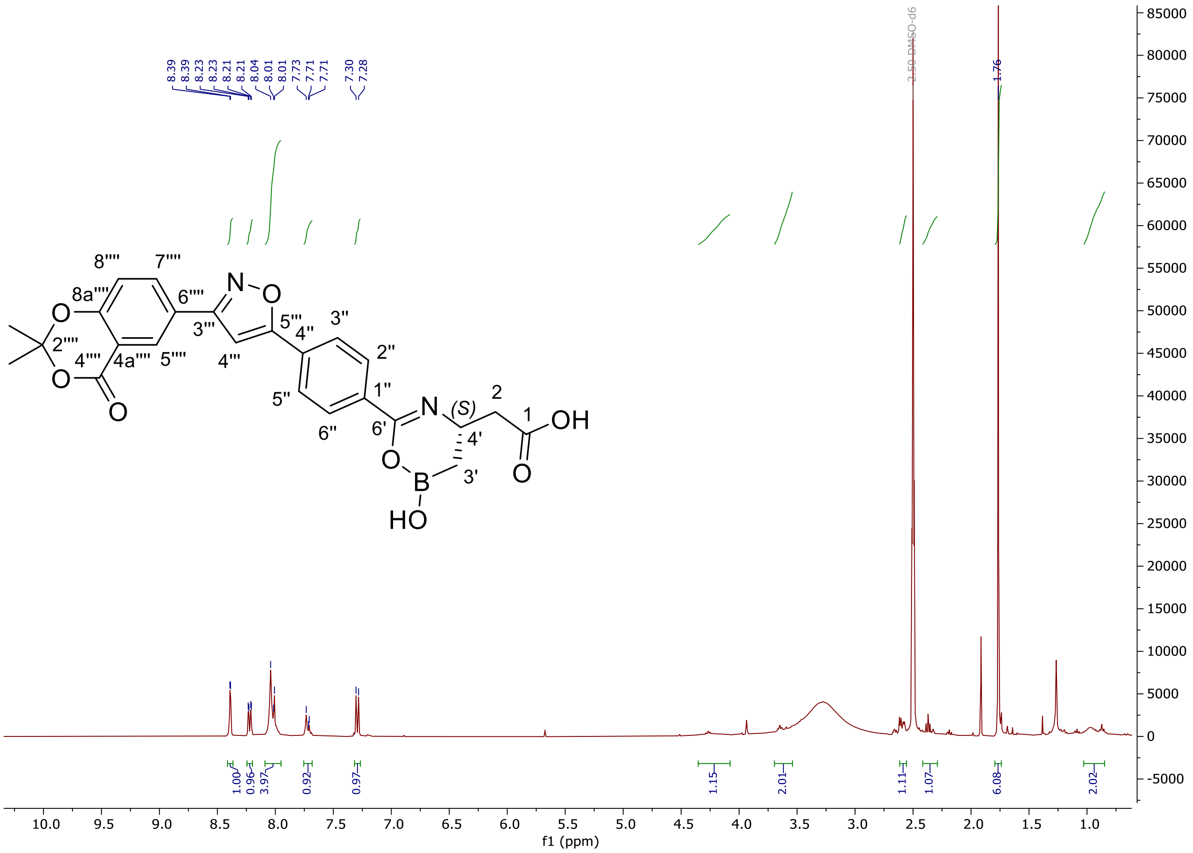

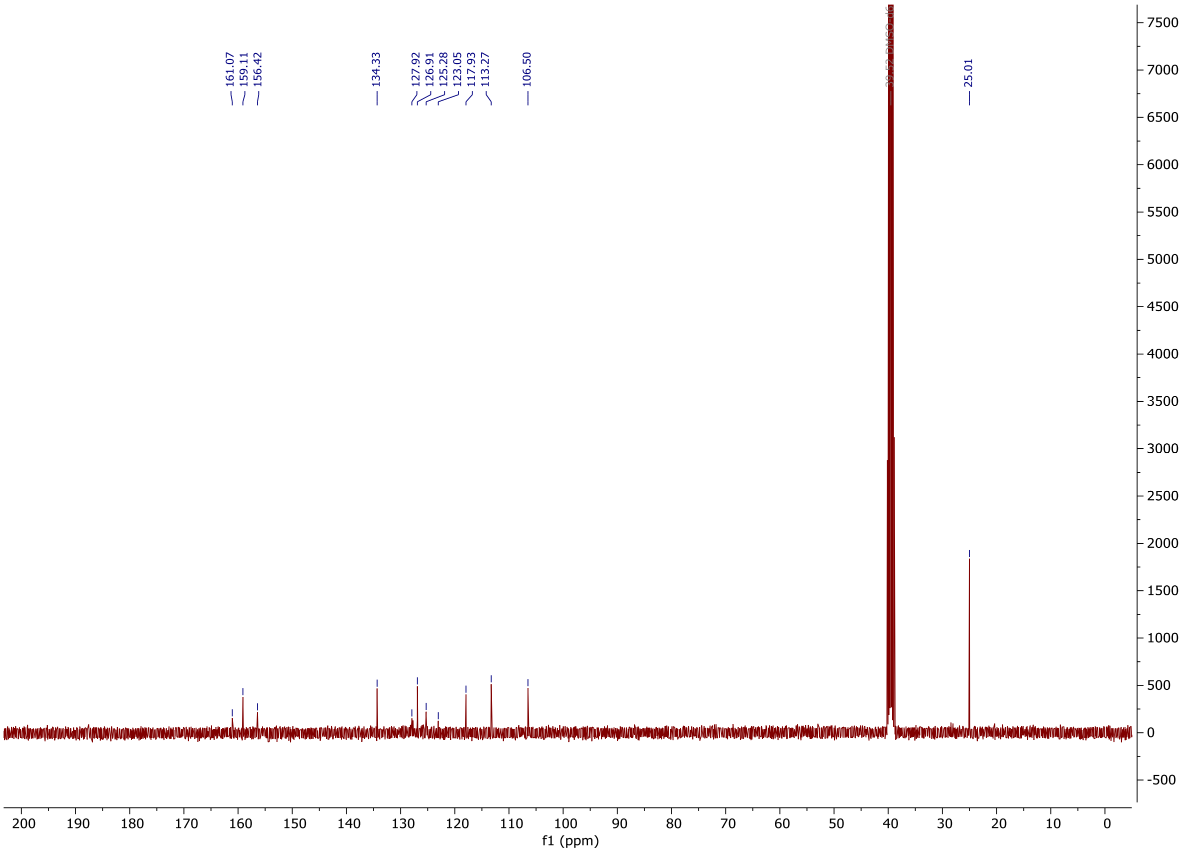

^{1}H and ^{13}C NMR spectra of (*R*)-2-(6-(4-(1-(2,2-dimethyl-4-oxo-4*H*-benzo[*d*][1,3]dioxin-6-yl)-1*H*-1,2,3-triazol-4-yl)phenyl)-2-hydroxy-3,4-dihydro-2*H*-1,5,2-oxazaborinin-4-yl)acetic acid (**53**).

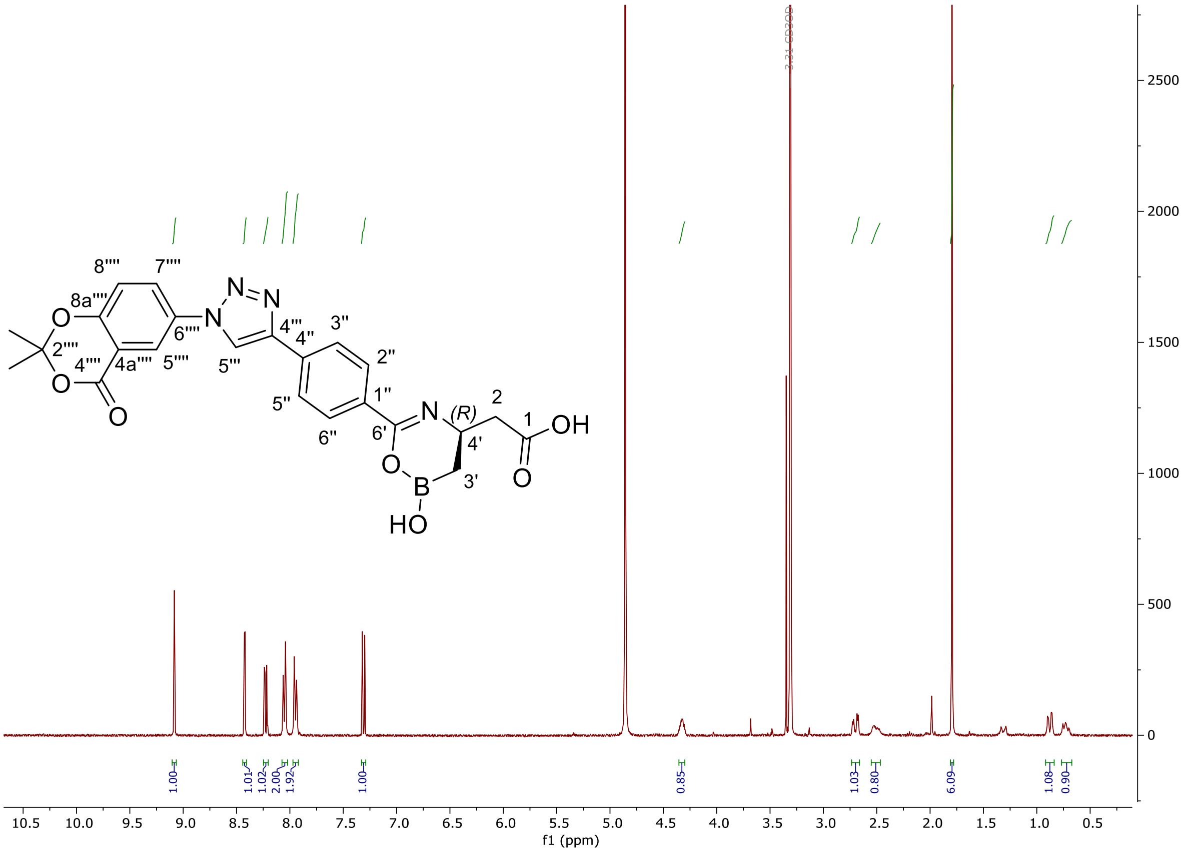

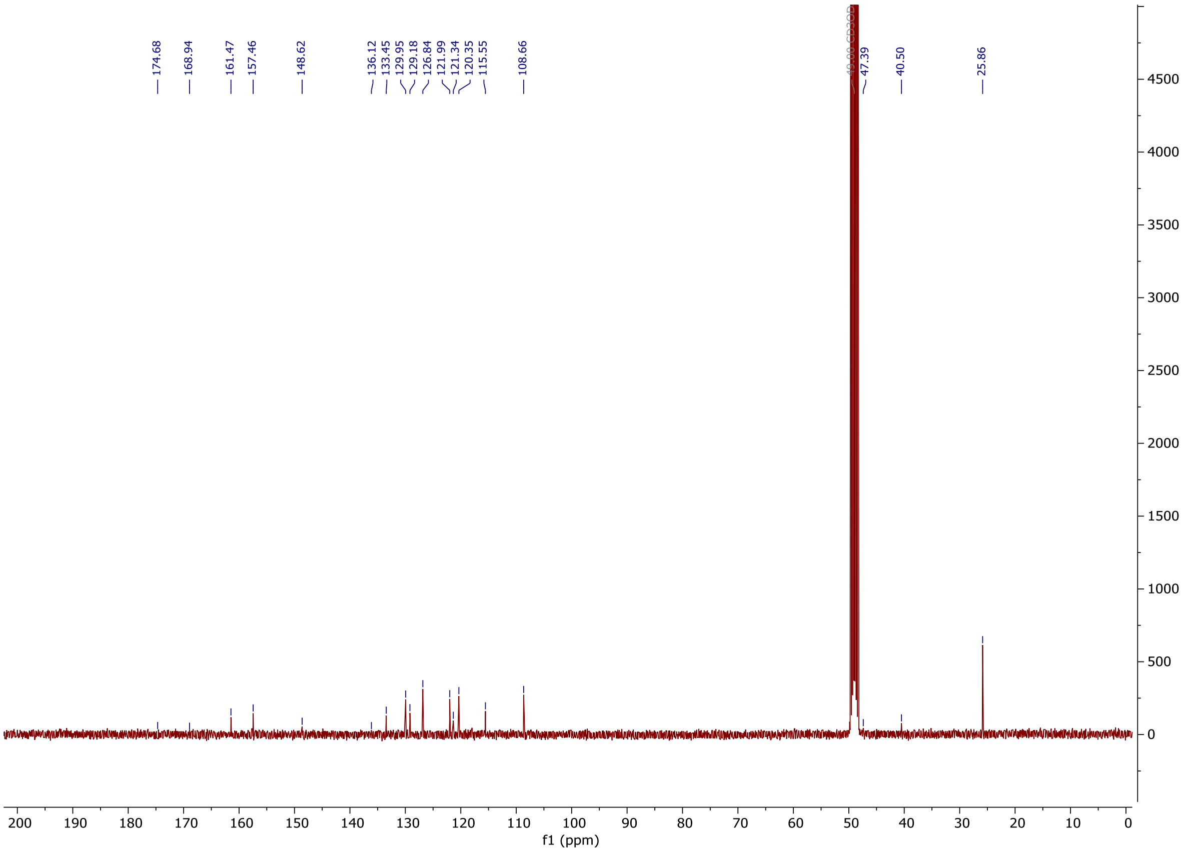

^{1}H and ^{13}C NMR spectra of (*S*)-2-(6-(4-(1-(2,2-dimethyl-4-oxo-4*H*-benzo[*d*][1,3]dioxin-6-yl)-1*H*-1,2,3-triazol-4-yl)phenyl)-2-hydroxy-3,4-dihydro-2*H*-1,5,2-oxazaborinin-4-yl)acetic acid (**54**).

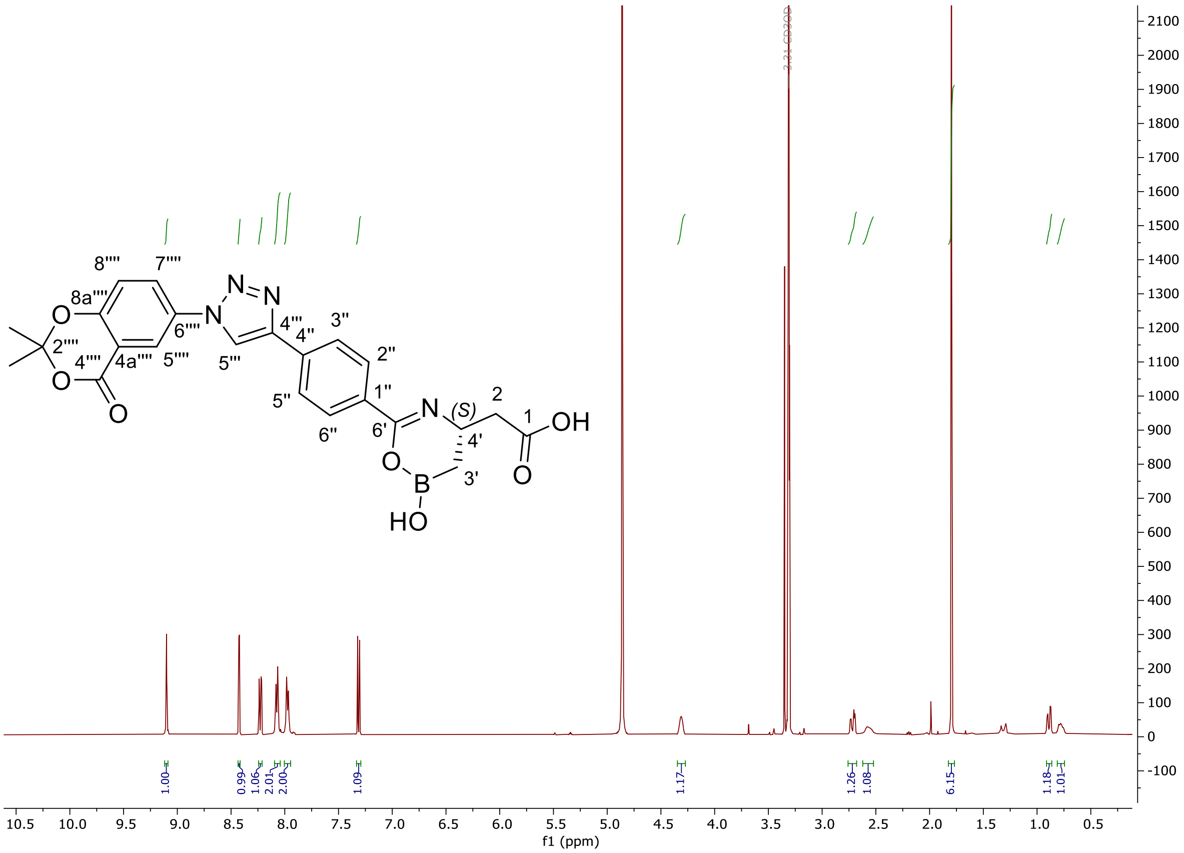

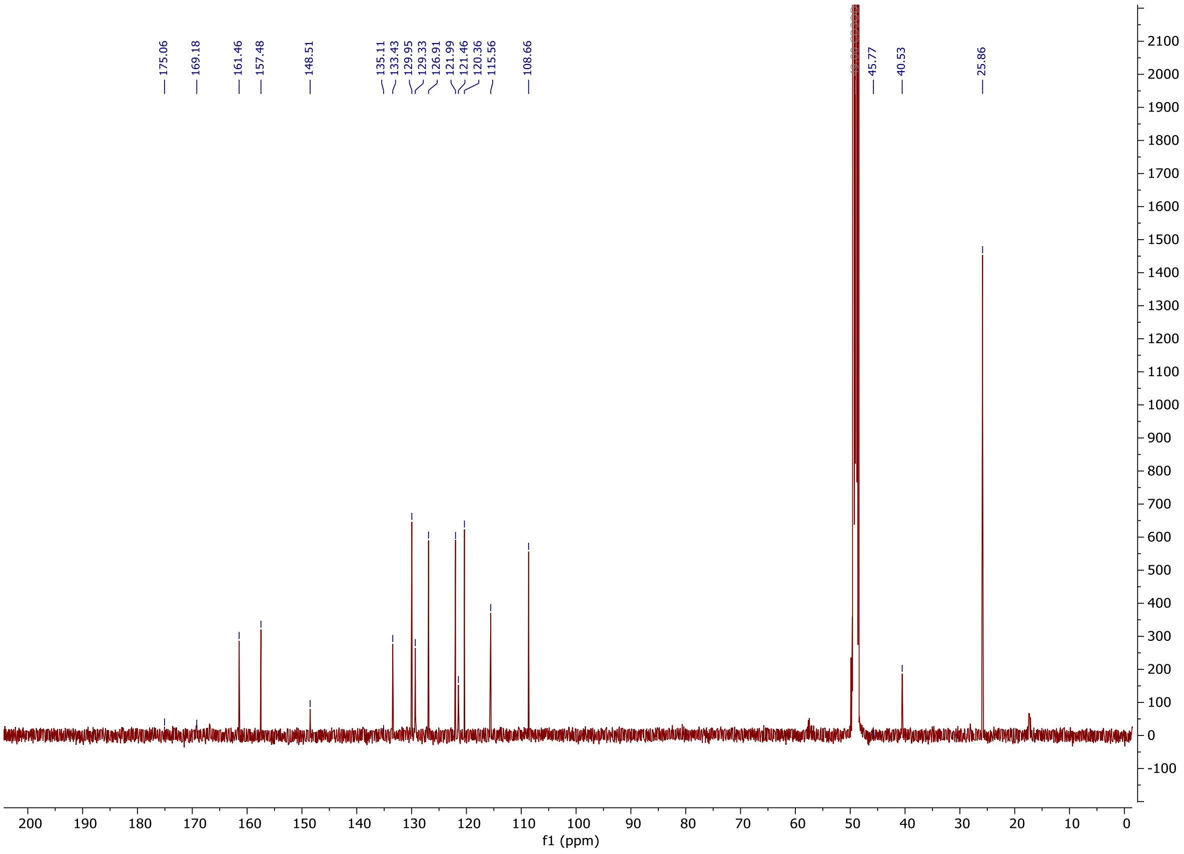

^{1}H and ^{13}C NMR spectra of 1-(benzyloxy)-1-oxohex-5-en-3-aminium (**57**).

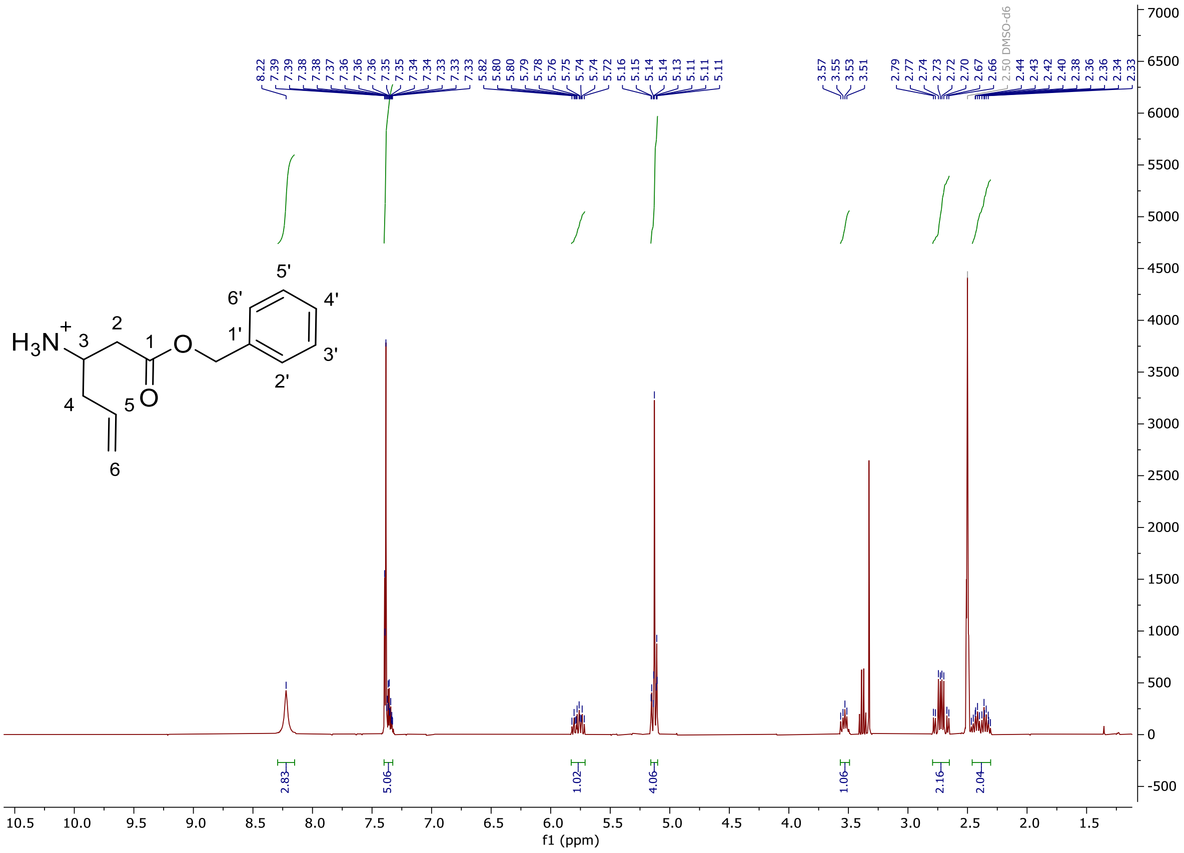

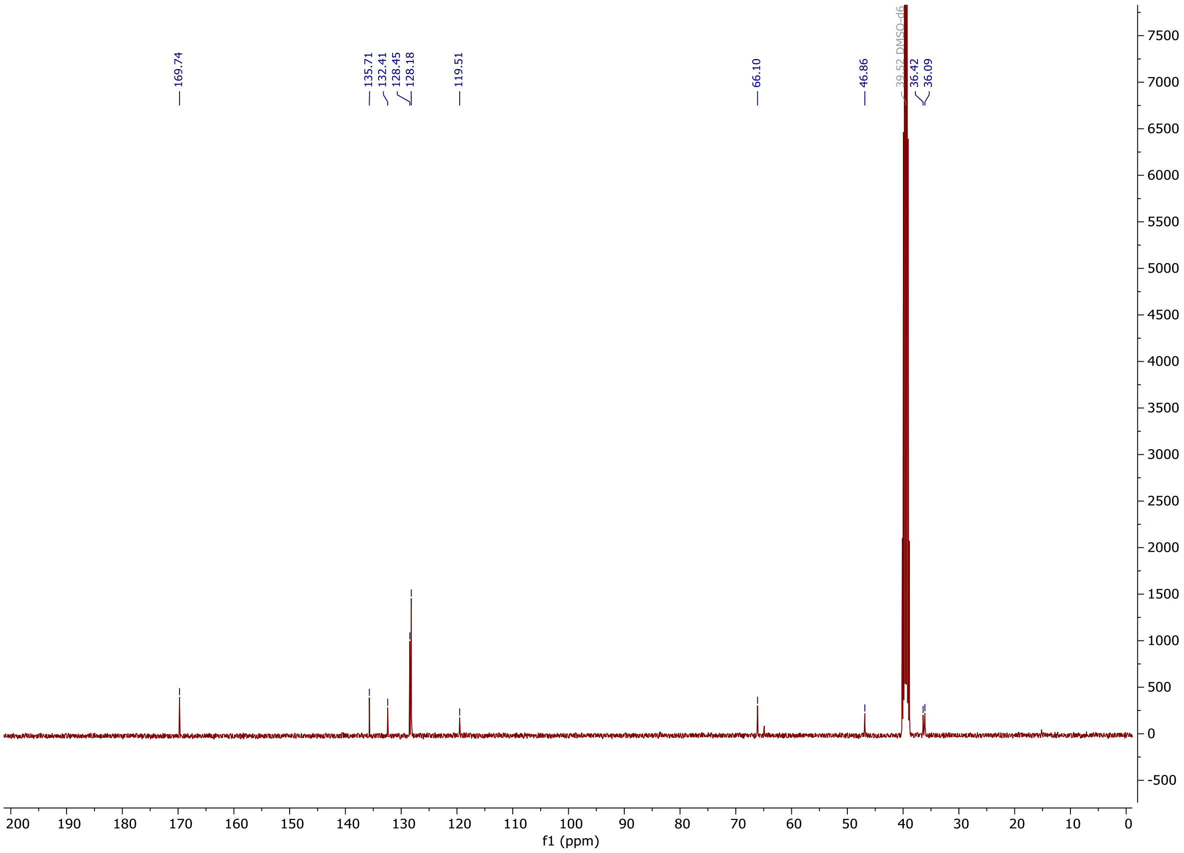

^{1}H and ^{13}C NMR spectra of benzyl 3-(4-ethynylbenzamido)hex-5-enoate (**58**).

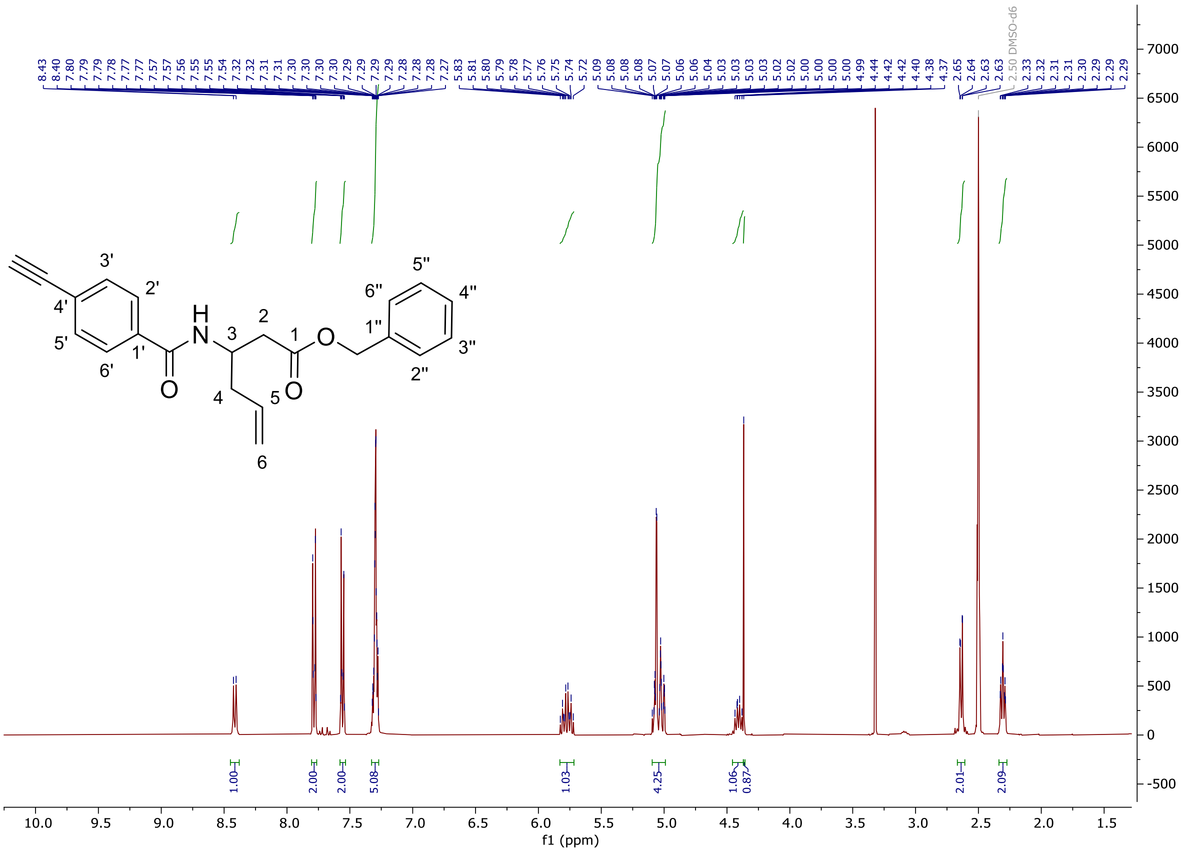

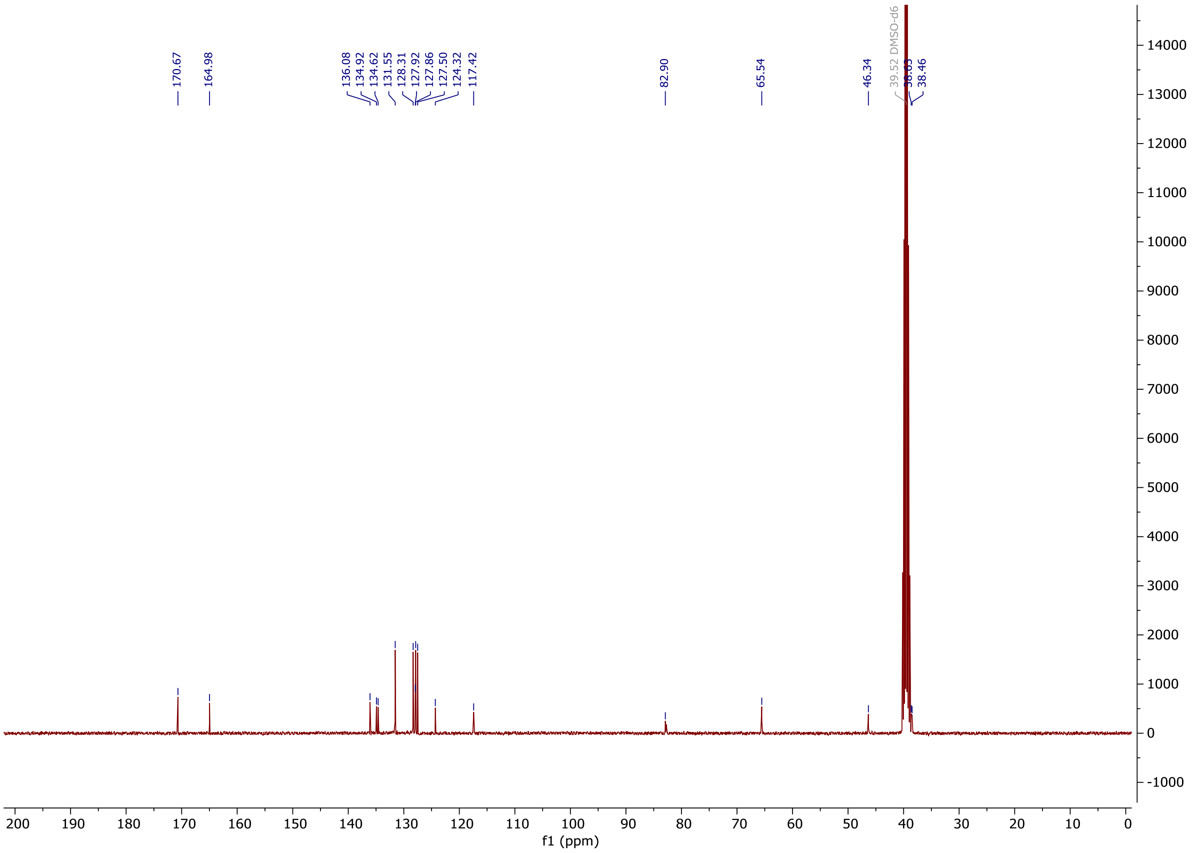

^{1}H and ^{13}C NMR spectra of benzyl 3-(4-(3-(2,2-dimethyl-4-oxo-4*H*-benzo[*d*][1,3]dioxin-6-yl)isoxazol-5-yl)benzamido)hex-5-enoate (**59**).

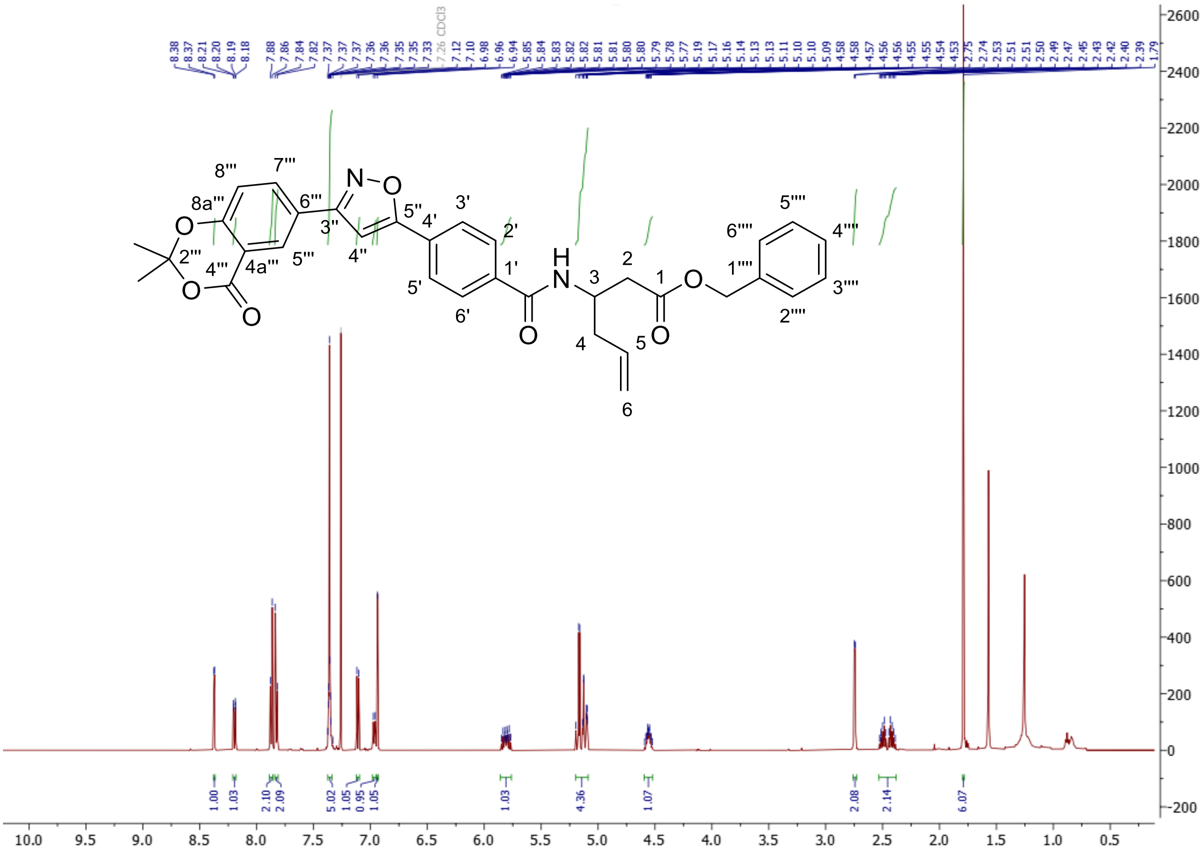

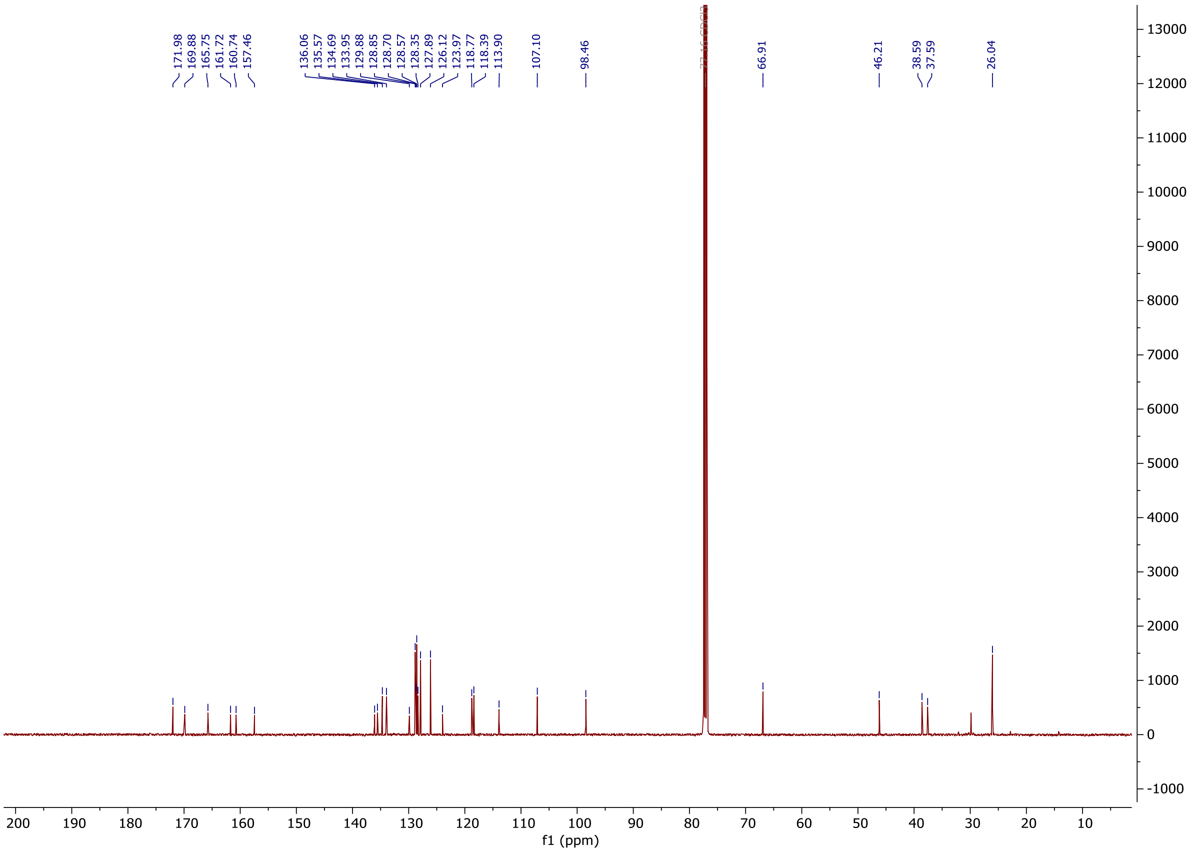

^{1}H and ^{13}C NMR spectra of 5-(5-(4-((1-carboxypent-4-en-2-yl)carbamoyl)phenyl)isoxazol-3-yl)-2-hydroxybenzoic acid (**60**).

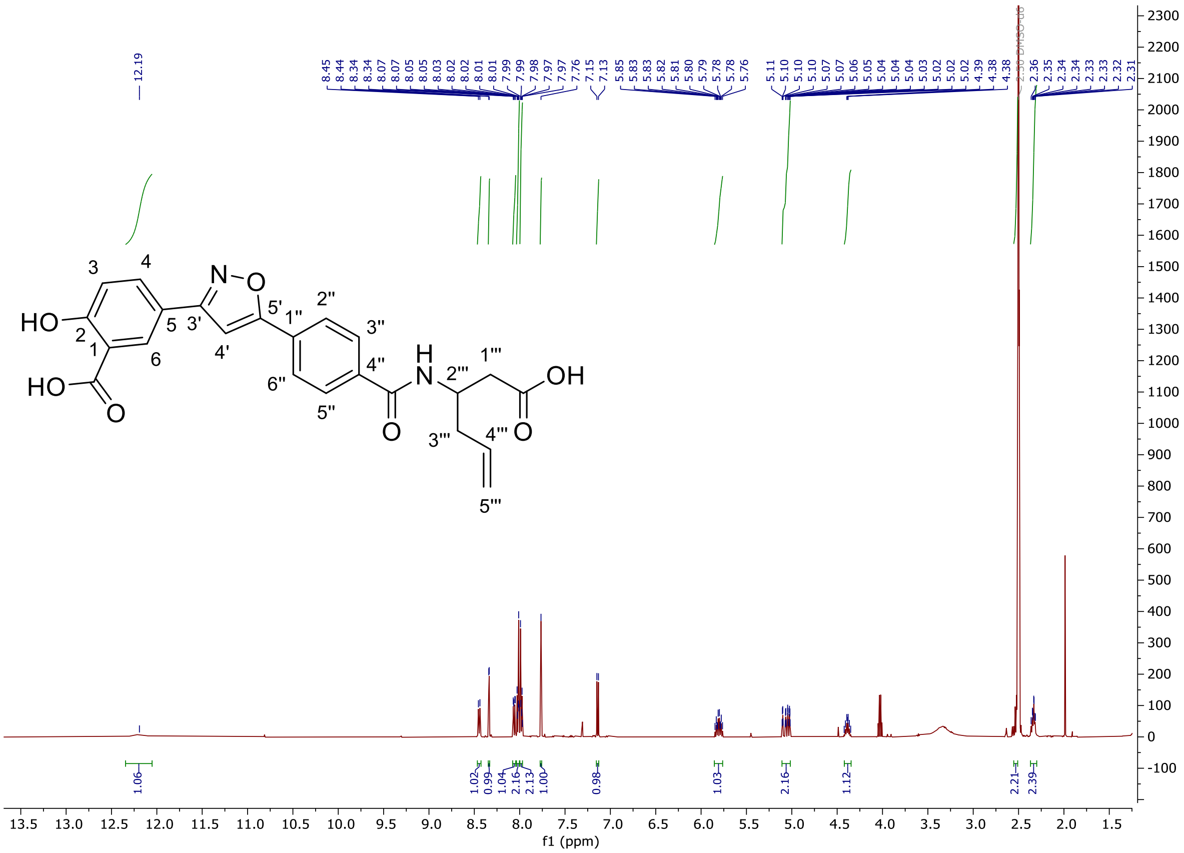

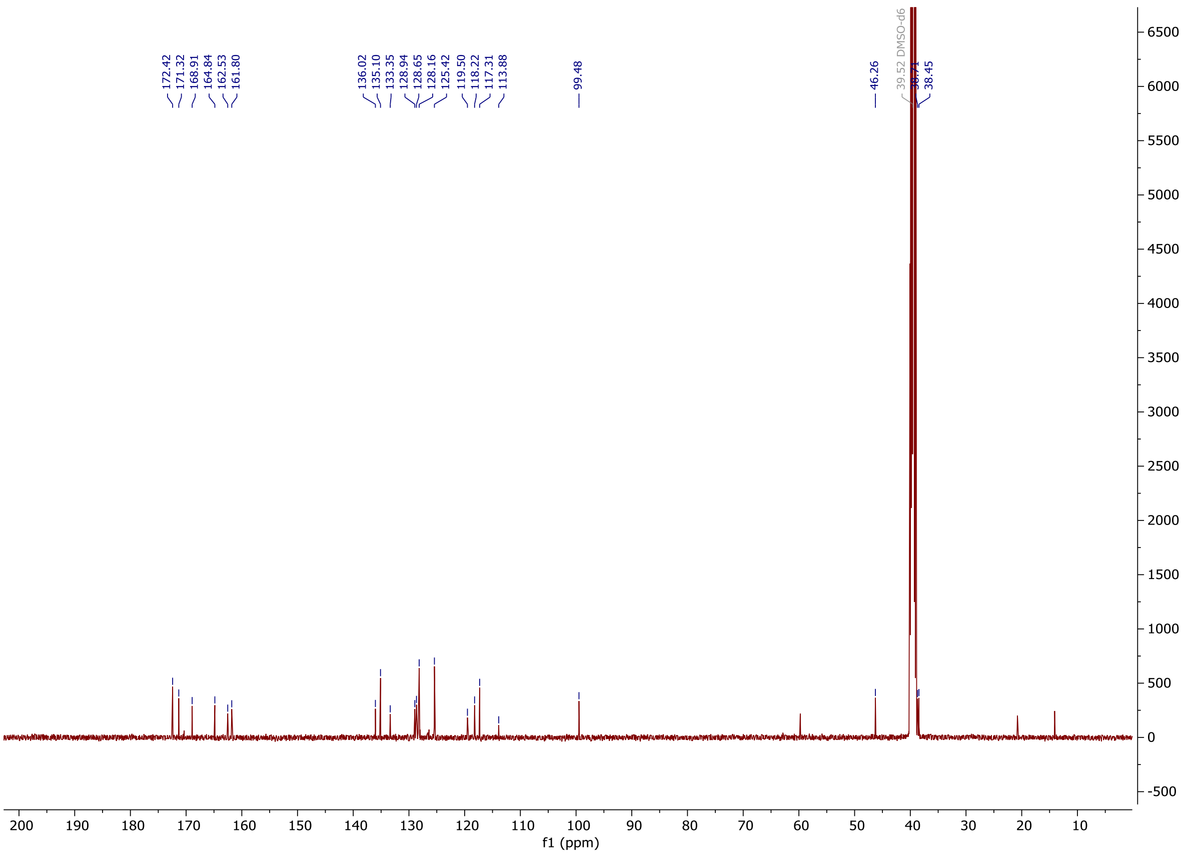

¹H and ¹³C NMR spectra of 5-(5-(4-((1-carboxy-4-oxobutan-2-yl)carbamoyl)phenyl)isoxazol-3-yl)-2-hydroxybenzoic acid (**61**).

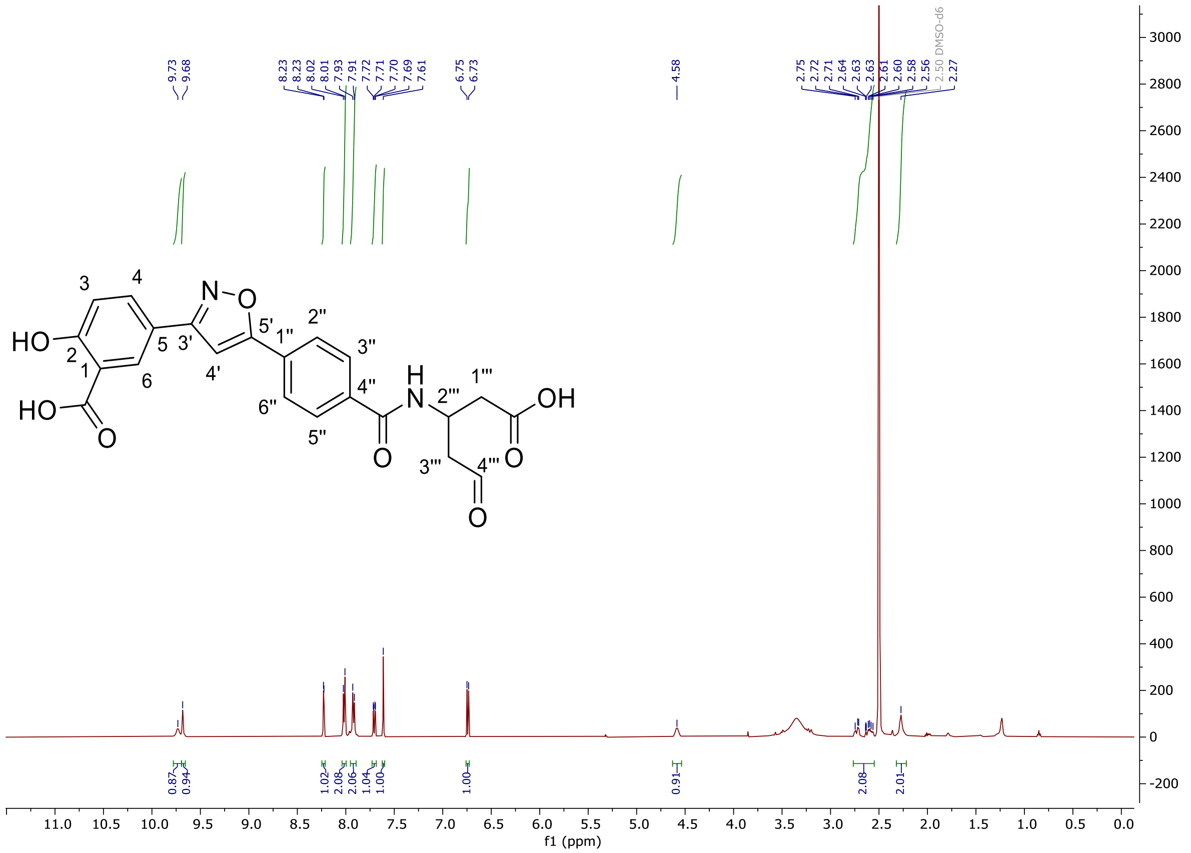

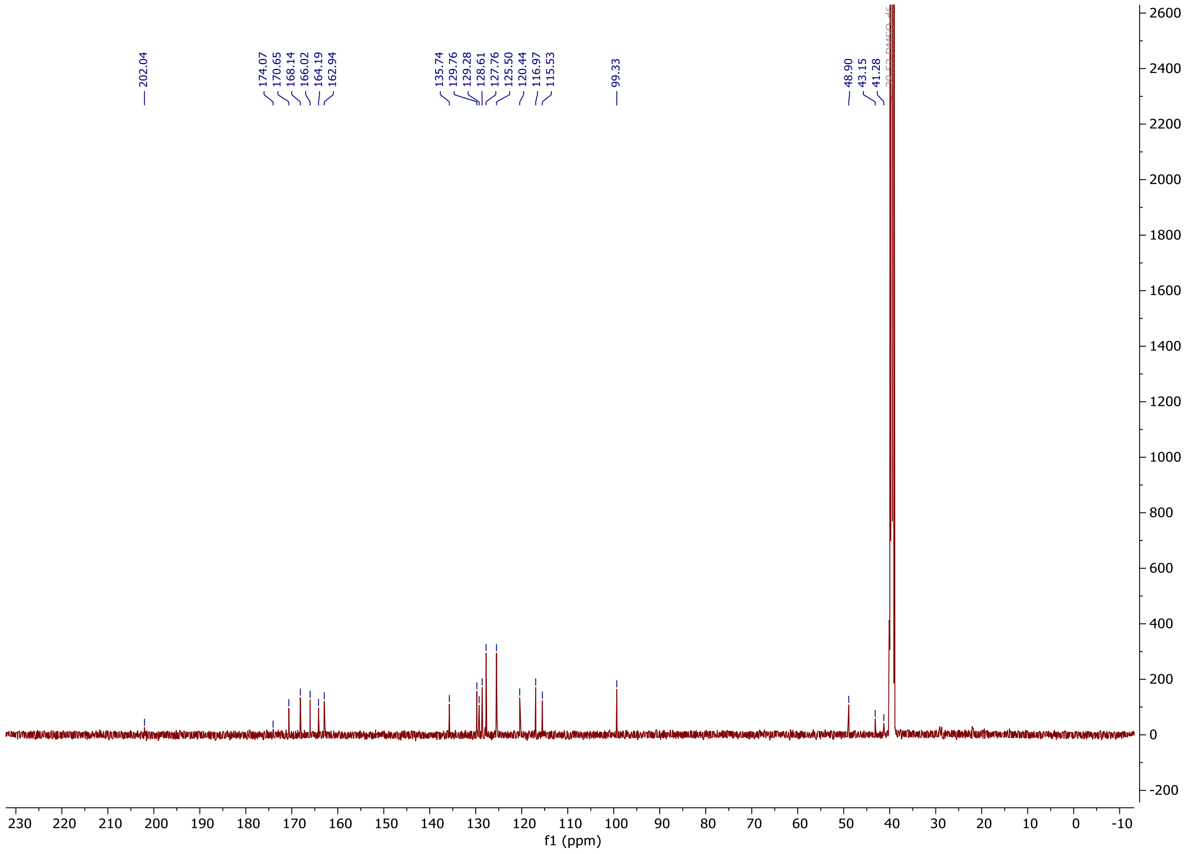

^{1}H and ^{13}C NMR spectra of benzyl 3-(4-(1-(2,2-dimethyl-4-oxo-4*H*-benzo[*d*][1,3]dioxin-6-yl)-1*H*-1,2,3-triazol-4-yl)benzamido)hex-5-enoate (**62**).

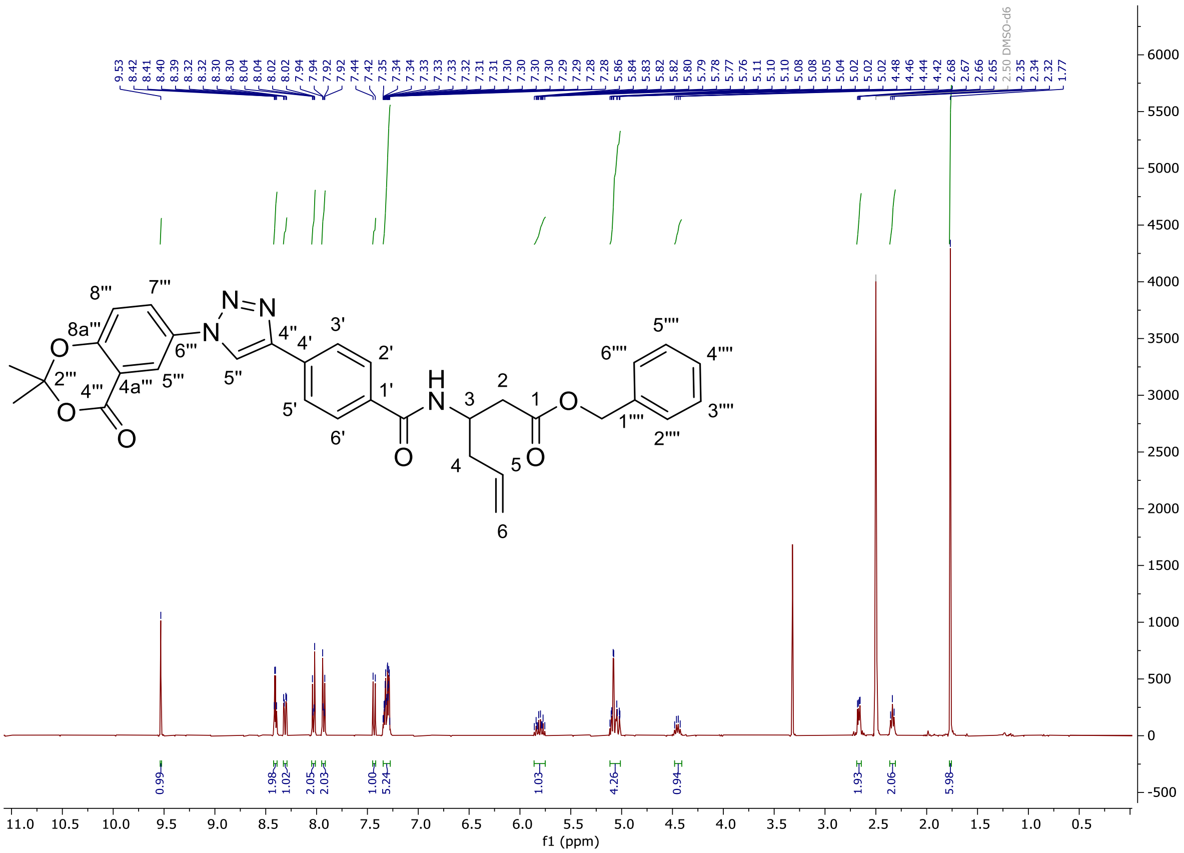

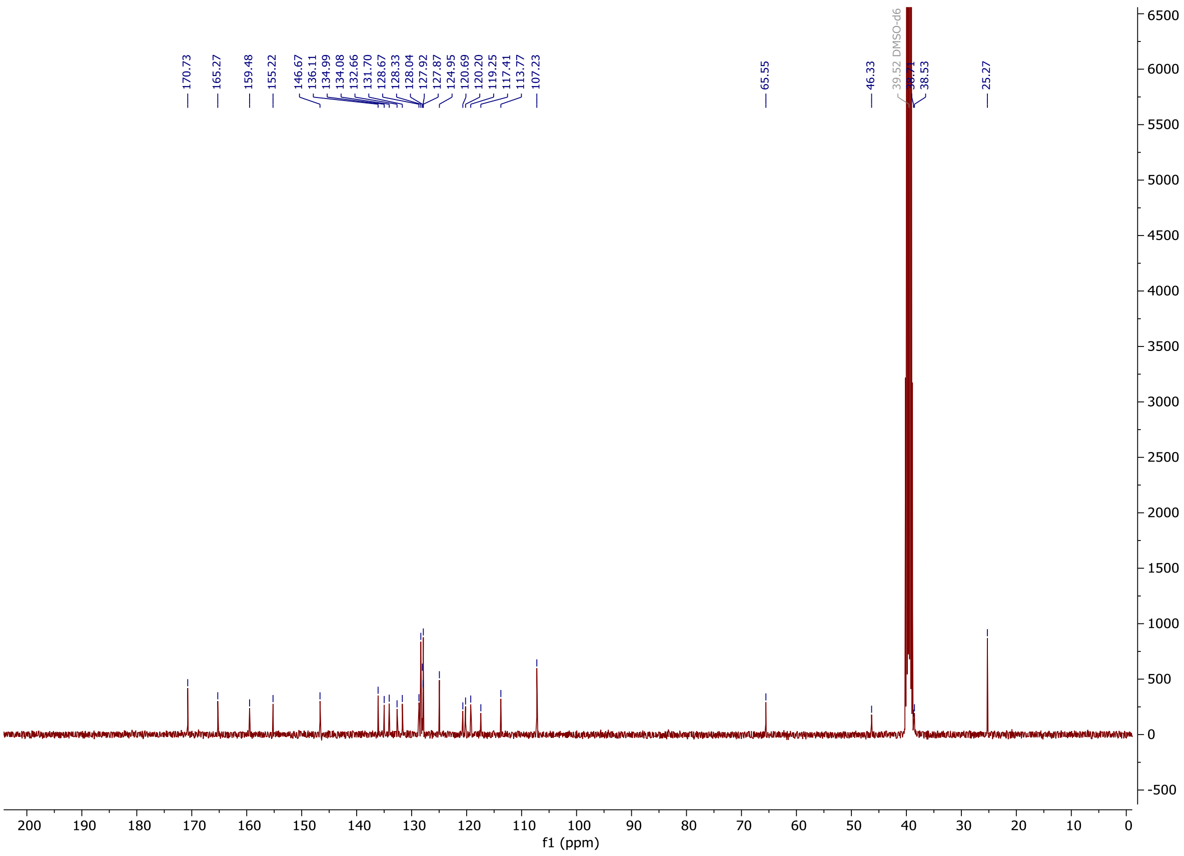

^{1}H and ^{13}C NMR spectra of 5-(4-(4-((1-carboxypent-4-en-2-yl)carbamoyl)phenyl)-1*H*-1,2,3-triazol-1-yl)-2-hydroxybenzoic acid (**63**).

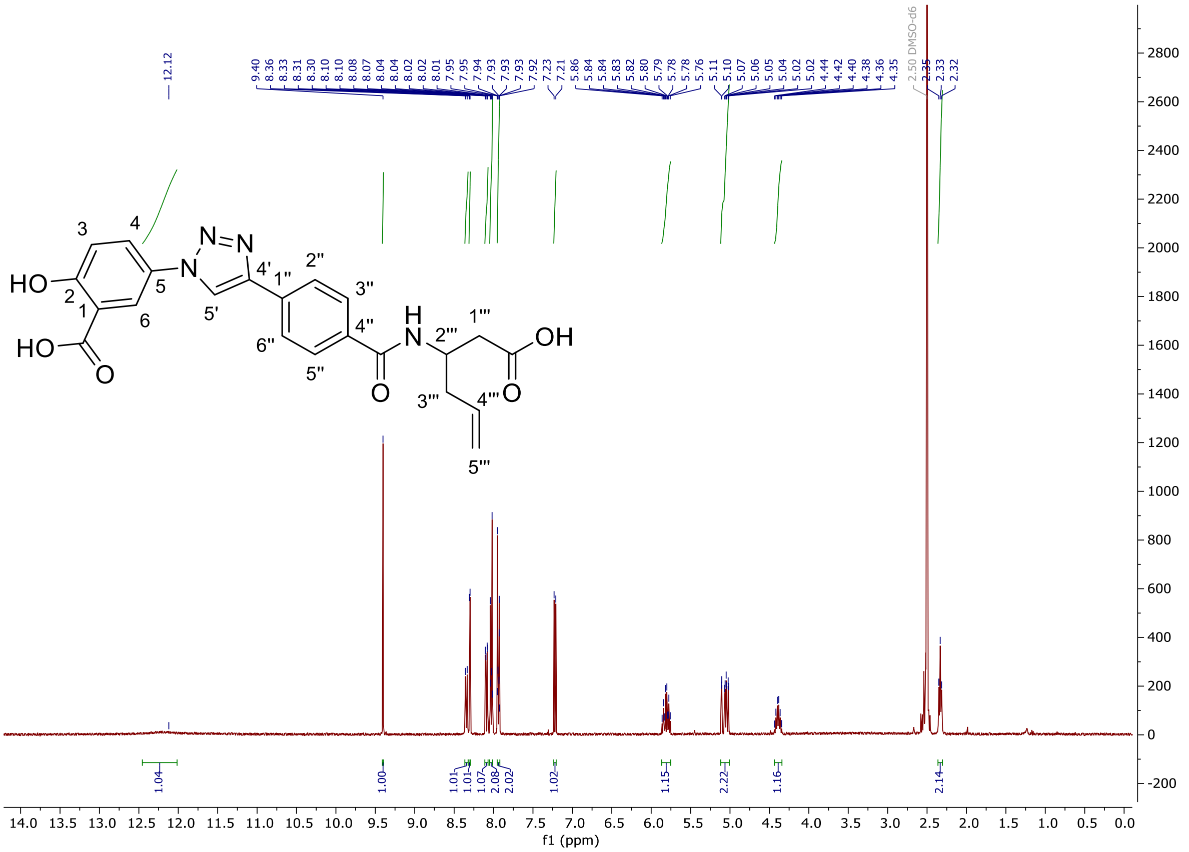

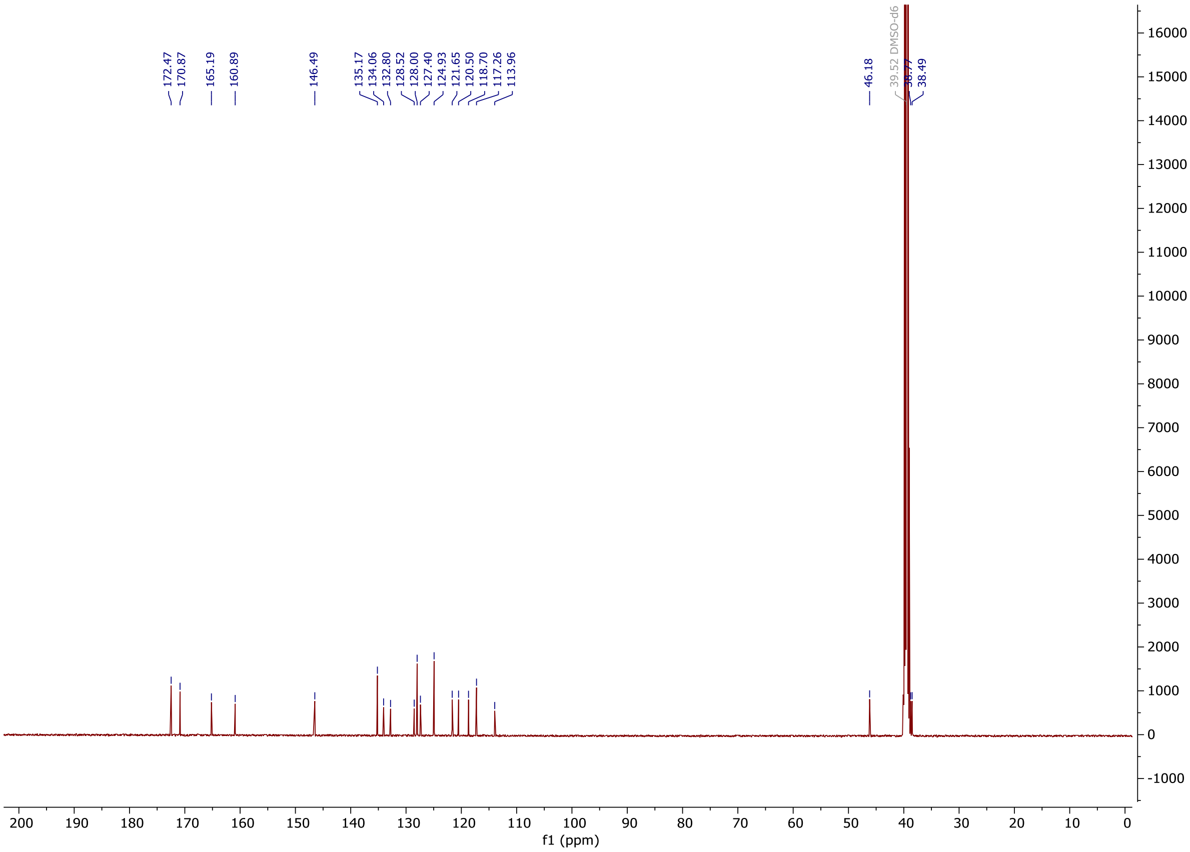

^{1}H and ^{13}C NMR spectra of 5-(4-(4-((1-carboxy-4-oxobutan-2-yl)carbamoyl)phenyl)-1*H*-1,2,3-triazol-1-yl)-2-hydroxybenzoic acid (**64**).

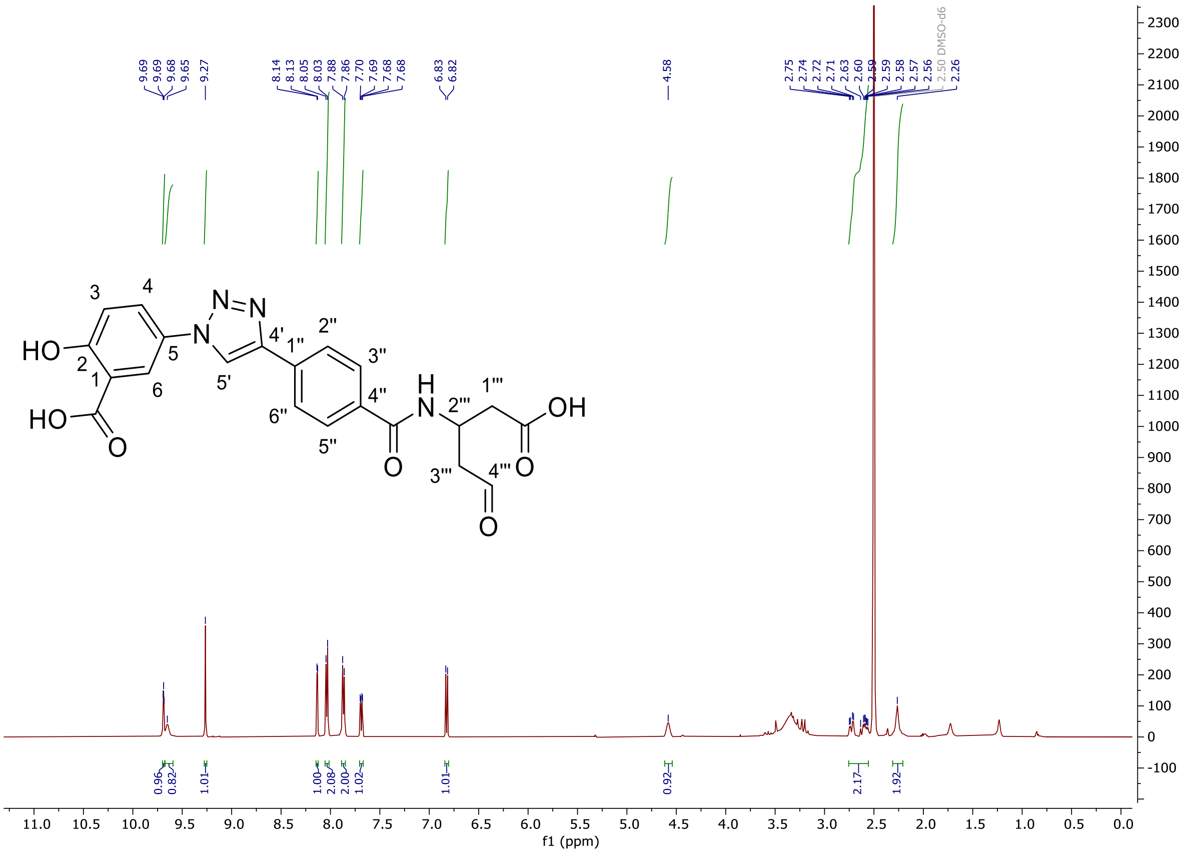

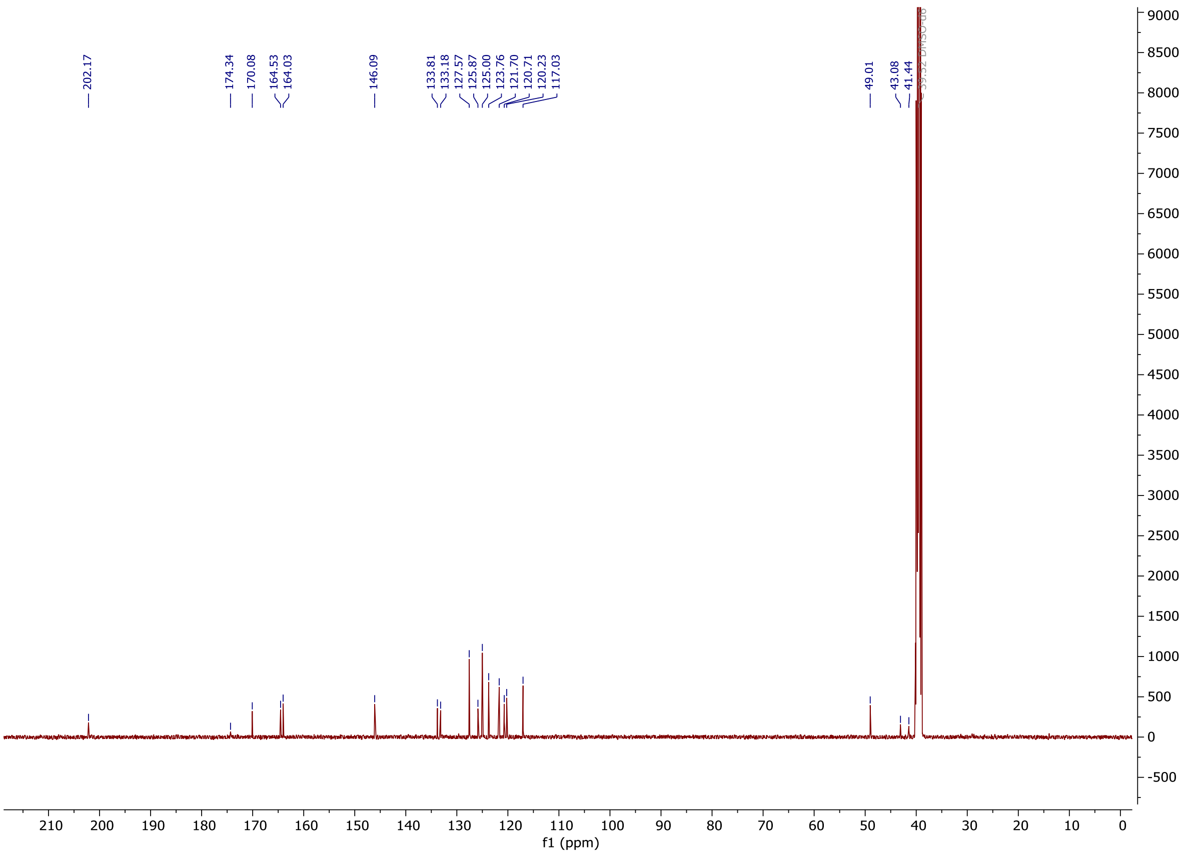

S2. HPLC chromatograms of tested compounds

HPLC chromatrogram of (*R*)-5-(5-(4-((1-carboxy-3-cyanopropan-2-yl)carbamoyl)phenyl)isoxazol-3-yl)-2-hydroxybenzoic acid (**47**).

Chromatogram and Results

Injection Details

Injection Name:	**RW-SAH-11**	*Run Time (min):*	**14,99**
Vial Number:	**Vial:13**	*Injection Volume:*	**5 µL**
Injection Type:	**Unknown**	*Wavelength:*	**210 nm**
		Wavelength:	**254 nm**
Instrument Method:	**Trudel Gradient MeOH Phosphatpuffer pH 5**	*Flow rate:*	**1,0 ml/min**
Column:	**Zorbax Eclipe Plus C18 4,6 x 150mm 3,5µm**	*Column Temperatur:*	**50 °C**
Injection Date/Time:	**21.Jan.25 12:15**	*Pump Channel A:*	**5**
		Pump Channel B:	**95,0 Puffer pH 5**

Chromatogram

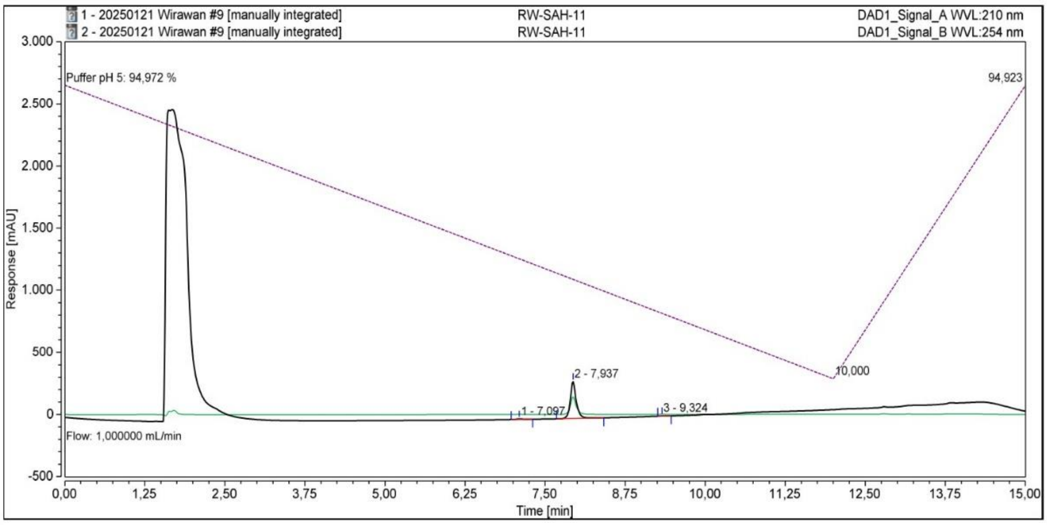

DAD1_Signal_A

No.	Retention Time min	Area mAU*min	Relative Area %
1	7,097	0,410	1,24
2	7,937	32,389	98,15
3	9,324	0,200	0,61
**Total:**		**32,999**	**100**

DAD1_Signal_B

No.	Retention Time min	Area mAU*min	Relative Area %
1	7,097	0,201	1,28
2	7,937	15,089	96,13
3	9,317	0,113	0,72
4	12,791	0,294	1,87
**Total:**		**15,697**	**100**

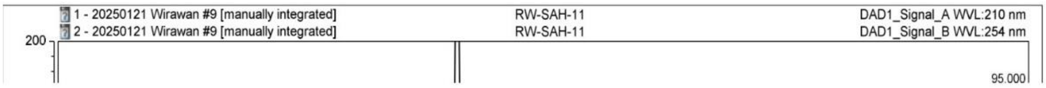

HPLC chromatrogram of (*S*)-5-(5-(4-((1-carboxy-3-cyanopropan-2-yl)carbamoyl)phenyl)isoxazol-3-yl)-2-hydroxybenzoic acid (**48**).

Chromatogram and Results

Injection Details

Injection Name:	**RW-SAH-18**	*Run Time (min):*	**14,99**
Vial Number:	**Vial:14**	*Injection Volume:*	**5 µL**
Injection Type:	**Unknown**	*Wavelength:*	**210 nm**
		Wavelength:	**254 nm**
Instrument Method:	**Trudel Gradient MeOH Phosphatpuffer pH 5**	*Flow rate:*	**1,0 ml/min**
Column:	**Zorbax Eclipe Plus C18 4,6 x 150mm 3,5µm**	*Column Temperatur:*	**50 °C**
Injection Date/Time:	**21.Jan.25 12:31**	*Pump Channel A:*	**5**
		Pump Channel B:	**95,0 Puffer pH 5**

Chromatogram

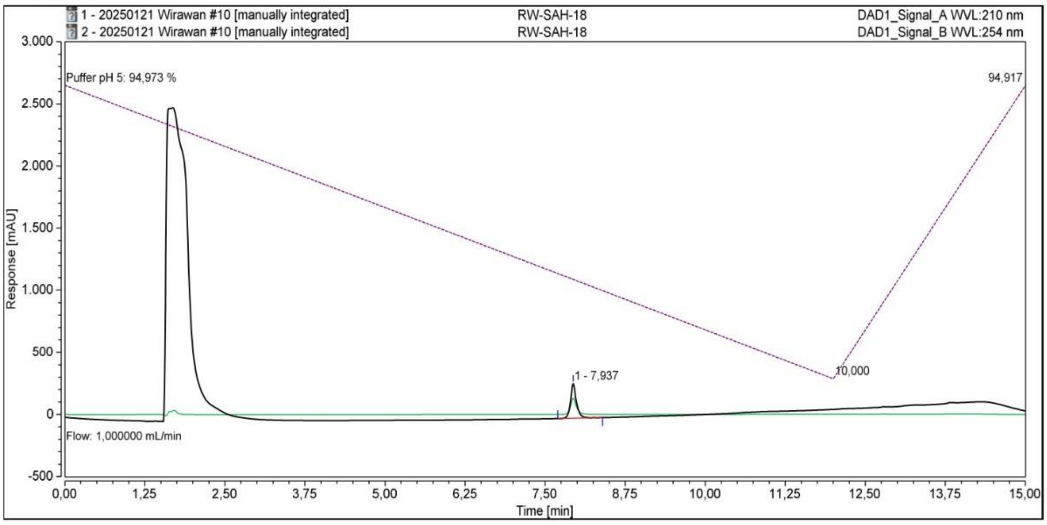

DAD1_Signal_A

No.	Retention Time min	Area mAU*min	Relative Area %
1	7,937	29,993	100,00
**Total:**		**29,993**	**100**

DAD1_Signal_B

No.	Retention Time min	Area mAU*min	Relative Area %
1	7,937	14,336	98,77
2	12,784	0,179	1,23
**Total:**		**14,515**	**100**

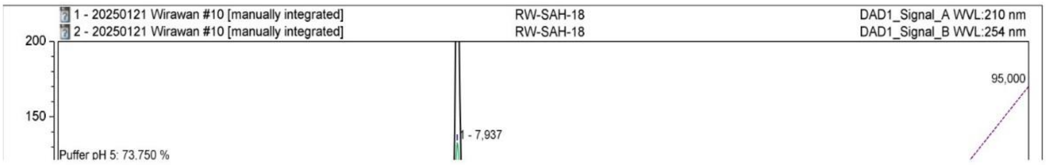

HPLC chromatrogram of (*R*)-5-(4-(4-((1-carboxy-3-cyanopropan-2-yl)carbamoyl)phenyl)-1*H*-1,2,3-triazol-1-yl)-2-hydroxybenzoic acid (**49**).

Chromatogram and Results

Injection Details

Injection Name:	**RW-183**	*Run Time (min):*	**14,99**
Vial Number:	**Vial:19**	*Injection Volume:*	**5 µL**
Injection Type:	**Unknown**	*Wavelength:*	**210 nm**
		Wavelength:	**254 nm**
Instrument Method:	**Trudel Gradient MeOH Phosphatpuffer pH 5**	*Flow rate:*	**1,0 ml/min**
Column:	**Zorbax Eclipe Plus C18 4,6 x 150mm 3,5µm**	*Column Temperatur:*	**50 °C**
Injection Date/Time:	**21.Jan.25 13:49**	*Pump Channel A:*	**5**
		Pump Channel B:	**95,0 Puffer pH 5**

Chromatogram

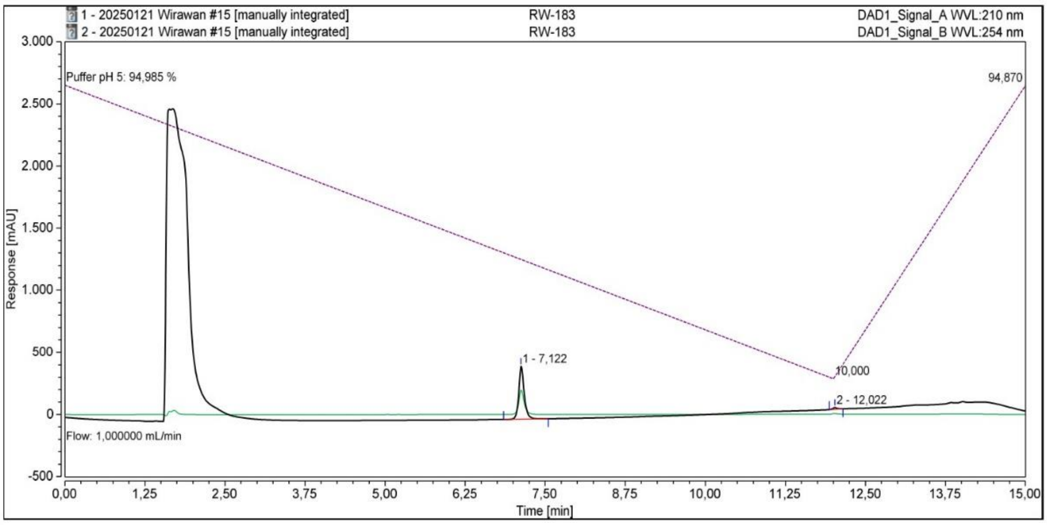

DAD1_Signal_A

No.	Retention Time min	Area mAU*min	Relative Area %
1	7,122	41,887	97,52
2	12,022	1,067	2,48
**Total:**		**42,953**	**100**

DAD1_Signal_B

No.	Retention Time min	Area mAU*min	Relative Area %
1	7,122	19,353	97,14
2	12,022	0,569	2,86
**Total:**		**19,922**	**100**

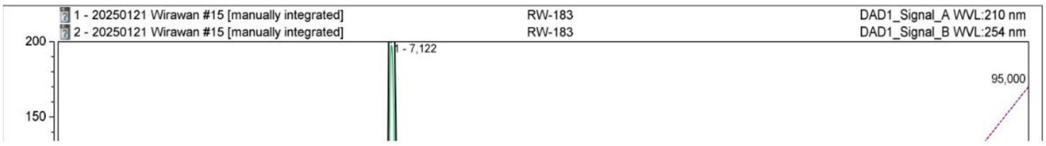

Reinheit Trudel/Integration

Chromeleon (c) Dionex Version 7.2.9.11323

HPLC chromatrogram of (*S*)-5-(4-(4-((1-carboxy-3-cyanopropan-2-yl)carbamoyl)phenyl)-1*H*-1,2,3-triazol-1-yl)-2-hydroxybenzoic acid (**50**).

Chromatogram and Results

Injection Details

Injection Name:	**RW-191**	*Run Time (min):*	**14,99**
Vial Number:	**Vial:20**	*Injection Volume:*	**5 µL**
Injection Type:	**Unknown**	*Wavelength:*	**210 nm**
		Wavelength:	**254 nm**
Instrument Method:	**Trudel Gradient MeOH Phosphatpuffer pH 5**	*Flow rate:*	**1,0 ml/min**
Column:	**Zorbax Eclipe Plus C18 4,6 x 150mm 3,5µm**	*Column Temperatur:*	**50 °C**
Injection Date/Time:	**21.Jan.25 14:05**	*Pump Channel A:*	**5**
		Pump Channel B:	**95,0 Puffer pH 5**

Chromatogram

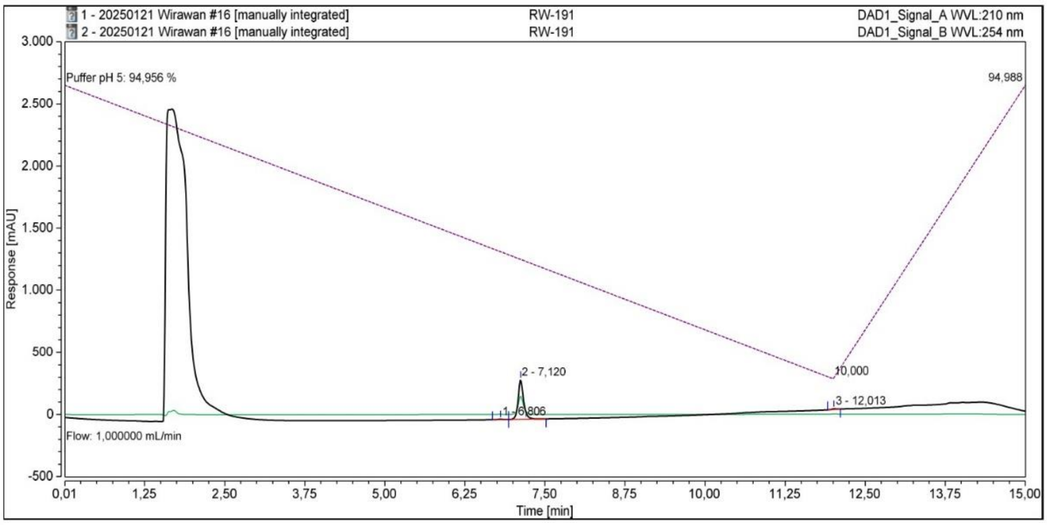

DAD1_Signal_A

No.	Retention Time min	Area mAU*min	Relative Area %
1	6,806	0,292	0,91
2	7,120	31,161	97,67
3	12,013	0,453	1,42
**Total:**		**31,906**	**100**

DAD1_Signal_B

No.	Retention Time min	Area mAU*min	Relative Area %
1	6,800	0,148	1,00
2	7,120	14,461	97,00
3	12,013	0,298	2,00
**Total:**		**14,907**	**100**

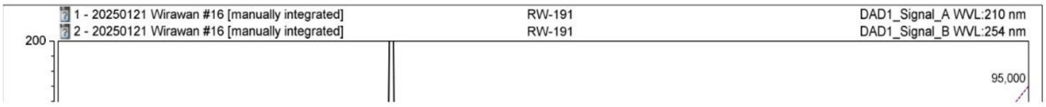

HPLC chromatrogram of (*R*)-2-(6-(4-(3-(2,2-dimethyl-4-oxo-4*H*-benzo[*d*][1,3]dioxin-6-yl)isoxazol-5-yl)phenyl)-2-hydroxy-3,4-dihydro-2*H*-1,5,2-oxazaborinin-4-yl)acetic acid (**51**).

Instrument:Trudel Sequence:20250122 Wirawan 3 Page 1 of 2

Chromatogram and Results

Injection Details

Injection Name:	**RW-136 Stock mit Wasser**	*Run Time (min):*	**11,99**
Vial Number:	**Vial:31**	*Injection Volume:*	**5 µL**
Injection Type:	**Unknown**	*Wavelength:*	**210 nm**
		Wavelength:	**254 nm**
Instrument Method:	**MeOH Wasser +0,1% TFA Gradient 2**	*Flow rate:*	**1,0 ml/min**
Column:	**Zorbax Eclipe Plus C18 4,6 x 150mm 3,5µm**	*Column Temperatur:*	**50 °C**
Injection Date/Time:	**24.Jan.25 11:08**	*Pump Channel A:*	**10**
		Pump Channel B:	**90,0 Wasser + 0,1% TFA**

Chromatogram

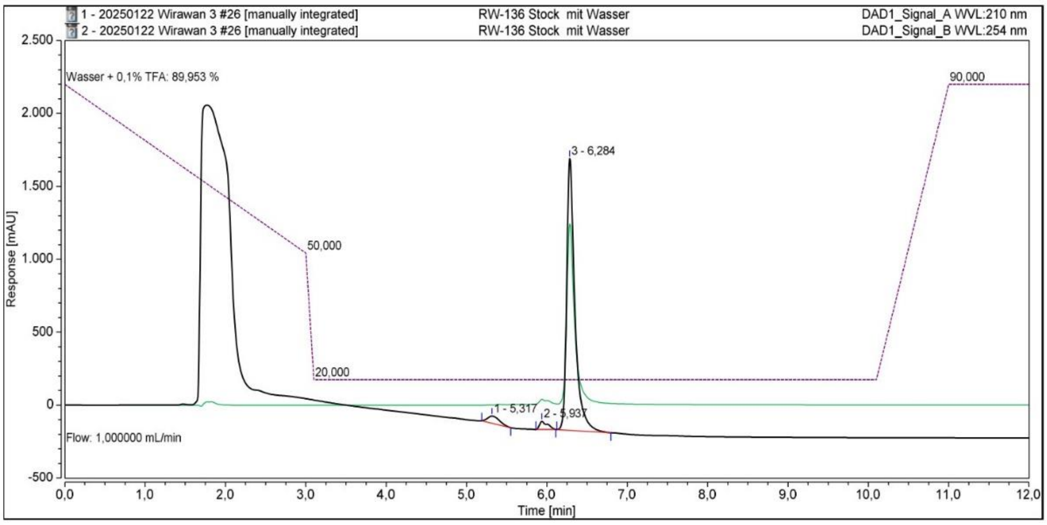

DAD1_Signal_A

No.	Retention Time min	Area mAU*min	Relative Area %
1	5,317	8,482	3,60
2	5,937	6,420	2,72
3	6,284	220,737	93,68
**Total:**		**235,639**	**100**

DAD1_Signal_B

No.	Retention Time min	Area mAU*min	Relative Area %
1	5,937	4,144	2,82
2	6,284	142,868	97,18
**Total:**		**147,012**	**100**

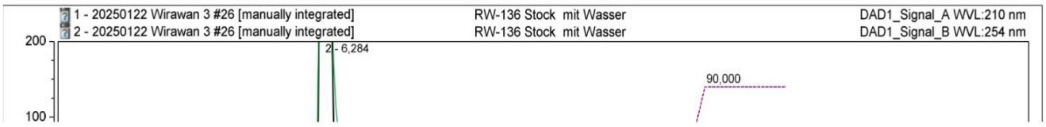

Reinheit Trudel/Integration

Chromeleon (c) Dionex
Version 7.2.9.11323

HPLC chromatrogram of (*S*)-2-(6-(4-(3-(2,2-dimethyl-4-oxo-4*H*-benzo[*d*][1,3]dioxin-6-yl)isoxazol-5-yl)phenyl)-2-hydroxy-3,4-dihydro-2*H*-1,5,2-oxazaborinin-4-yl)acetic acid (**52**).

Chromatogram and Results

Injection Details

Injection Name:	**RW-155-2**	*Run Time (min):*	**11,09**
Vial Number:	**Vial:92**	*Injection Volume:*	**2,00**
Injection Type:	**Unknown**	*Wavelength A:*	**210**
Column:	**Eclipse Plus C18 3,5µm 4,6x 150mm 959963-902**	*Wavelength B:*	**254**
Instrument Method:	**Methanol Wassser 0,1% TFA Gradient 2**		
Processing Method:	**SG-094**	*Flow rate:*	**1,000 mL/min**
Injection Date/Time:	**30.Jan.25 15:05**	*Column Temperatu*	**50,0 °C**
Pump Channel A:	**0,00** Acetonitril		
Pump Channel B:	**10** Methanol		
Pump ChannelC:	**90** Wasser 0,1% TFA		
Pump Channel D:	Wasser		

Chromatogram

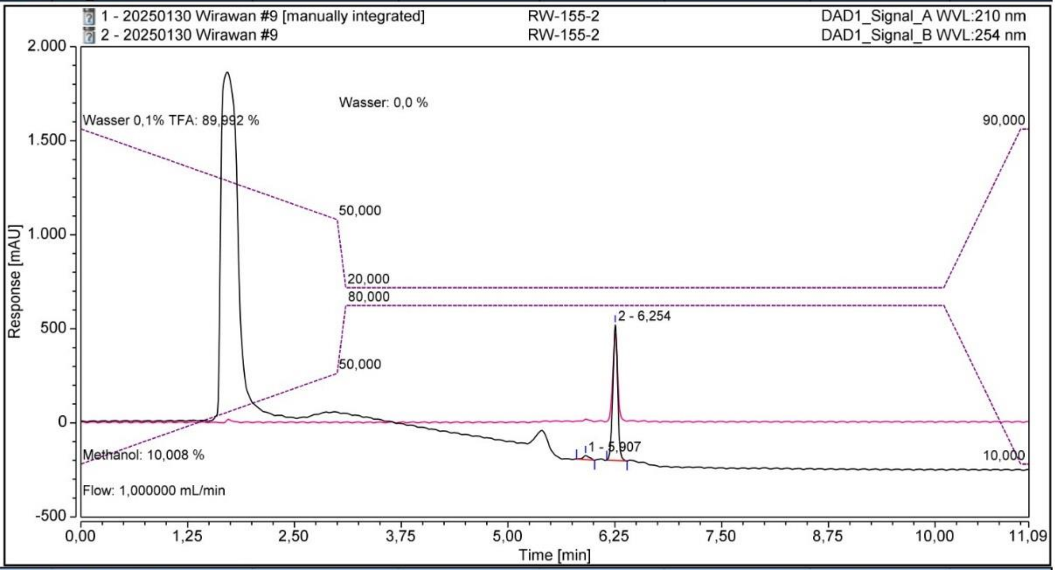

Integration Results

210nm

No.	Peak Name	Retention Time min	Area mAU*min	Relative Area %
1		5,907	1,753	3,6
2		6,254	47,468	96,4
**Total:**			**49,221**	**100,00**

254mn

No.	Peak Name	Retention Time min	Area mAU*min	Relative Area %
n.a.	n.a.	n.a.	n.a.	n.a.
1		6,254	31,159	100,0
**Total:**			**31,159**	**100,00**

Chromeleon (c) Dionex
Version 7.2.9.11323

HPLC chromatrogram of (*R*)-2-(6-(4-(1-(2,2-dimethyl-4-oxo-4*H*-benzo[*d*][1,3]dioxin-6-yl)-1*H*-1,2,3-triazol-4-yl)phenyl)-2-hydroxy-3,4-dihydro-2*H*-1,5,2-oxazaborinin-4-yl)acetic acid (**53**).

Instrument:Trudel Sequence:20250122 Wirawan 3 Page 1 of 2

Chromatogram and Results

Injection Details

Injection Name:	**RW-186 mit Wasser**	*Run Time (min):*	**11,99**
Vial Number:	**Vial:21**	*Injection Volume:*	**5 µL**
Injection Type:	**Unknown**	*Wavelength:*	**210 nm**
		Wavelength:	**254 nm**
Instrument Method:	**50 MeOH 50 Wasser +0,1% TFA**	*Flow rate:*	**1,0 ml/min**
Column:	**Zorbax Eclipe Plus C18 4,6 x 150mm 3,5µm**	*Column Temperatur:*	**50 °C**
Injection Date/Time:	**23.Jan.25 13:30**	*Pump Channel A:*	**50**
		Pump Channel B:	**50,0 Wasser + 0,1% TFA**

Chromatogram

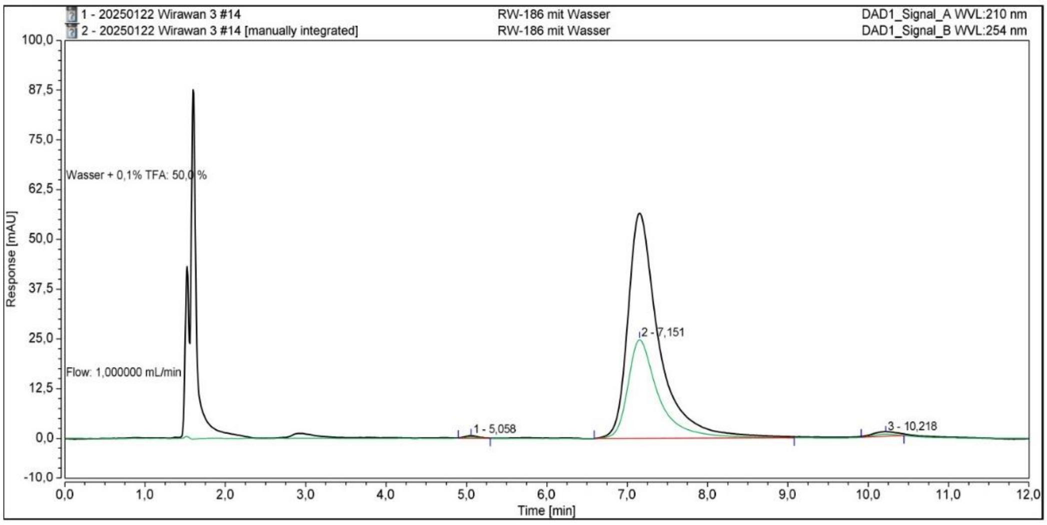

DAD1_Signal_A

No.	Retention Time min	Area mAU*min	Relative Area %
1	7,151	25,012	100,00
**Total:**		**25,012**	**100**

DAD1_Signal_B

No.	Retention Time min	Area mAU*min	Relative Area %
1	5,058	0,054	0,48
2	7,151	10,905	97,99
3	10,218	0,170	1,53
**Total:**		**11,129**	**100**

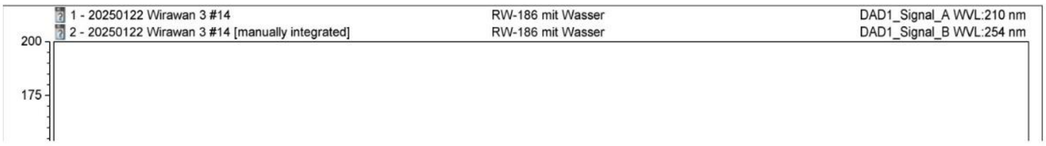

Reinheit Trudel/Integration Chromeleon (c) Dionex Version 7.2.9.11323

HPLC chromatrogram of (*S*)-2-(6-(4-(1-(2,2-dimethyl-4-oxo-4*H*-benzo[*d*][1,3]dioxin-6-yl)-1*H*-1,2,3-triazol-4-yl)phenyl)-2-hydroxy-3,4-dihydro-2*H*-1,5,2-oxazaborinin-4-yl)acetic acid (**54**).

Instrument:Trudel Sequence:20250122 Wirawan 3 — Page 1 of 2

Chromatogram and Results

Injection Details

Injection Name:	**RW-193 mit Wasser**	*Run Time (min):*	**11,99**
Vial Number:	**Vial:22**	*Injection Volume:*	**5 µL**
Injection Type:	**Unknown**	*Wavelength:*	**210 nm**
		Wavelength:	**254 nm**
Instrument Method:	**50 MeOH 50 Wasser +0,1% TFA**	*Flow rate:*	**1,0 ml/min**
Column:	**Zorbax Eclipe Plus C18 4,6 x 150mm 3,5µm**	*Column Temperatur:*	**50 °C**
Injection Date/Time:	**23.Jan.25 13:42**	*Pump Channel A:*	**50**
		Pump Channel B:	**50,0 Wasser + 0,1% TFA**

Chromatogram

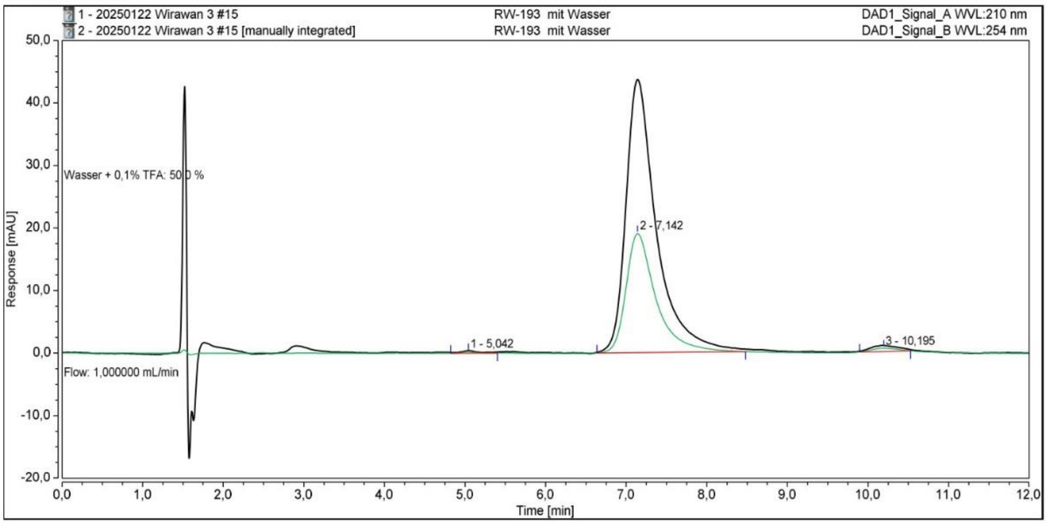

DAD1_Signal_A

No.	Retention Time min	Area mAU*min	Relative Area %
1	7,142	19,382	100,00
**Total:**		**19,382**	**100**

DAD1_Signal_B

No.	Retention Time min	Area mAU*min	Relative Area %
1	5,042	0,042	0,50
2	7,142	8,183	97,33
3	10,195	0,182	2,17
**Total:**		**8,407**	**100**

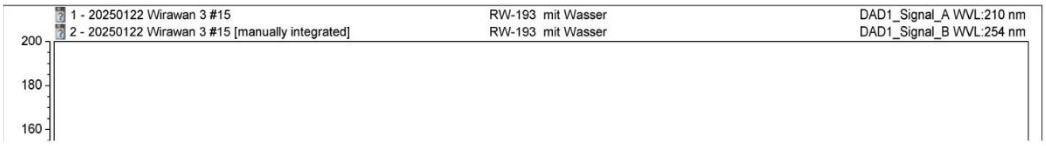

Reinheit Trudel/Integration — Chromeleon (c) Dionex Version 7.2.9.11323

HPLC chromatrogram of 5-(5-(4-((1-carboxy-4-oxobutan-2-yl)carbamoyl)phenyl)isoxazol-3-yl)-2-hydroxybenzoic acid (**61**).

Chromatogram and Results

Injection Details

Injection Name:	**RW-170**	*Run Time (min):*	**14,99**
Vial Number:	**Vial:17**	*Injection Volume:*	**5 µL**
Injection Type:	**Unknown**	*Wavelength:*	**210 nm**
		Wavelength:	**254 nm**
Instrument Method:	**Trudel Gradient MeOH Phosphatpuffer pH 5**	*Flow rate:*	**1,0 ml/min**
Column:	**Zorbax Eclipe Plus C18 4,6 x 150mm 3,5µm**	*Column Temperatur:*	**50 °C**
Injection Date/Time:	**21.Jan.25 13:18**	*Pump Channel A:*	**5**
		Pump Channel B:	**95,0 Puffer pH 5**

Chromatogram

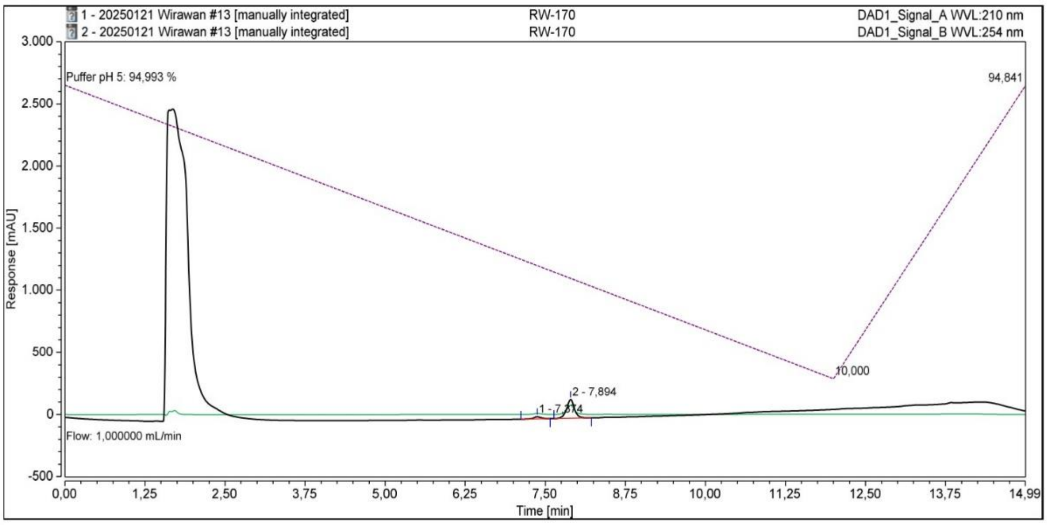

DAD1_Signal_A

No.	Retention Time min	Area mAU*min	Relative Area %
1	7,374	2,295	9,65
2	7,894	21,497	90,35
**Total:**		**23,792**	**100**

DAD1_Signal_B

No.	Retention Time min	Area mAU*min	Relative Area %
1	7,374	1,005	8,71
2	7,894	10,539	91,29
**Total:**		**11,544**	**100**

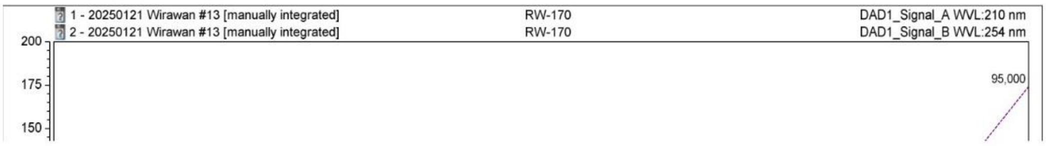

HPLC chromatrogram of 5-(4-(4-((1-carboxy-4-oxobutan-2-yl)carbamoyl)phenyl)-1*H*-1,2,3-triazol-1-yl)-2-hydroxybenzoic acid (**64**).

Chromatogram and Results

Injection Details

Injection Name:	**RW-196**	*Run Time (min):*	**14,99**
Vial Number:	**Vial:21**	*Injection Volume:*	**5 µL**
Injection Type:	**Unknown**	*Wavelength:*	**210 nm**
		Wavelength:	**254 nm**
Instrument Method:	**Trudel Gradient MeOH Phosphatpuffer pH 5**	*Flow rate:*	**1,0 ml/min**
Column:	**Zorbax Eclipe Plus C18 4,6 x 150mm 3,5µm**	*Column Temperatur:*	**50 °C**
Injection Date/Time:	**21.Jan.25 14:21**	*Pump Channel A:*	**5**
		Pump Channel B:	**95,0 Puffer pH 5**

Chromatogram

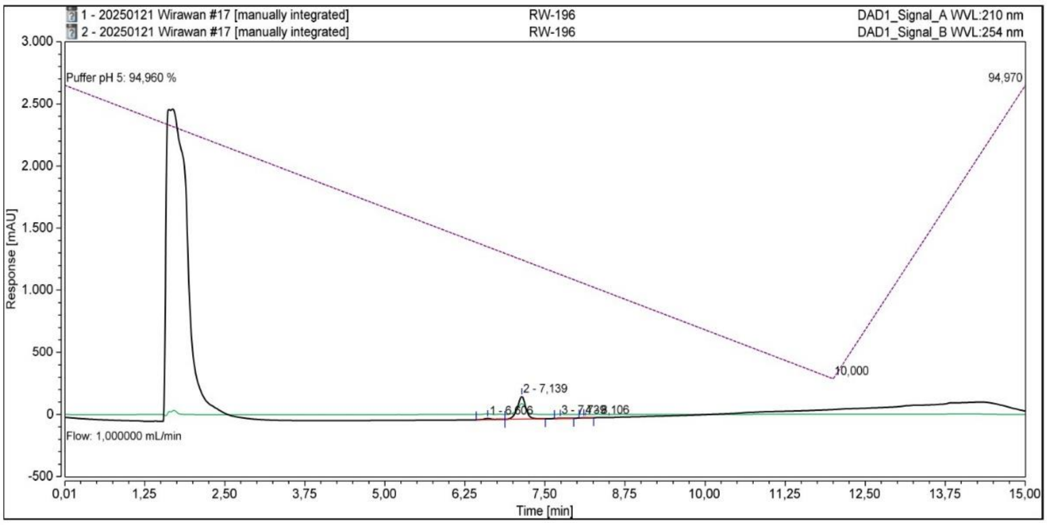

DAD1_Signal_A

No.	Retention Time min	Area mAU*min	Relative Area %
1	6,606	1,309	4,49
2	7,139	27,468	94,27
3	7,739	0,209	0,72
4	8,106	0,153	0,52
**Total:**		**29,138**	**100**

DAD1_Signal_B

No.	Retention Time min	Area mAU*min	Relative Area %
1	6,599	0,676	4,69
2	7,139	13,201	91,63
3	7,732	0,121	0,84
4	8,106	0,144	1,00
5	8,312	0,125	0,87
6	8,719	0,139	0,97
**Total:**		**14,407**	**100**

S3. Crystal structure data of oxazoline 45

X-ray experimental details and crystal structure of benzyl (*S*)-2-(2-(4-ethynylphenyl)-4,5-dihydrooxazol-4-yl)acetate (**45**).

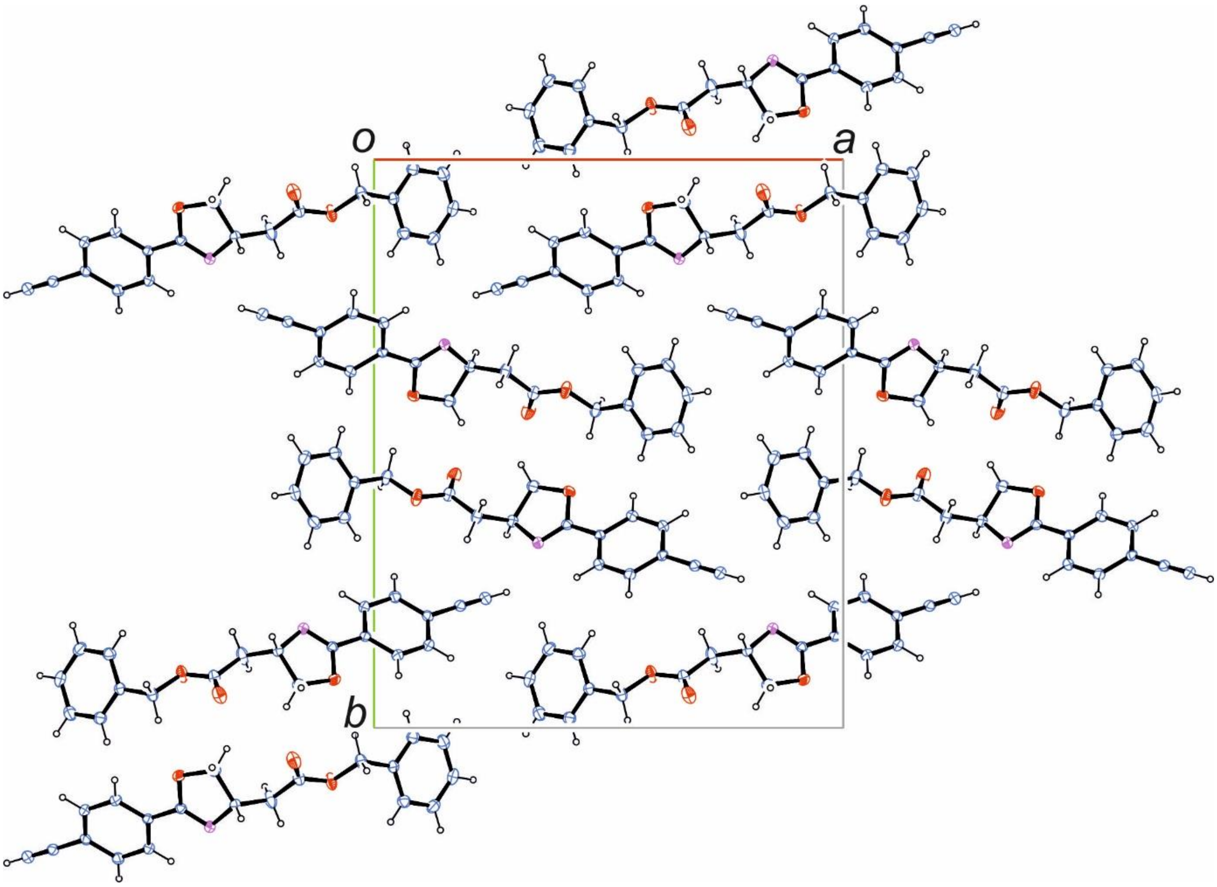

Experimental details

The X-ray intensity data of cv088 were measured on a Bruker D8 Venture TXS system equipped with a multilayer mirror monochromator and a Mo Kα rotating anode X-ray tube (λ = 0.71073 Å). The frames were integrated with the Bruker SAINT software package [47]. Data were corrected for absorption effects using the Multi-Scan method (SADABS) [48]. The structure was solved and refined using the Bruker SHELXTL Software Package [49]. All hydrogen atoms have been calculated in ideal geometry riding on their parent atoms. The figures have been drawn at the 25% ellipsoid probability level [50].

References:

47. Bruker. Bruker AXS Inc., Madison, Wisconsin, USA. SAINT (**2012**).

48. Sheldrick, G.M. University of Göttingen, Germany. SADABS (**1996**).

49. Sheldrick, G.M. SHELXT – Integrated space-group and crystal-structure determination. *Acta Cryst. A* **2015,** *A71, 3–8*. https://doi.org/10.1107/S2053273314026370.

50. Farrugia, L.J. WinGX and ORTEP for Windows: An update. *J. Appl. Cryst.* **2012**, *45, 849–854*. http://dx.doi.org/10.1107/S0021889812029111

net formula	$C_{20}H_{17}NO_3$	parameters	217
Mr/g mol−1	319.35	restraints	0
crystal size/mm	0.180 × 0.130 × 0.060	*$R(F_{obs})$*	0.0345
T/K	173.(2)	*$Rw(F^2)$*	0.0854
radiation	MoKα	*S*	1.105
diffractometer	'Bruker D8 Venture TXS'	shift/errormax	0.001
crystal system	orthorhombic	max electron density/e Å^{-3}	0.106
space group	'P 21 21 21'	min electron density/e Å^{-3}	−0.131
a/Å	17.2267(8)		
b/Å	20.8646(10)		
c/Å	4.5968(2)		
α/°	90		
β/°	90		
γ/°	90		
V/Å^3	1652.22(13)		
Z	4		
calc. density/g cm^{-3}	1.284		
μ/mm^{-1}	0.087		
absorption correction	Multi-Scan		
transmission factor range	0.95–0.99		
refls. measured	30396		
Rint	0.0420		
mean σ(I)/I	0.0202		
θ range	3.067–25.349		
observed refls.	2861		
x, y (weighting scheme)	0.0284, 0.4773		
hydrogen refinement	constr		
Flack parameter	0.0(4)		

refls in refinement	3037
